# Supplementary material for: Genomic insights into rapid speciation within the world’s largest tree genus Syzygium
Source: Nat Commun. 2022 Sep 12;13:5031. doi: 10.1038/s41467-022-32637-x (PMC9468008; doi:10.1038/s41467-022-32637-x)
Supplement: Supplementary file 3 — Description of Additional Supplementary Files [file 41467_2022_32637_MOESM3_ESM.pdf]

Description of Additional Supplementary Files

File name: Supplementary Data 1
Description: Species identity, collection location, and voucher information on the 292 *Syzygium* and outgroup accessions sequenced using Illumina HiSeqX technology.

File name: Supplementary Data 2
Description: Assembly and BUSCO information for the 292 Illumina-resequenced accessions.
