# Supplementary material for: Genomic insights into rapid speciation within the world’s largest tree genus Syzygium
Source: Nat Commun. 2022 Sep 12;13:5031. doi: 10.1038/s41467-022-32637-x (PMC9468008; doi:10.1038/s41467-022-32637-x)
Supplement: Supplementary file 1 — Supplementary Information [file 41467_2022_32637_MOESM1_ESM.pdf]

Genomic insights into rapid speciation within the world's largest tree genus *Syzygium*

Low *et al.*

Supplemental Information

A. Supplementary Notes

Supplementary Note 1. Description of the Smithsonian Institution (SI)-ForestGeo long-term forest dynamics plots.
One of the fundamental aspects in forest ecology is to study the structure and dynamics of tree communities *in situ*, and the functions and services of forest ecosystems. In order to pursue this long-term ecological research agenda, SI-ForestGeo has established a worldwide network of permanent forest dynamics plots ranging in size from 2-ha to 120-ha[1]. Trees in the permanent plots ≥ 1 cm diameter at breast height have been tagged and identified and re-censusing is carried out for these plots every five years to document species gained or lost. In this study, we sampled as many identified and undetermined *Syzygium* spp. as possible from the two long-term ecological plots listed below.

(i) Bukit Timah Nature Reserve (BTNR), Singapore
The Bukit Timah Nature Reserve is a 1.64 km^2 nature reserve situated in the central region of Singapore. The name is derived from the highest hill in Singapore, Bukit Timah (about 164 m at sea level), which is also located within the nature reserve. Prior to its conversion to a nature reserve, part of the site was an active granite quarry until the mid 1900s. Today, Bukit Timah Nature Reserve has the last remaining patch of intact hill-dipterocarp forest in Singapore and is an important site for preserving and protecting native flora and fauna. A total of 34 *Syzygium* species are recorded to occur in the nature reserve, but only 23 species are enumerated in the 2-ha primary forest and 2-ha secondary forest plots[2].

(ii) Danum Valley Conservation Area (DVCA), Lahad Datu, Sabah, Malaysia
The Danum Valley Conservation Area is located in the interior of the Malaysian state of Sabah, on its east coast; it covers an area of 438 km^2 encompassing undisturbed lowland dipterocarp forest. A total of 24 *Syzygium* spp. is recorded from the 50-ha ForestGEO plot, although some taxa require further attention due to the lack of flowering and fruiting materials needed for species identification.

Supplementary Note 2. Morphological characters examined.
States were coded for three morphological characters – specifically (i) inflorescence habit (erect vs. pendent), (ii) shedding fused corolla present as a true calyptra, a pseudocalyptra, vs. corolla free at anthesis, and (iii) mature fruit colour (green, white or cream, black, pink, purple, red, brown, orange, yellow, blue, or grey) – were gathered from living material, herbarium specimens, published flora accounts, and species protologues.

(i) Inflorescence habit of *Syzygium* can in general be categorised into two groups based on the orientation of the inflorescences being displayed and presented on branchlets. The group of taxa with erect inflorescences generally have inflorescences presented in an upright position, possibly influenced by pollination syndromes. The other group of taxa have pendulous inflorescences.

(ii) The *Syzygium* perianth can be divided into two main categories, the first category based on morphological traits of the calyx, and the other based on the corolla. In general, calyx lobes are free (Supplementary Fig. 132; b1, b2, b1 and b2), but they can also be fused in the bud and eventually split free into equal portions along a suture as the stamens expand (Supplementary Fig. 132; d1 and d2: *S. fibrosum*). Calyx lobes have also been recorded as fused into a true calyptra that tears irregularly as the stamens expand (Supplementary Fig. 132; a1 and s2: *S. paradoxum*). Apart from the calyx, petals have been recorded to be free, spreading, and persistent, such as in *S. grande* (Supplementary Fig. 132; c1 and c2). However, the corolla can also form a pseudocalyptra, in which the petals are tightly folded above the stamens to form a cap that tears along the attachment at the base, the cohered petals shedding like a calyptra at anthesis (Supplementary Fig. 132; b1 and b2: *S. cumini*).

(iii) Mature fruit colour of *Syzygium* is extremely diverse and broad in colour spectrum, ranging from very bright hues to dark-coloured fruits that maximise visual detection by specific dispersers (Figs. 4d-e). It has been shown that fruits dispersed by birds tend to be in the red part of the spectrum, while mammalian dispersed fruits display the green part of the spectrum.

Supplementary Note 3. Additional information on the transcriptome assembly process.
We first ran Transabyss with multiple kmer options to reliably assemble the common transcripts and capture the rare transcripts. For the S1 RNASeq library, the average N50 value across all kmers was 1,500 bp, and the average BUSCO score across kmers was 88.5%. The average number of transcripts of size >/=500 bp for each kmer value was 56,400. Regarding Trinity, we could only select kmer values between 25 and 32. Hence, we decided to go with one iteration and used the default kmer value 25. Trinity, with default kmer 25, provided a substantially better N50 value (2,075 bp), a higher BUSCO score of 91.9%, and yielded 62,744 transcripts of size >= 500 bp. We combined all the transcripts using EvidentialGene and assessed the contribution of Transabyss and Trinity to the final transcriptome. From the final count of 57,738 high quality transcripts, 13,681 transcripts came from Trinity, clearly indicating that the default kmer setting in Trinity captured more complete (and likely reliable) transcripts compared to most of the higher kmers from Transabyss.

We provided the least weight to the self-training gene predictor genemark-es at value 3 since the predictions were mostly fragmented. The next highest weight was provided to the homology-based gene predictor GeMoMa at value 4. Higher weights were provided to *ab initio* gene predictor braker with value 6 for RNASeq library S1, 5 for S2 and 5 for S3 based on higher prediction quality compared to genemark-es and GeMoMa. As recommended by the tool author, we provided the maximum weights to evidence from PASA with the alignments receiving value 7 for RNASeq library S1, 8 for S2 and 9 for S3 while the predicted ORFs received value 10 for S1, 11 for S2 and 12 for S3.

B. Supplementary Figures

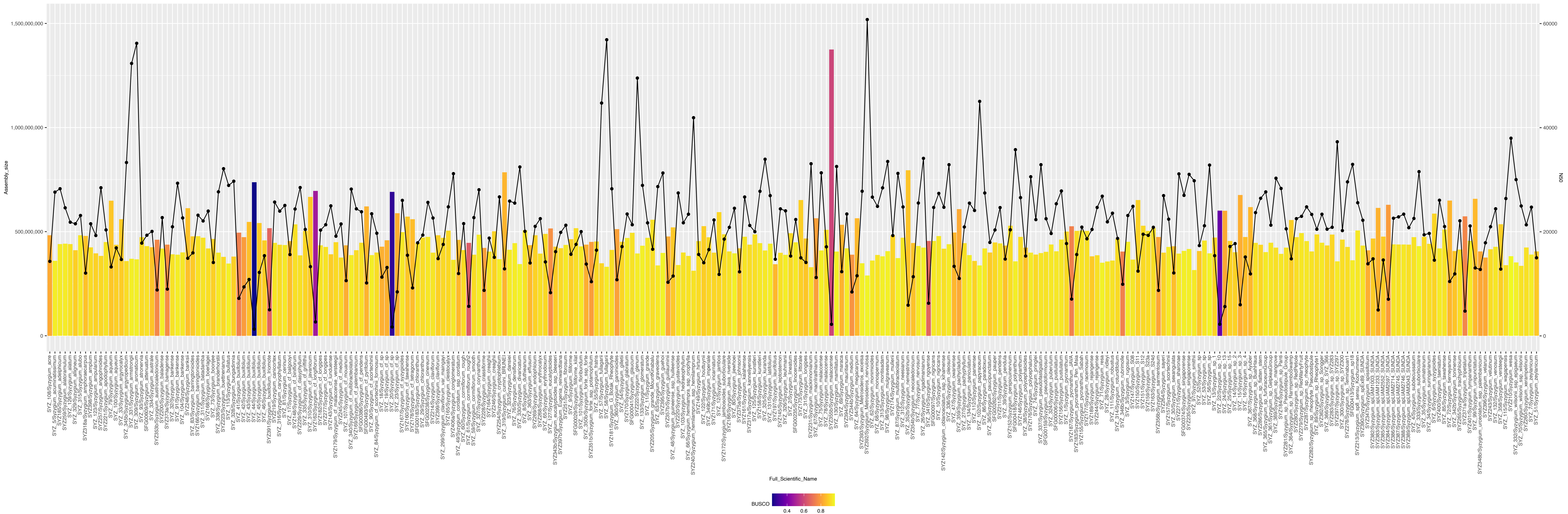

Supplementary Figure 1. Resequenced MaSuRCA assemblies. These are coloured by BUSCO completeness; left y-axis (histogram) is assembly size, right y-axis (black line) is contig N50.

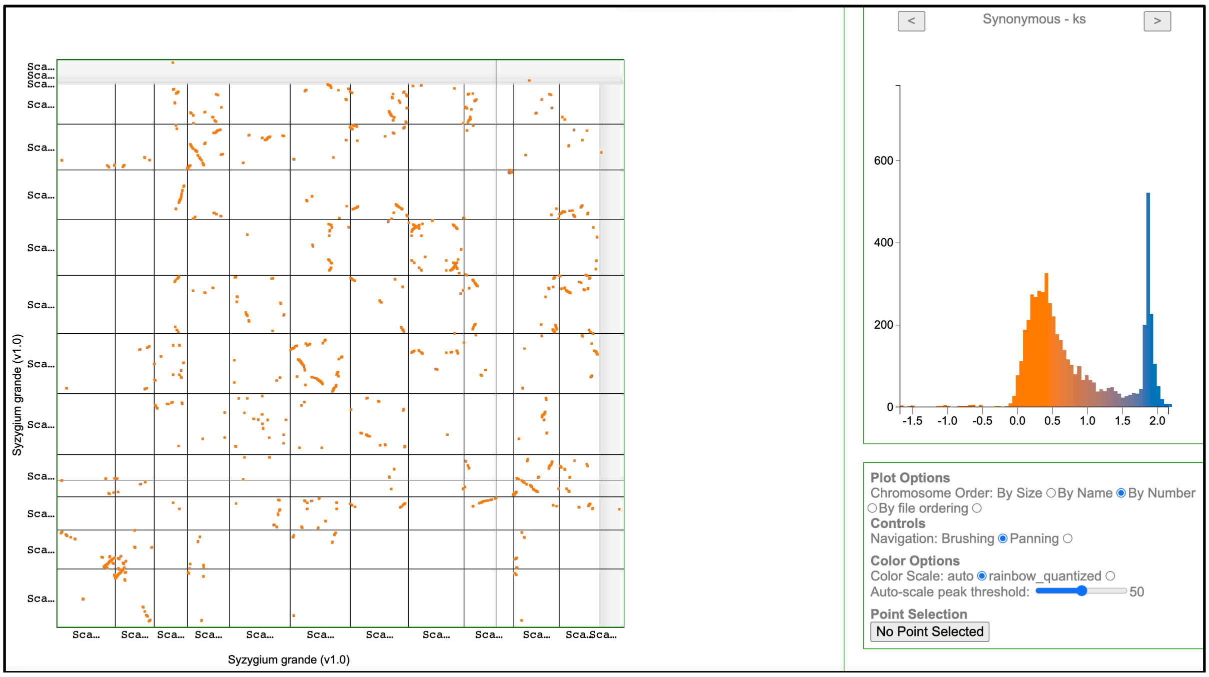

Supplementary Figure 2. A SynMap self:self syntenic dotplot (left) for *Syzygium grande* with coloration by Ks (histogram) reveals internal paralogy suggesting one ancestral WGD in *Syzygium* following the *gamma* paleohexaploidy event. The analysis can be regenerated at https://genomevolution.org/r/1gh12.

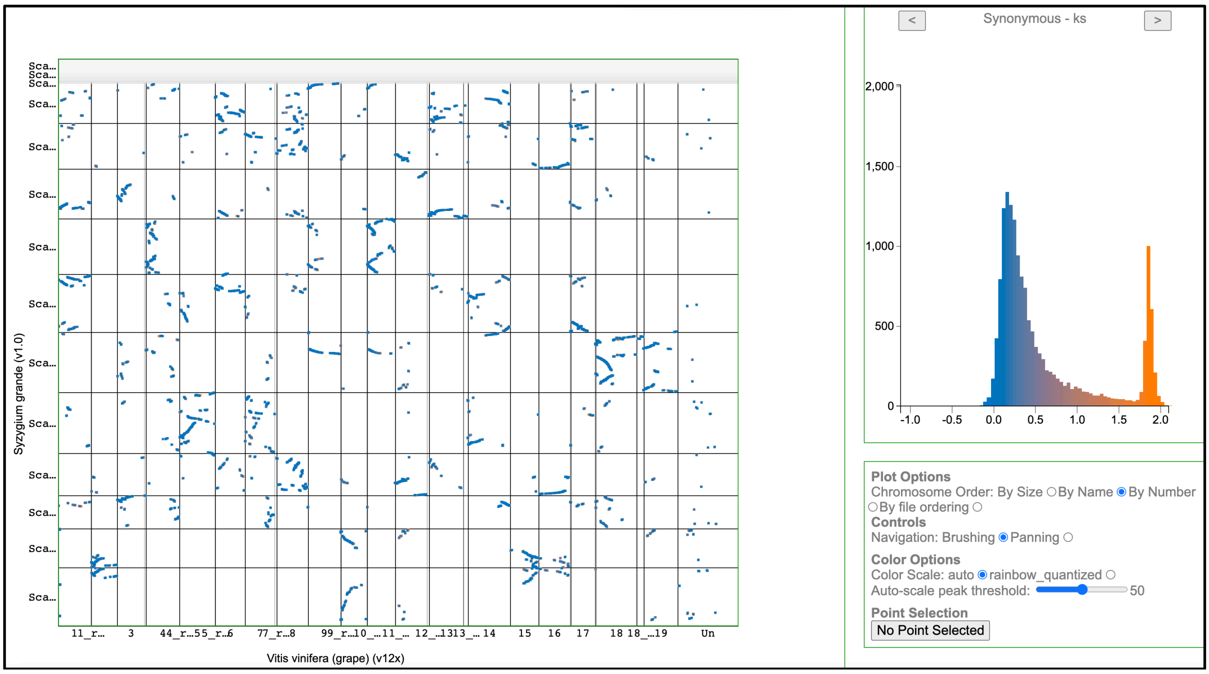

Supplementary Figure 3. A SynMap syntenic dotplot (left) for *Syzygium grande*:*Vitis vinifera* with coloration by Ks (histogram) reveals a 2:1 relationship, indicating one WGD in *Syzygium* following its species split with Vitis, which otherwise only contains the ancient *gamma* hexaploidy event. The medium-blue blocks are 2:1 syntenic orthologs, and the more dispersed and fractionated cyan blocks are syntenic paralogs dating from the ancient *gamma* event. The orange peak represents irrational Ks values from poor CDS alignments. The analysis can be regenerated at https://genomevolution.org/r/1i4rm.

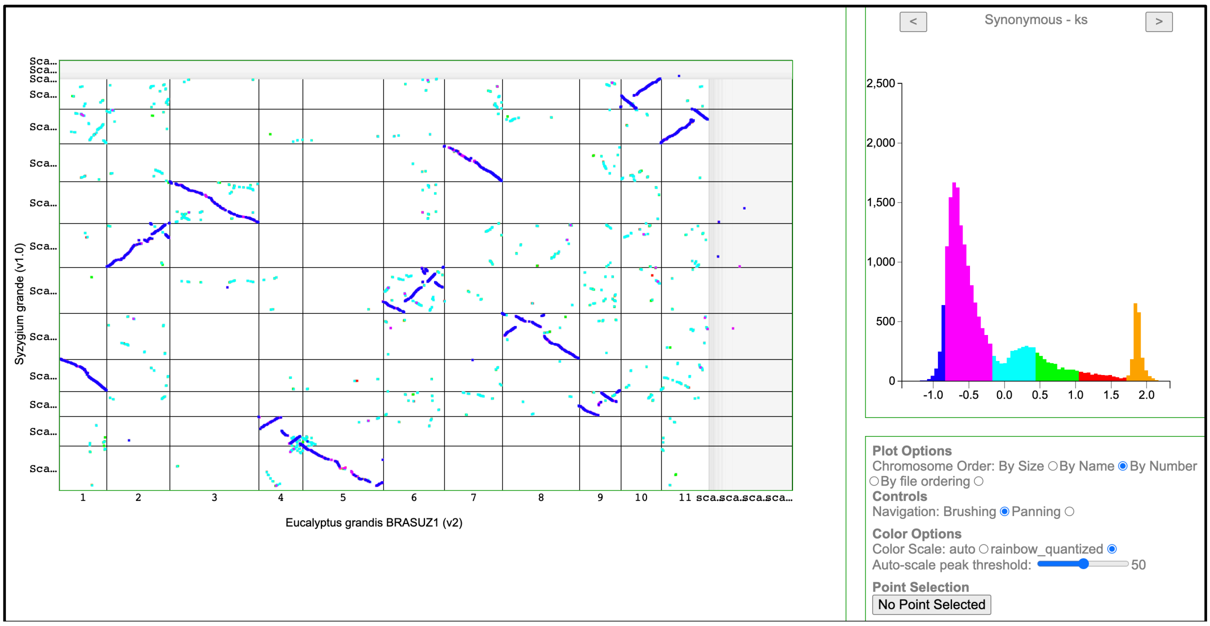

Supplementary Figure 4. A SynMap syntenic dotplot (left) of *Syzygium grande* against *Eucalyptus grandis* with coloration by Ks (histogram). The violet blocks are 1:1 syntenic orthologs, and the more dispersed and fractionated cyan blocks are syntenic paralogs. The orange peak represents irrational Ks values from poor CDS alignments. The analysis can be regenerated at https://genomevolution.org/r/1i4rm.

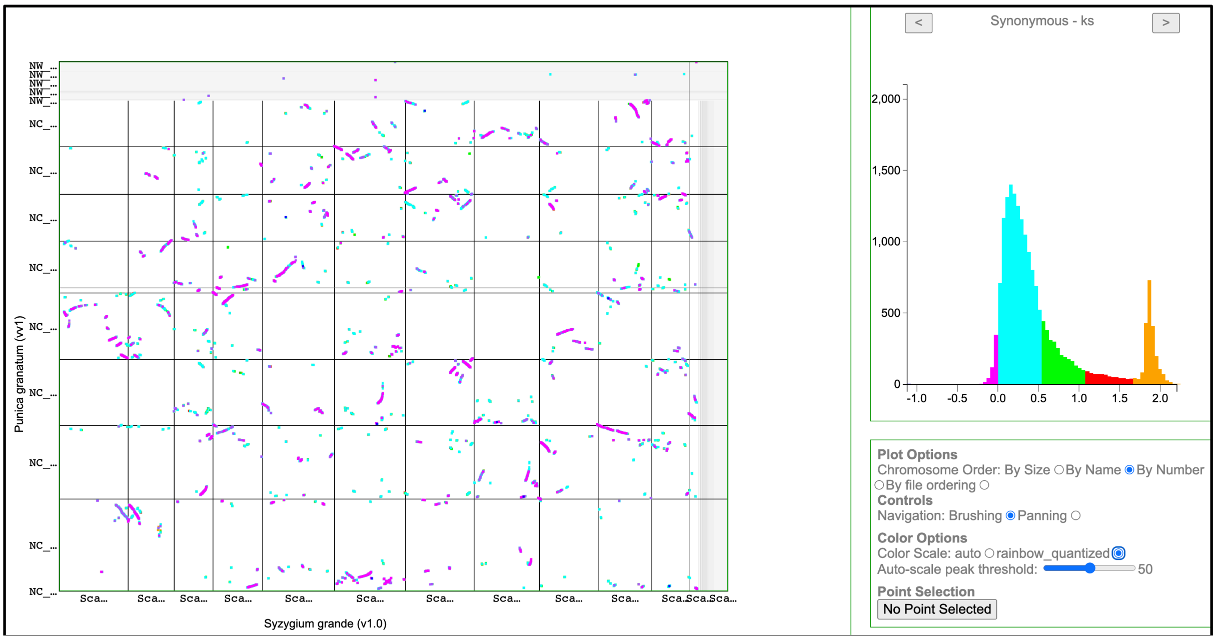

Supplementary Figure 5. SynMap syntenic dotplot (left) of *Syzygium grande* against *Punica granatum* with coloration by Ks. The pink blocks are 1:1 syntenic orthologs, and the more dispersed and fractionated cyan blocks are syntenic paralogs. The orange peak represents irrational Ks values from poor CDS alignments. The analysis can be regenerated at https://genomevolution.org/r/1hxo0.

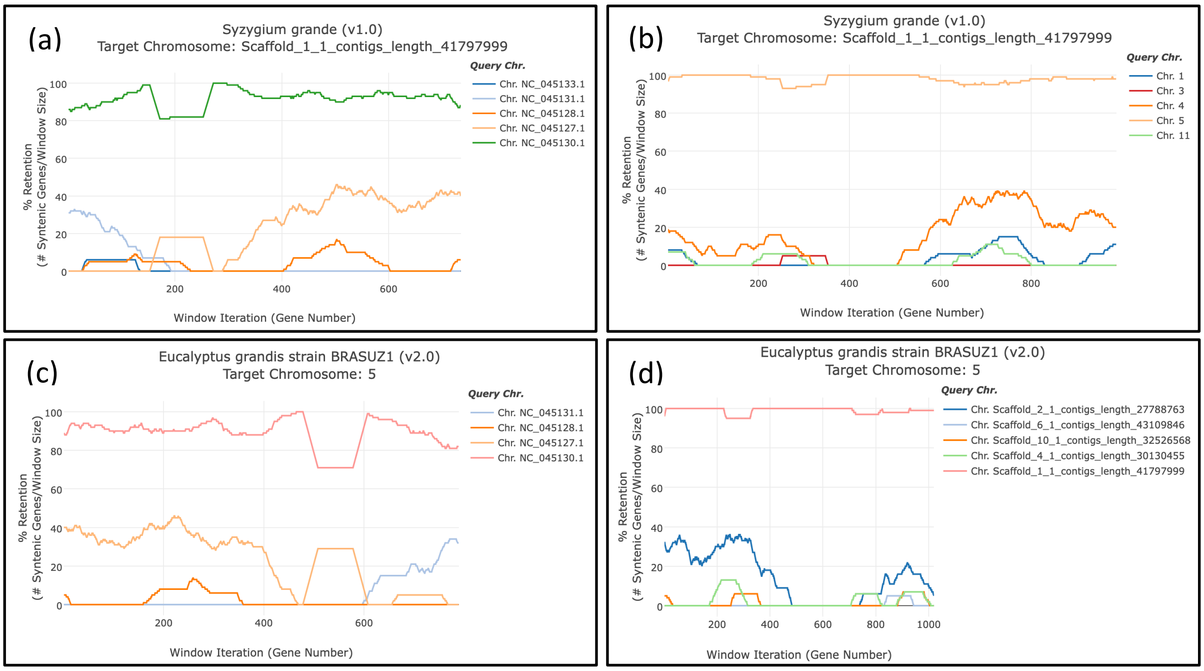

Supplementary Figure 6. FractBias mappings of the (a) *Punica granatum* and (b) *Eucalyptus grandis* genomes against *Syzygium grande*, and of (c) *Punica granatum* and (d) *Syzygium grande* against *Eucalyptus grandis*, reveal the same syntenic depths and fractionation (percent gene retention) patterns between all pairwise genomic block comparisons, regardless of reference genome, demonstrating that any underlying whole genome duplications among Lythraceae and Myrtaceae represent the same polyploidy events. All comparisons show orthologous syntenic blocks with around 80-100% gene retention, and paralogous syntenic blocks that reach ~40% gene retention. These analyses can be regenerated at https://genomevolution.org/r/1kpma, https://genomevolution.org/r/1kkx4, https://genomevolution.org/r/1kpm9, and https://genomevolution.org/r/1kpmb, respectively.

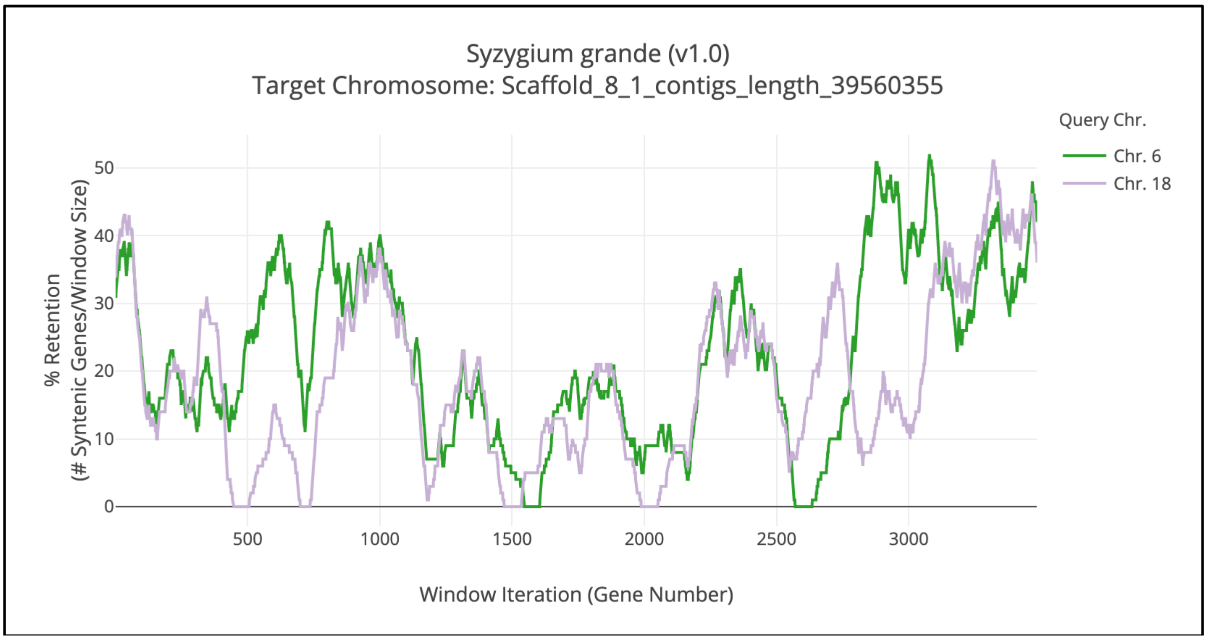

Supplementary Figure 7. FractBias mapping of the *Populus trichocarpa* genome against *Syzygium grande* shows a 2:2 relationship between the species, confirming independent polyploidy events in the two lineages. This analysis can be regenerated at https://genomevolution.org/r/1ig9q.

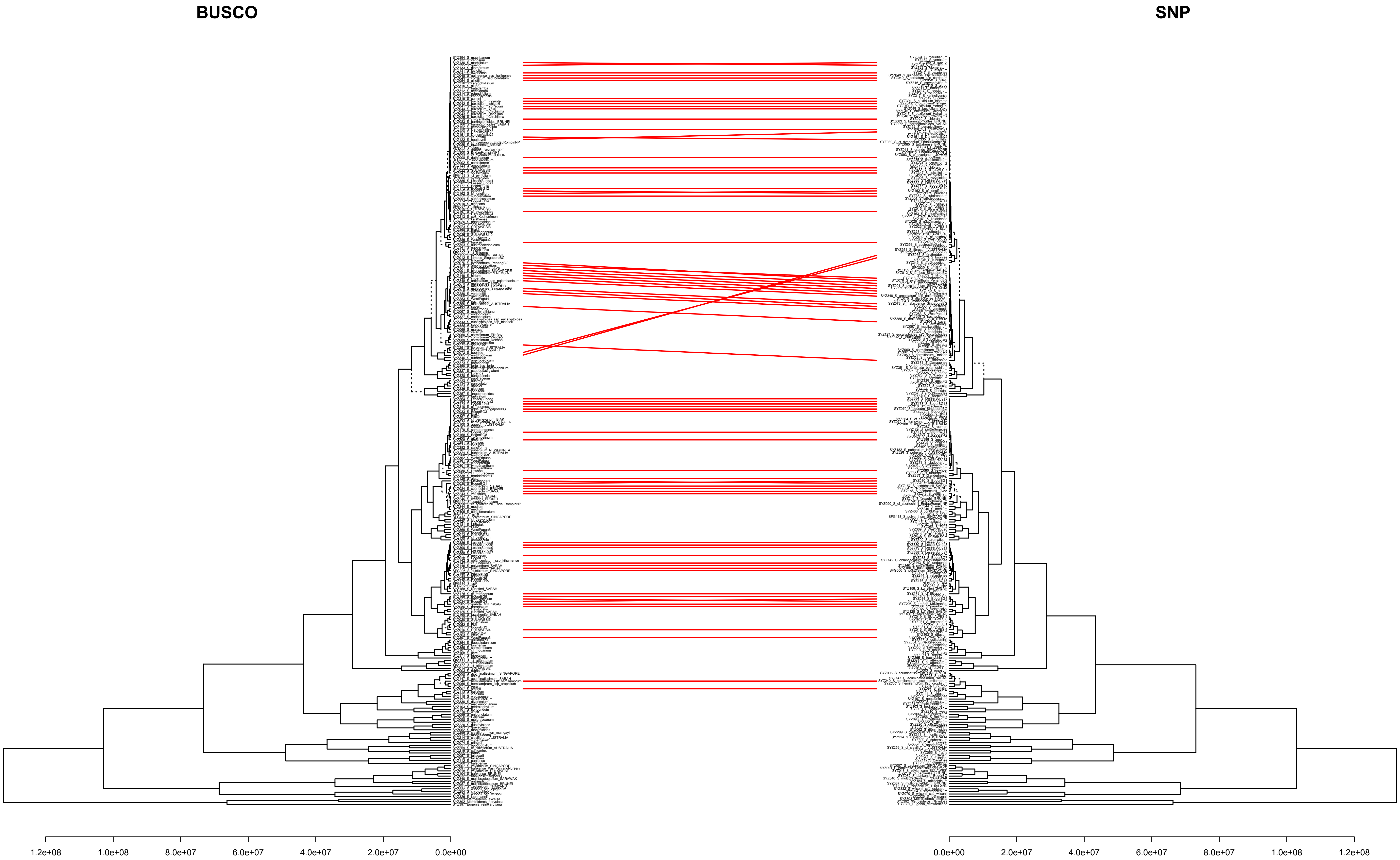

Supplementary Figure 8. Tanglegram comparing the BUSCO- and SNP-based phylogenetic trees. The R package dendextend (version 3.5.1) was used. Specifically, the tanglegram() function was used. Branches that contribute to unique sub-trees are marked with black dotted lines; incongruent relationships are shown with red lines. The BUSCO species tree and genome-wide SNP tree are both well-resolved, with robust support throughout. Five major clades, *Syzygium* subgenus *Acmena*, *S.* subgenus *Perikion*, *S.* subgenus *Sequestratum*, *S.* subgenus *Syzygium*, and a yet-to-be-named clade (*S.* cf. *attenuatum-rugosum*-SULAWESI2 clade), are consistently present in both trees. Minor discordances between these two phylogenies are present, but these do not affect the positions of the five clades recognised in this study.

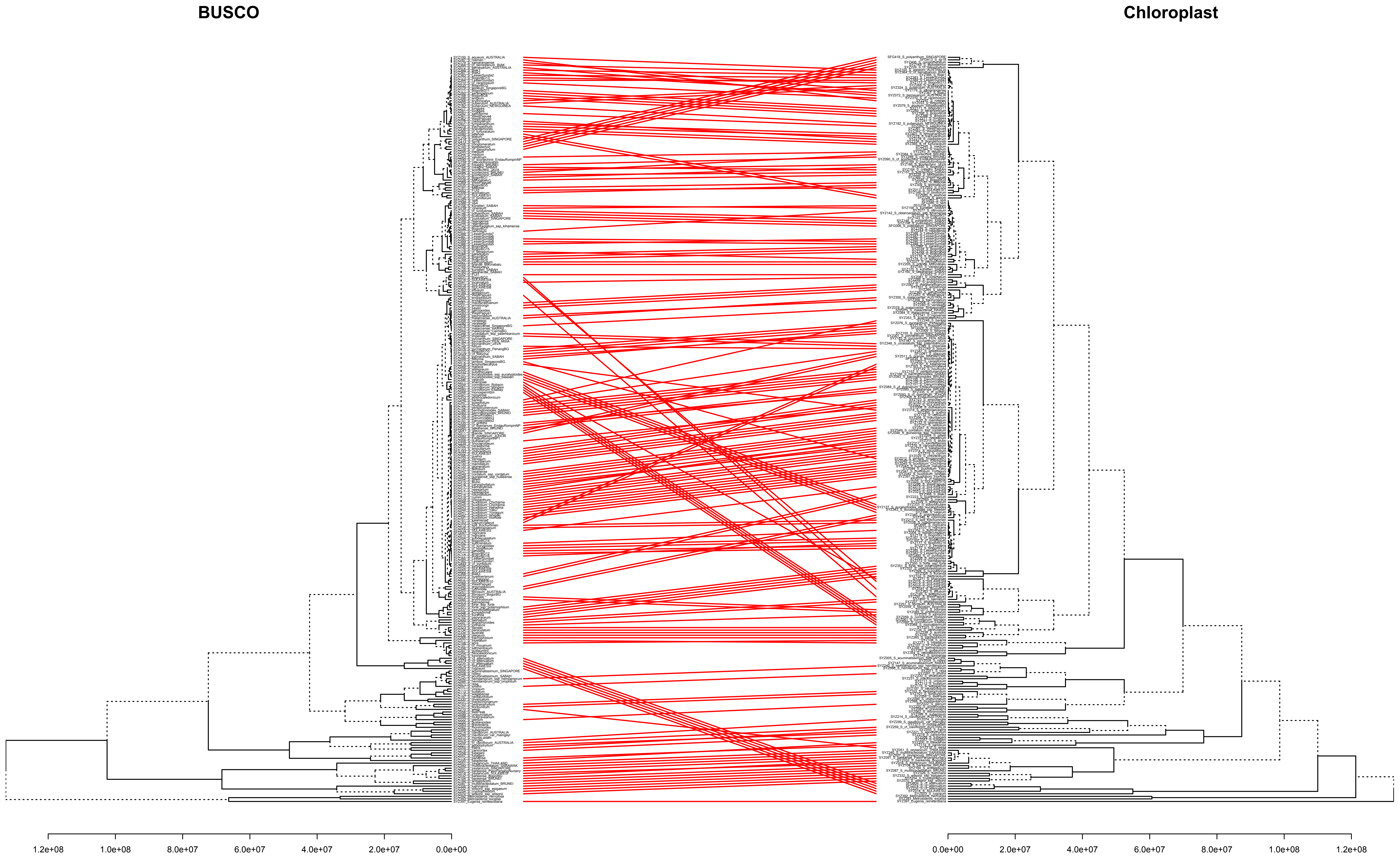

Supplementary Figure 9. Tanglegram comparing the plastome- and BUSCO-based phylogenetic trees. The R package dendextend (version 3.5.1) was used. Specifically, the tanglegram() function was used. Branches that contribute to unique sub-trees are marked with black dotted lines; incongruent relationships are shown with red lines. These results show that *Syzygium* phylogeny inferred from plastome data is well-resolved, but still with some internal and external branches having low bootstrap supports distributed throughout the tree. Five major clades are recognised on our plastome tree, namely *Syzygium* subgenus *Acmena*, *S.* subgenus *Perikion*, *S.* subgenus *Sequestratum*, *S.* subgenus *Syzygium*, and a yet-to-be-named clade (*S.* cf. *attenuatum-rugosum*-SULAWESI2 clade), while the placement of *Syzygium wesa* is poorly supported, although robustly nestled in the *S.* subgenus *Acmena* clade in both the BUSCO genes and genome-wide SNP trees. The most significant finding is that relationships within *Syzygium* subgenus *Syzygium*, the largest of the five clades recognised, are well-resolved. One disparate placement is the position of *Syzygium jambos*; in the plastome tree, *S. jambos* is embedded in a clade otherwise comprised of all *Syzygium buxifolium* taxa, whereas in the BUSCO genes and genome-wide SNP trees, *S. jambos* is sister to *S. filiforme* in a small clade that comprised of 11 other *Syzygium* individuals. One possible explanation for the incongruent placements for *S. jambos* and *S. wesa* on the plastome tree against the nuclear inferred trees could be chloroplast capture through ancient hybridisation events, or possibly even deep ILS.

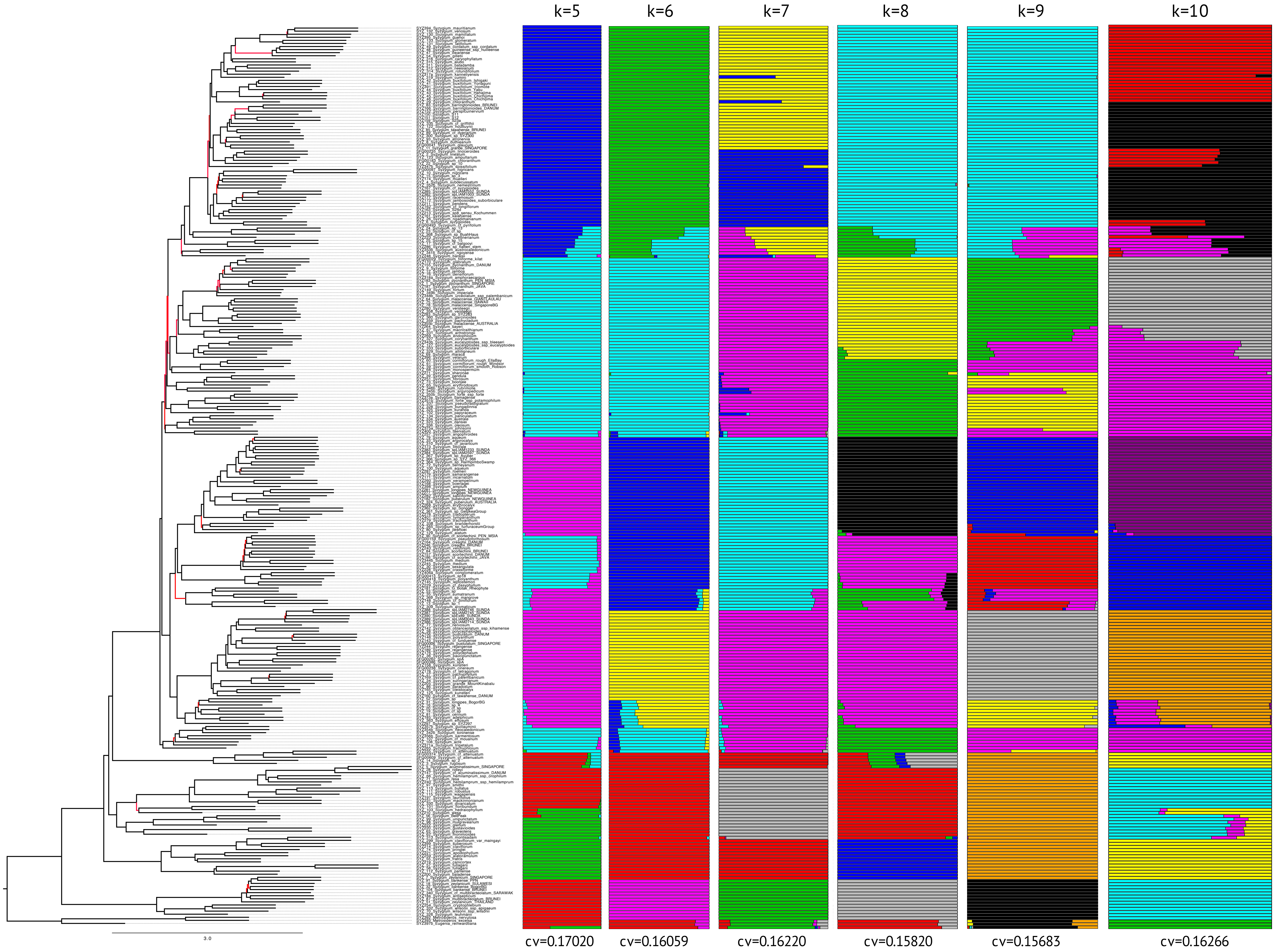

Supplementary Figure 10. ADMIXTURE results for *K* = 5-10 as shown with the BUSCO species tree. Colouration follows *K* clusters.

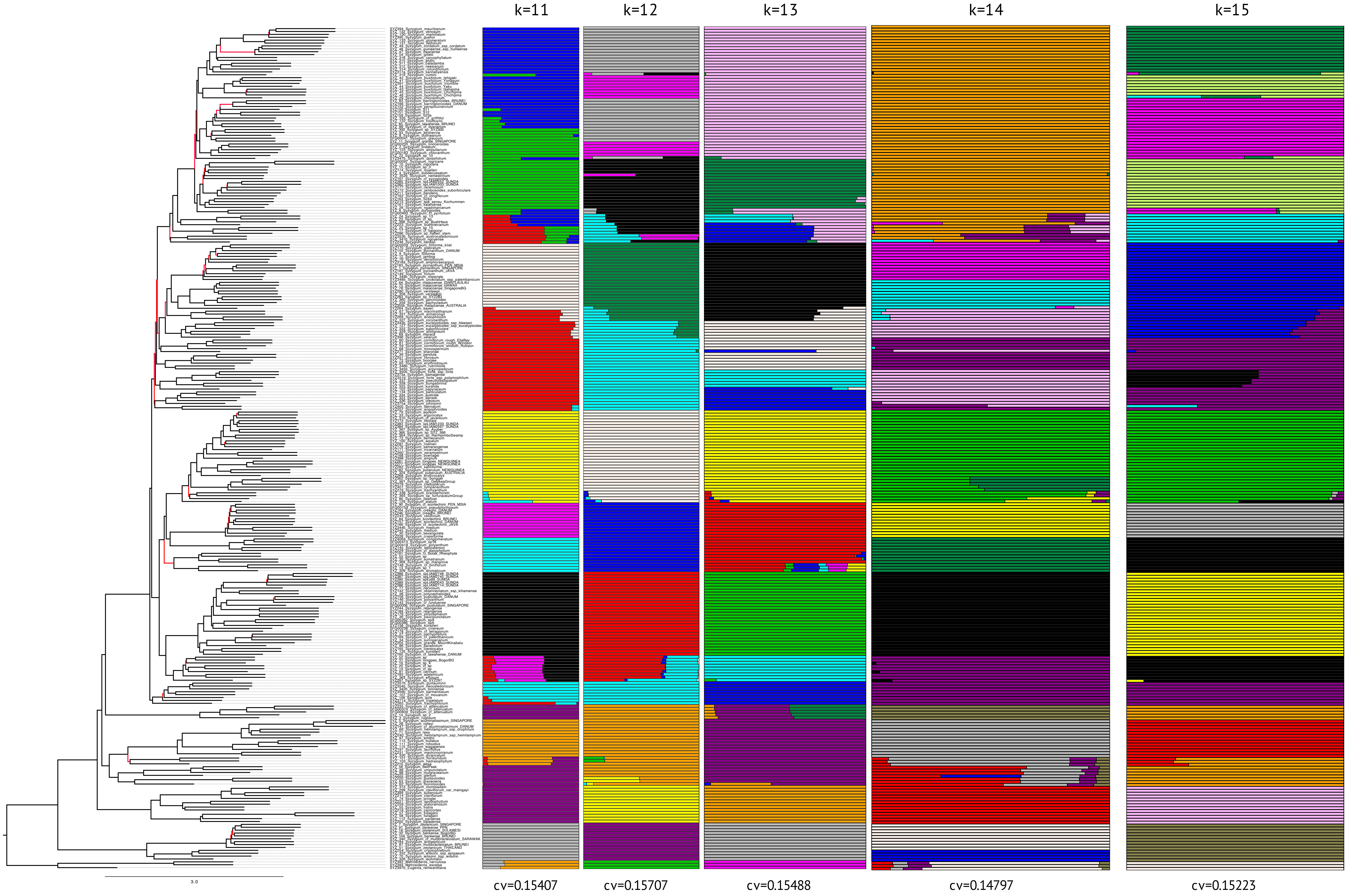

Supplementary Figure 11. ADMIXTURE results for *K* = 11-15 as shown with the BUSCO species tree. Colouration follows *K* clusters.

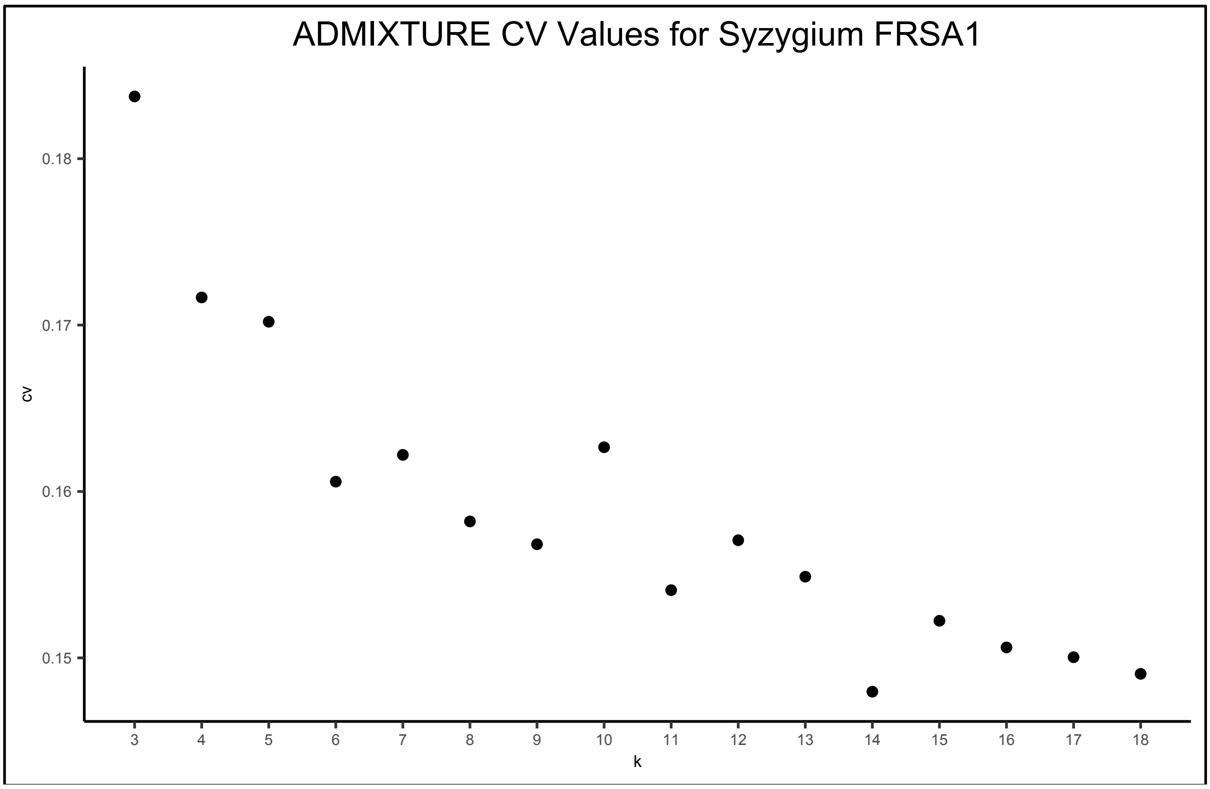

Supplementary Figure 12. ADMIXTURE cross-validation scores for dataset FRSA1. This analysis indicates that *K*=14 is the best supported number of clusters.

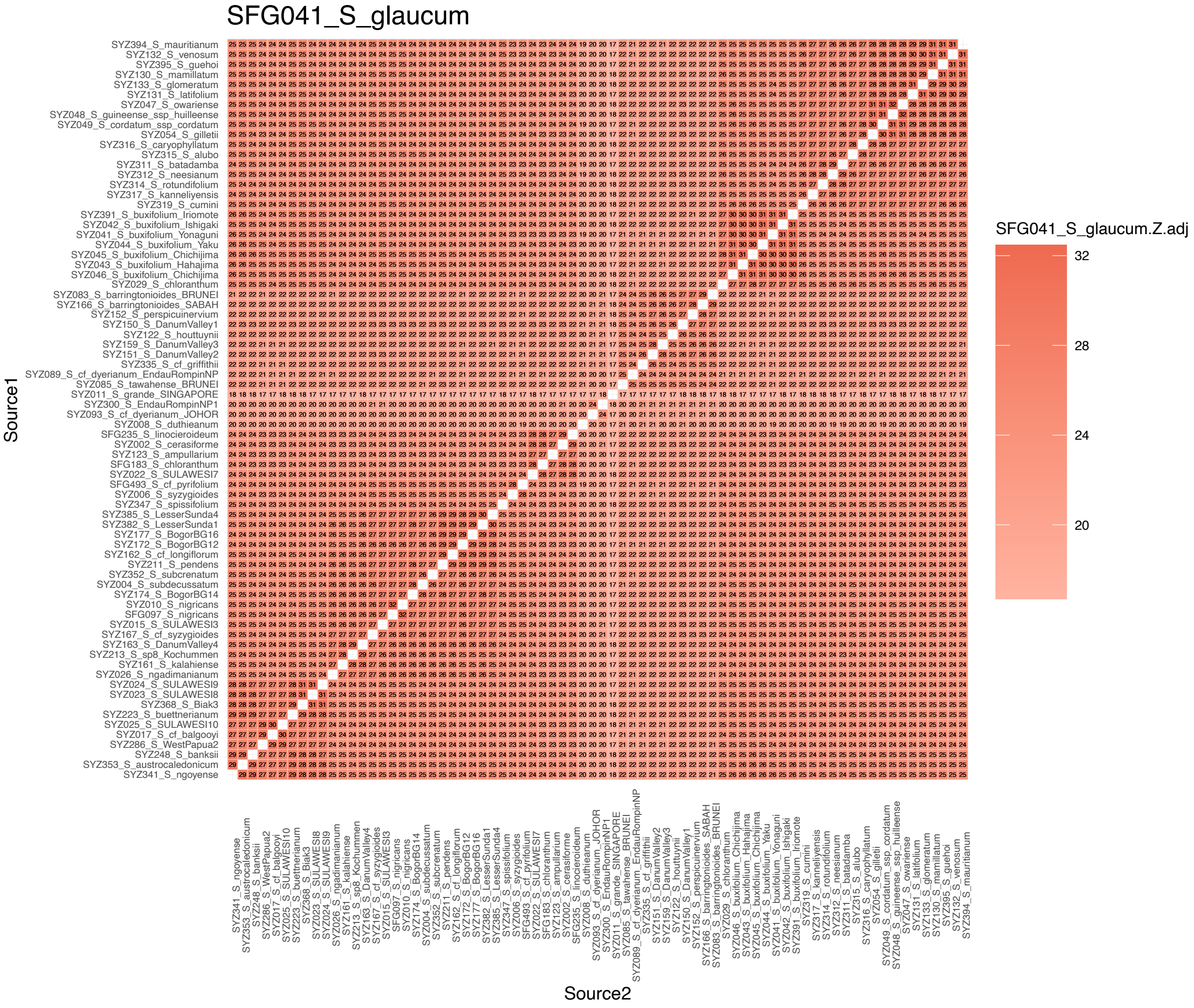

Supplementary Figure 13. F3 heatmap for SFG041_S_glaucum as target, with other members of the *Syzygium grande* group as Source1 or Source2. Z-scores adjusted for multiple tests are depicted.

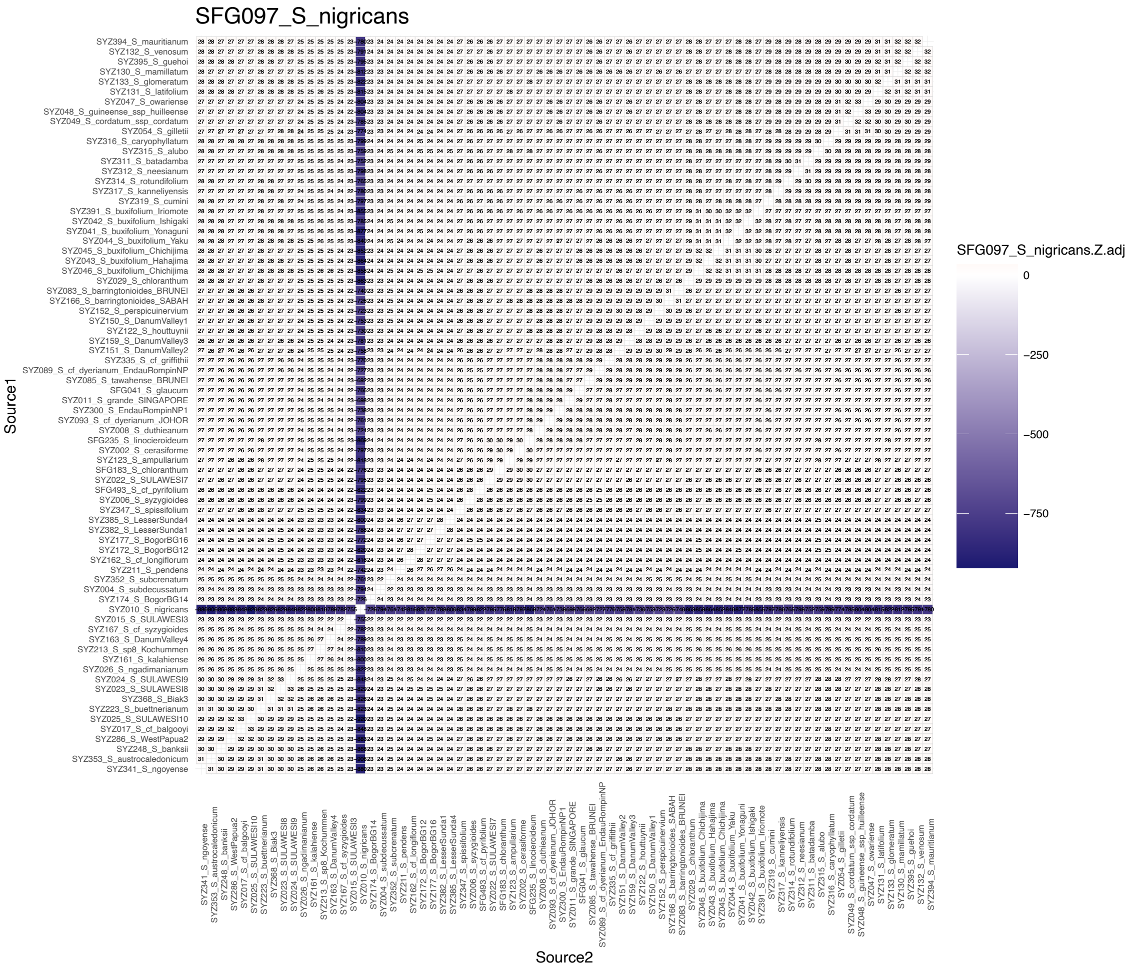

Supplementary Figure 14. F3 heatmap for SFG097_S_nigricans as target, with other members of the *Syzygium grande* group as Source1 or Source2. Z-scores adjusted for multiple tests are depicted.

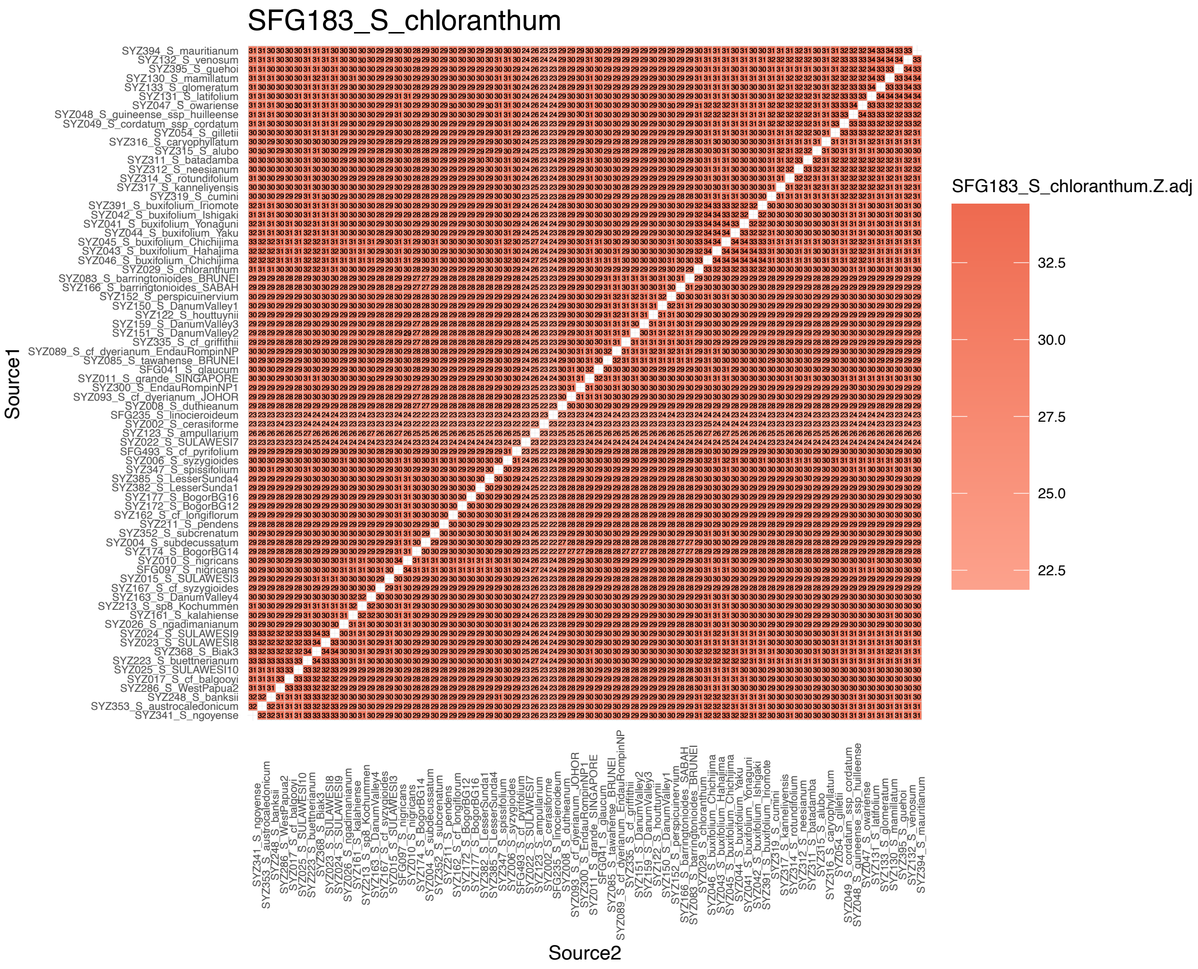

Supplementary Figure 15. F3 heatmap for SFG183_S_chloranthum as target, with other members of the *Syzygium grande* group as Source1 or Source2. *Z*-scores adjusted for multiple tests are depicted.

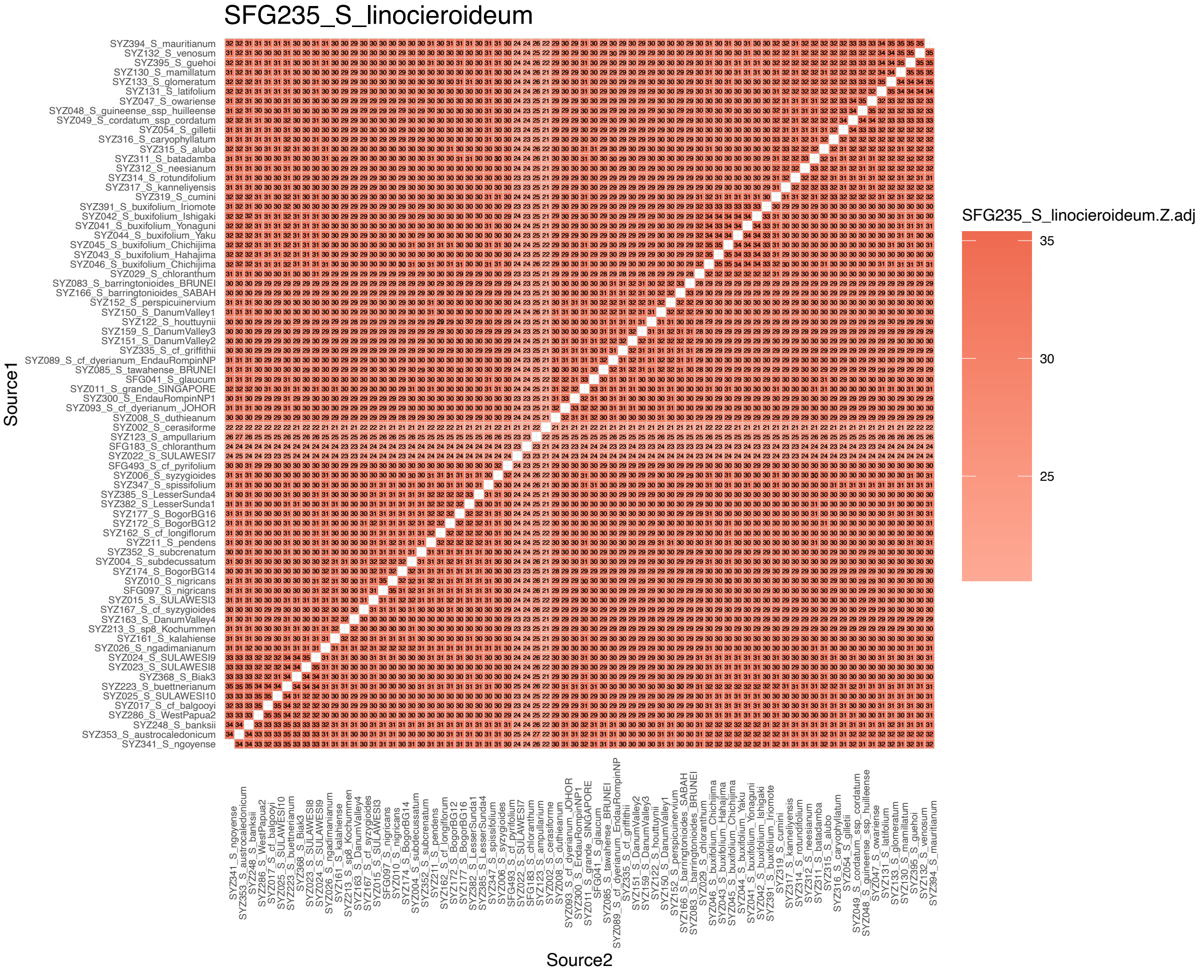

Supplementary Figure 16. F3 heatmap for SFG235_S_linocieroideum as target, with other members of the *Syzygium grande* group as Source1 or Source2. *Z*-scores adjusted for multiple tests are depicted.

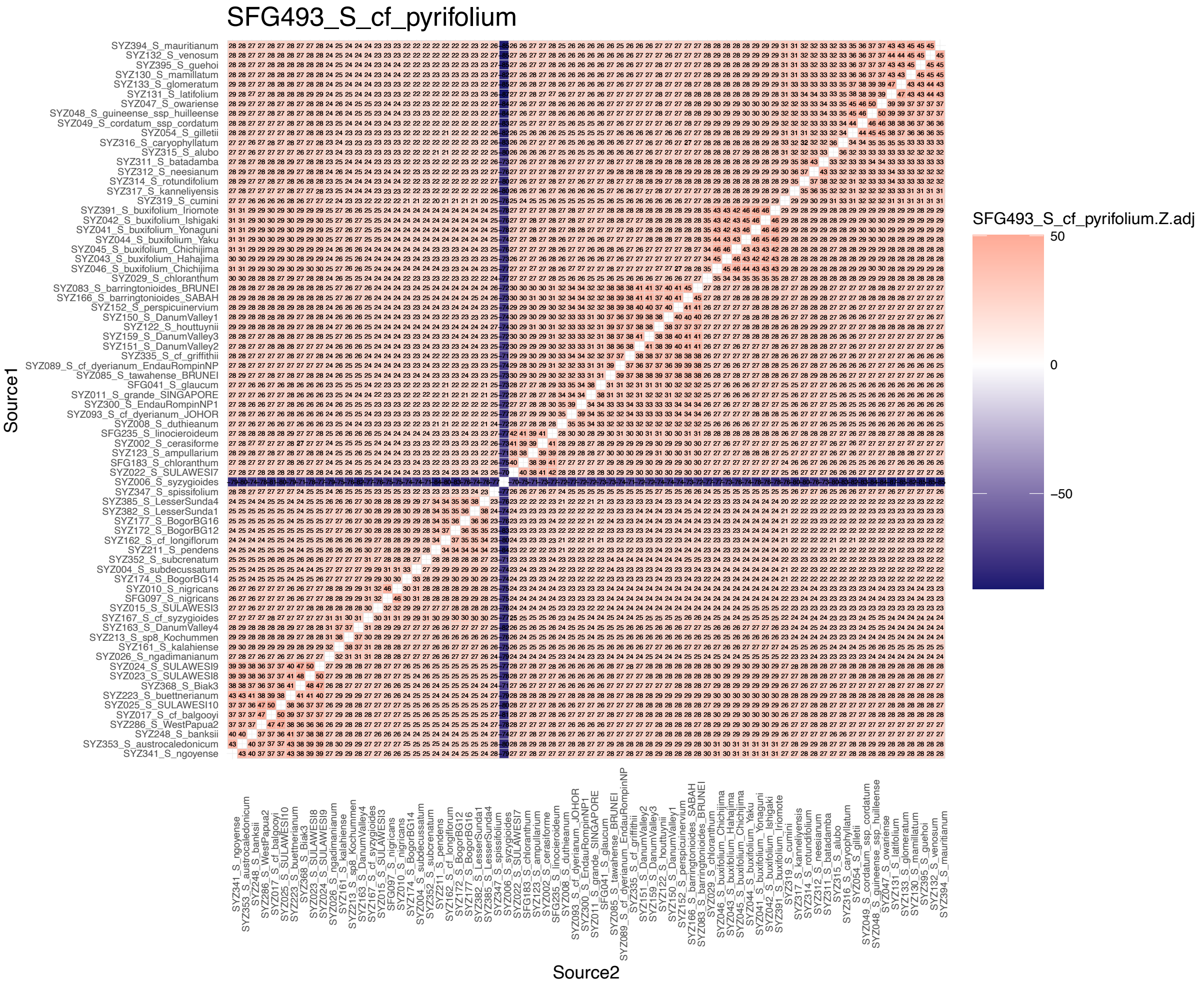

Supplementary Figure 17. F3 heatmap for SFG493_S_cf_pyrifolium as target, with other members of the *Syzygium grande* group as Source1 or Source2. *Z*-scores adjusted for multiple tests are depicted.

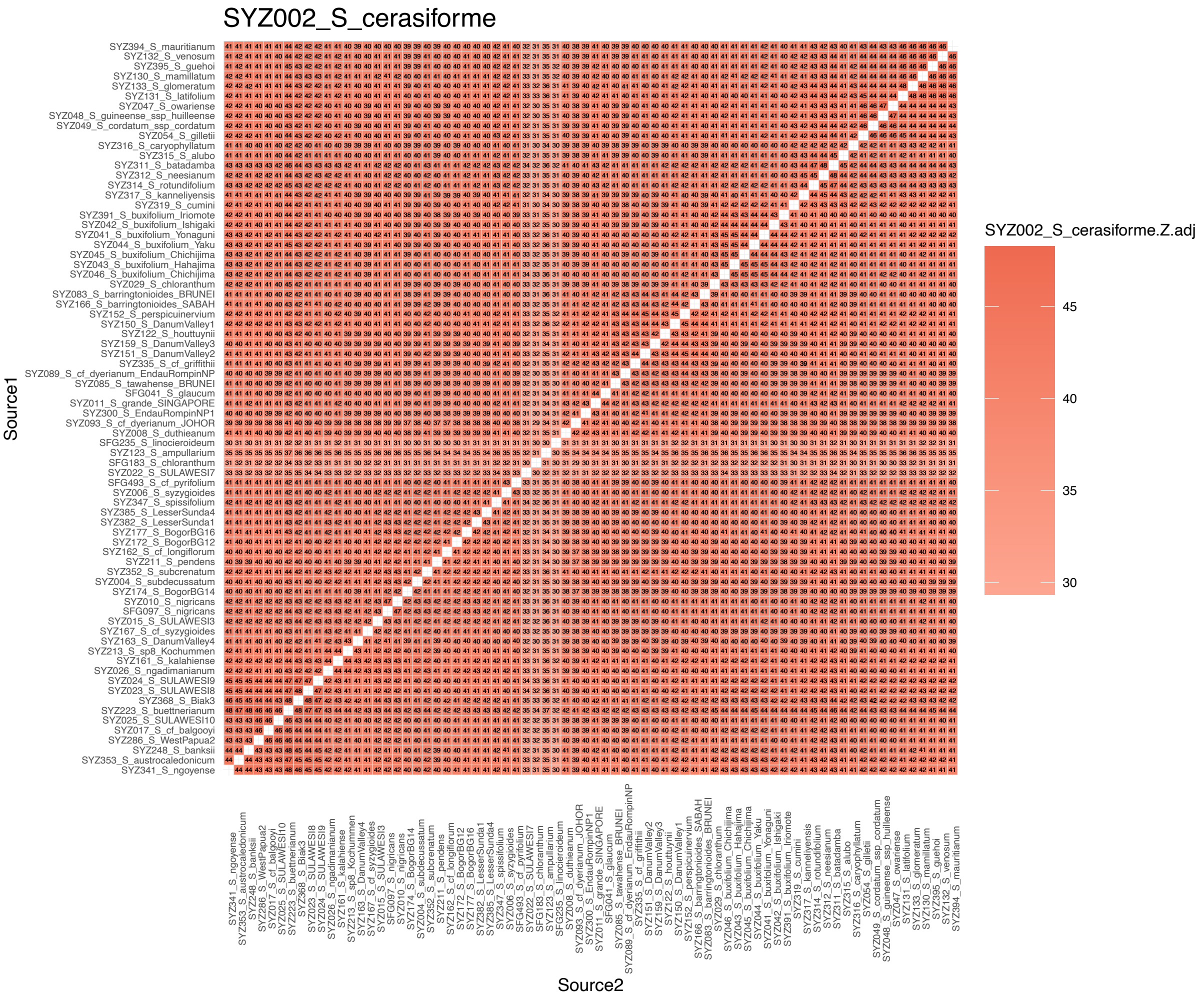

Supplementary Figure 18. F3 heatmap for SYZ002_S_cerasiforme as target, with other members of the *Syzygium grande* group as Source1 or Source2. Z-scores adjusted for multiple tests are depicted.

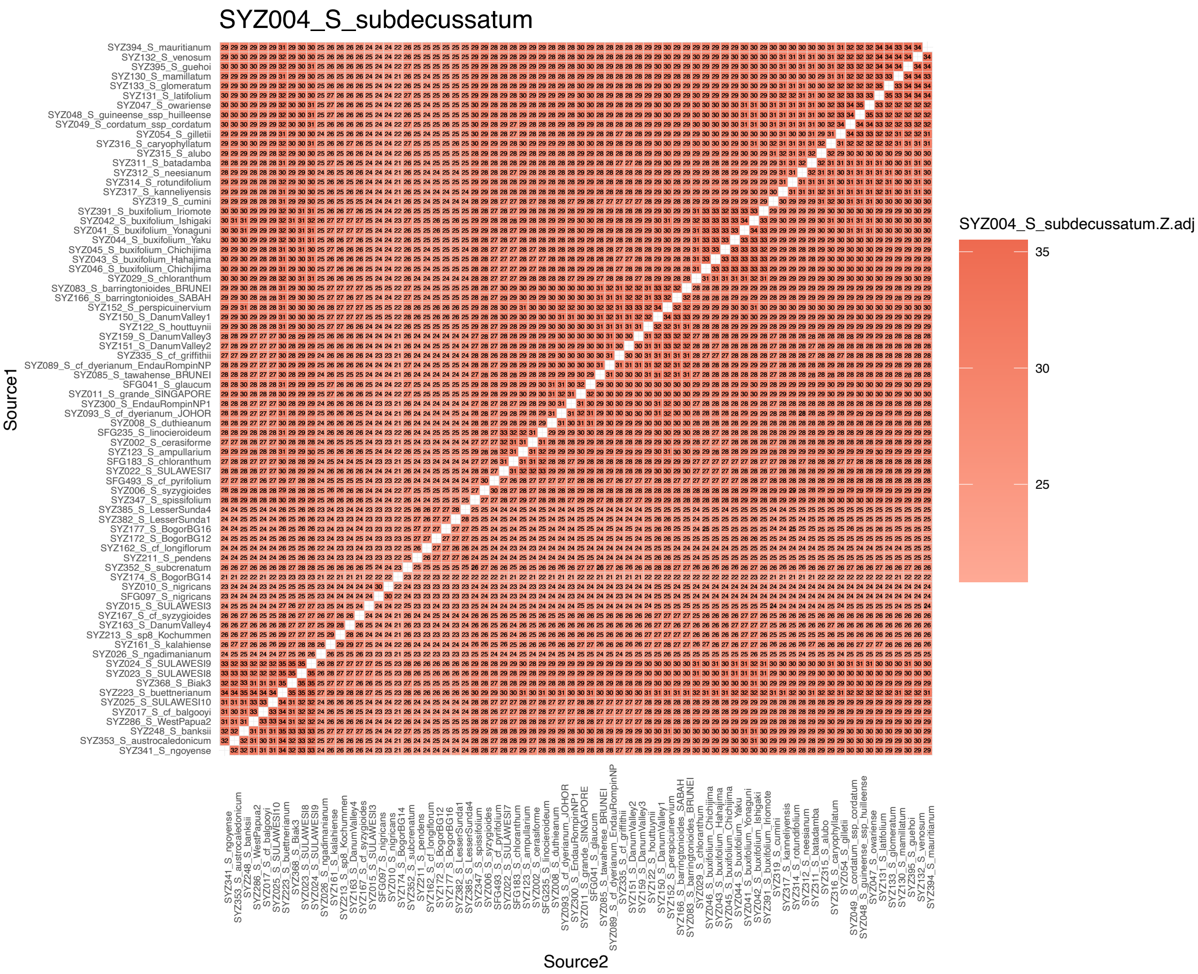

Supplementary Figure 19. F3 heatmap for SYZ004_S_subdecussatum as target, with other members of the *Syzygium grande* group as Source1 or Source2. *Z*-scores adjusted for multiple tests are depicted.

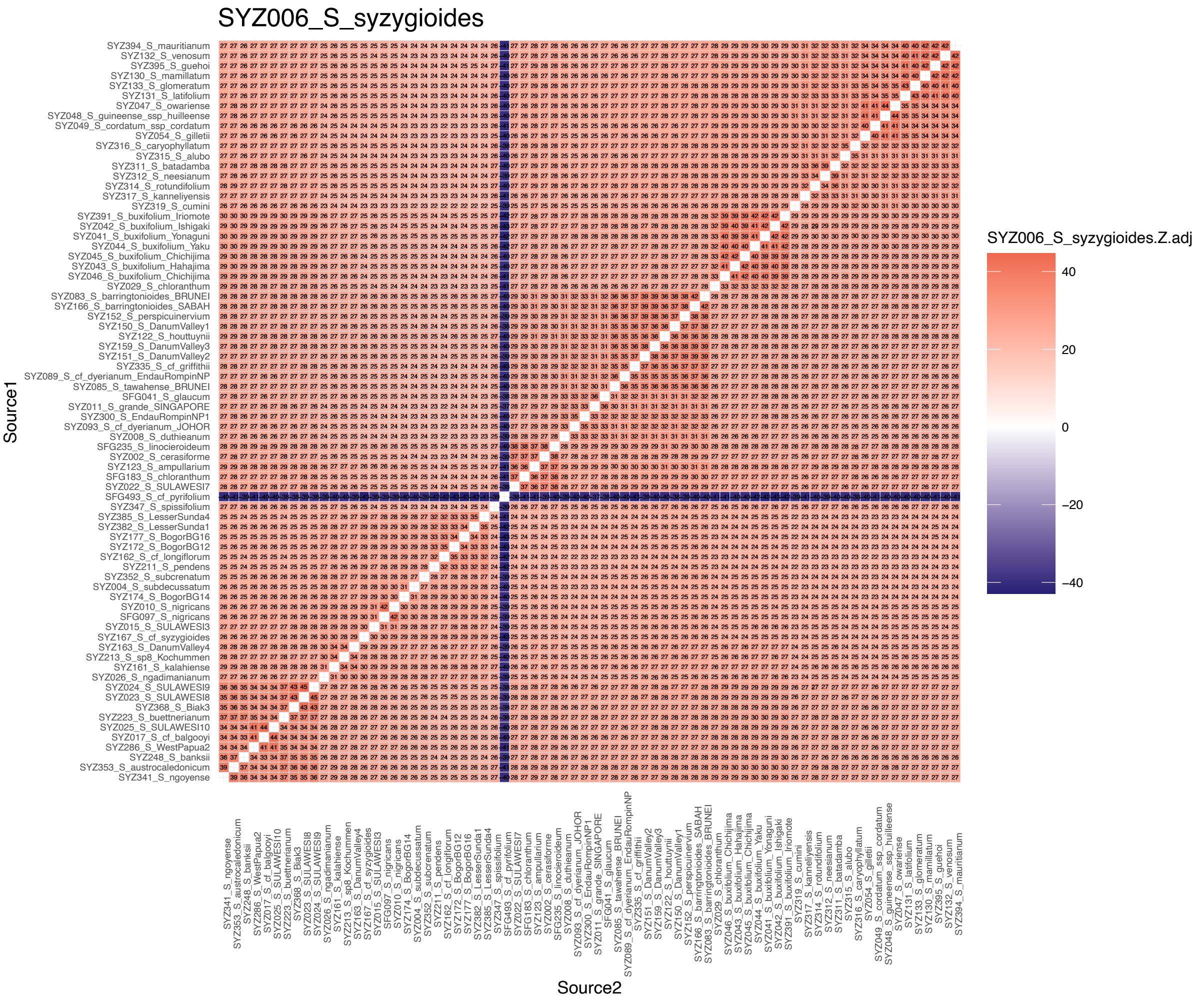

Supplementary Figure 20. F3 heatmap for SYZ006_S_syzygioides as target, with other members of the *Syzygium grande* group as Source1 or Source2. Z-scores adjusted for multiple tests are depicted.

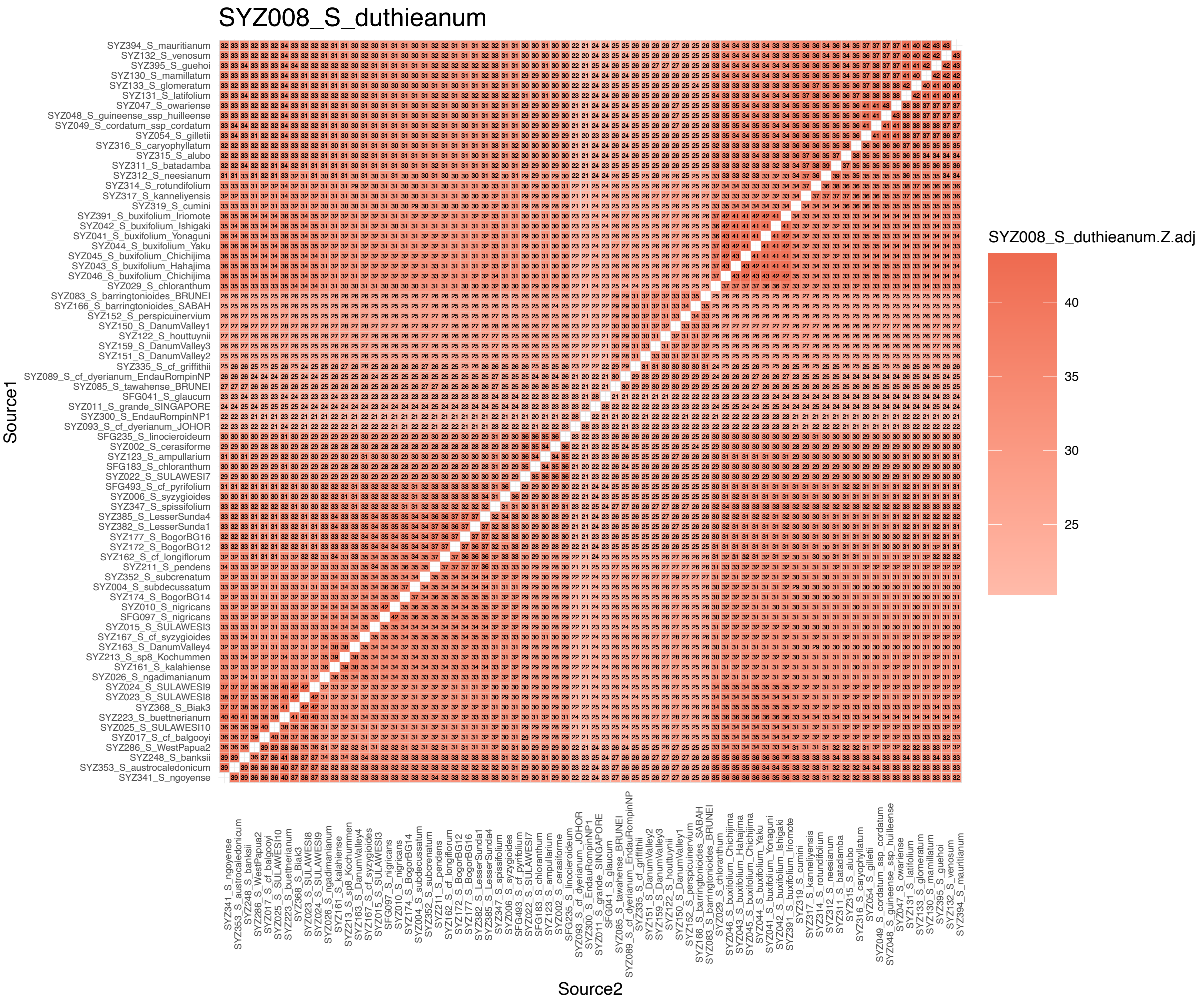

Supplementary Figure 21. F3 heatmap for SYZ008_S_duthieanum as target, with other members of the *Syzygium grande* group as Source1 or Source2. Z-scores adjusted for multiple tests are depicted.

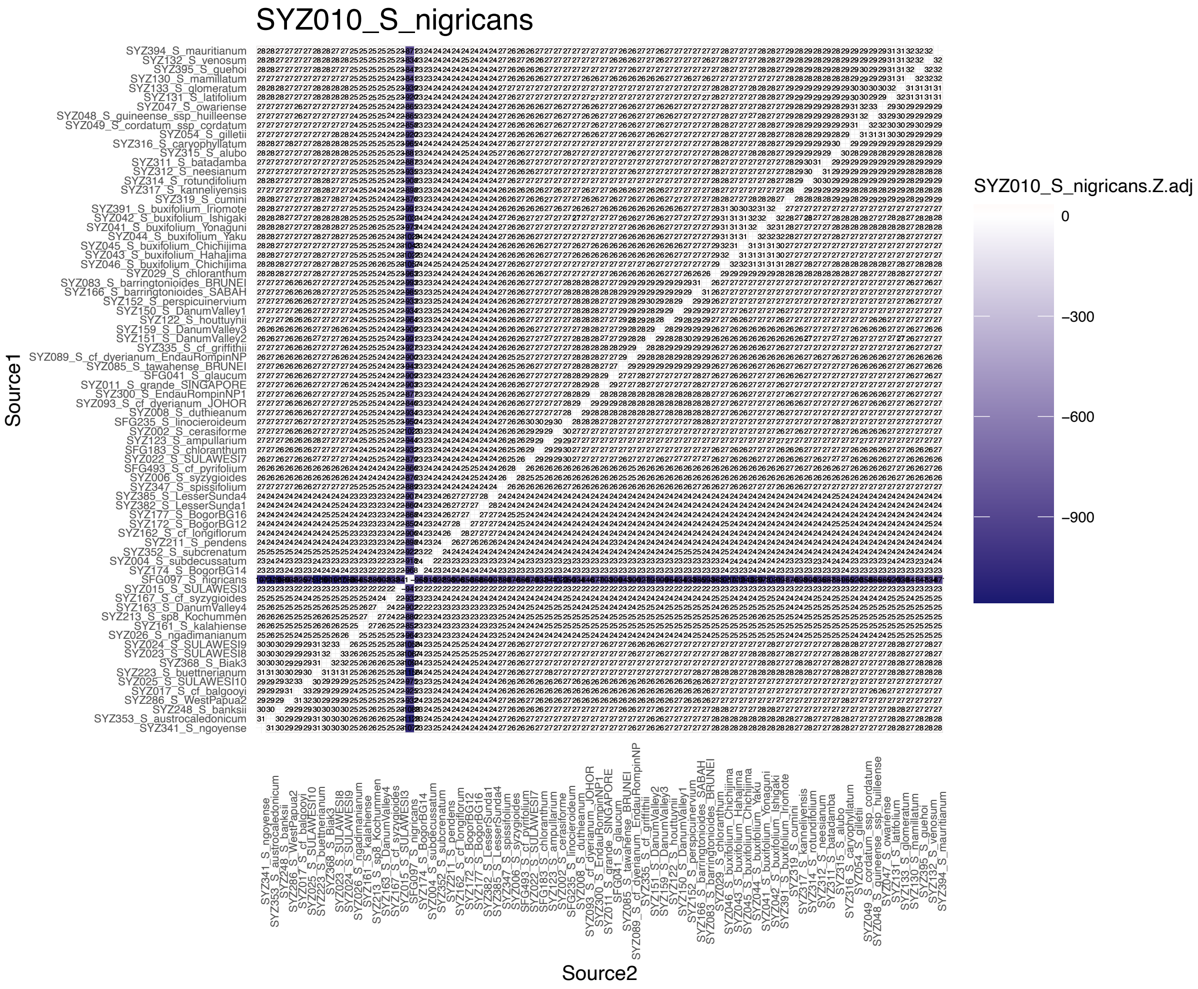

Supplementary Figure 22. F3 heatmap for SYZ010_S_nigricans as target, with other members of the *Syzygium grande* group as Source1 or Source2. Z-scores adjusted for multiple tests are depicted.

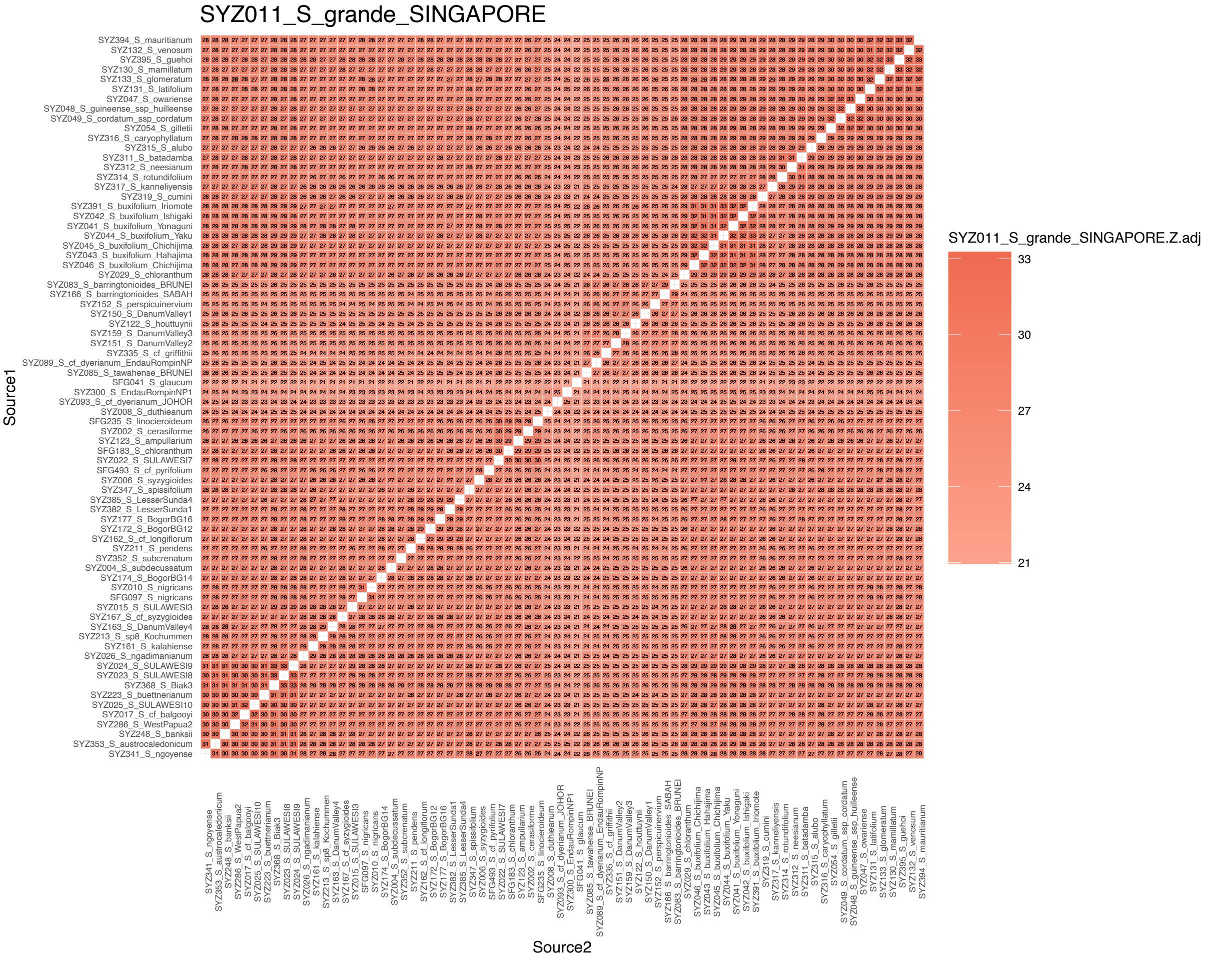

Supplementary Figure 23. F3 heatmap for SYZ011_S_gande_SINGAPORE as target, with other members of the *Syzygium grande* group as Source1 or Source2. Z-scores adjusted for multiple tests are depicted.

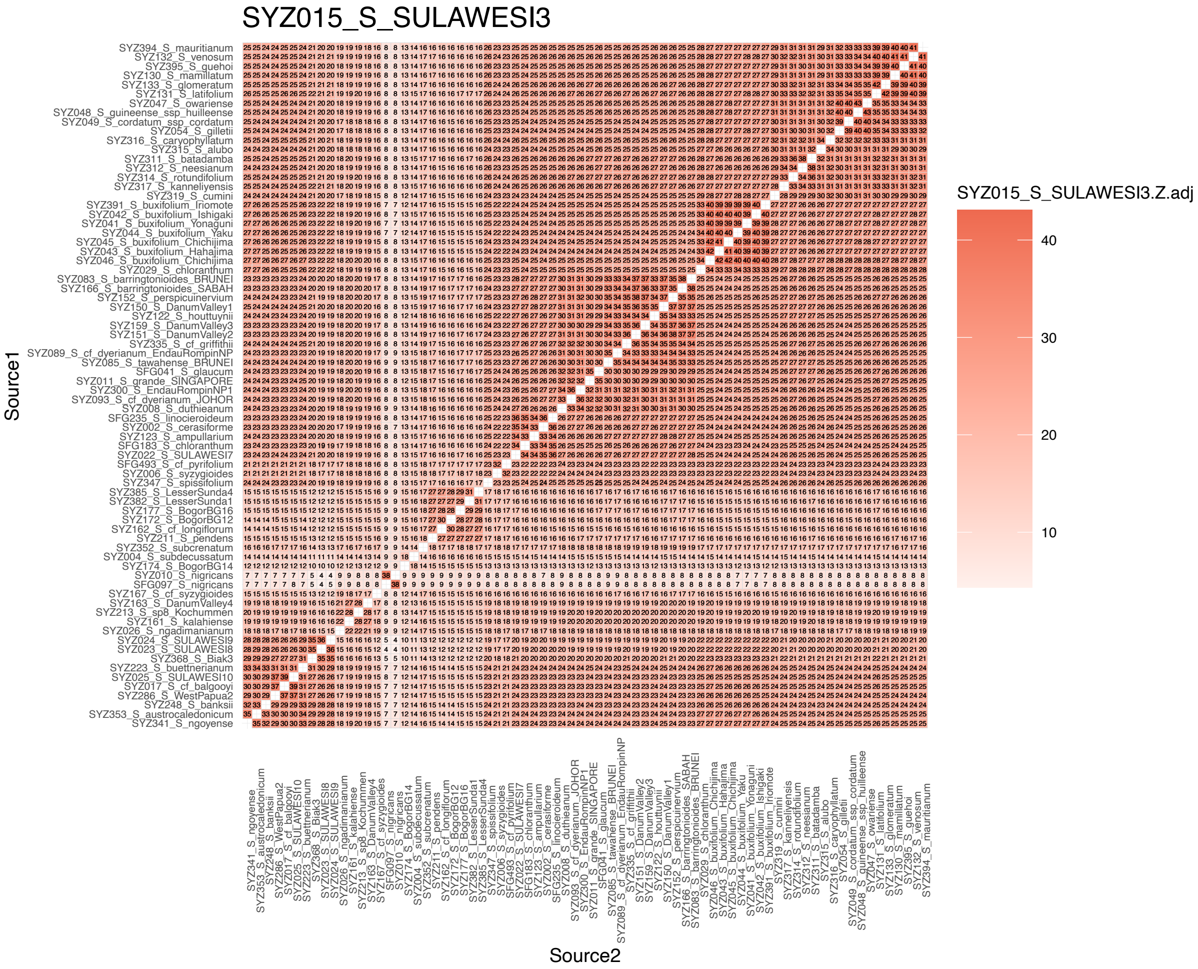

Supplementary Figure 24. F3 heatmap for SYZ015_S_SULAWESI3 as target, with other members of the *Syzygium grande* group as Source1 or Source2. *Z*-scores adjusted for multiple tests are depicted.

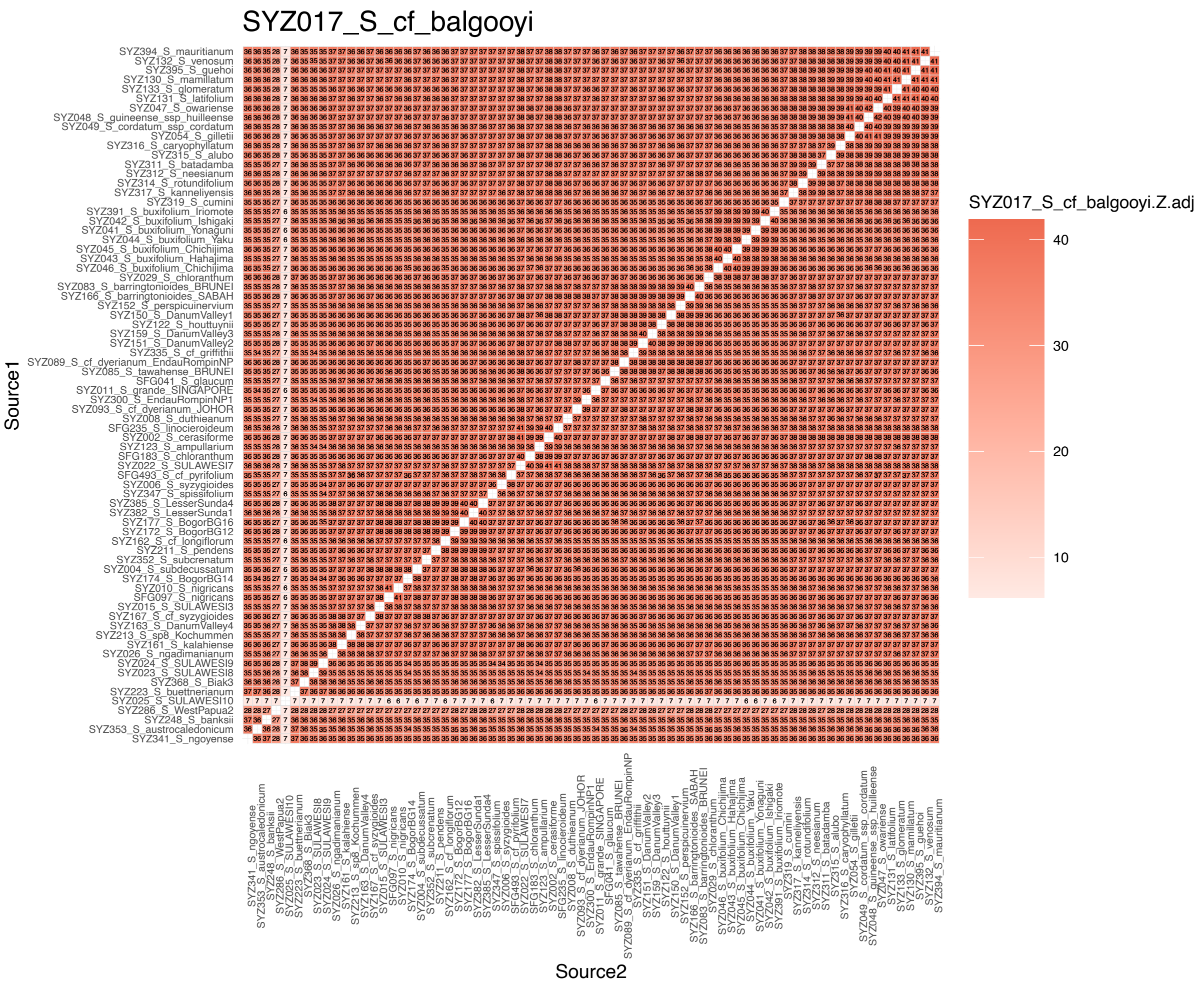

Supplementary Figure 25. F3 heatmap for SYZ017_S_cf_balgooyi as target, with other members of the *Syzygium grande* group as Source1 or Source2. Z-scores adjusted for multiple tests are depicted.

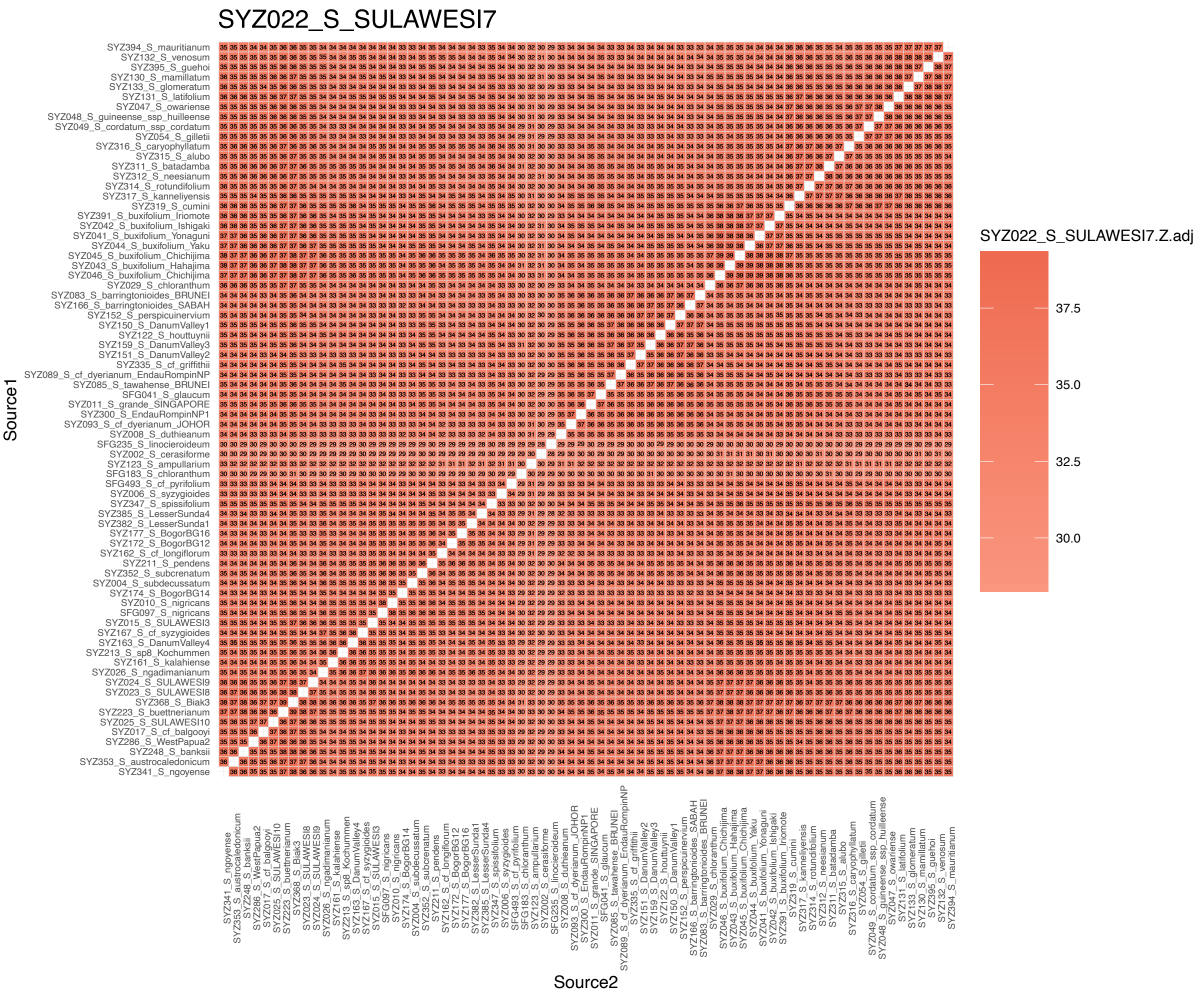

Supplementary Figure 26. F3 heatmap for SYZ022_S_SULAWESI7 as target, with other members of the *Syzygium grande* group as Source1 or Source2. Z-scores adjusted for multiple tests are depicted.

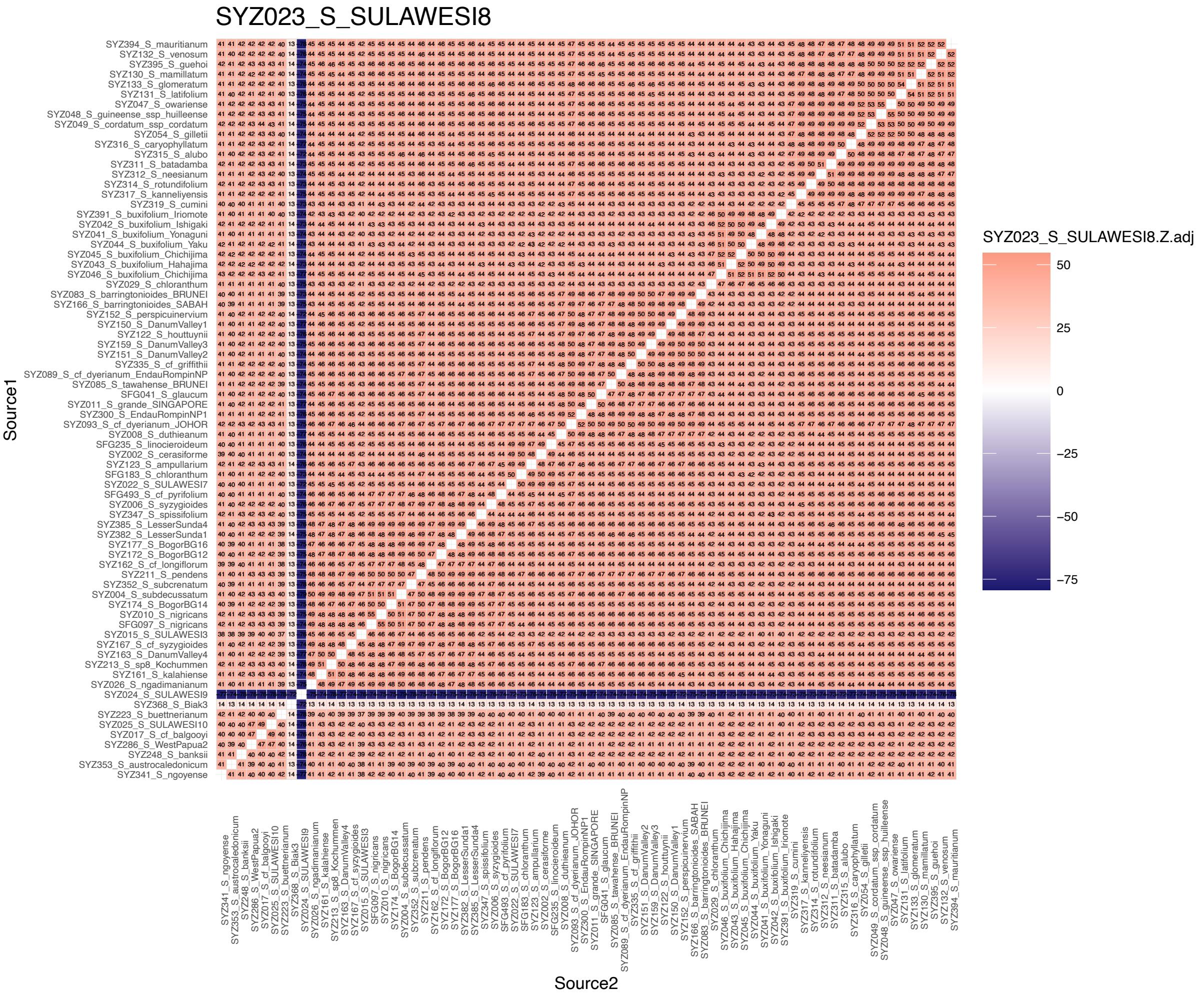

Supplementary Figure 27. F3 heatmap for SYZ023_S_SULAWESI8 as target, with other members of the *Syzygium grande* group as Source1 or Source2. Z-scores adjusted for multiple tests are depicted.

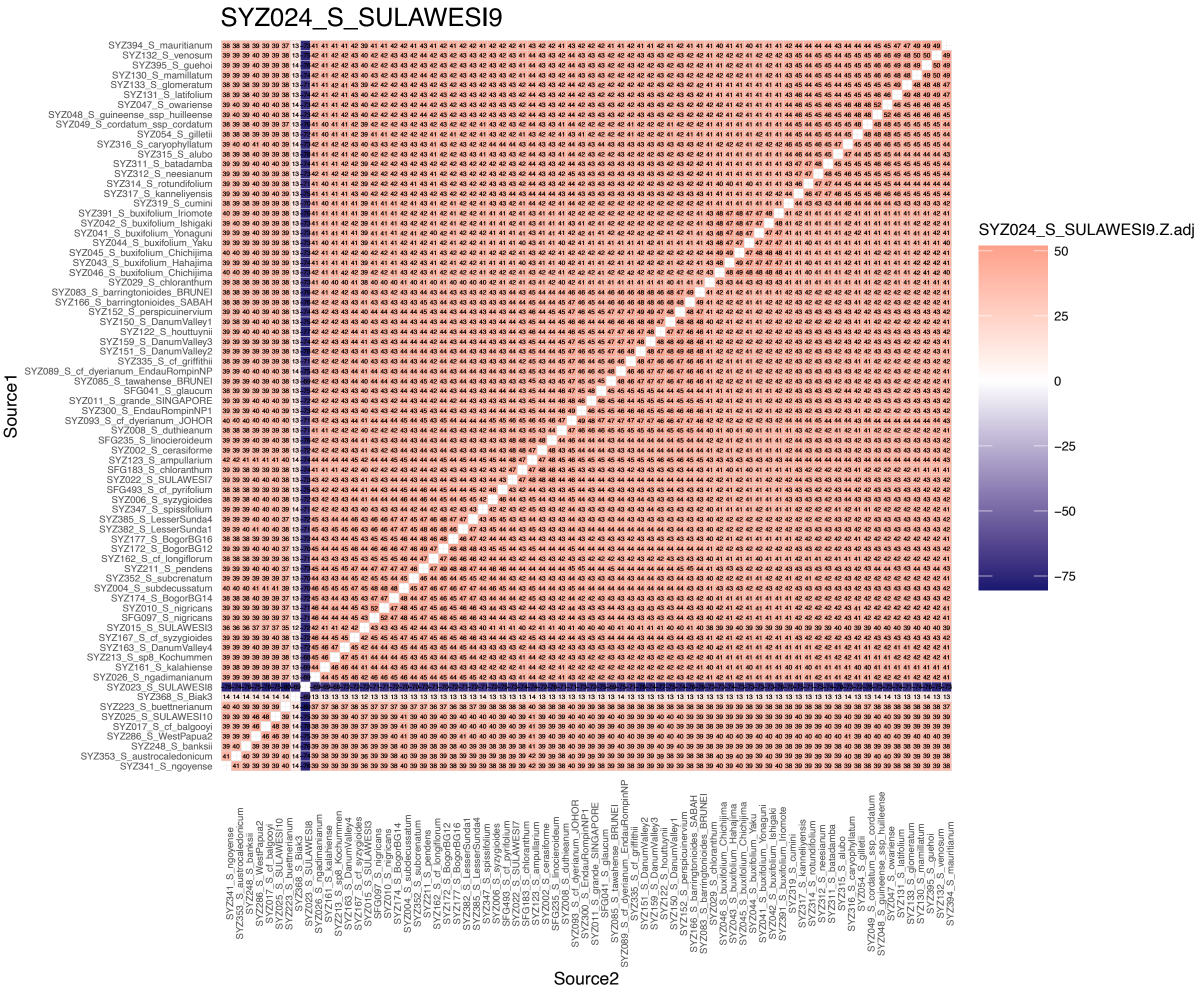

Supplementary Figure 28. F3 heatmap for SYZ024_S_SULAWESI9 as target, with other members of the *Syzygium grande* group as Source1 or Source2. *Z*-scores adjusted for multiple tests are depicted.

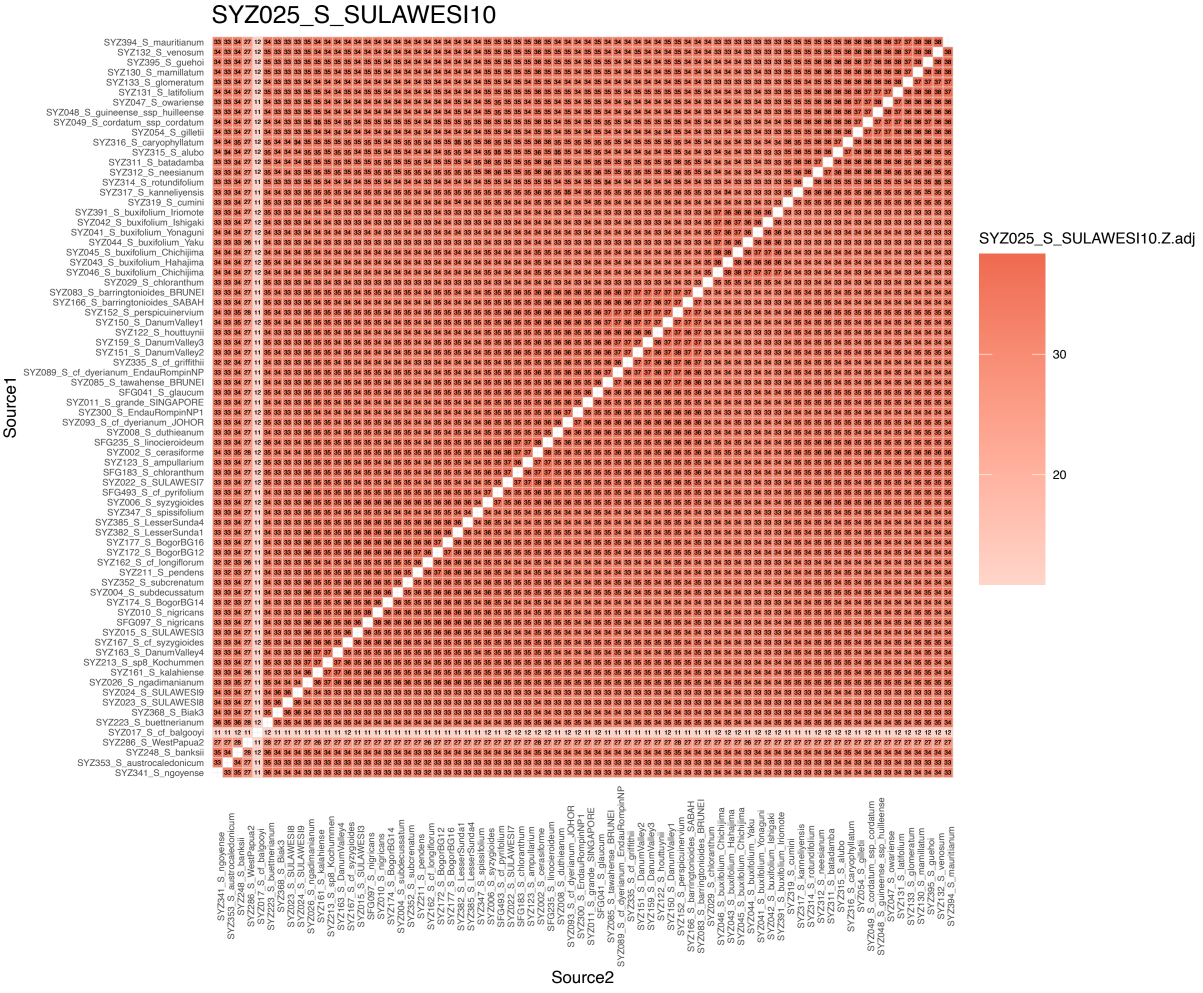

Supplementary Figure 29. F3 heatmap for SYZ025_S_SULAWESI10 as target, with other members of the *Syzygium grande* group as Source1 or Source2. *Z*-scores adjusted for multiple tests are depicted.

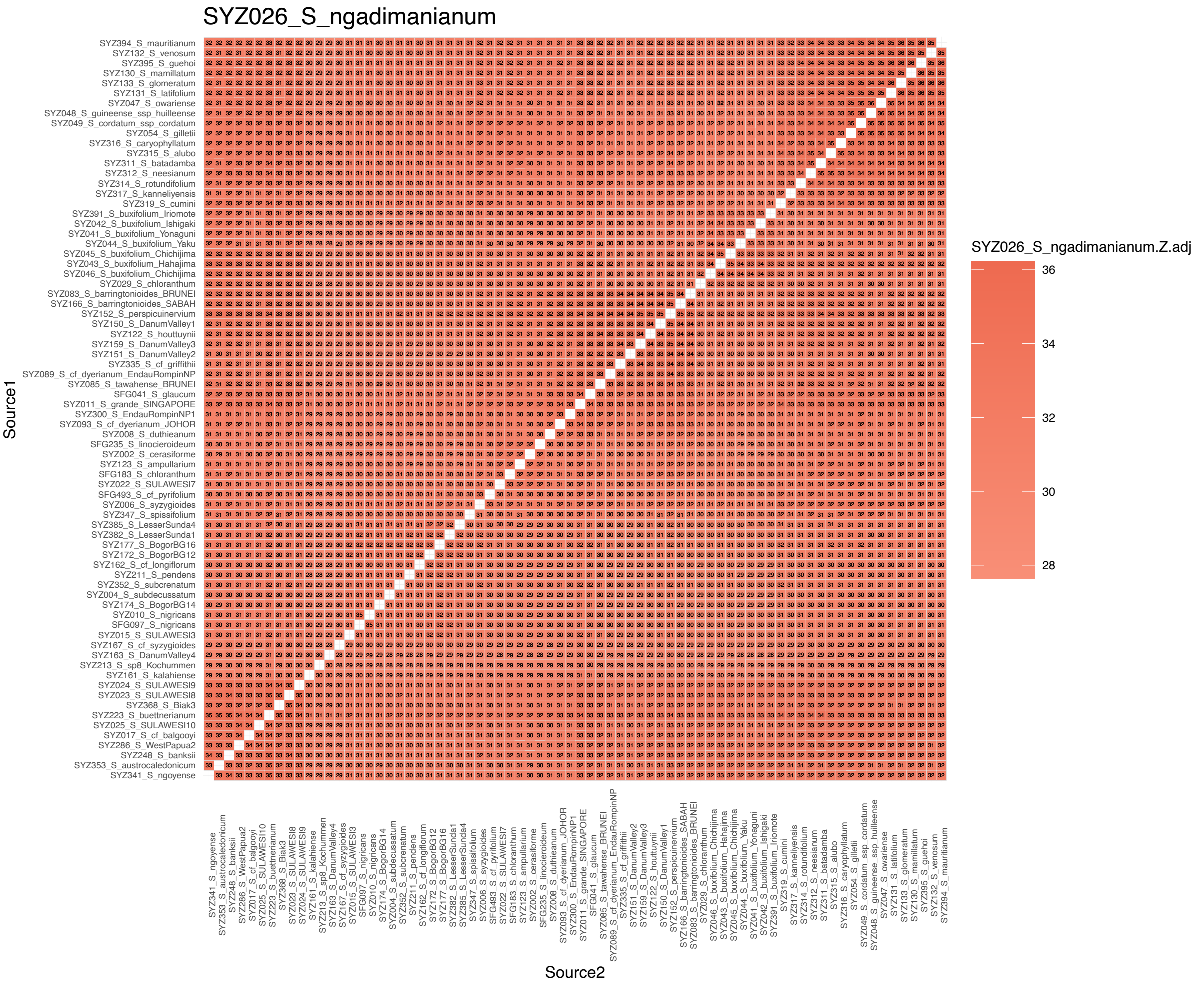

Supplementary Figure 30. F3 heatmap for SYZ026_S_ngadimanianum as target, with other members of the *Syzygium grande* group as Source1 or Source2. *Z*-scores adjusted for multiple tests are depicted.

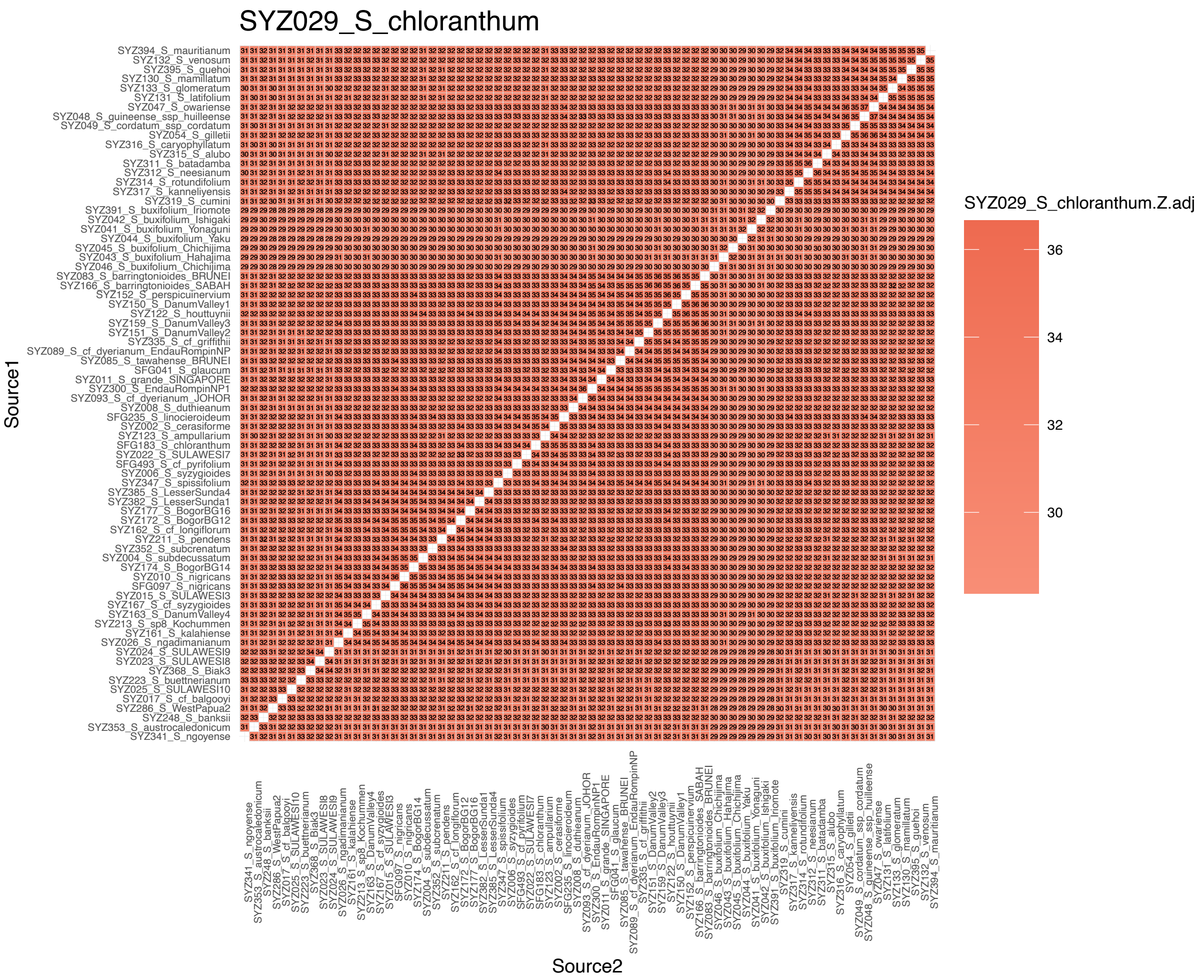

Supplementary Figure 31. F3 heatmap for SYZ029_S_chloranthum as target, with other members of the *Syzygium grande* group as Source1 or Source2. *Z*-scores adjusted for multiple tests are depicted.

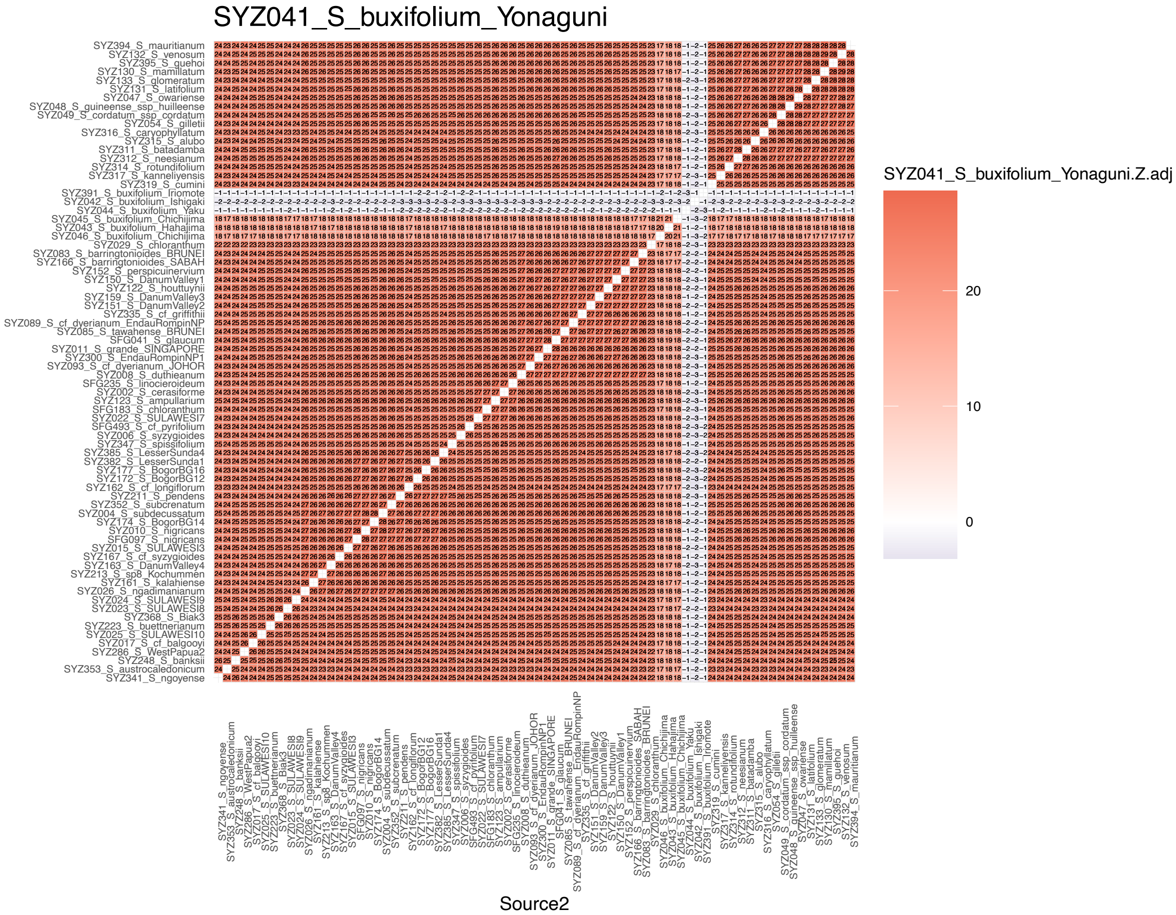

Supplementary Figure 32. F3 heatmap for SYZ041_S_buxifolium_Yonaguni as target, with other members of the *Syzygium grande* group as Source1 or Source2. Z-scores adjusted for multiple tests are depicted.

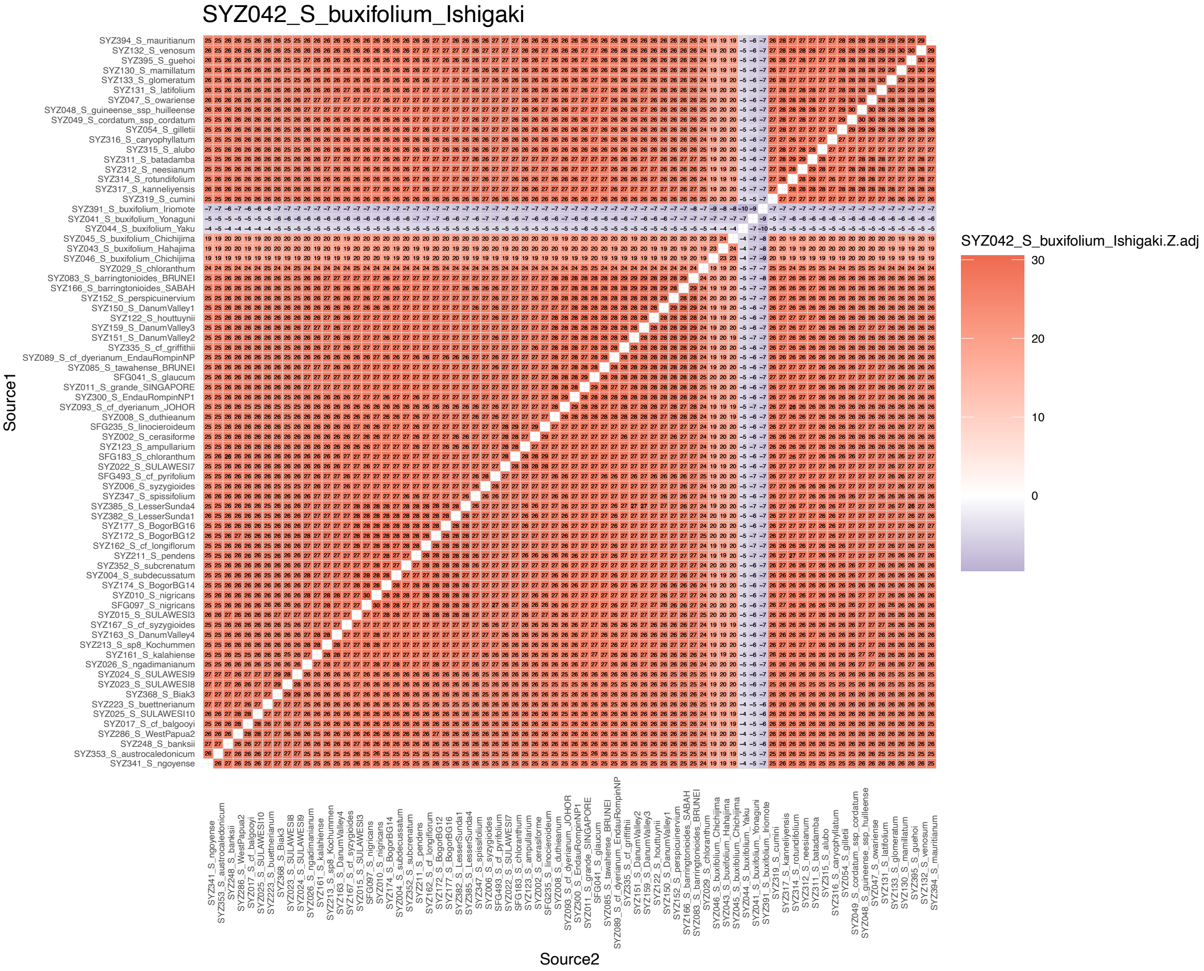

Supplementary Figure 33. F3 heatmap for SYZ042_S_buxifolium_Ishigaki as target, with other members of the *Syzygium grande* group as Source1 or Source2. Z-scores adjusted for multiple tests are depicted.

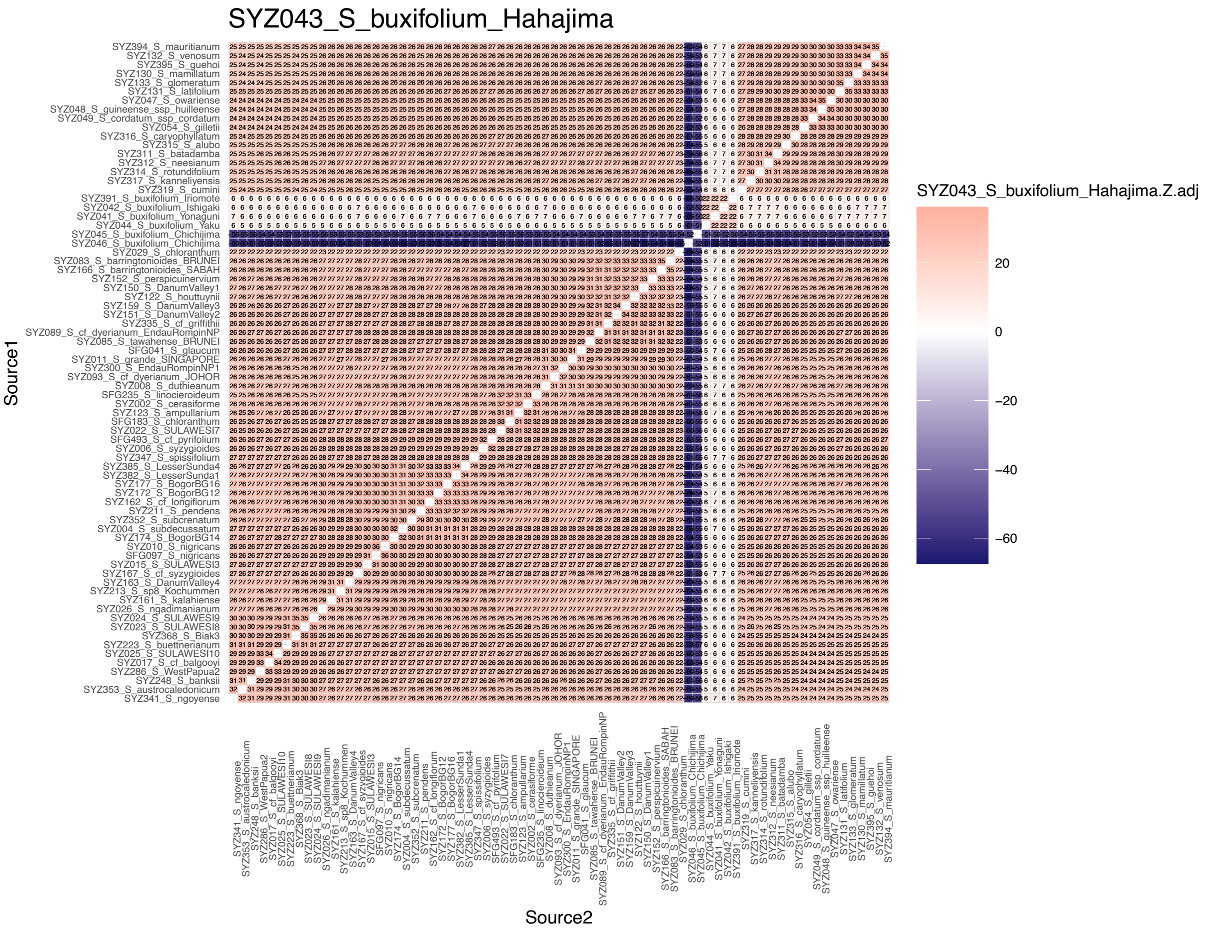

Supplementary Figure 34. F3 heatmap for SYZ043_S_buxifolium_Hahajima as target, with other members of the *Syzygium grande* group as Source1 or Source2. Z-scores adjusted for multiple tests are depicted.

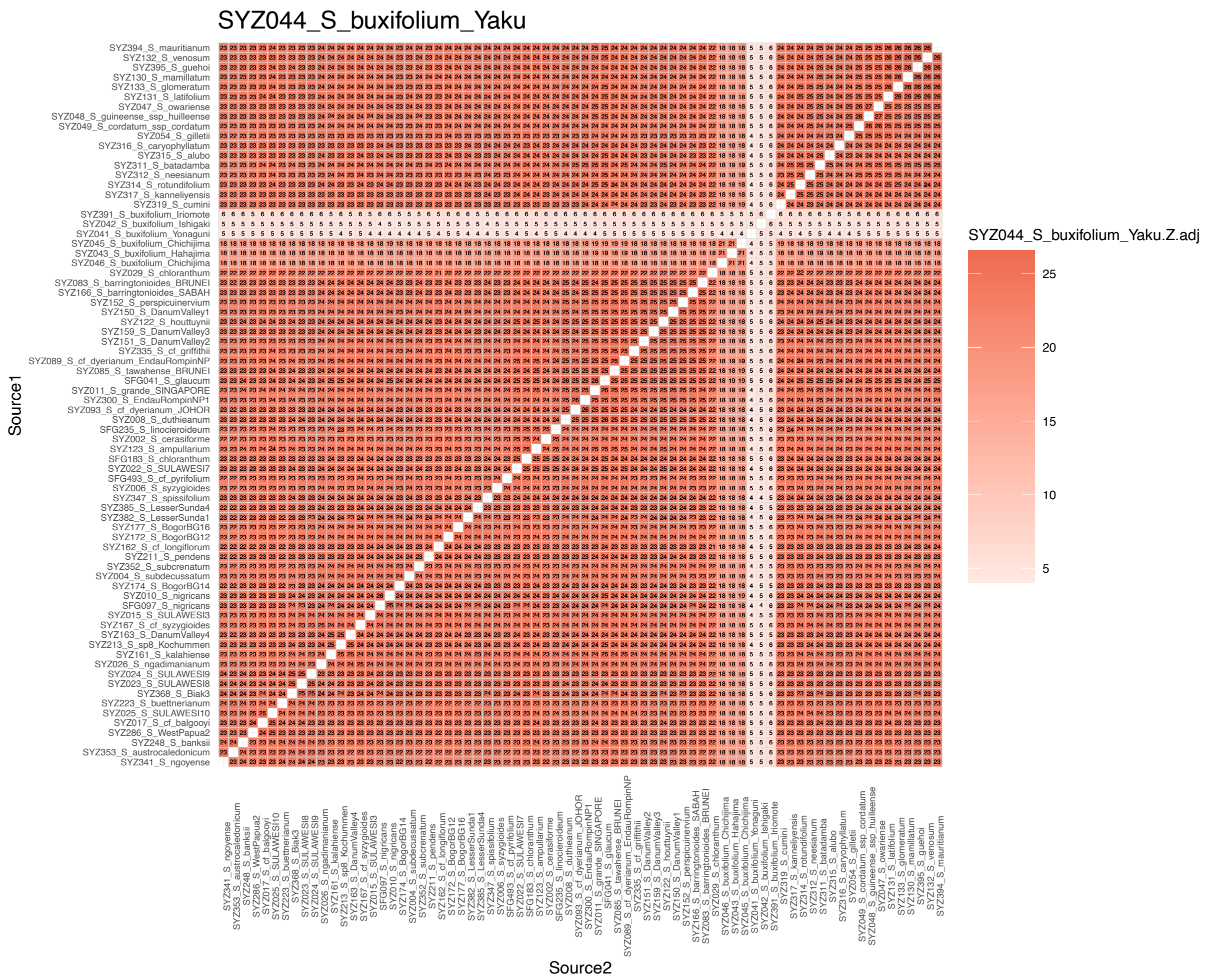

Supplementary Figure 35. F3 heatmap for SYZ044_S_buxifolium_Yaku as target, with other members of the *Syzygium grande* group as Source1 or Source2. *Z*-scores adjusted for multiple tests are depicted.

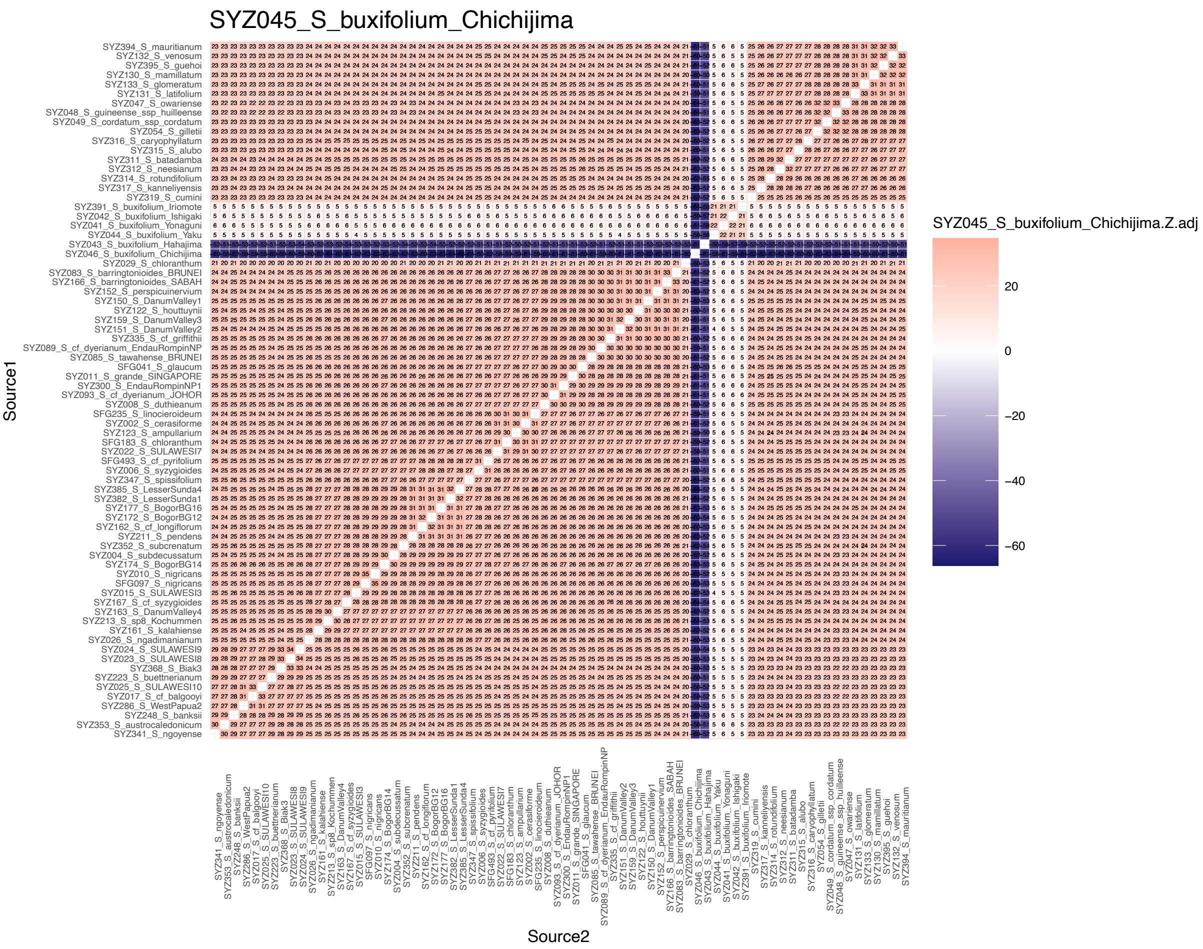

Supplementary Figure 36. F3 heatmap for SYZ045_S_buxifolium_Chichijima as target, with other members of the *Syzygium grande* group as Source1 or Source2. *Z*-scores adjusted for multiple tests are depicted.

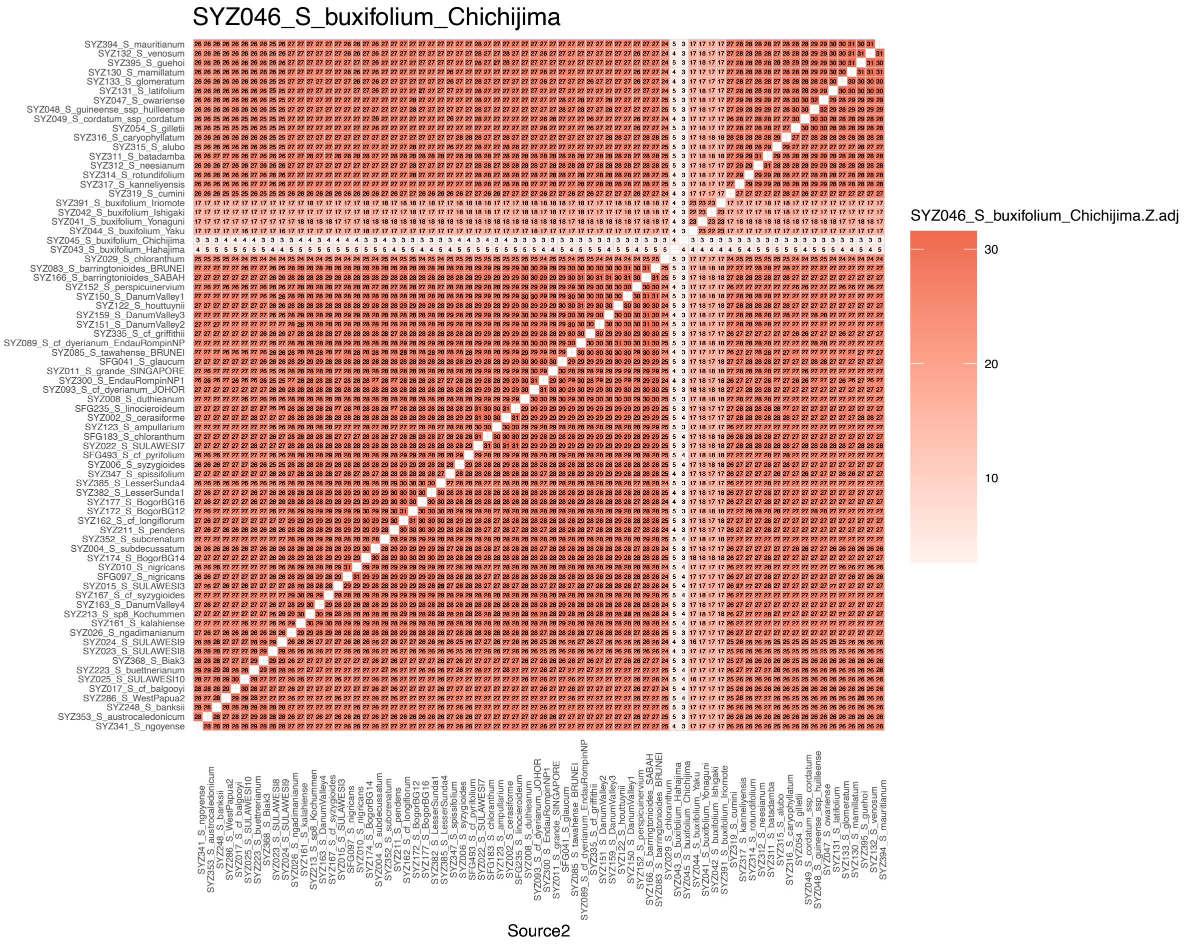

Supplementary Figure 37. F3 heatmap for SYZ046_S_buxifolium_Chichijima as target, with other members of the *Syzygium grande* group as Source1 or Source2. Z-scores adjusted for multiple tests are depicted.

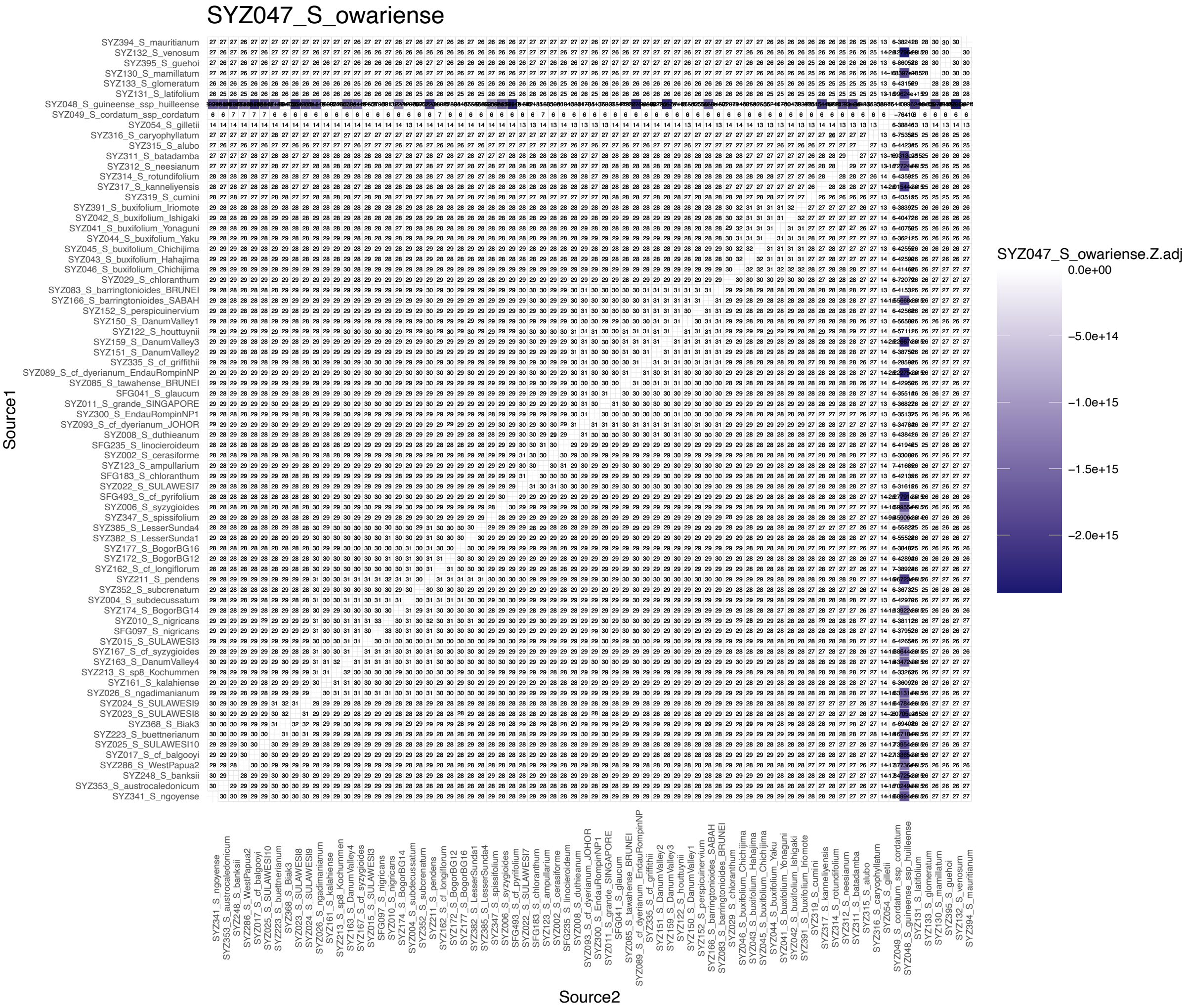

Supplementary Figure 38. F3 heatmap for SYZ047_S_owariense as target, with other members of the *Syzygium grande* group as Source1 or Source2. Z-scores adjusted for multiple tests are depicted.

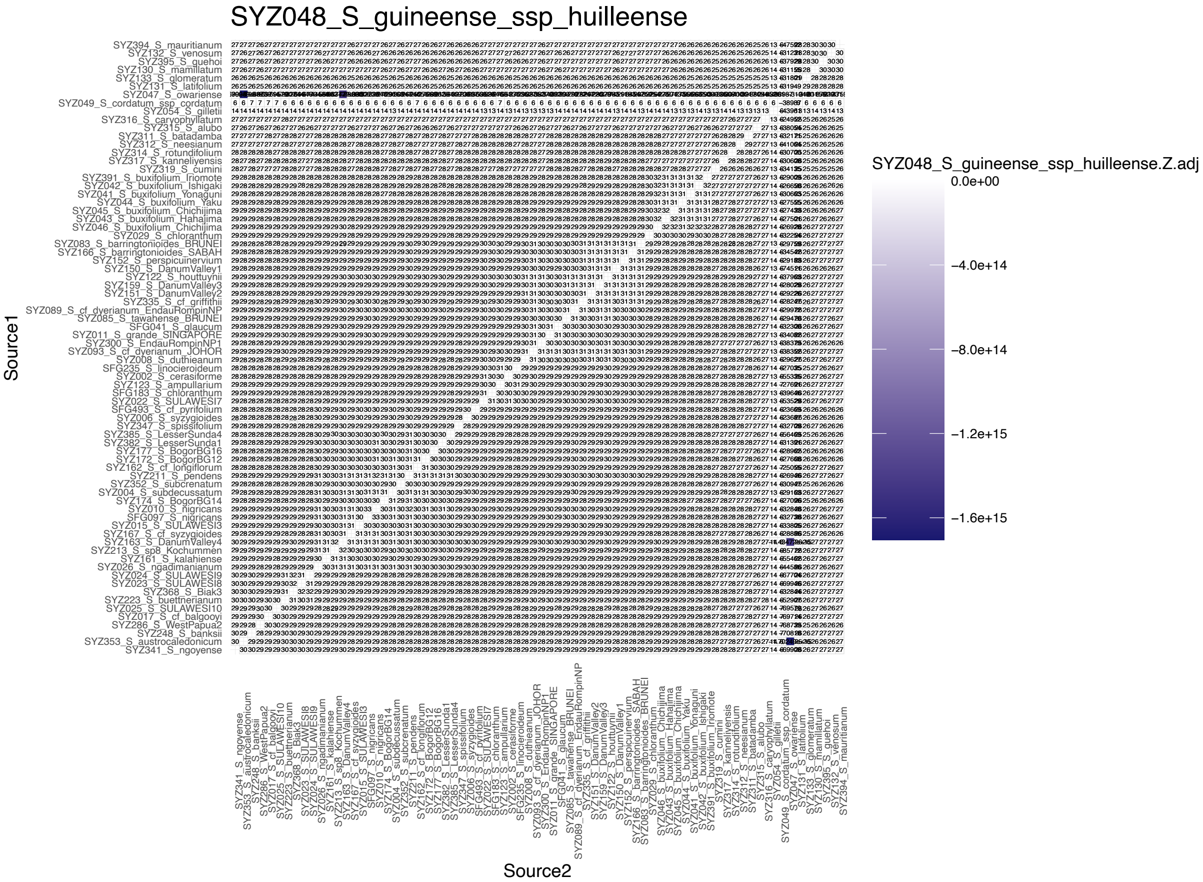

Supplementary Figure 39. F3 heatmap for SYZ048_S_guineense_ssp_huilleense as target, with other members of the *Syzygium grande* group as Source1 or Source2. Z-scores adjusted for multiple tests are depicted.

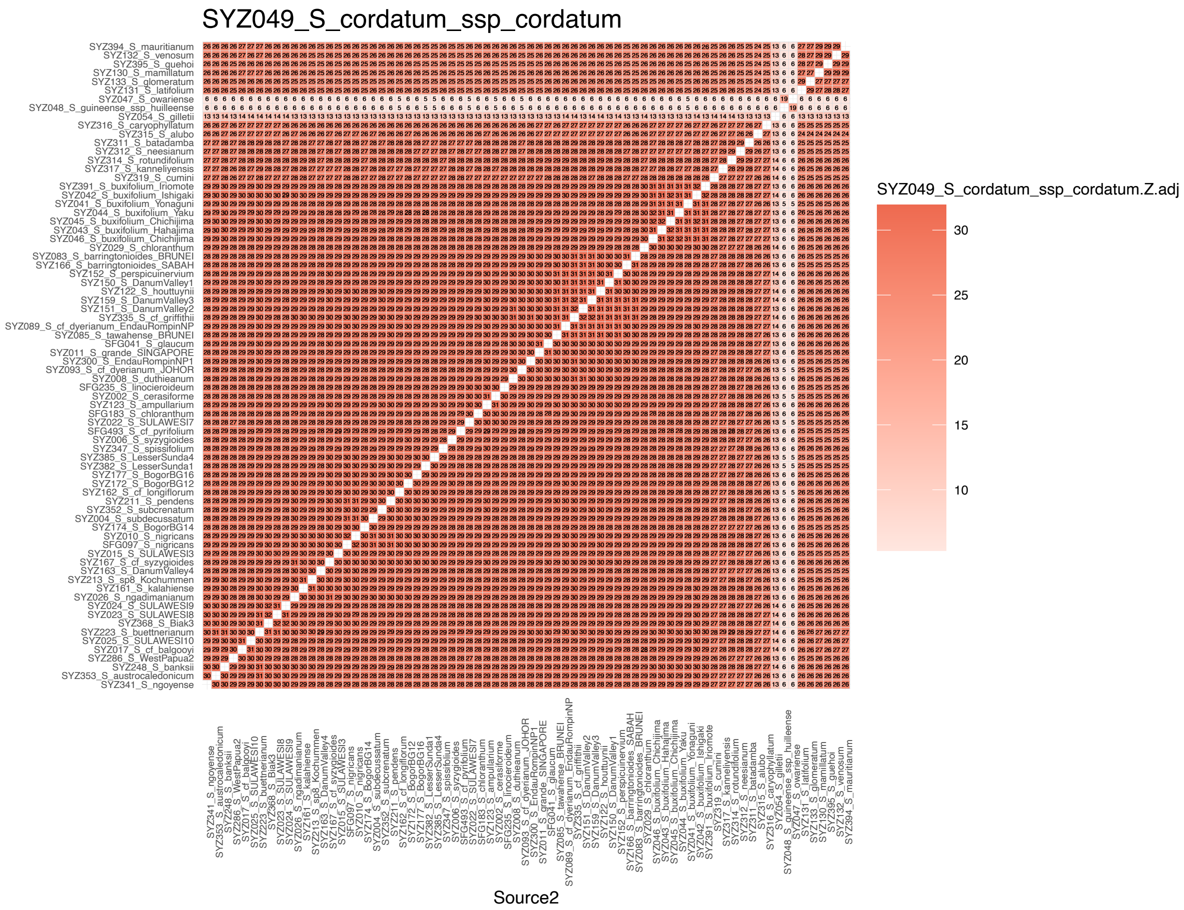

Supplementary Figure 40. F3 heatmap for SYZ049_S_cordatum_ssp_cordatum as target, with other members of the *Syzygium grande* group as Source1 or Source2. *Z*-scores adjusted for multiple tests are depicted.

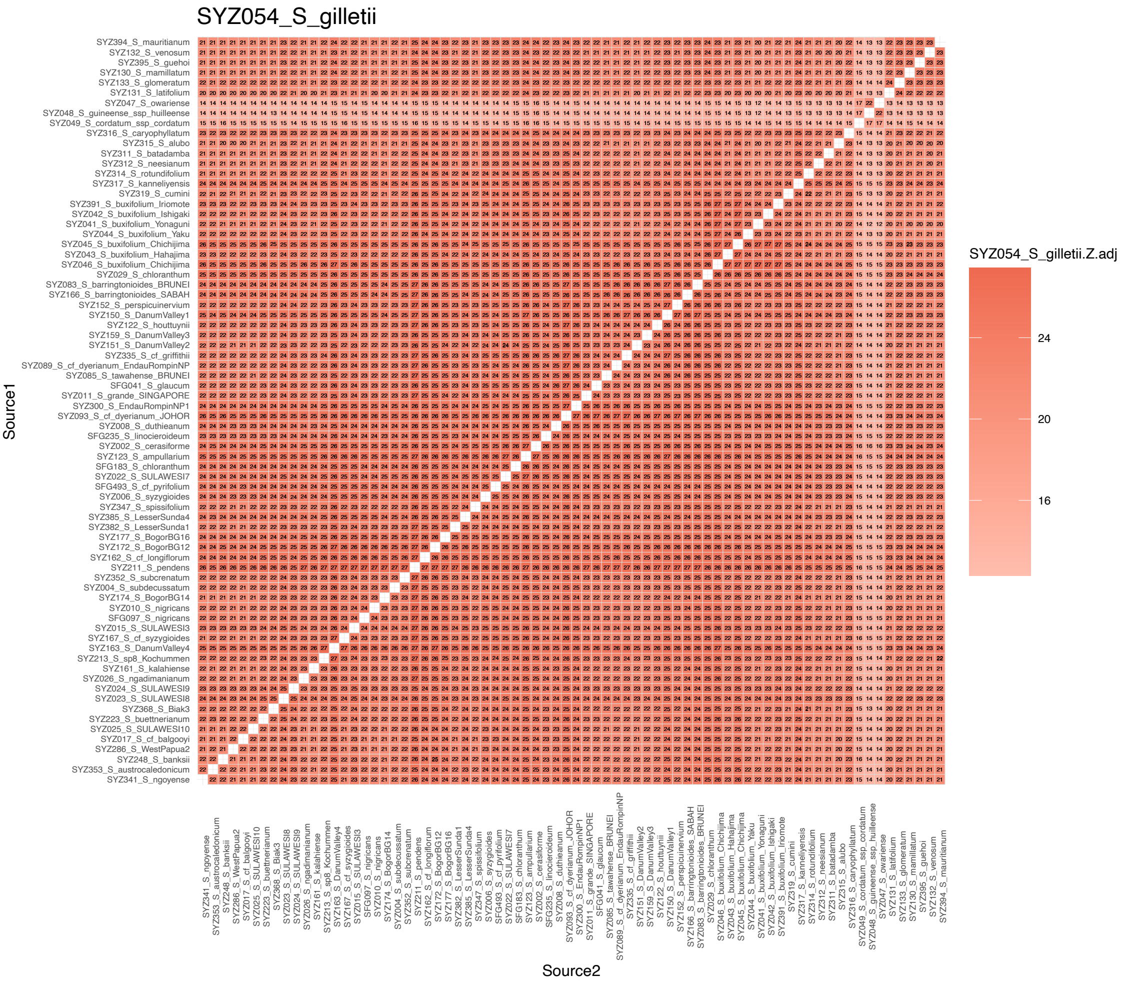

Supplementary Figure 41. F3 heatmap for SYZ054_S_gilletii as target, with other members of the *Syzygium grande* group as Source1 or Source2. Z-scores adjusted for multiple tests are depicted.

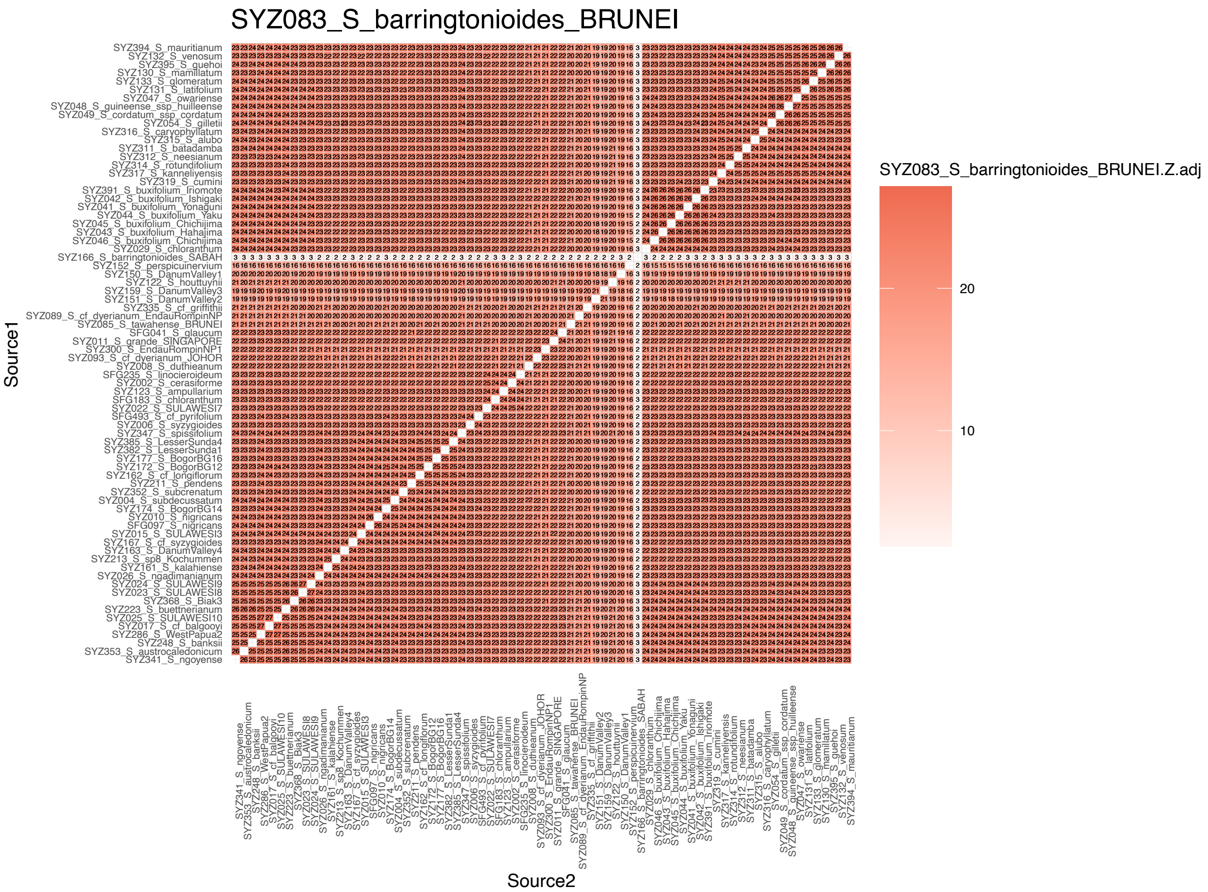

Supplementary Figure 42. F3 heatmap for SYZ083_S_barringtonioides as target, with other members of the *Syzygium grande* group as Source1 or Source2. *Z*-scores adjusted for multiple tests are depicted.

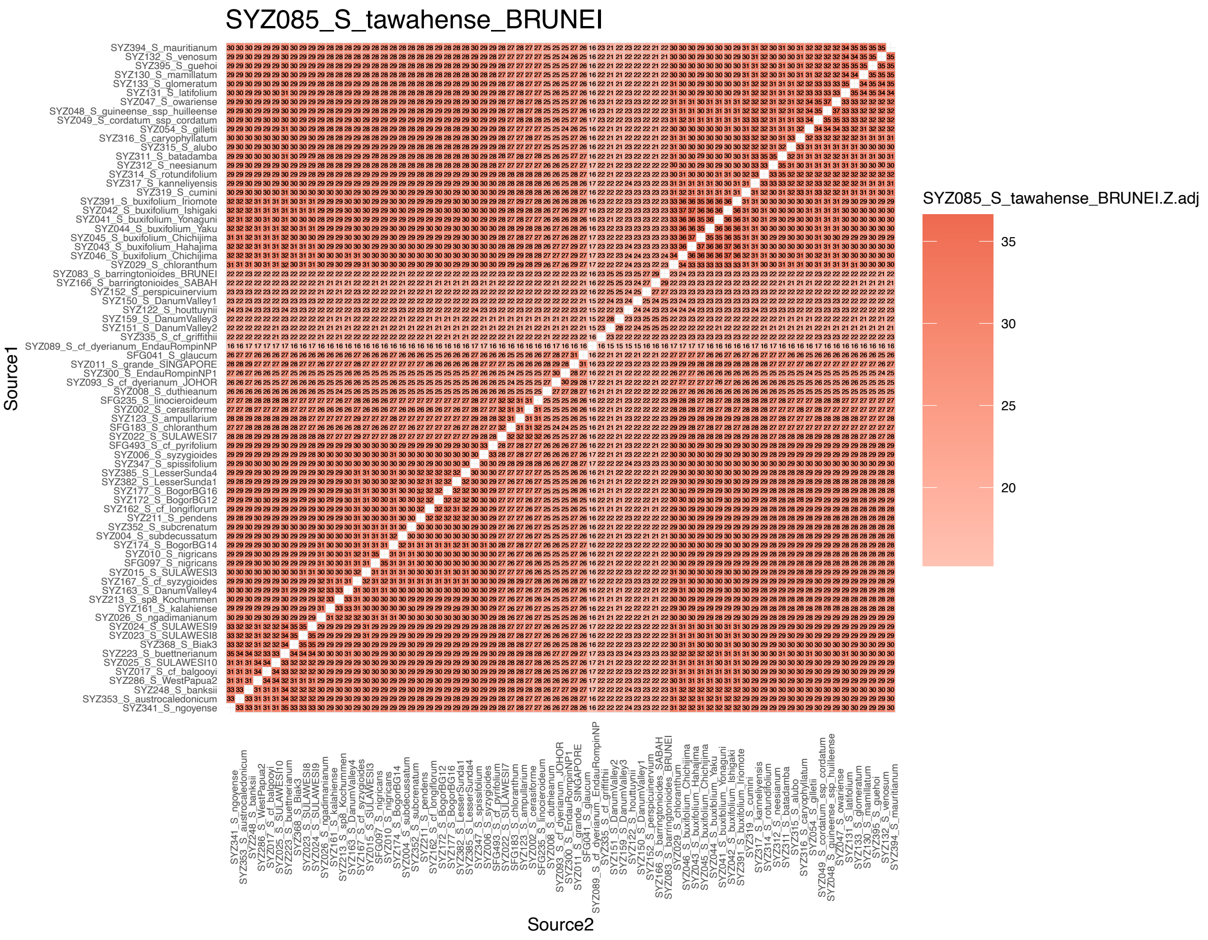

Supplementary Figure 43. F3 heatmap for SYZ085_S_tawahense_BRUNEI as target, with other members of the *Syzygium grande* group as Source1 or Source2. *Z*-scores adjusted for multiple tests are depicted.

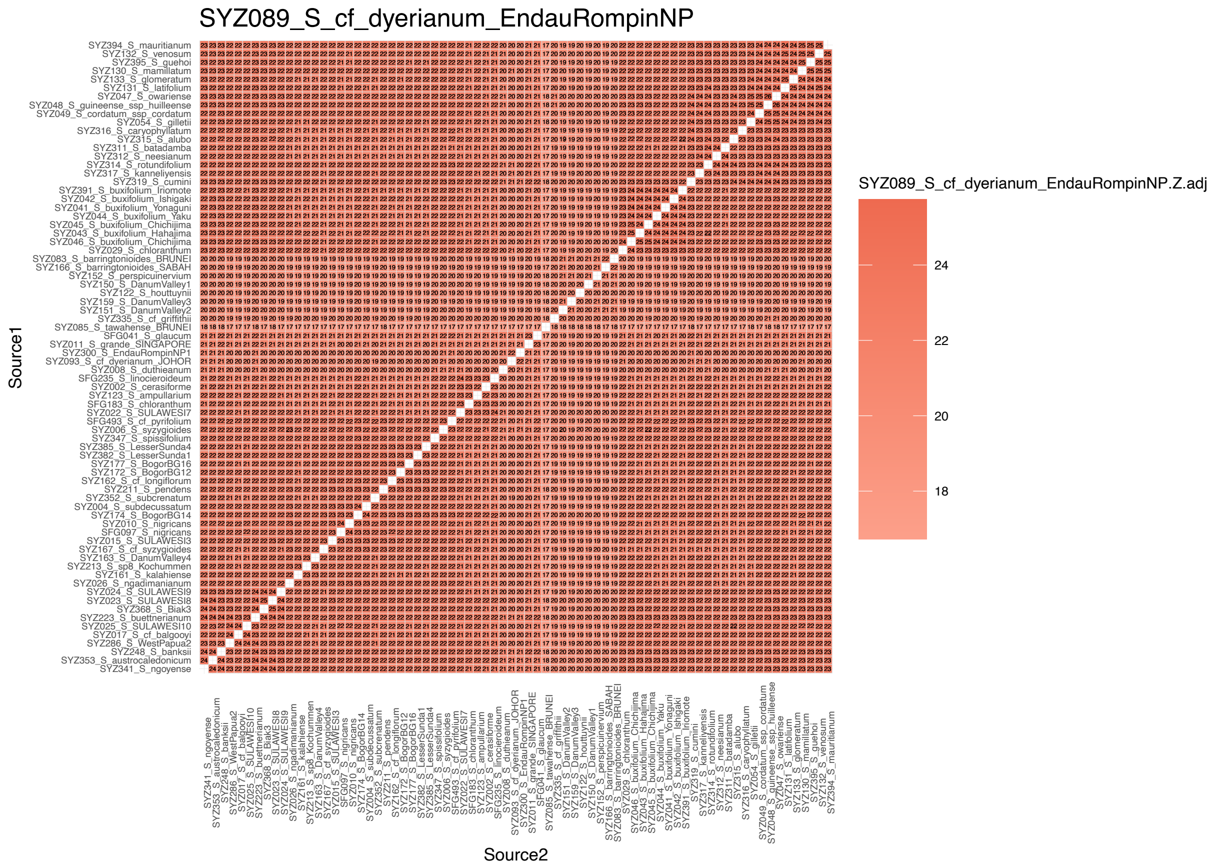

Supplementary Figure 44. F3 heatmap for SYZ089_S_cf_dyerianum_EndauRompinNP as target, with other members of the *Syzygium grande* group as Source1 or Source2. Z-scores adjusted for multiple tests are depicted.

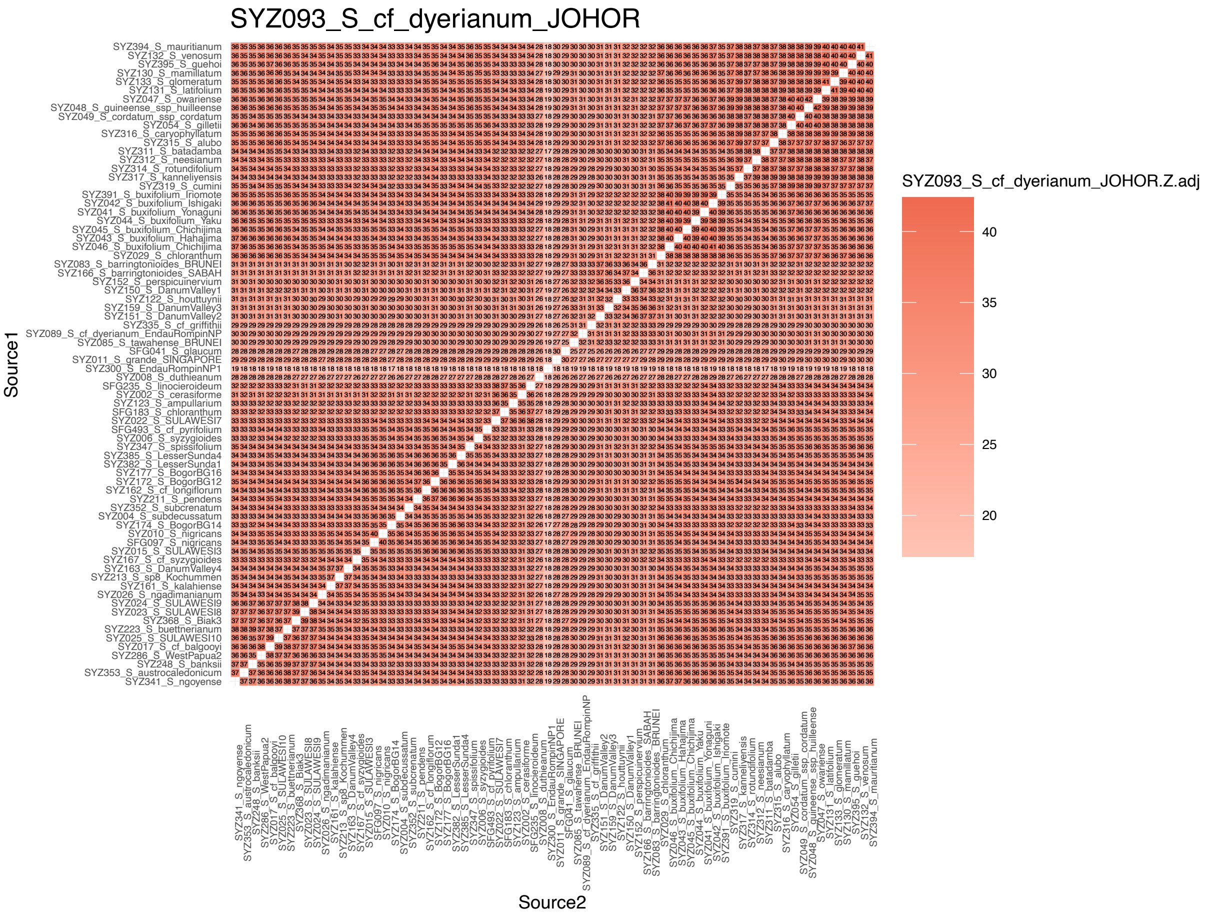

Supplementary Figure 45. F3 heatmap for SYZ093_S_cf_dyerianum_JOHOR as target, with other members of the *Syzygium grande* group as Source1 or Source2. *Z*-scores adjusted for multiple tests are depicted.

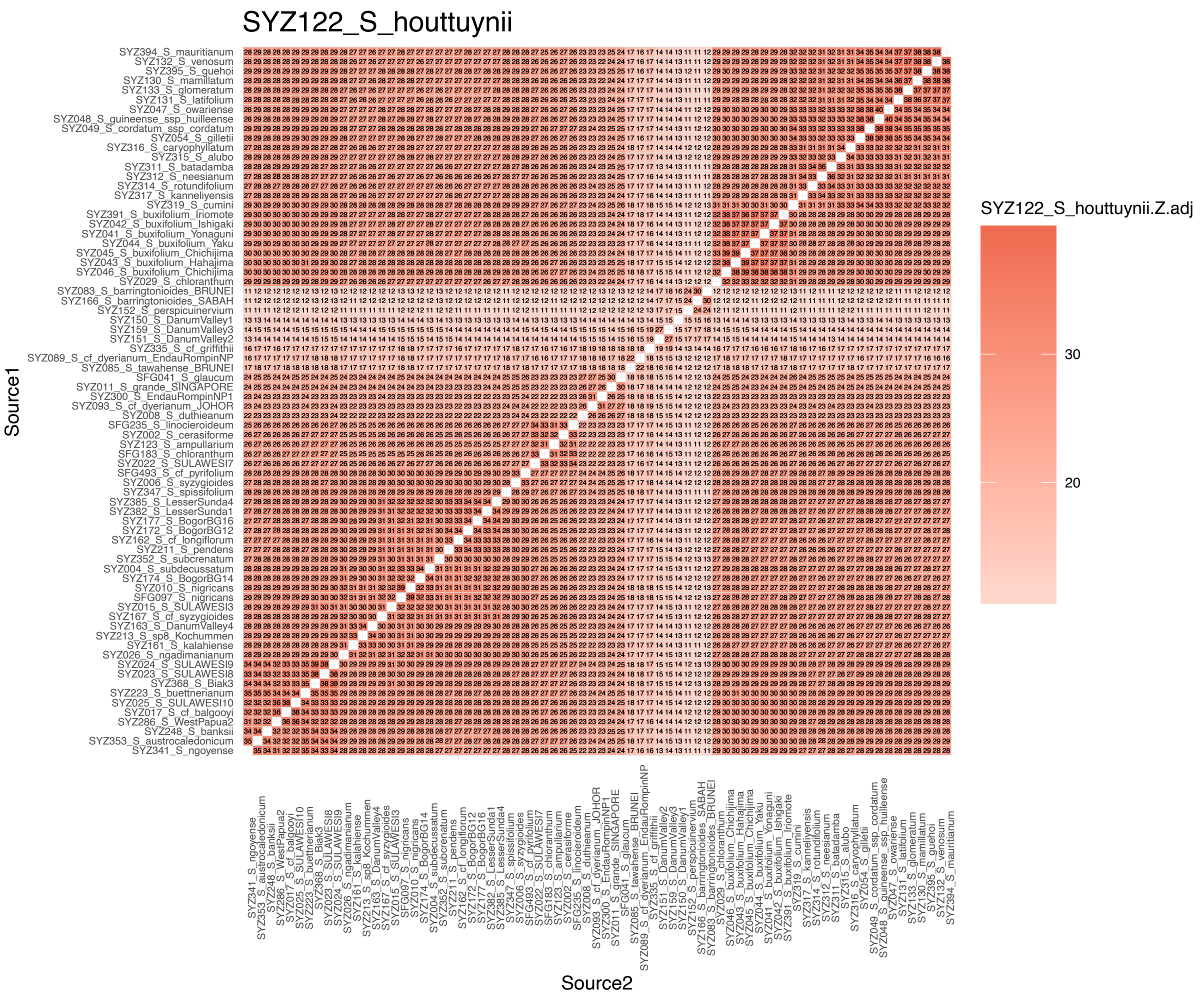

Supplementary Figure 46. F3 heatmap for SYZ122_S_houttuynii as target, with other members of the *Syzygium grande* group as Source1 or Source2. Z-scores adjusted for multiple tests are depicted.

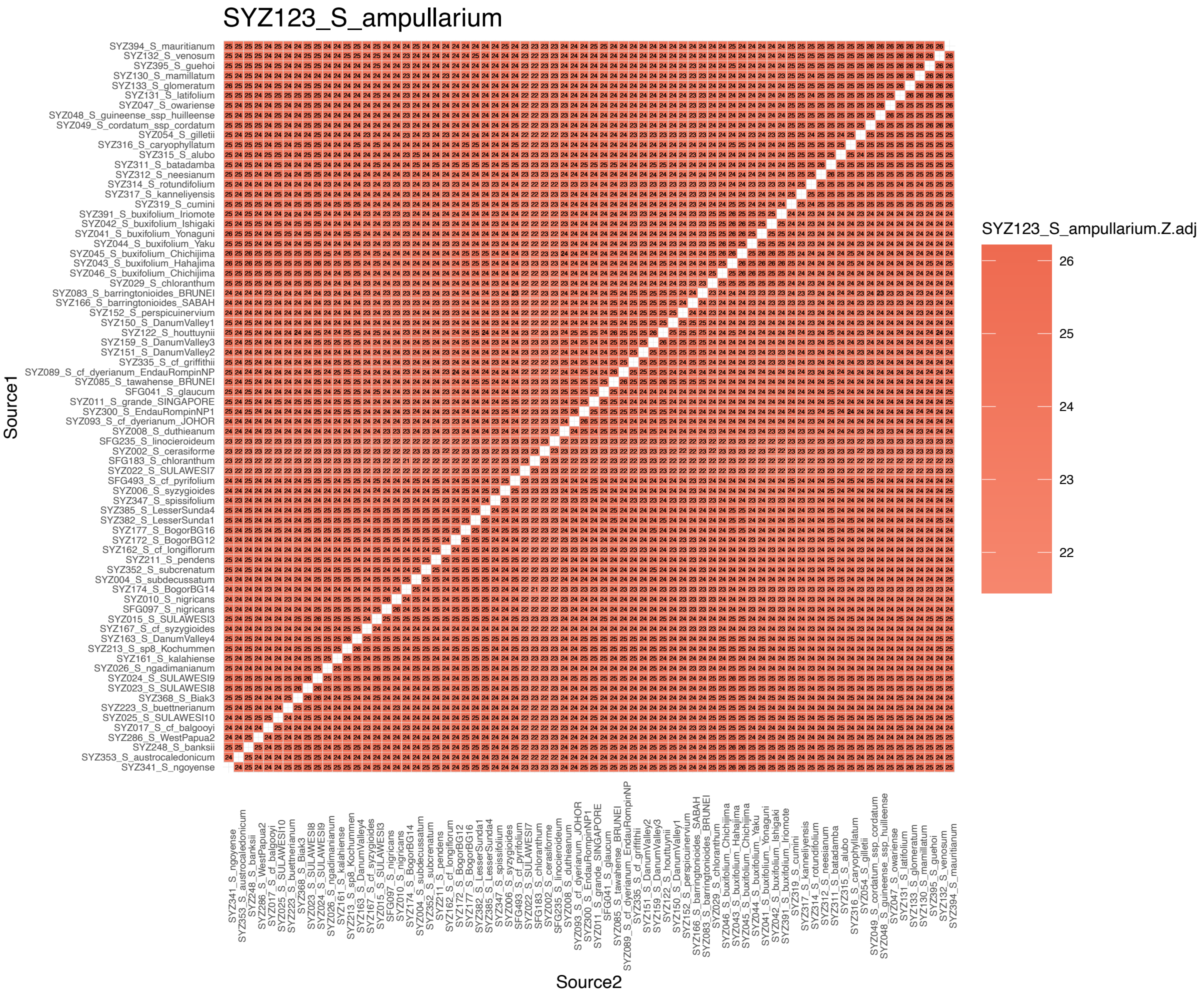

Supplementary Figure 47. F3 heatmap for SYZ123_S_ampullarium as target, with other members of the *Syzygium grande* group as Source1 or Source2. *Z*-scores adjusted for multiple tests are depicted.

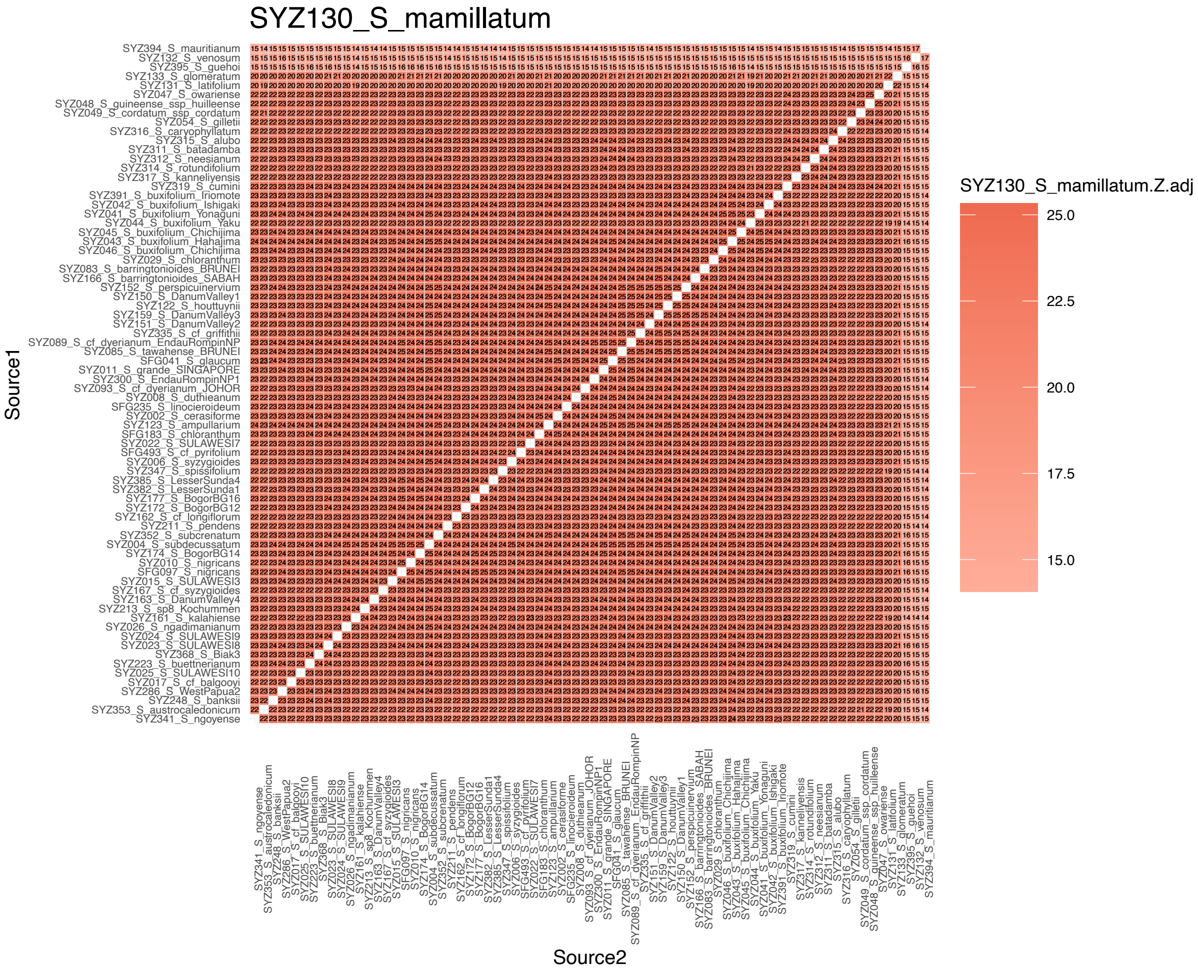

Supplementary Figure 48. F3 heatmap for SYZ130_S_mamillatum as target, with other members of the *Syzygium grande* group as Source1 or Source2. *Z*-scores adjusted for multiple tests are depicted.

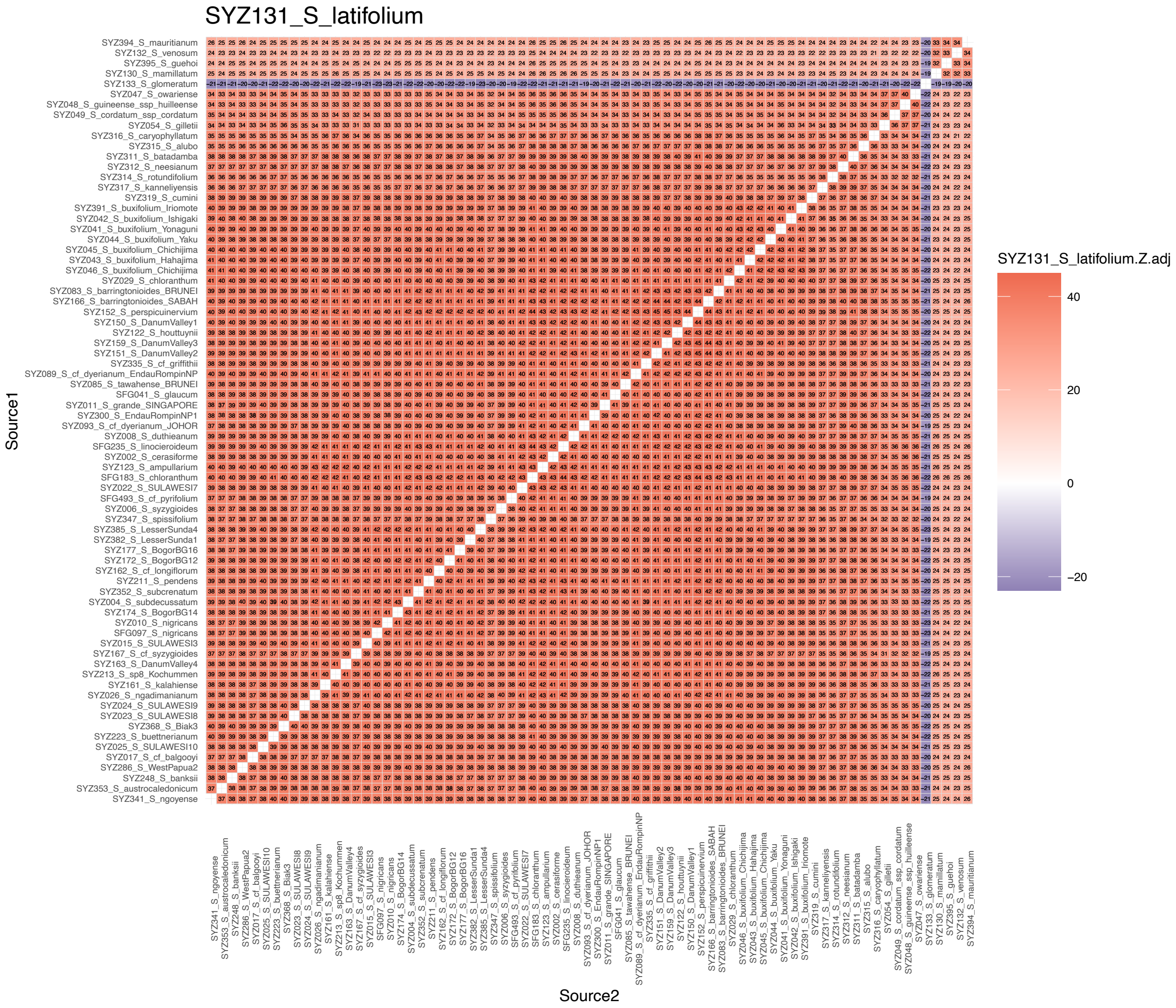

Supplementary Figure 49. F3 heatmap for SYZ131_S_latifolium as target, with other members of the *Syzygium grande* group as Source1 or Source2. Z-scores adjusted for multiple tests are depicted.

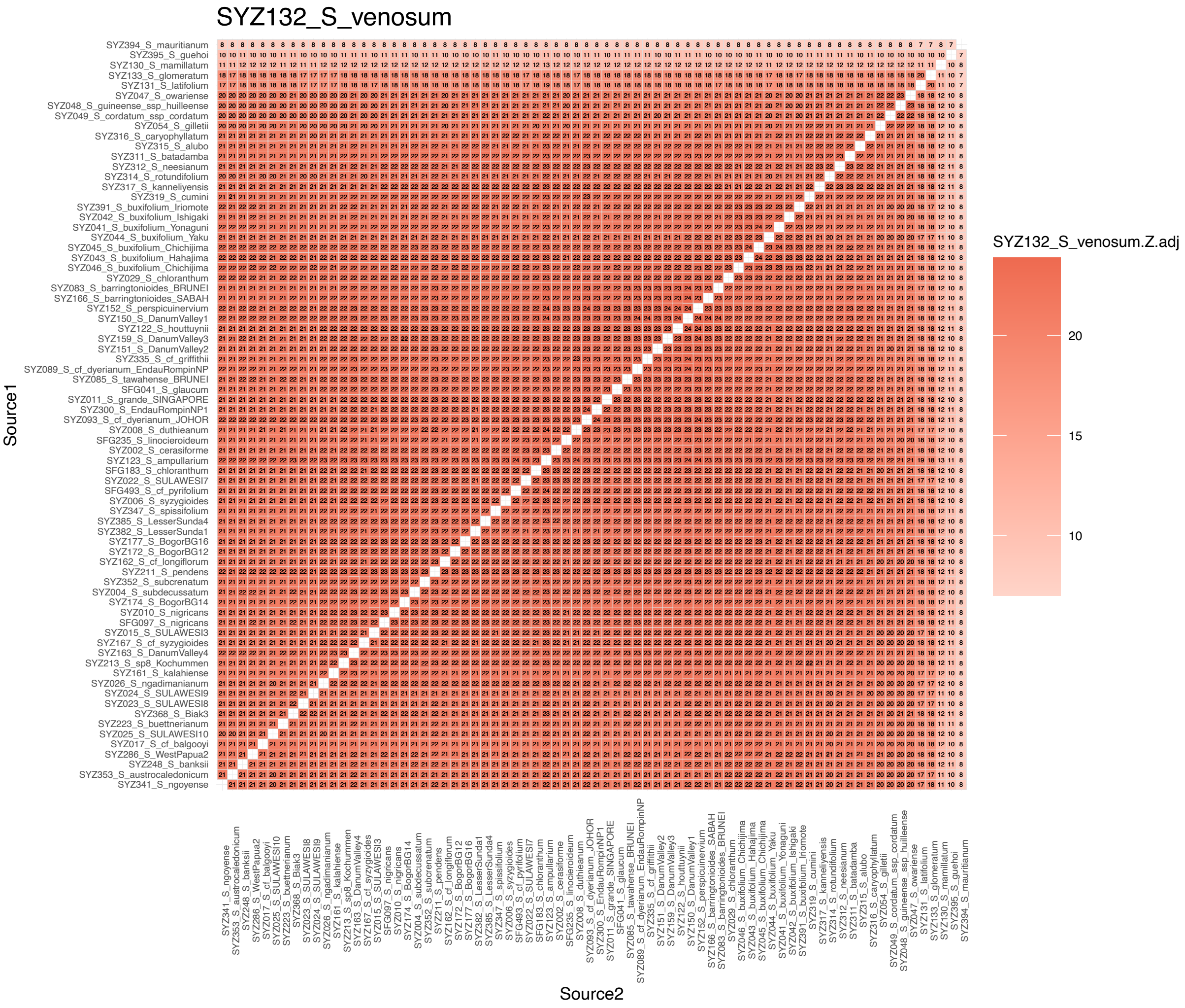

Supplementary Figure 50. F3 heatmap for SYZ132_S_venosum as target, with other members of the *Syzygium grande* group as Source1 or Source2. Z-scores adjusted for multiple tests are depicted.

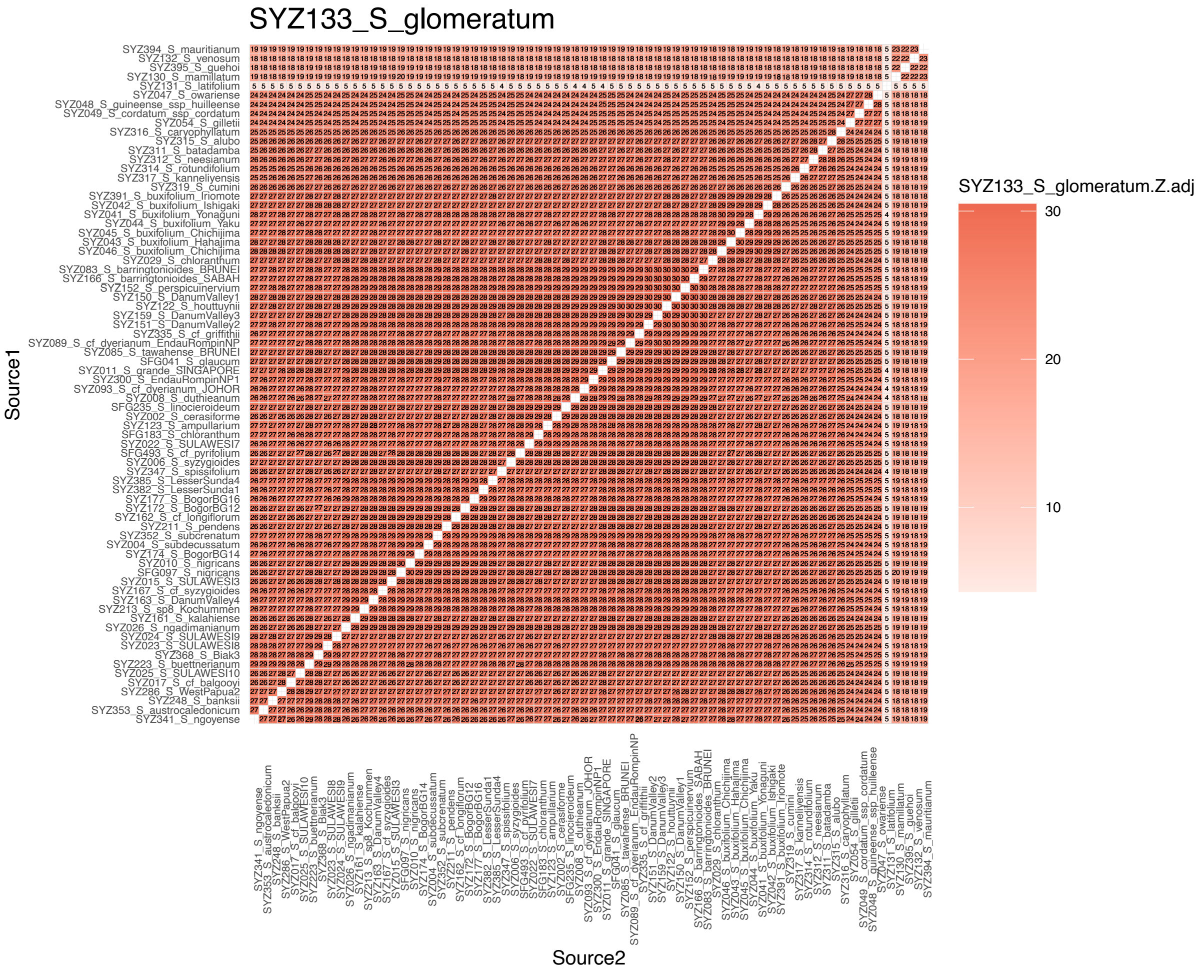

Supplementary Figure 51. F3 heatmap for SYZ133_S_glomeratum as target, with other members of the *Syzygium grande* group as Source1 or Source2. Z-scores adjusted for multiple tests are depicted.

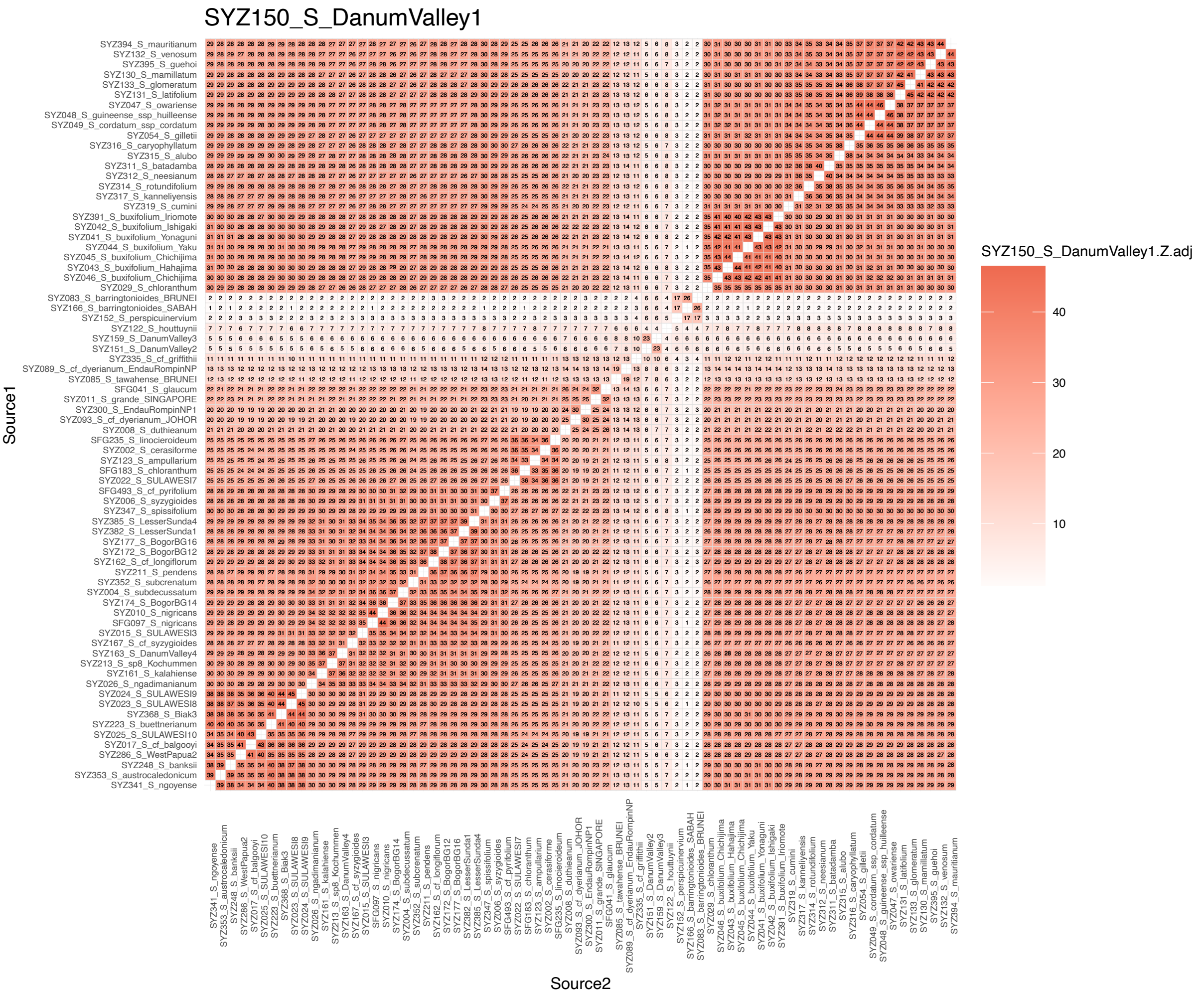

Supplementary Figure 52. F3 heatmap for SYZ150_S_DanumValley1 as target, with other members of the *Syzygium grande* group as Source1 or Source2. *Z*-scores adjusted for multiple tests are depicted.

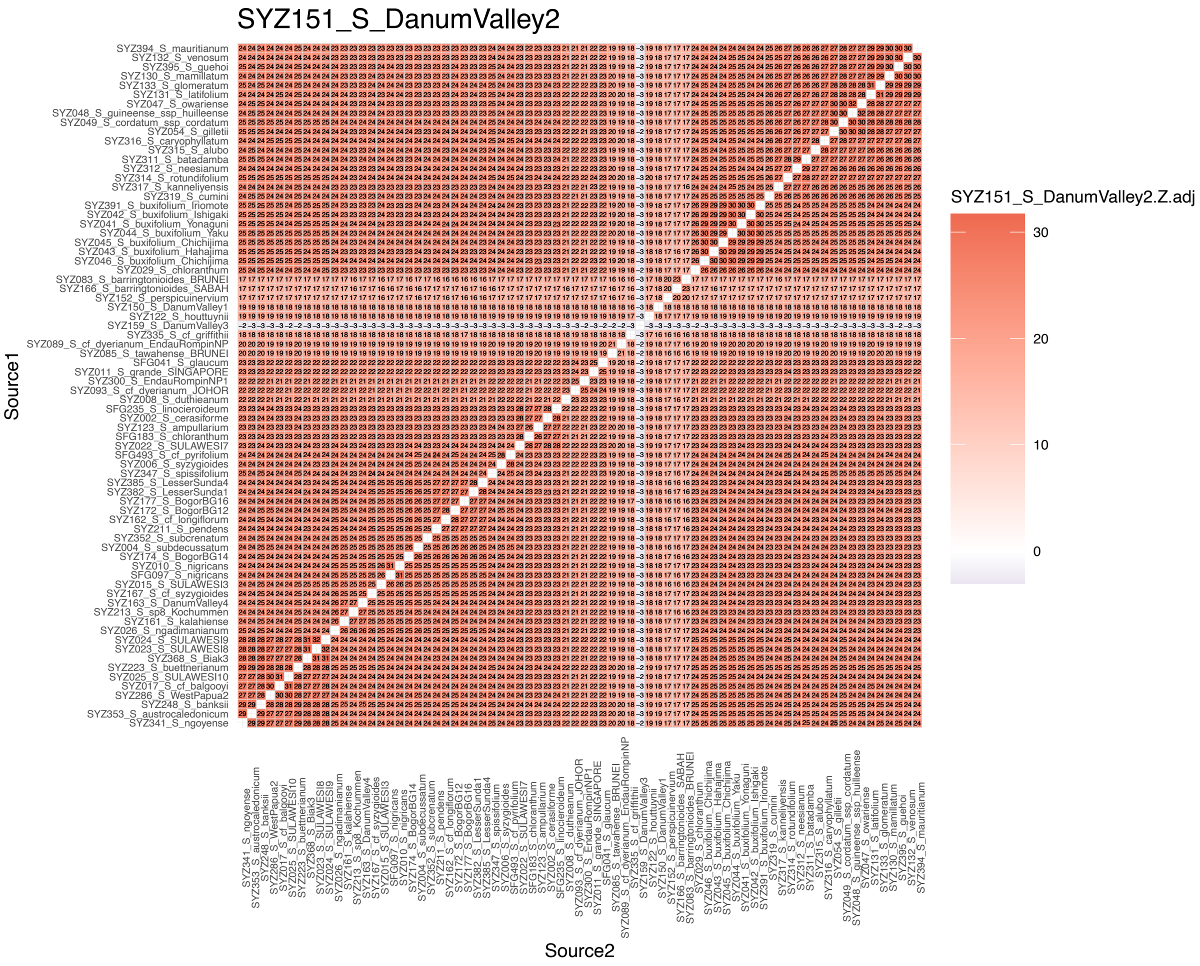

Supplementary Figure 53. F3 heatmap for SYZ151_S_DanumValley2 as target, with other members of the *Syzygium grande* group as Source1 or Source2. *Z*-scores adjusted for multiple tests are depicted.

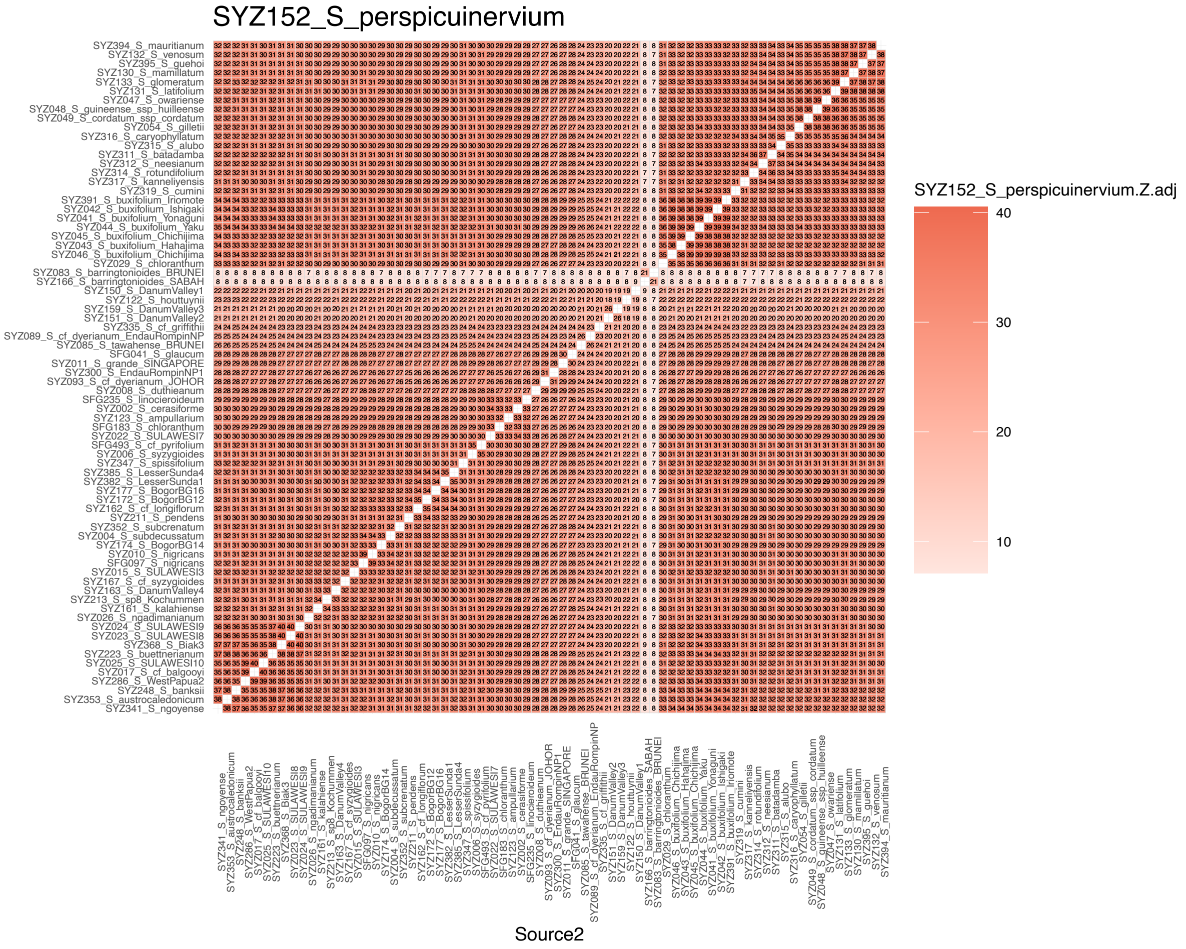

Supplementary Figure 54. F3 heatmap for SYZ152_S_perspicuinervium as target, with other members of the *Syzygium grande* group as Source1 or Source2. Z-scores adjusted for multiple tests are depicted.

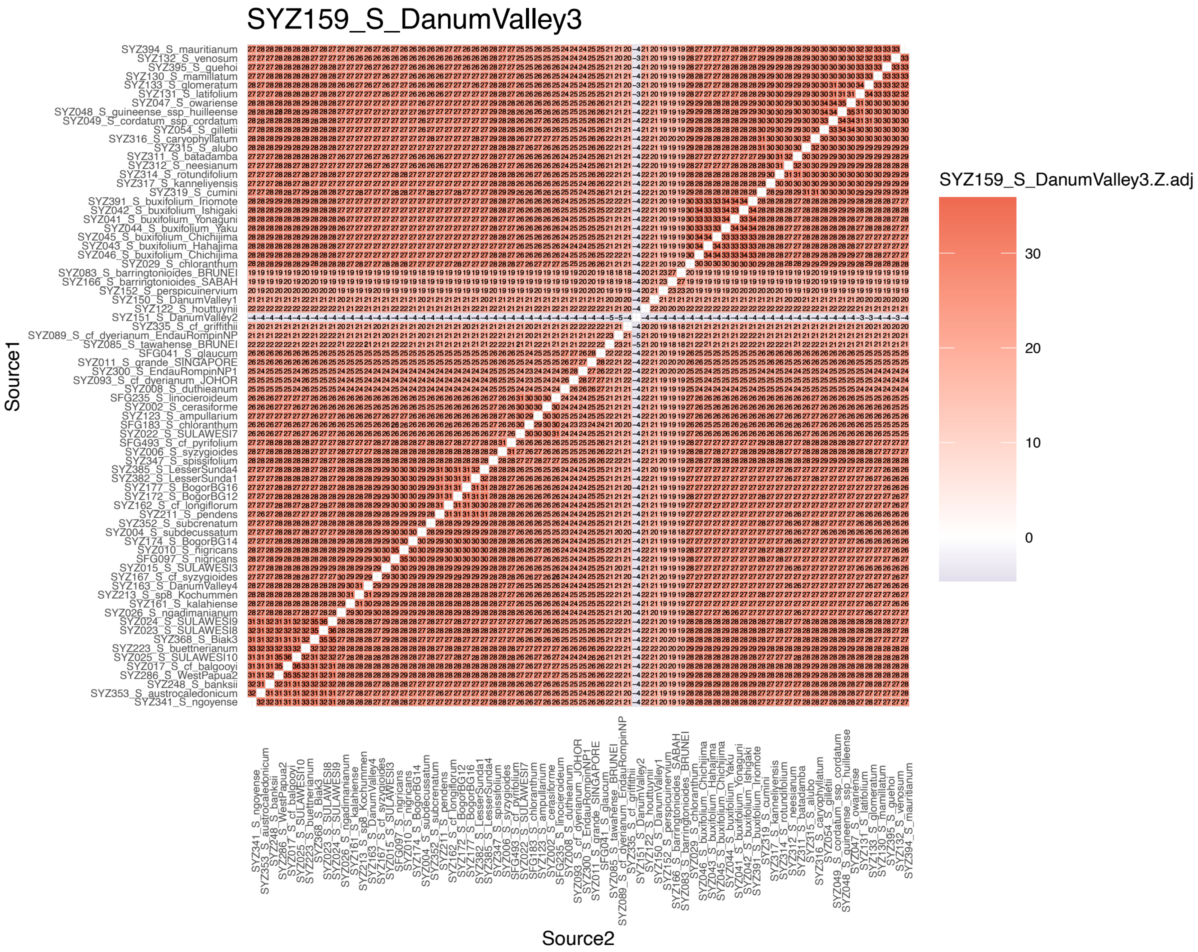

Supplementary Figure 55. F3 heatmap for SYZ159_S_DanumValley3 as target, with other members of the *Syzygium grande* group as Source1 or Source2. *Z*-scores adjusted for multiple tests are depicted.

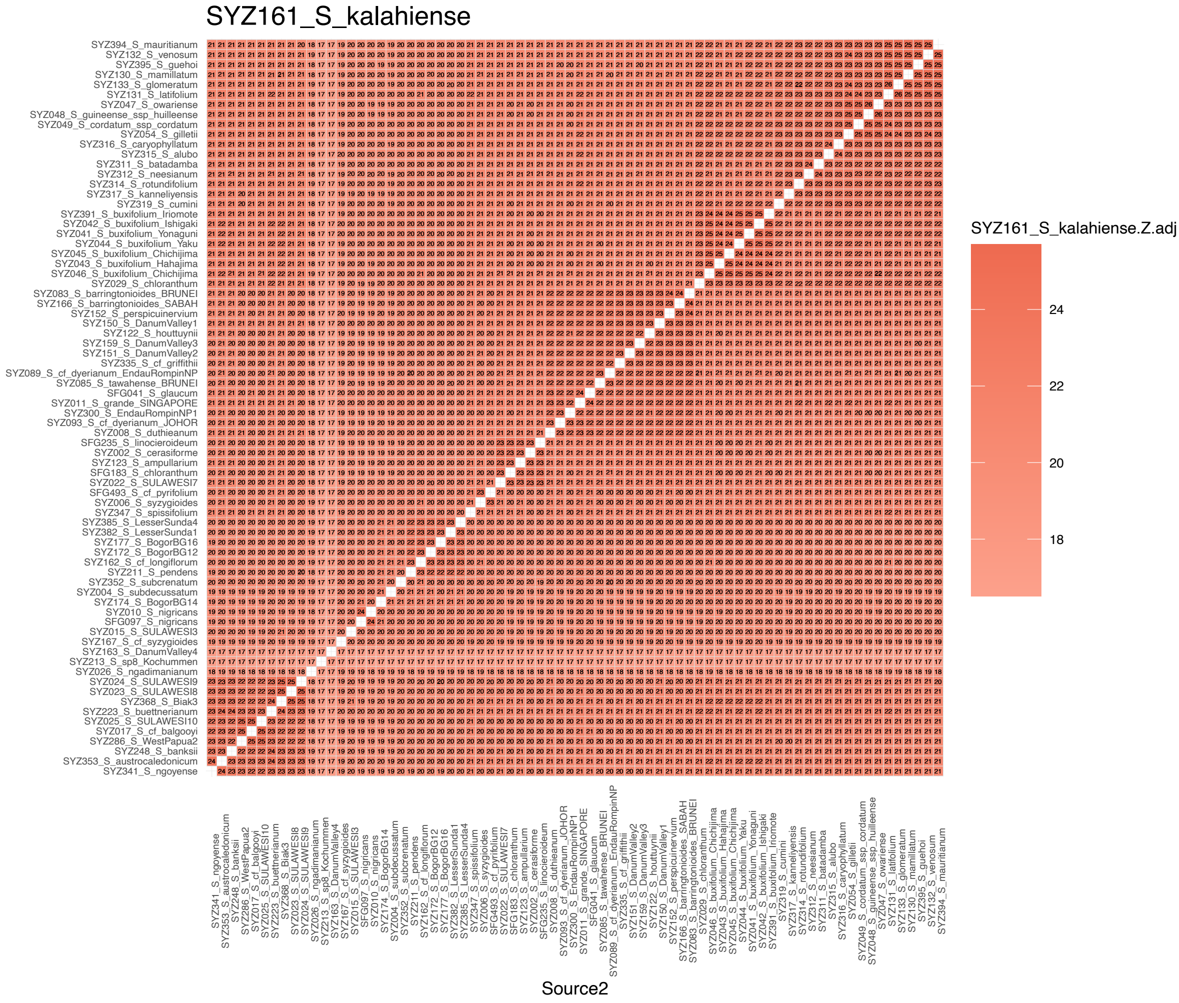

Supplementary Figure 56. F3 heatmap for SYZ161_S_kalahiense as target, with other members of the *Syzygium grande* group as Source1 or Source2. Z-scores adjusted for multiple tests are depicted.

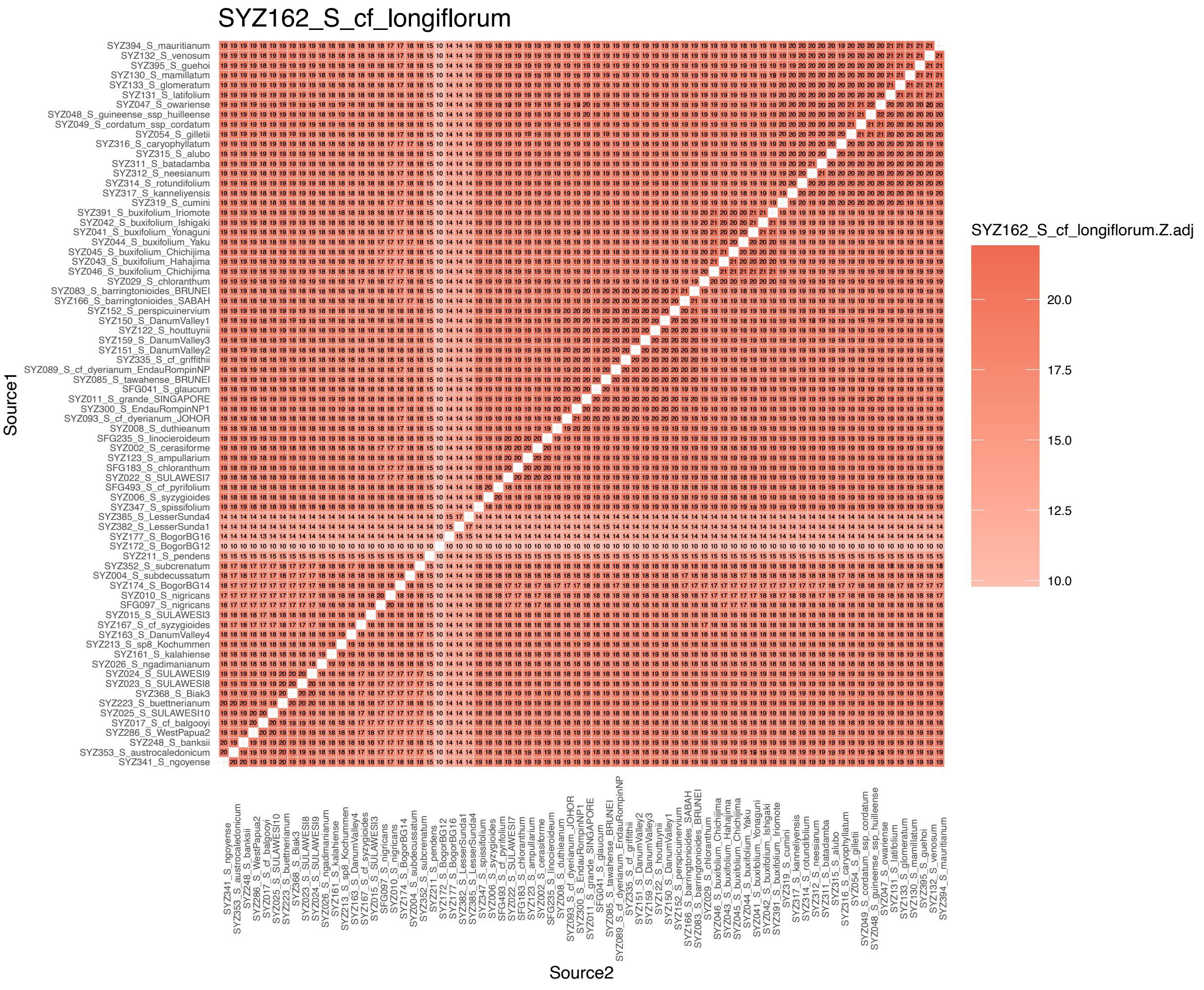

Supplementary Figure 57. F3 heatmap for SYZ162_S_cf_longiflorum as target, with other members of the *Syzygium grande* group as Source1 or Source2. *Z*-scores adjusted for multiple tests are depicted.

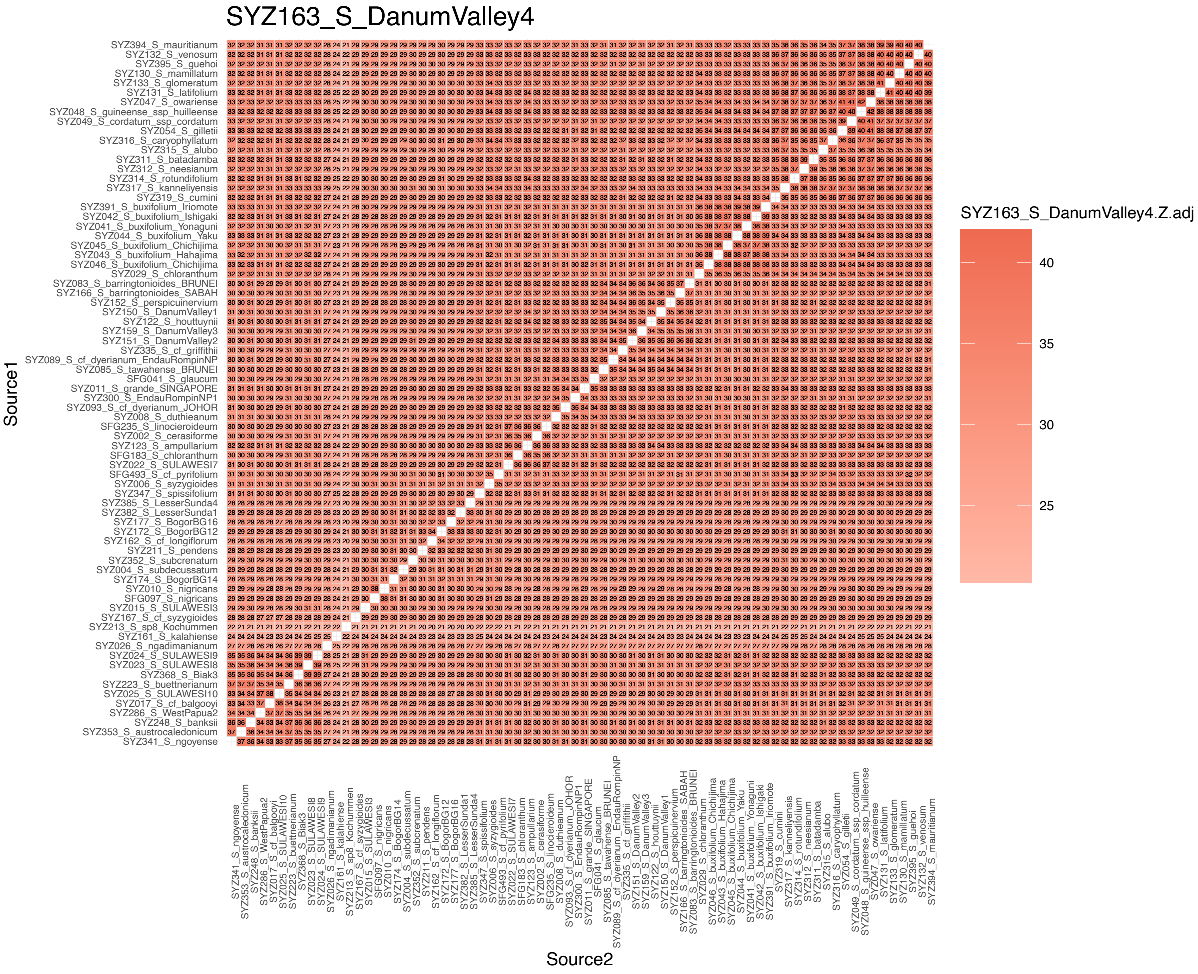

Supplementary Figure 58. F3 heatmap for SYZ163_S_DanumValley4 as target, with other members of the *Syzygium grande* group as Source1 or Source2. *Z*-scores adjusted for multiple tests are depicted.

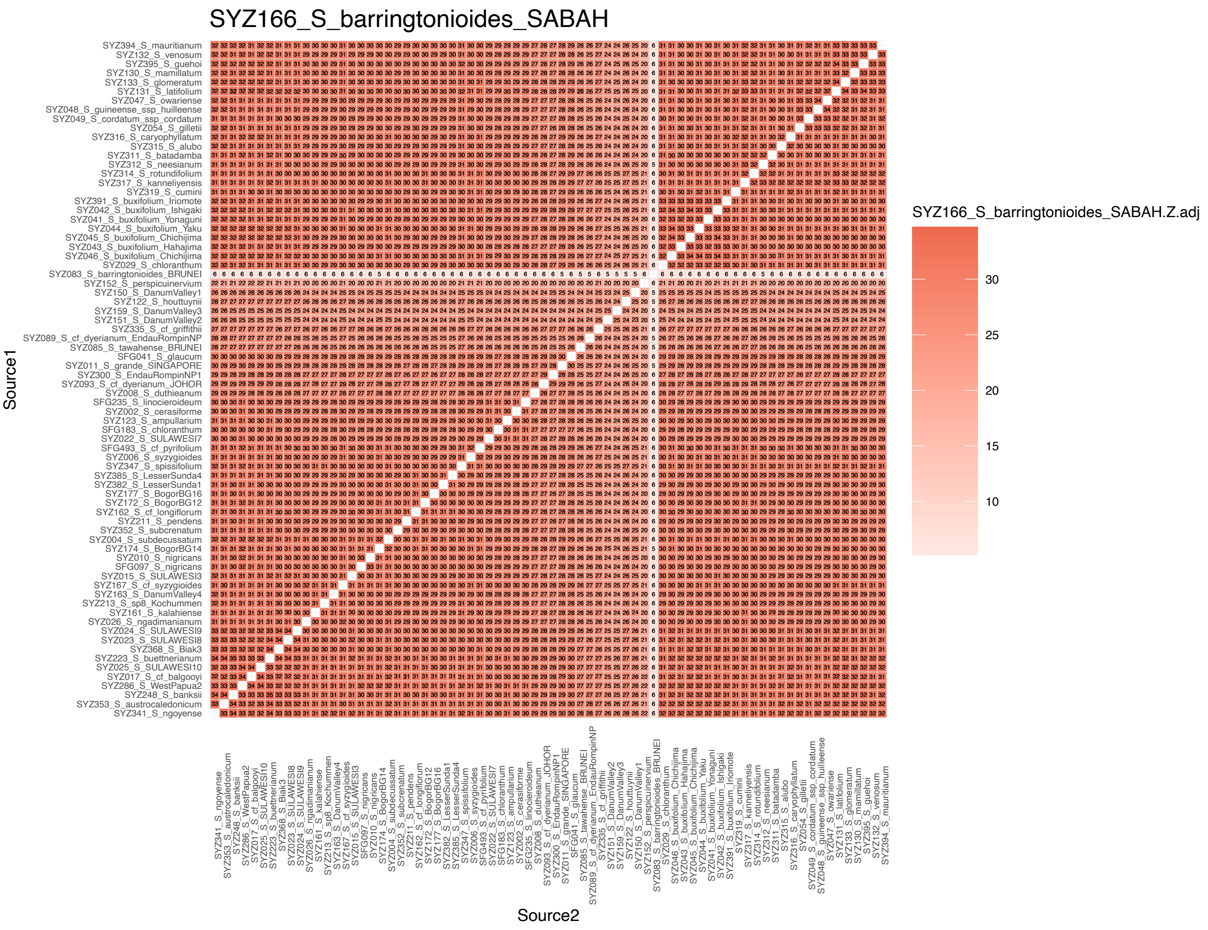

Supplementary Figure 59. F3 heatmap for SYZ166_S_barringtonioides_SABAH as target, with other members of the *Syzygium grande* group as Source1 or Source2. Z-scores adjusted for multiple tests are depicted.

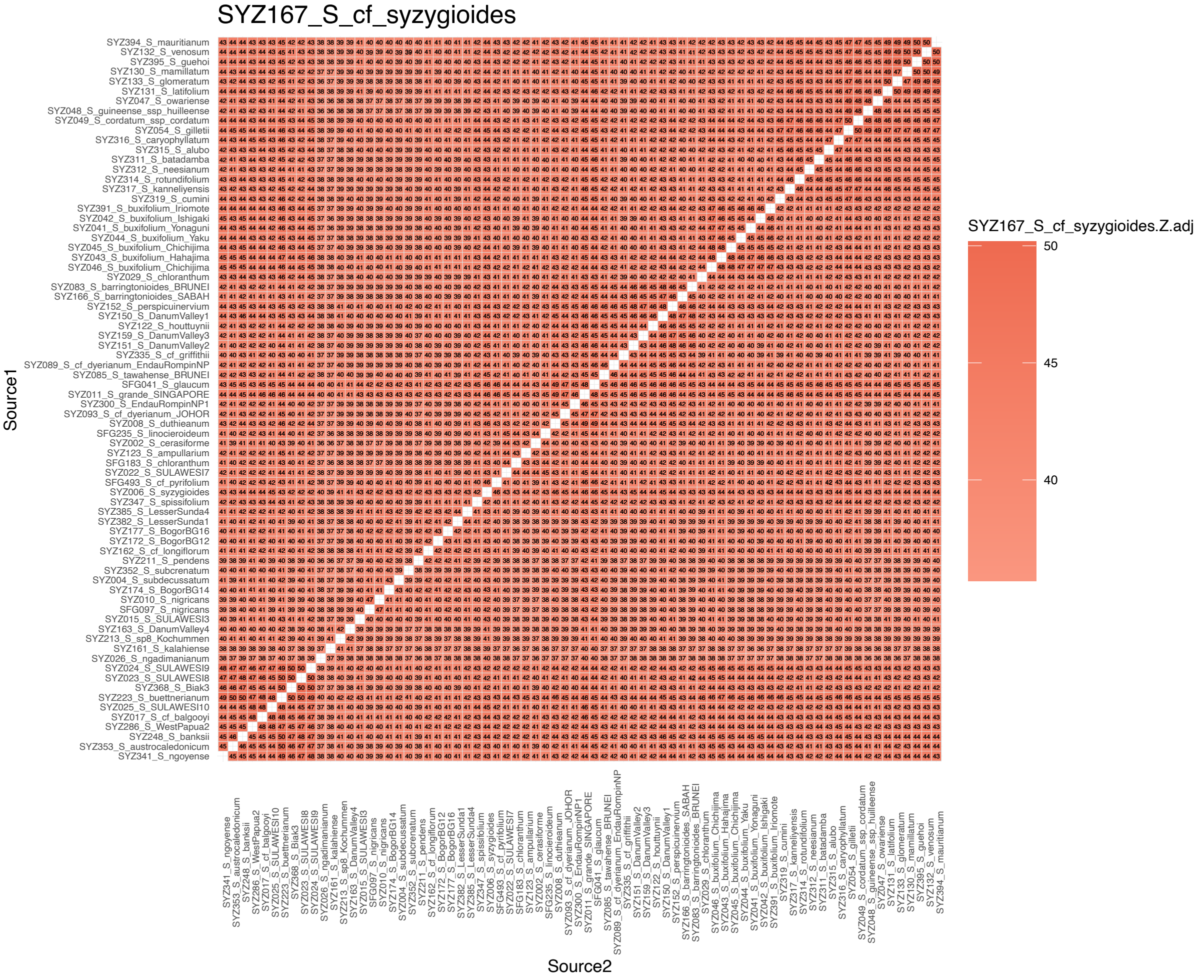

Supplementary Figure 60. F3 heatmap for SYZ167_S_cf_syzygioides as target, with other members of the *Syzygium grande* group as Source1 or Source2. *Z*-scores adjusted for multiple tests are depicted.

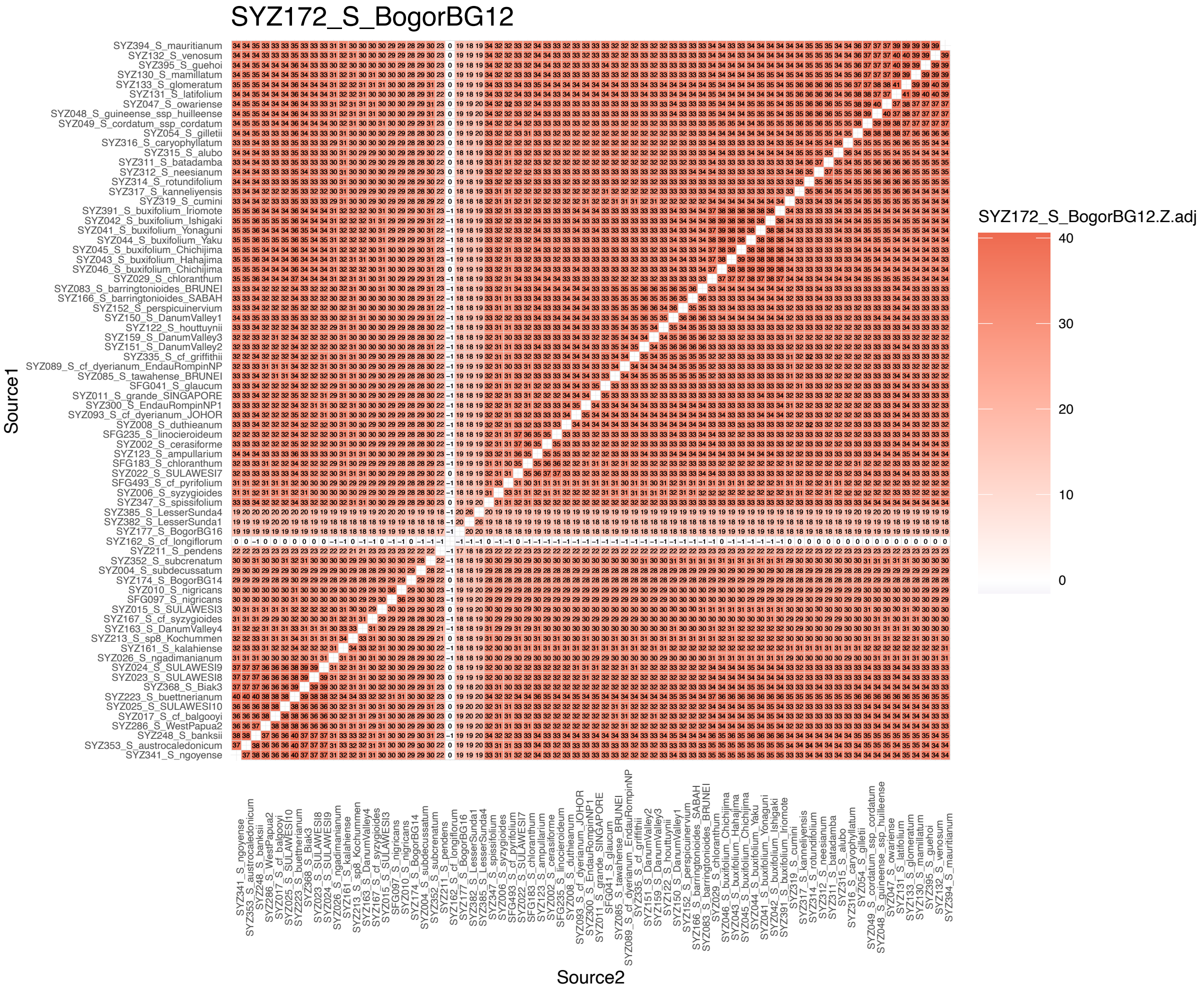

Supplementary Figure 61. F3 heatmap for SYZ172_S_BogorBG12 as target, with other members of the *Syzygium grande* group as Source1 or Source2. Z-scores adjusted for multiple tests are depicted.

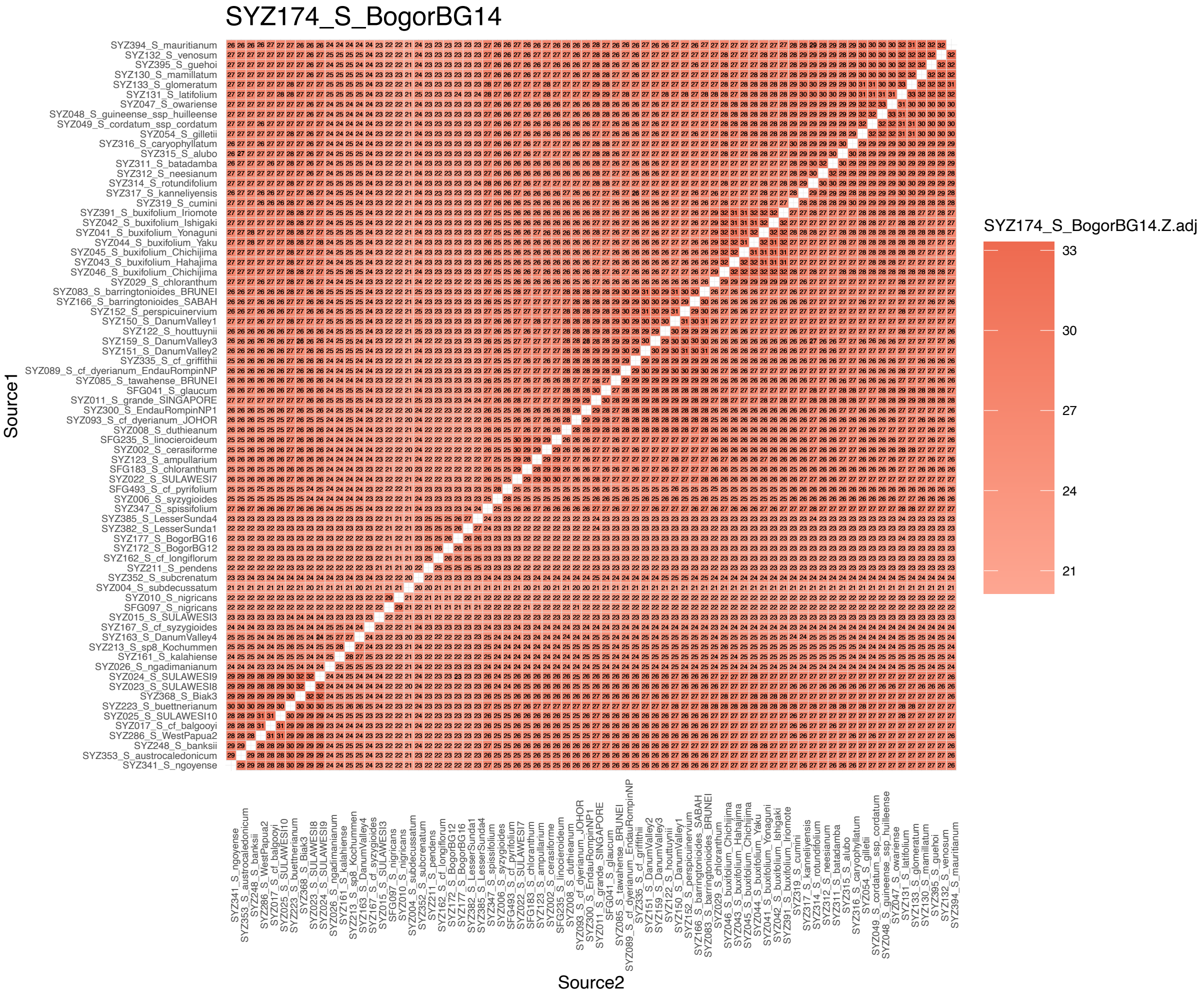

Supplementary Figure 62. F3 heatmap for SYZ174_S_BogorBG14 as target, with other members of the *Syzygium grande* group as Source1 or Source2. Z-scores adjusted for multiple tests are depicted.

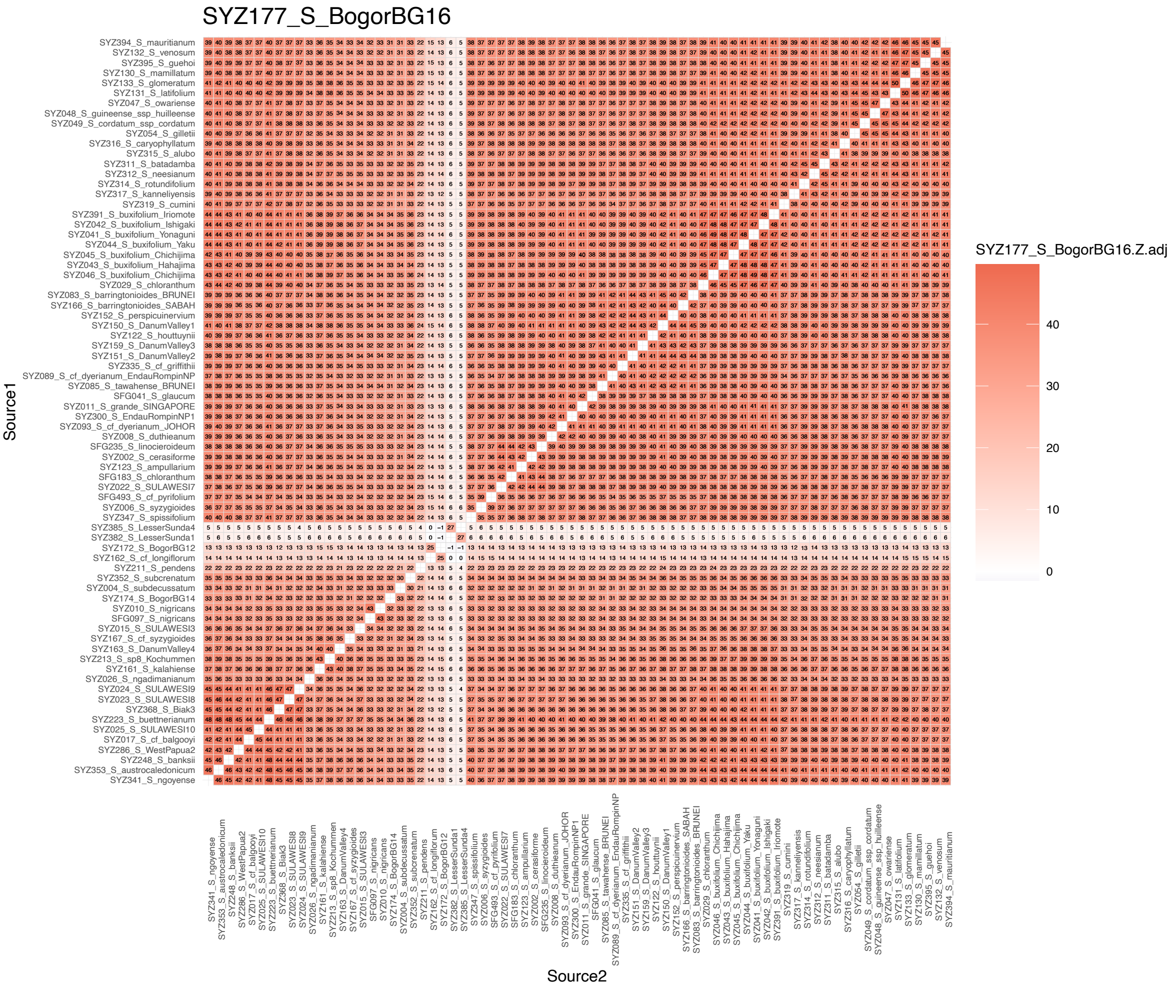

Supplementary Figure 63. F3 heatmap for SYZ177_S_BogorBG16 as target, with other members of the *Syzygium grande* group as Source1 or Source2. *Z*-scores adjusted for multiple tests are depicted.

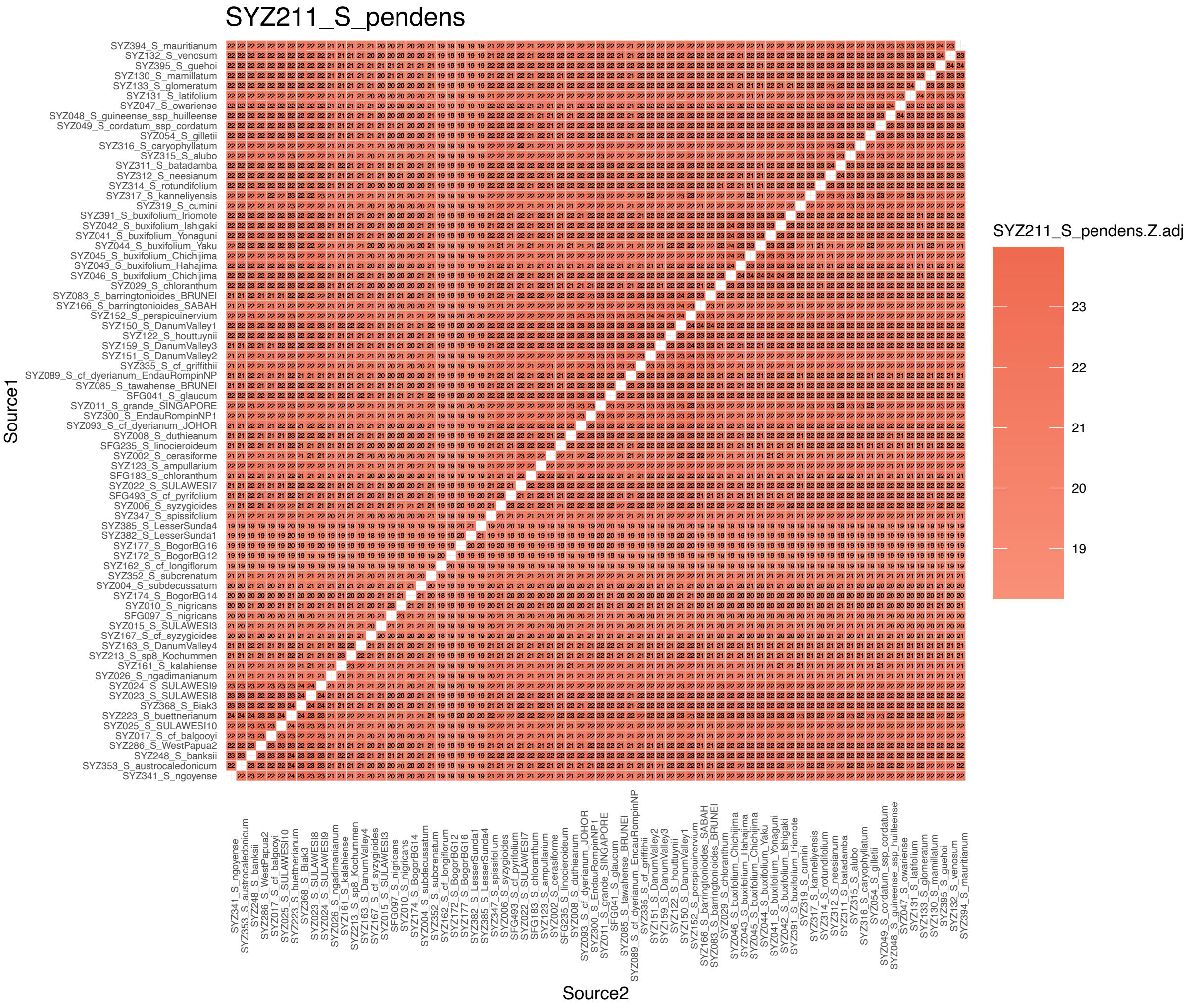

Supplementary Figure 64. F3 heatmap for SYZ211_S_pendens as target, with other members of the *Syzygium grande* group as Source1 or Source2. Z-scores adjusted for multiple tests are depicted.

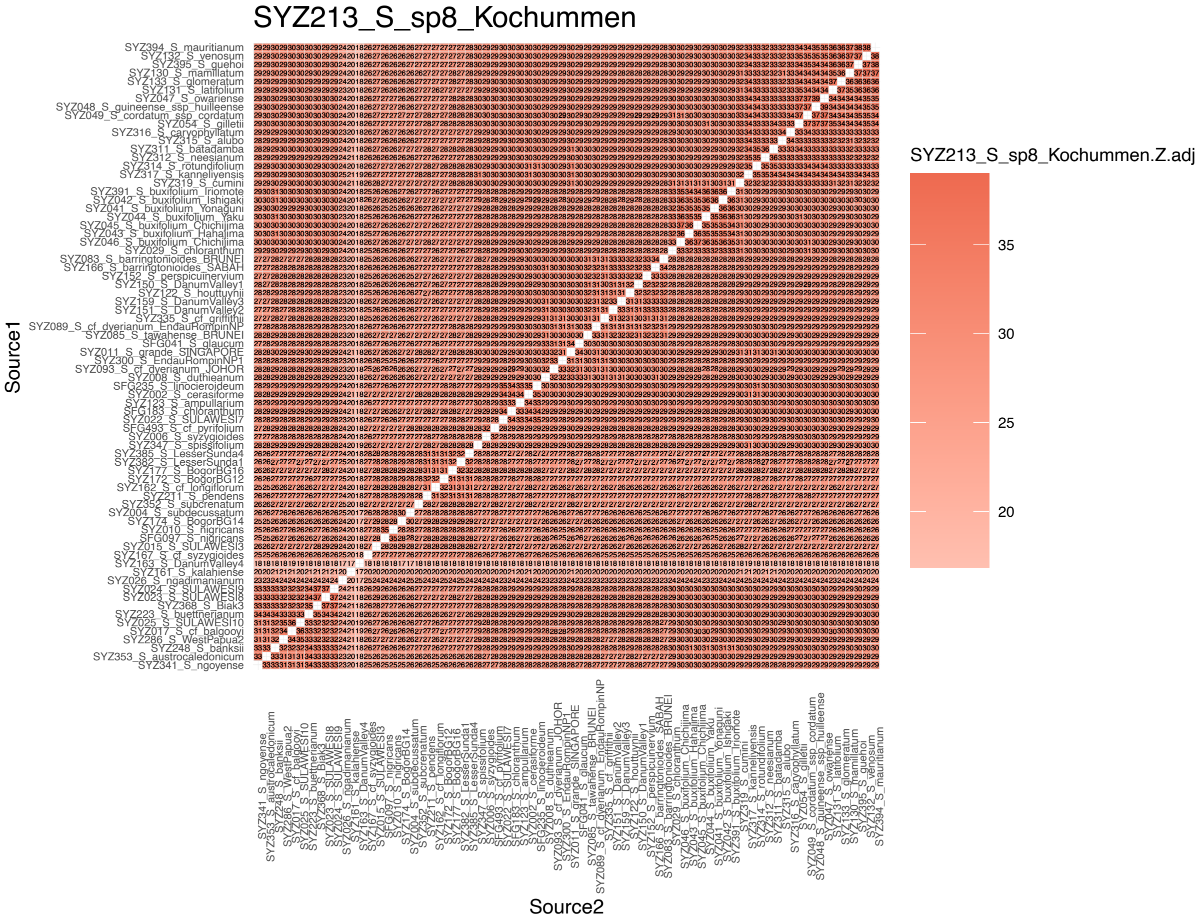

Supplementary Figure 65. F3 heatmap for SYZ213_S_sp8_Kochummen as target, with other members of the *Syzygium grande* group as Source1 or Source2. Z-scores adjusted for multiple tests are depicted.

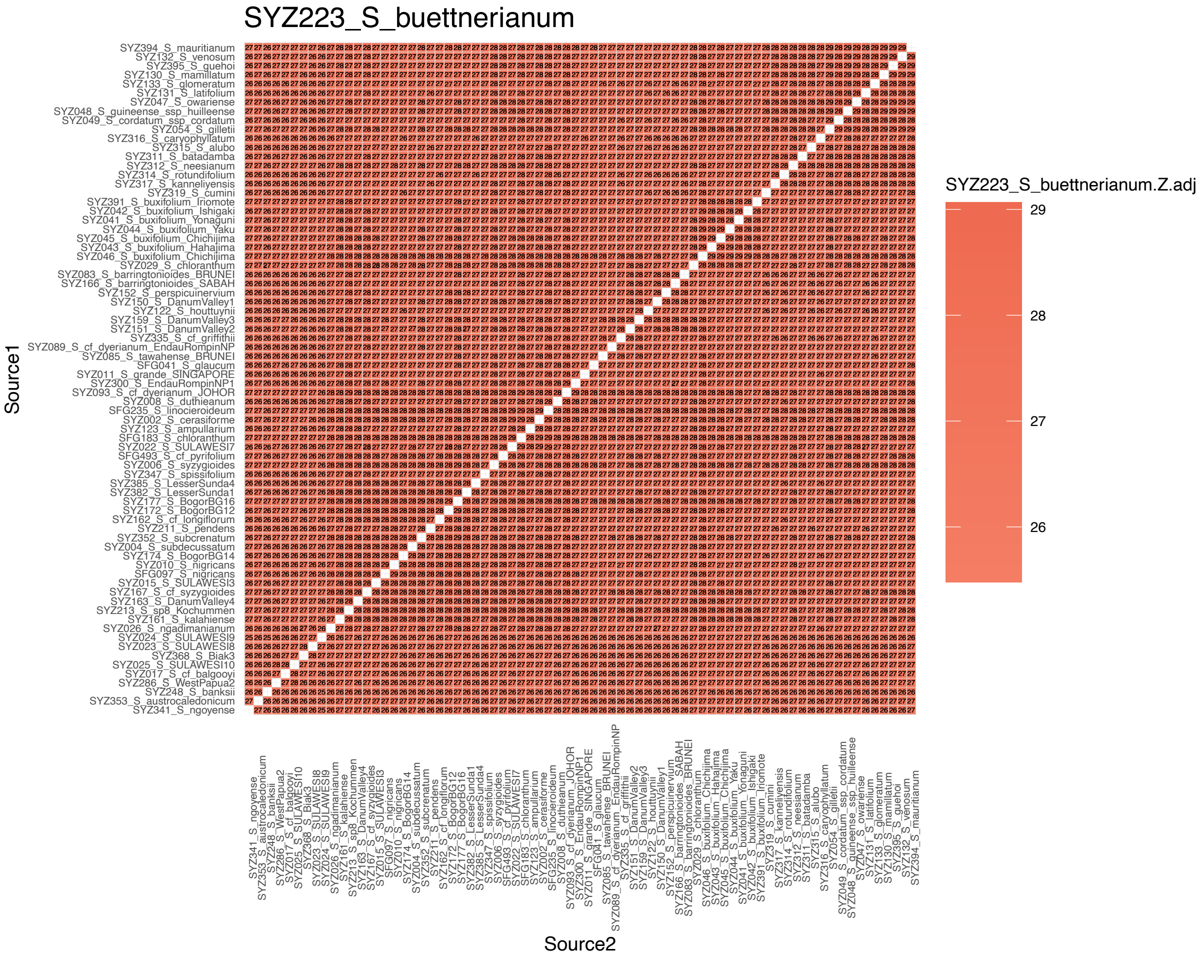

Supplementary Figure 66. F3 heatmap for SYZ223_S_buettnerianum as target, with other members of the *Syzygium grande* group as Source1 or Source2. *Z*-scores adjusted for multiple tests are depicted.

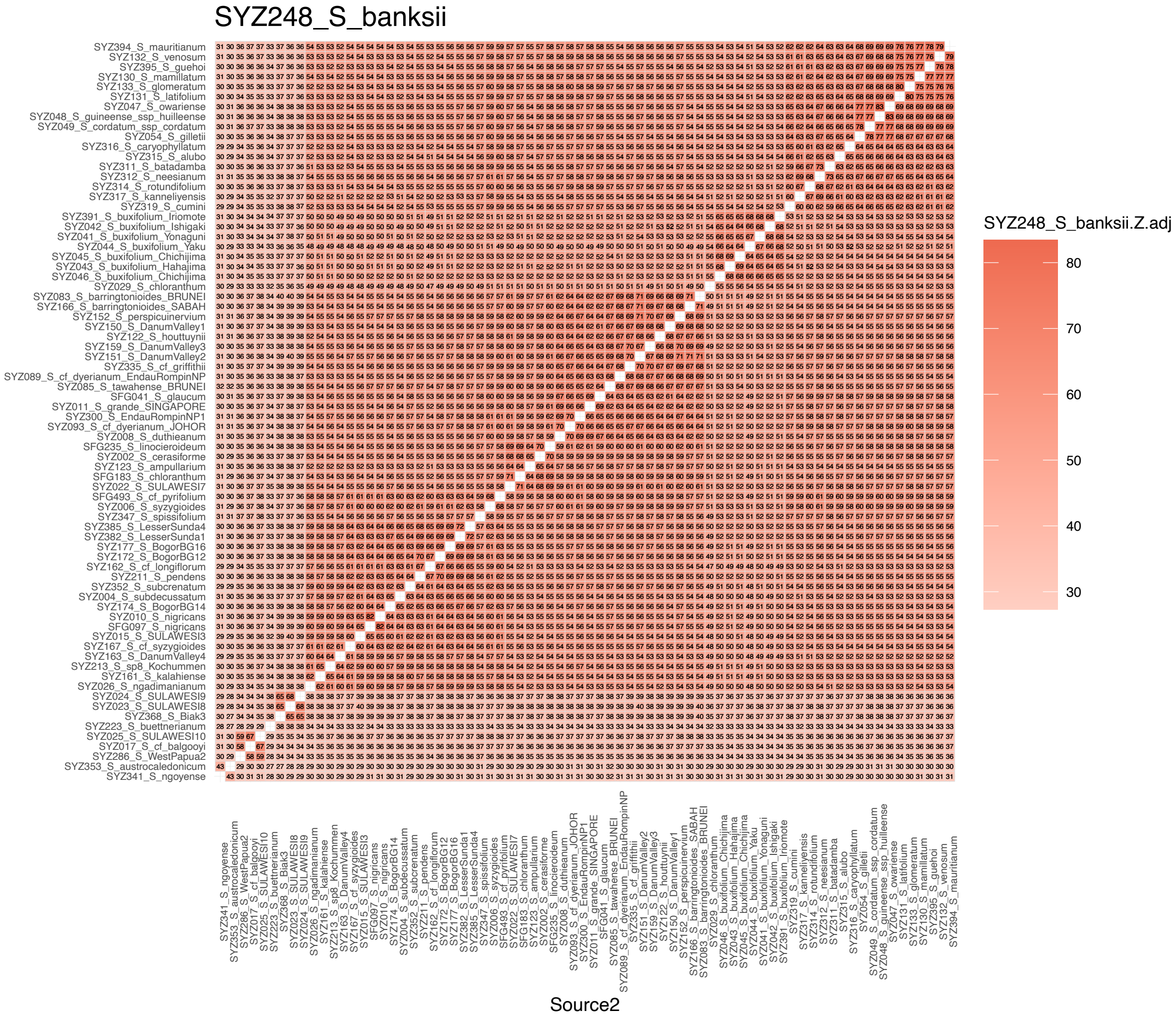

Supplementary Figure 67. F3 heatmap for SYZ248_S_banksii as target, with other members of the *Syzygium grande* group as Source1 or Source2. Z-scores adjusted for multiple tests are depicted.

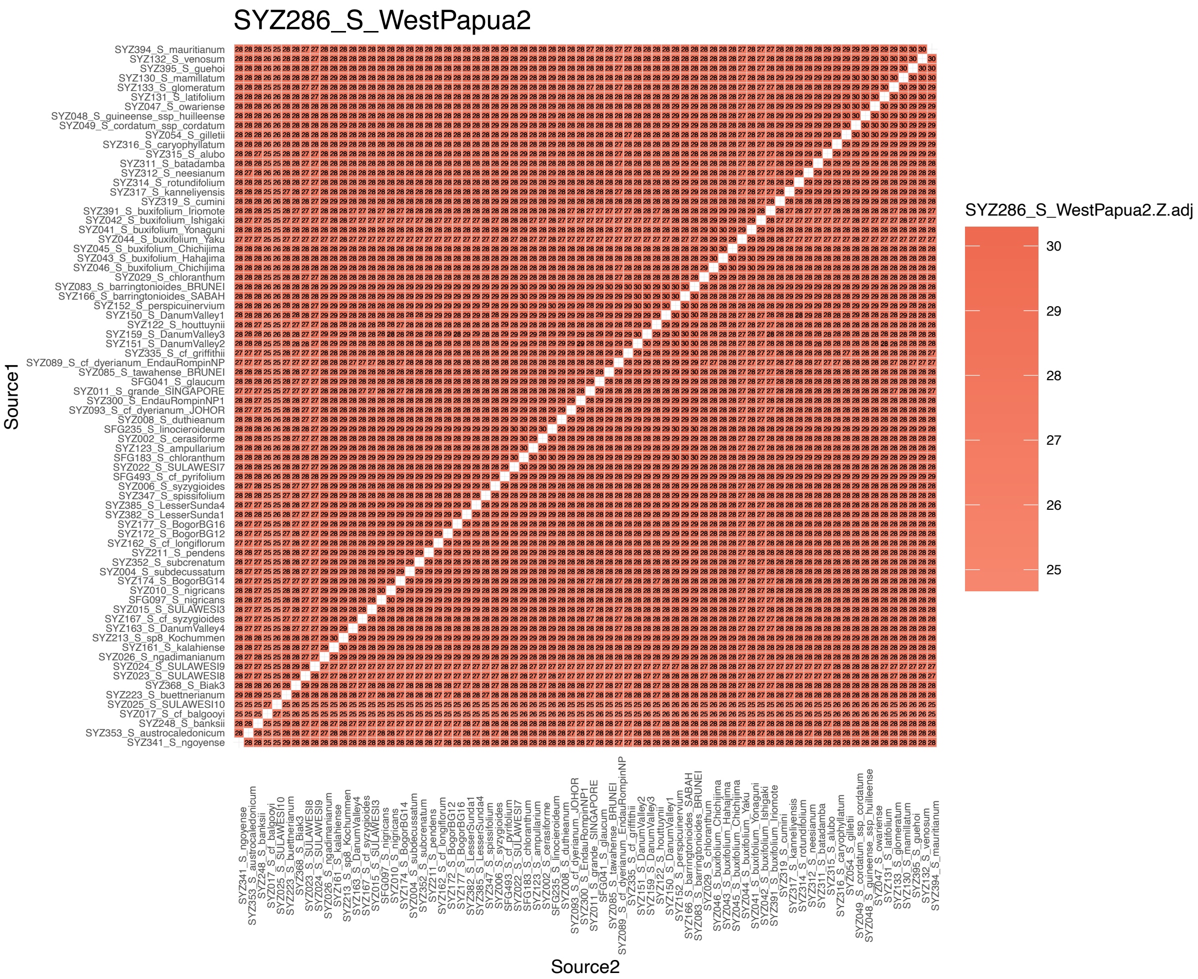

Supplementary Figure 68. F3 heatmap for SYZ286_S_WestPapua2 as target, with other members of the *Syzygium grande* group as Source1 or Source2. *Z*-scores adjusted for multiple tests are depicted.

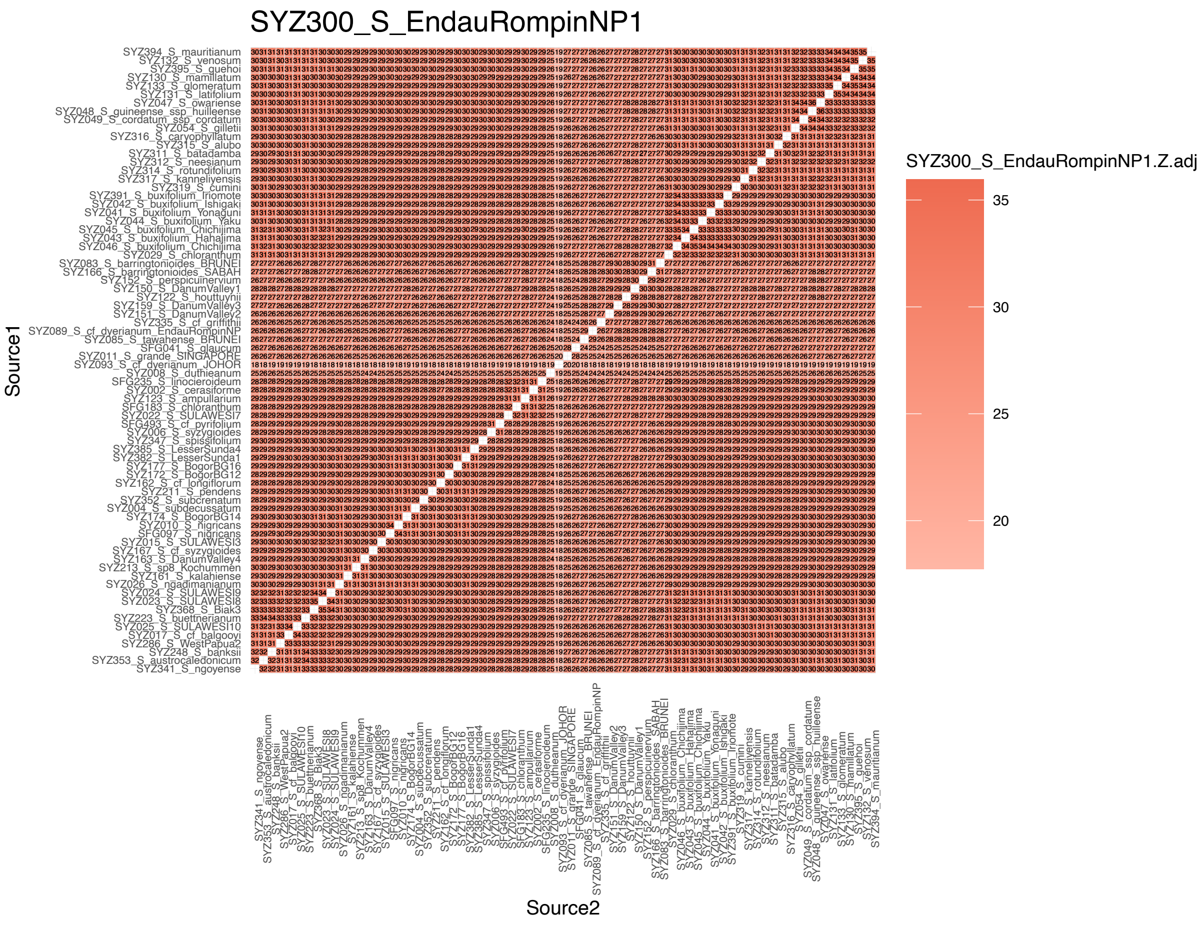

Supplementary Figure 69. F3 heatmap for SYZ300_S_EndauRompinNP1 as target, with other members of the *Syzygium grande* group as Source1 or Source2. *Z*-scores adjusted for multiple tests are depicted.

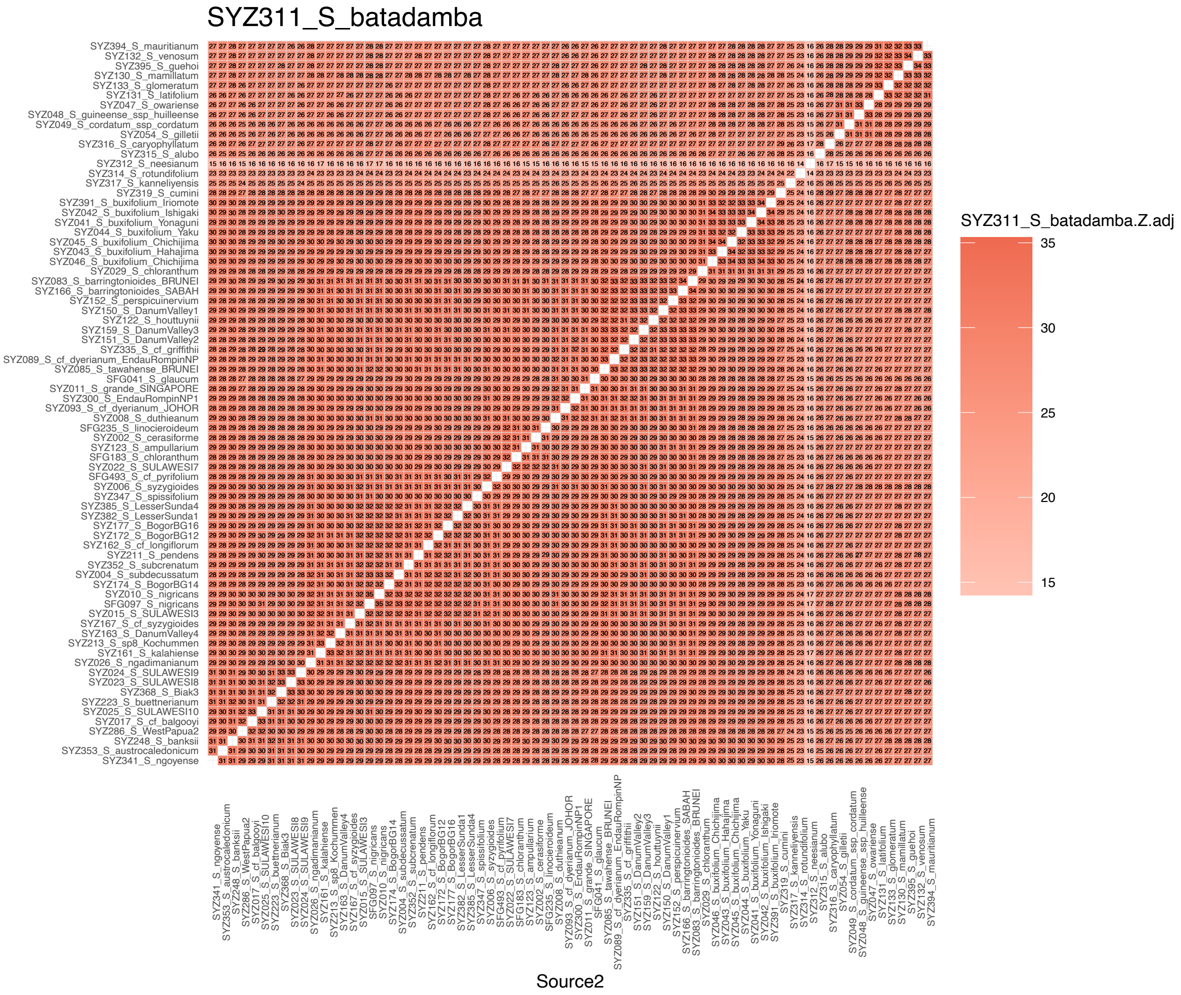

Supplementary Figure 70. F3 heatmap for SYZ311_S_batadamba as target, with other members of the *Syzygium grande* group as Source1 or Source2. Z-scores adjusted for multiple tests are depicted.

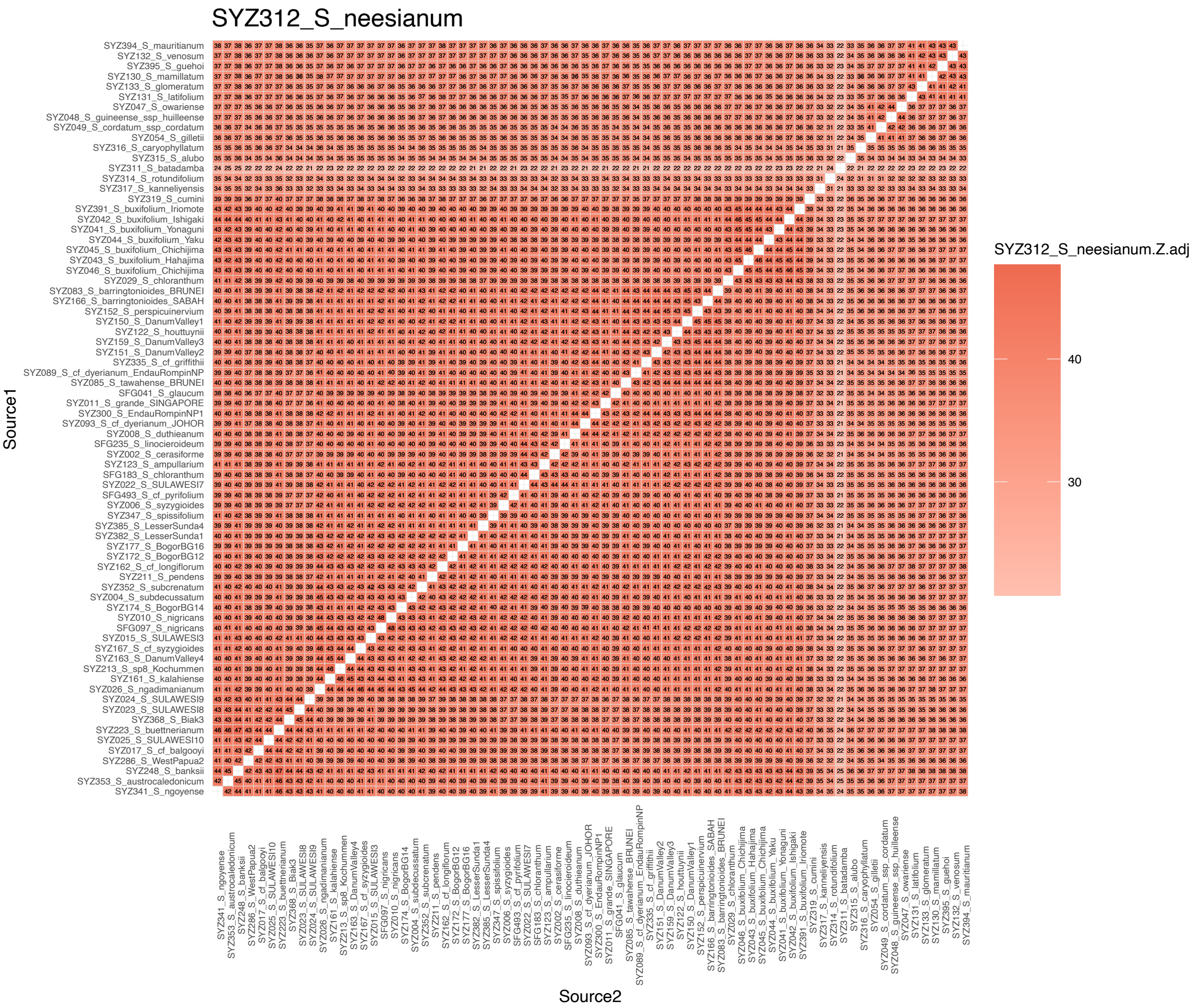

Supplementary Figure 71. F3 heatmap for SYZ312_S_neesianum as target, with other members of the *Syzygium grande* group as Source1 or Source2. Z-scores adjusted for multiple tests are depicted.

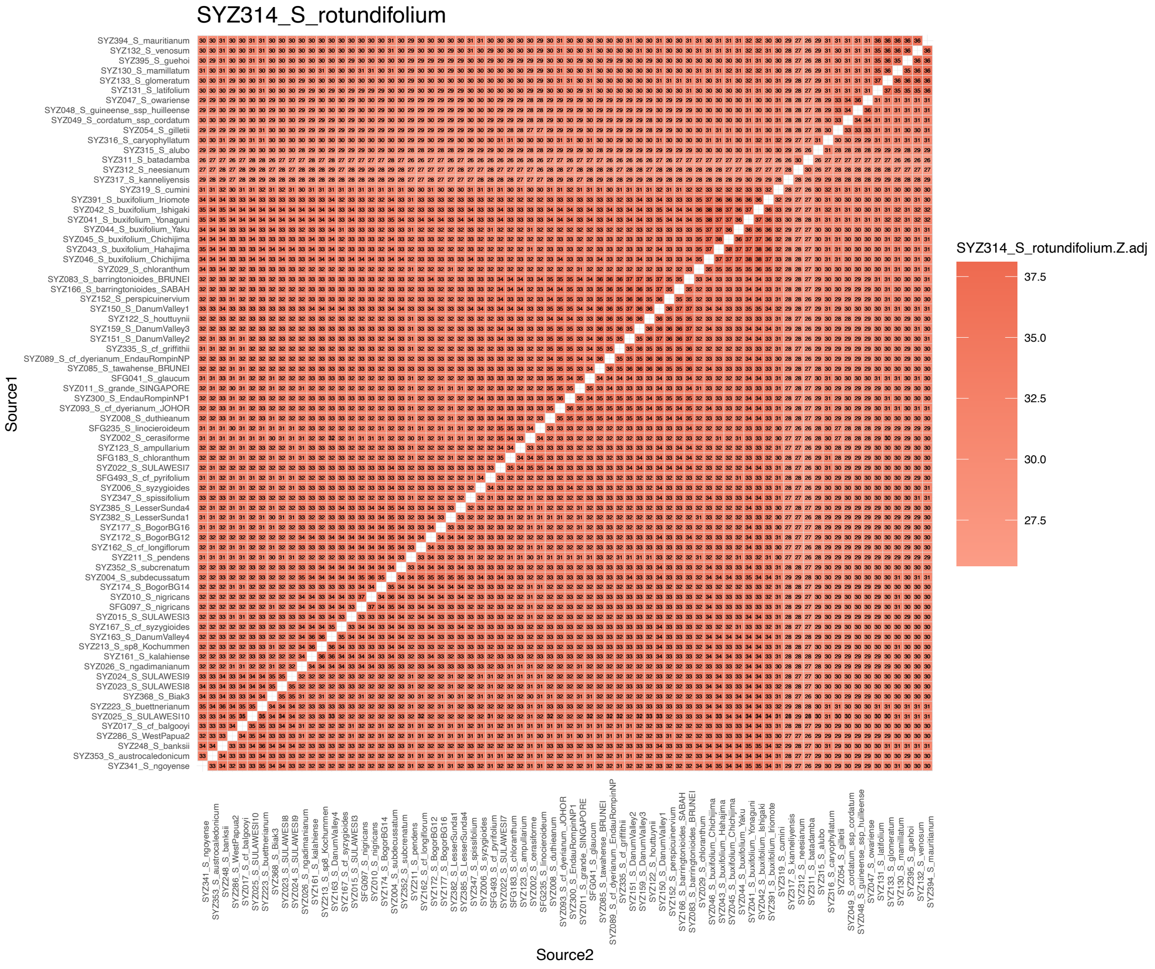

Supplementary Figure 72. F3 heatmap for SYZ314_S_rotundifolium as target, with other members of the *Syzygium grande* group as Source1 or Source2. *Z*-scores adjusted for multiple tests are depicted.

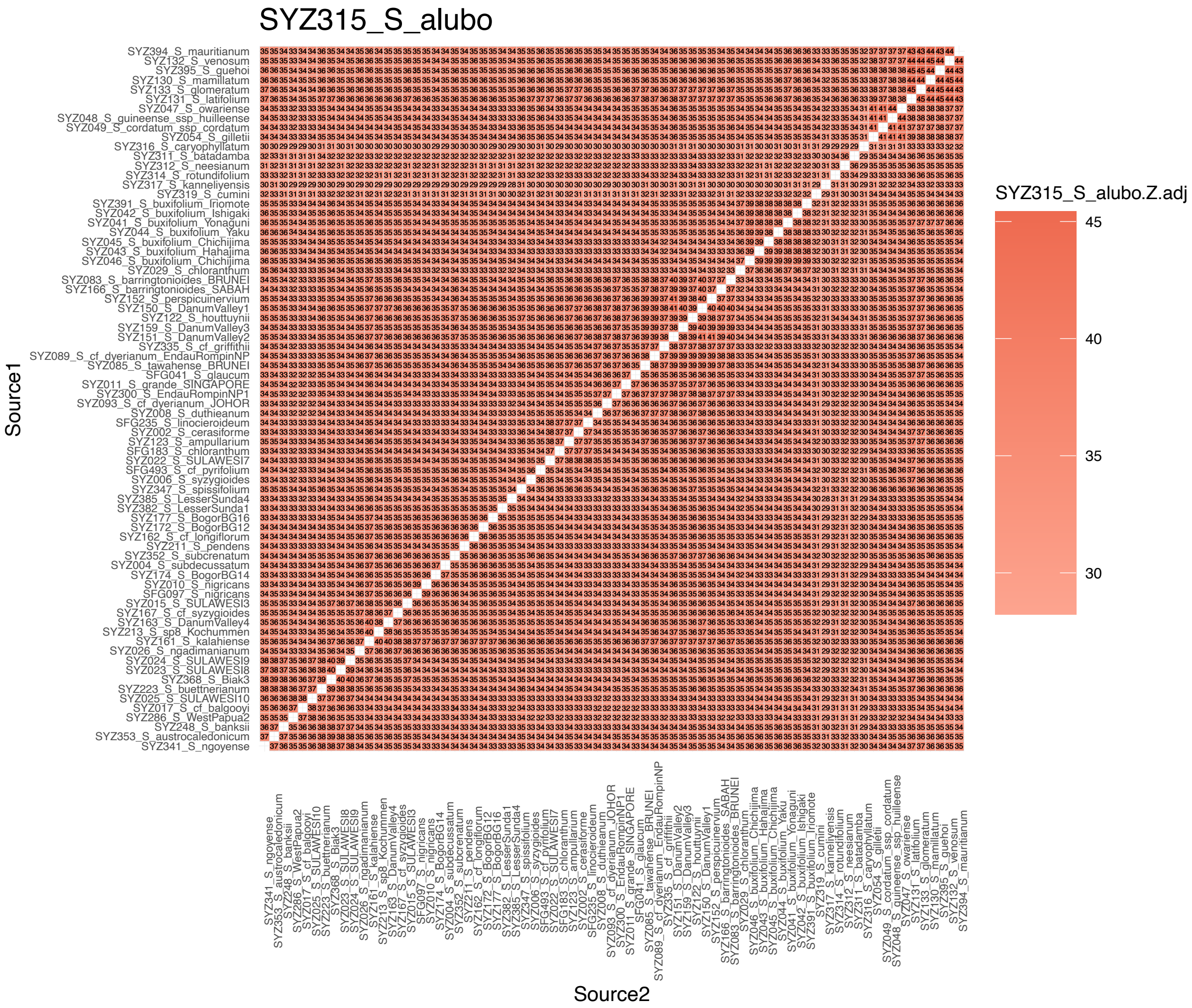

Supplementary Figure 73. F3 heatmap for SYZ315_S_alubo as target, with other members of the *Syzygium grande* group as Source1 or Source2. *Z*-scores adjusted for multiple tests are depicted.

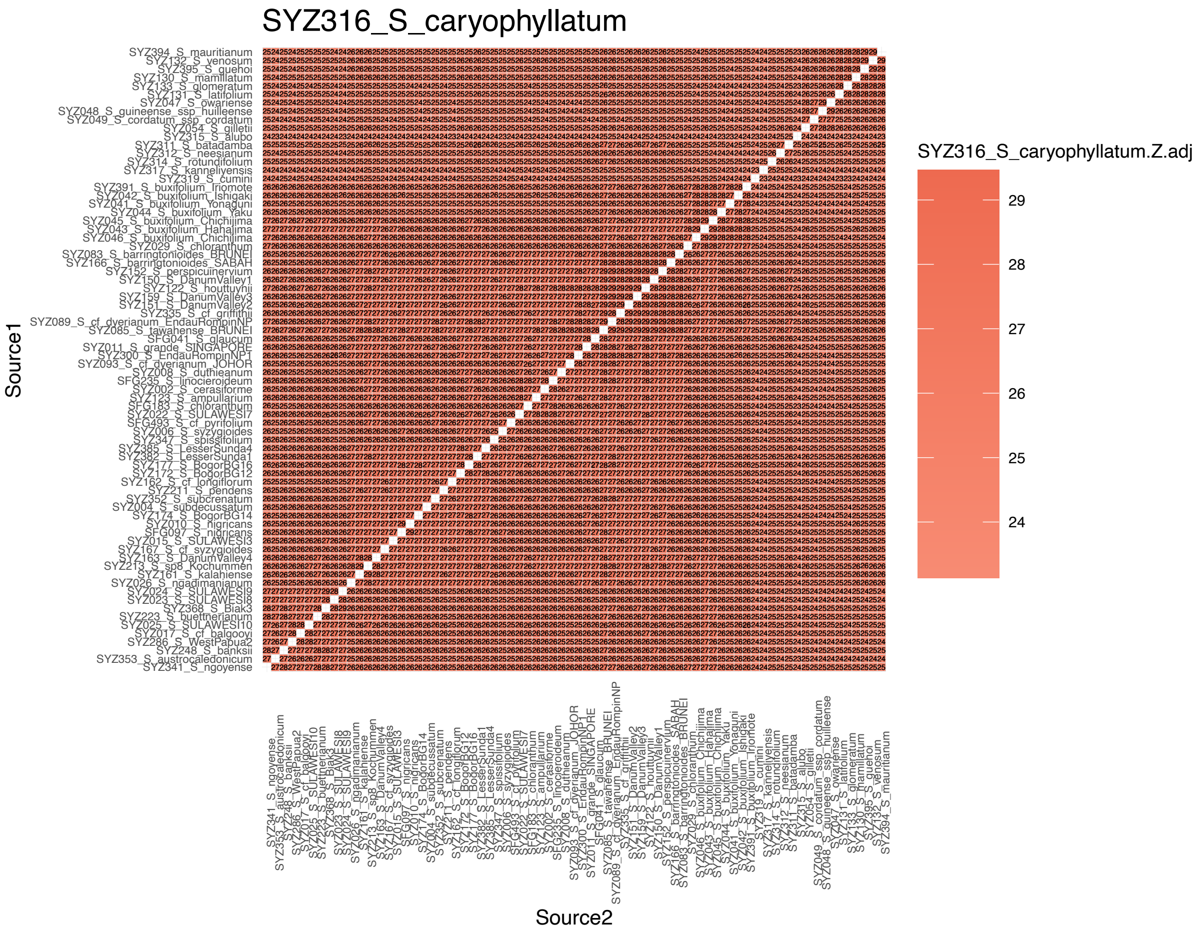

Supplementary Figure 74. F3 heatmap for SYZ316_S_caryophyllatum as target, with other members of the *Syzygium grande* group as Source1 or Source2. *Z*-scores adjusted for multiple tests are depicted.

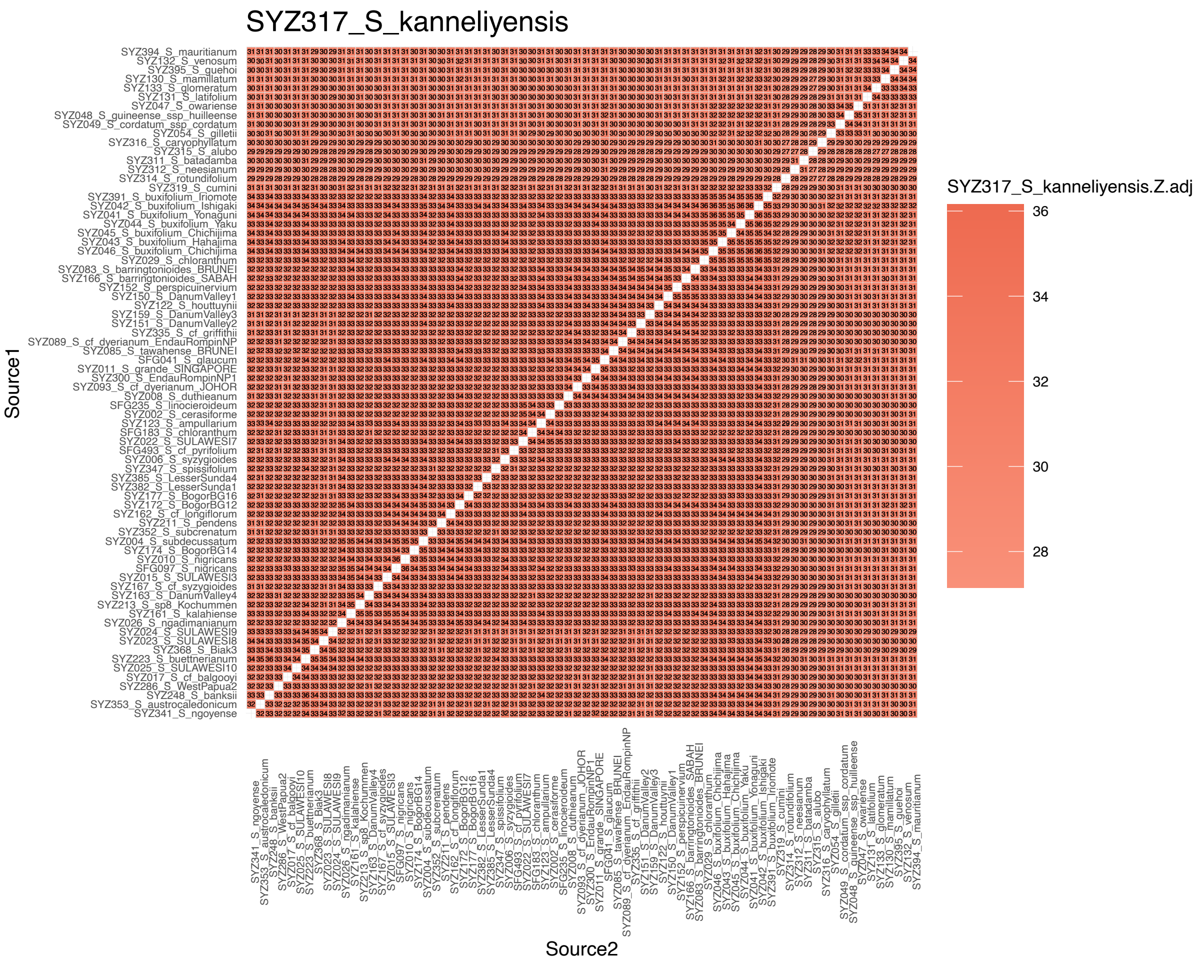

Supplementary Figure 75. F3 heatmap for SYZ317_S_kanneliyensis as target, with other members of the *Syzygium grande* group as Source1 or Source2. *Z*-scores adjusted for multiple tests are depicted.

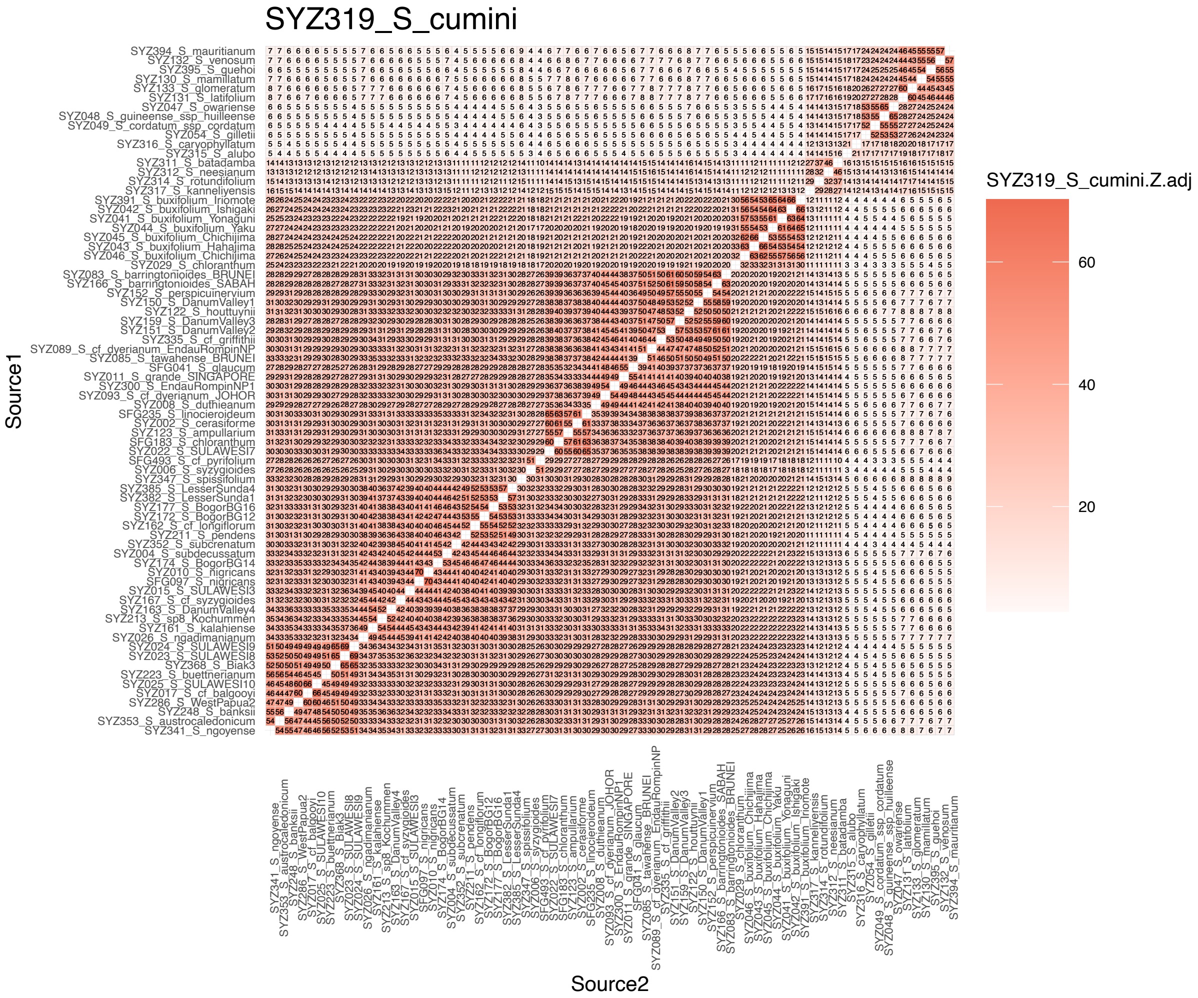

Supplementary Figure 76. F3 heatmap for SYZ319_S_cumini as target, with other members of the *Syzygium grande* group as Source1 or Source2. Z-scores adjusted for multiple tests are depicted.

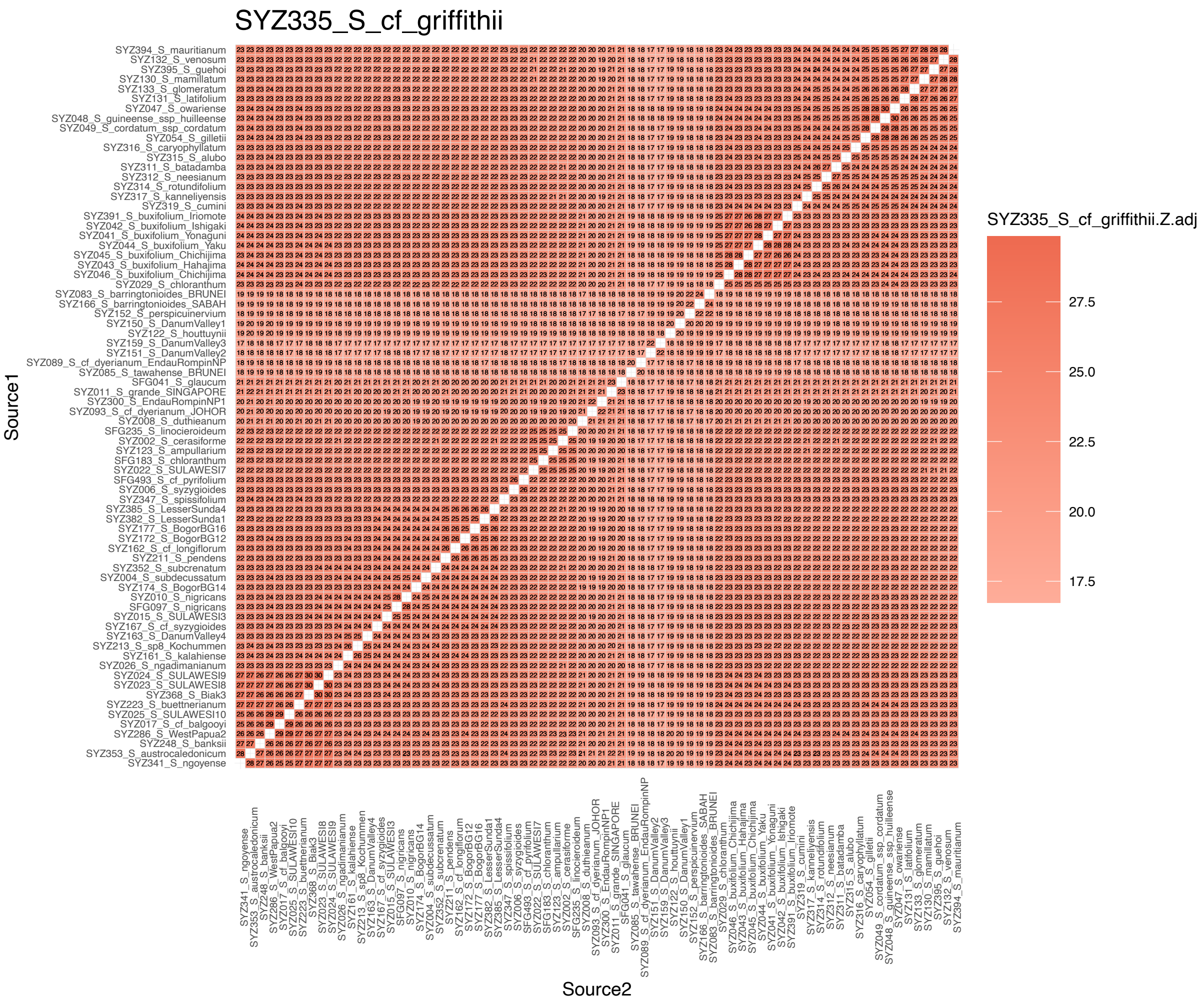

Supplementary Figure 77. F3 heatmap for SYZ335_S_cf_griffithii as target, with other members of the *Syzygium grande* group as Source1 or Source2. Z-scores adjusted for multiple tests are depicted.

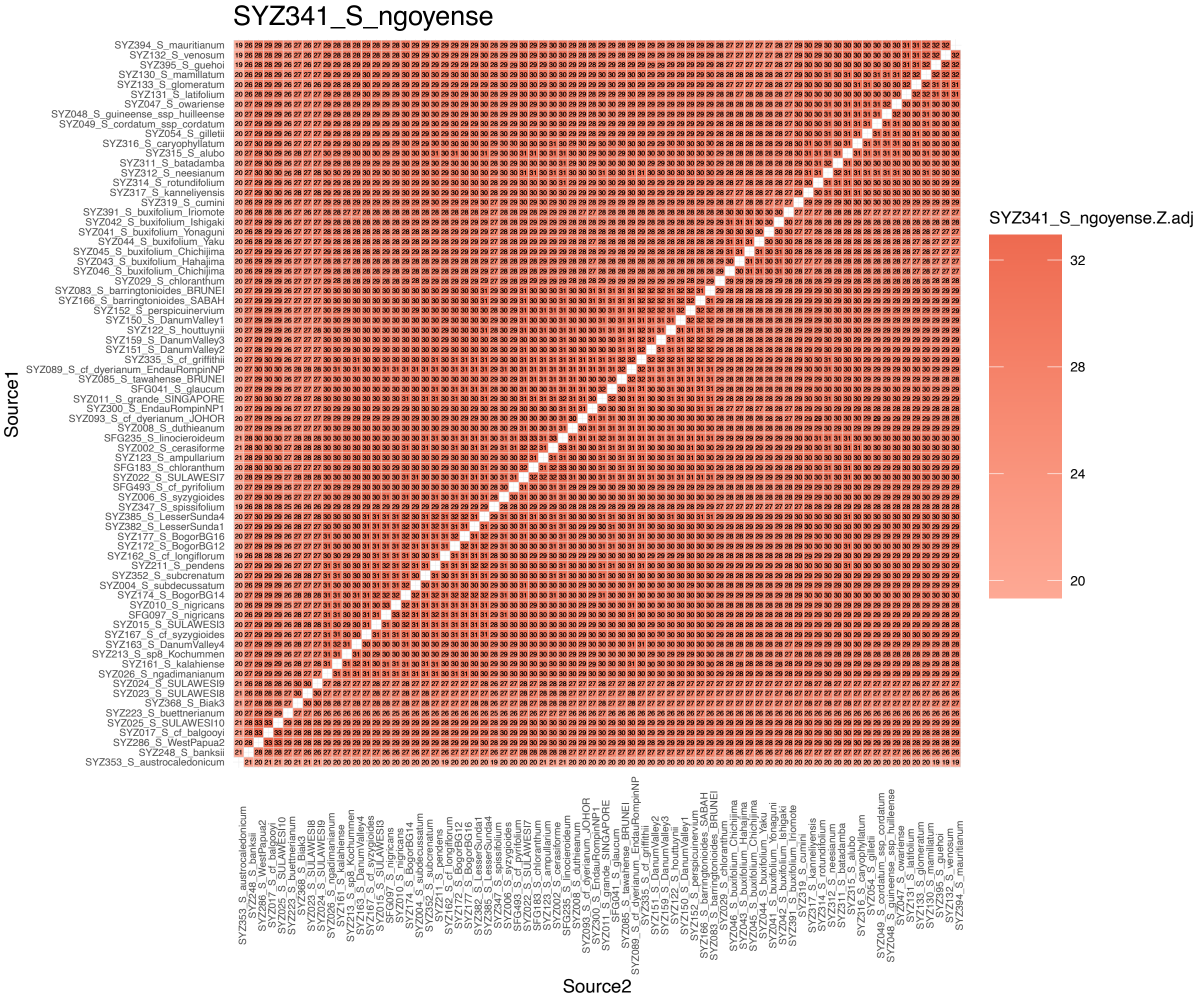

Supplementary Figure 78. F3 heatmap for SYZ341_S_ngoyense as target, with other members of the *Syzygium grande* group as Source1 or Source2. Z-scores adjusted for multiple tests are depicted.

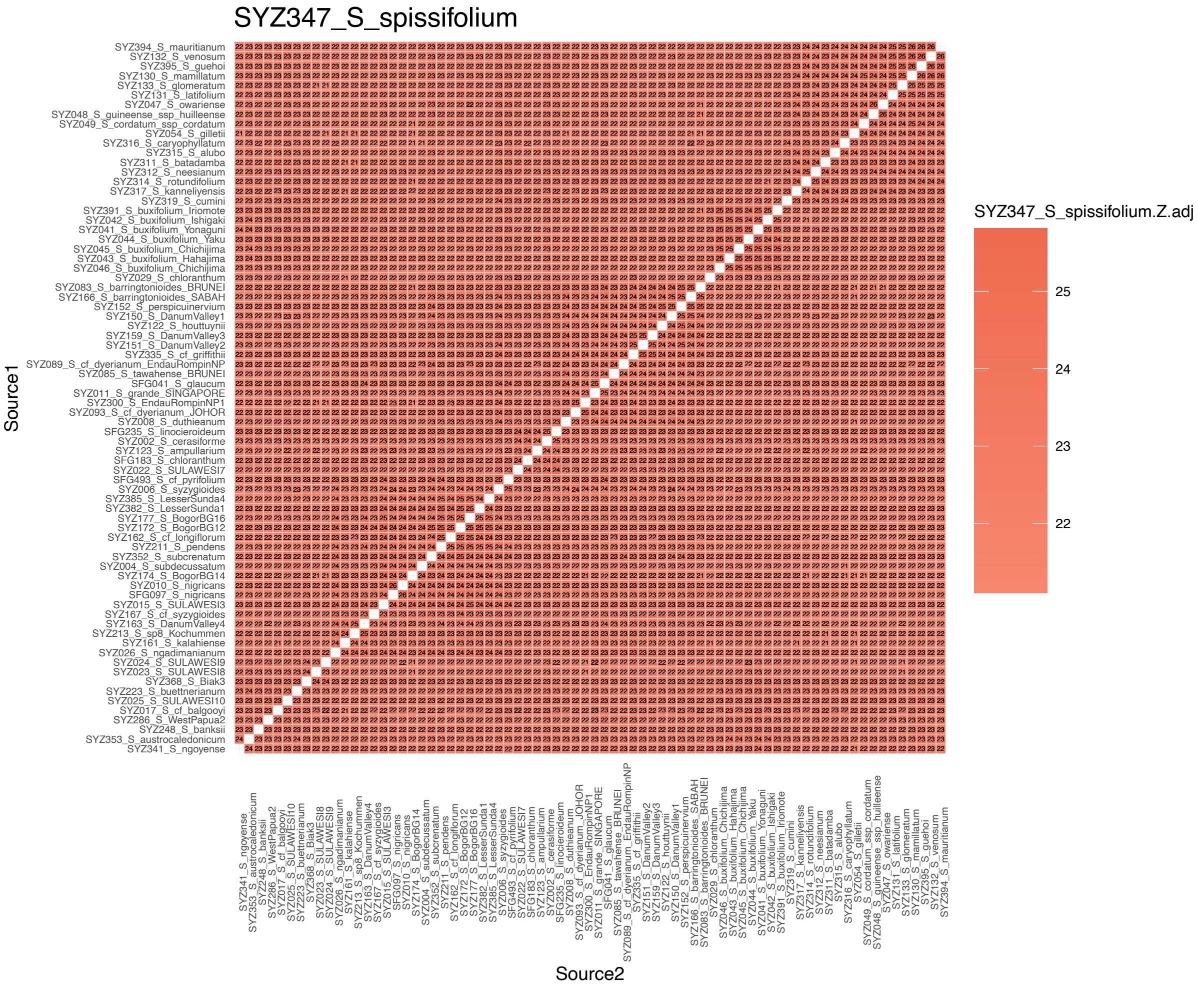

Supplementary Figure 79. F3 heatmap for SYZ341_S_ngoyense as target, with other members of the *Syzygium grande* group as Source1 or Source2. Z-scores adjusted for multiple tests are depicted.

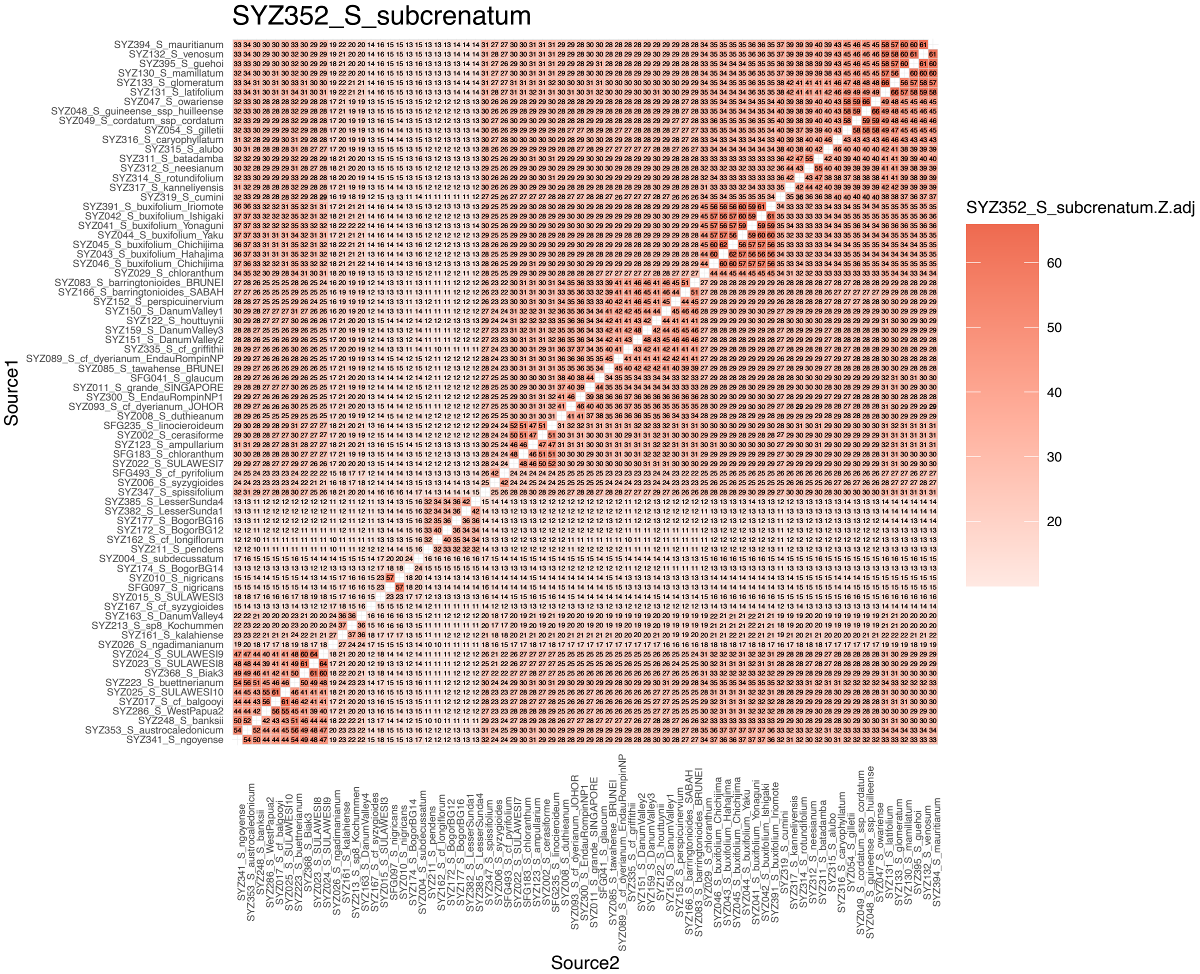

Supplementary Figure 80. F3 heatmap for SYZ352_S_subcrenatum as target, with other members of the *Syzygium grande* group as Source1 or Source2. *Z*-scores adjusted for multiple tests are depicted.

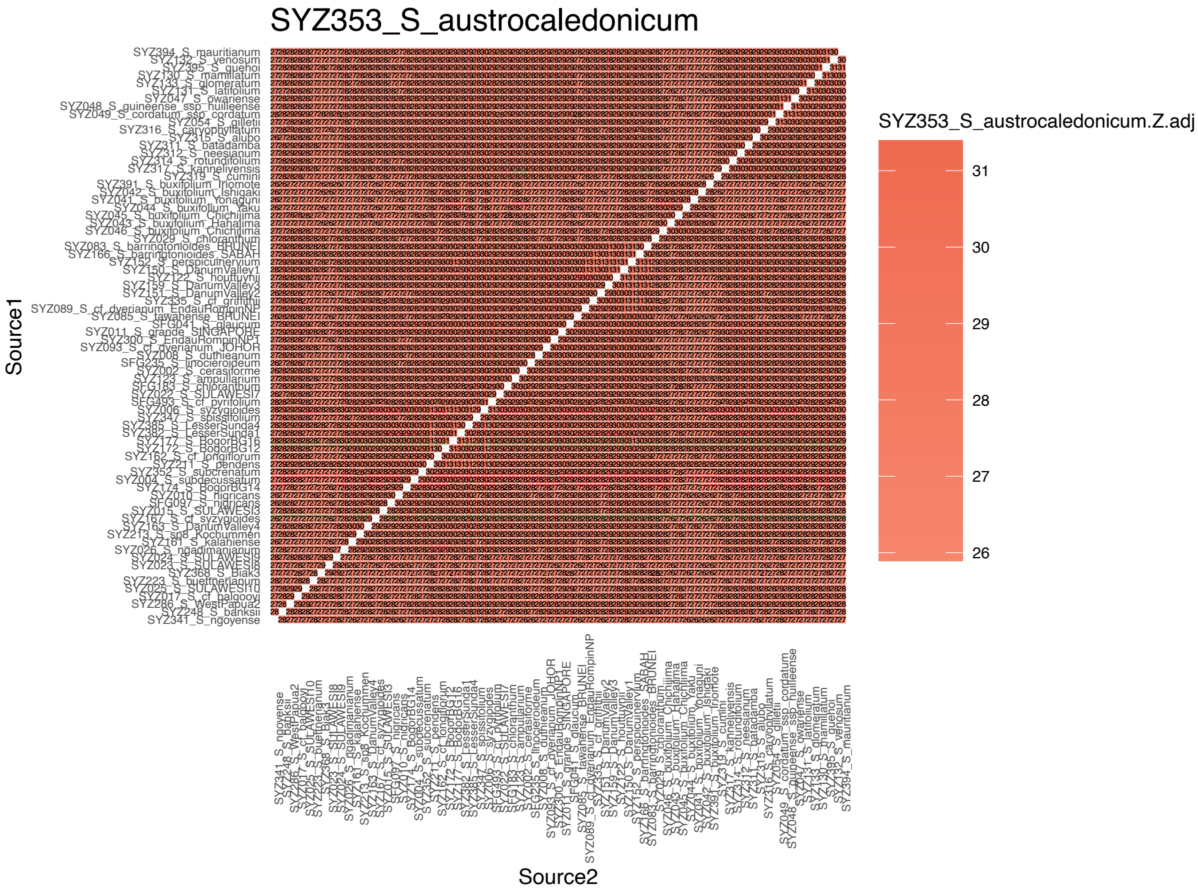

Supplementary Figure 81. F3 heatmap for SYZ353_S_austrocaledonicum as target, with other members of the *Syzygium grande* group as Source1 or Source2. Z-scores adjusted for multiple tests are depicted.

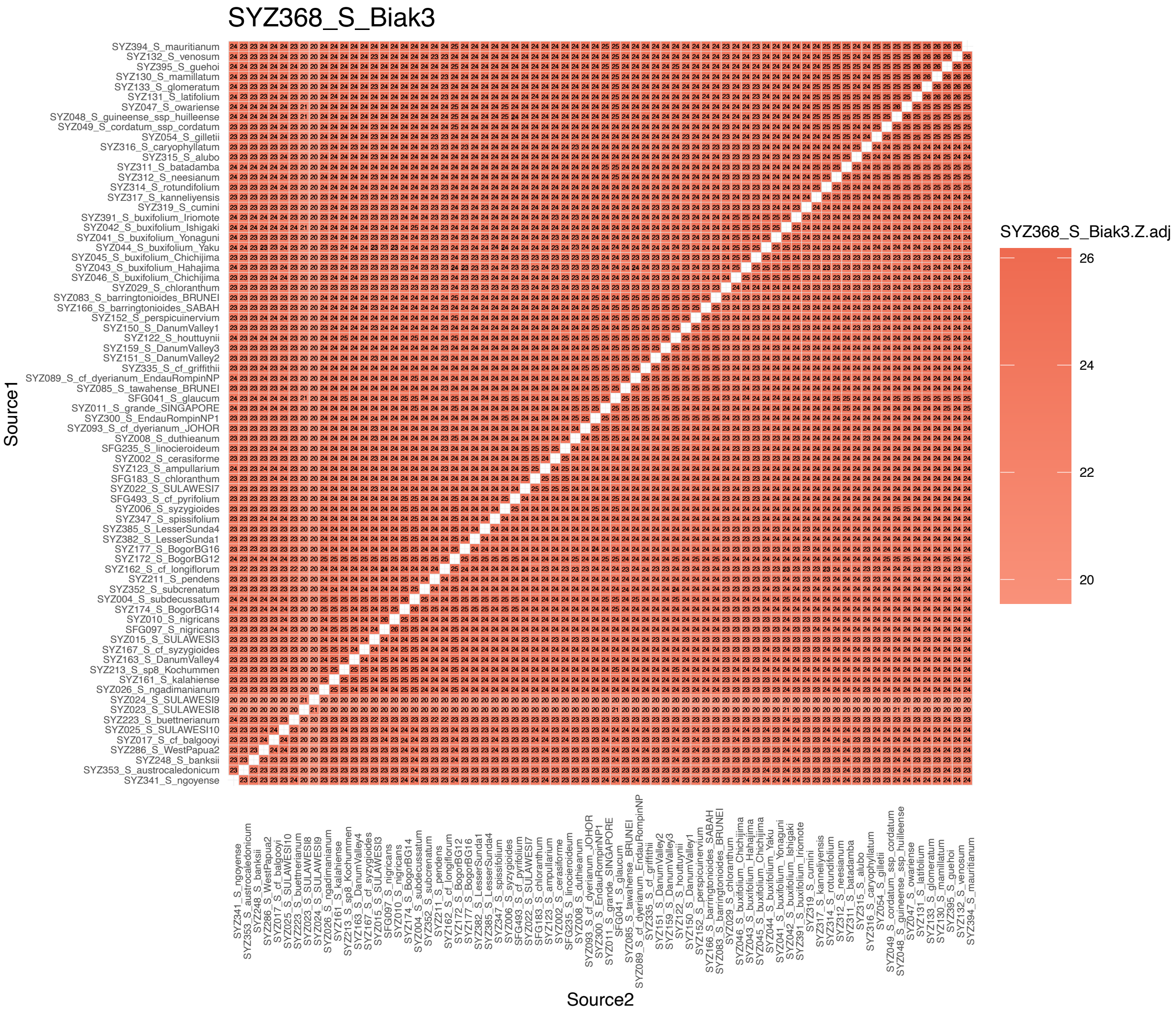

Supplementary Figure 82. F3 heatmap for SYZ368_S_Biak3 as target, with other members of the *Syzygium grande* group as Source1 or Source2. Z-scores adjusted for multiple tests are depicted.

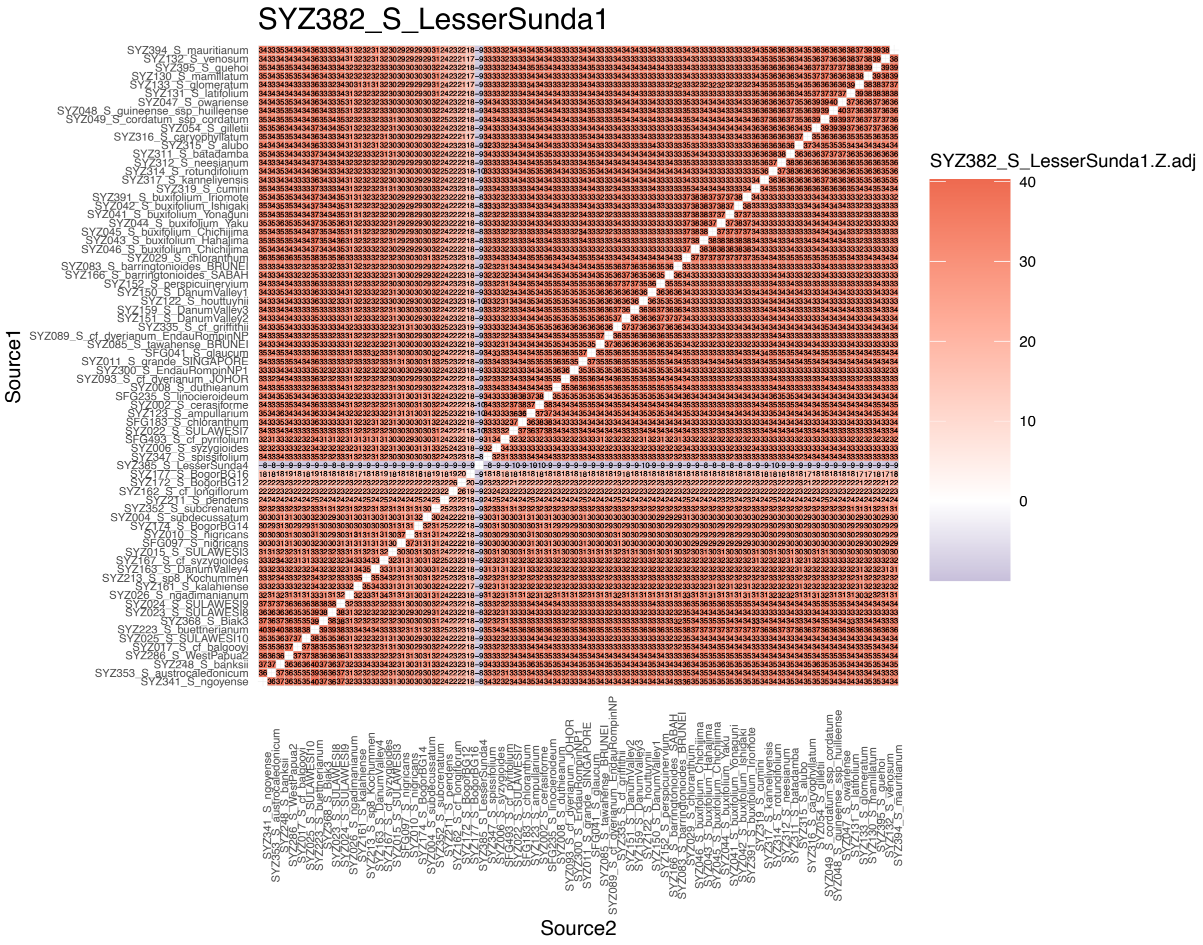

Supplementary Figure 83. F3 heatmap for SYZ382_S_LesserSunda1 as target, with other members of the *Syzygium grande* group as Source1 or Source2. *Z*-scores adjusted for multiple tests are depicted.

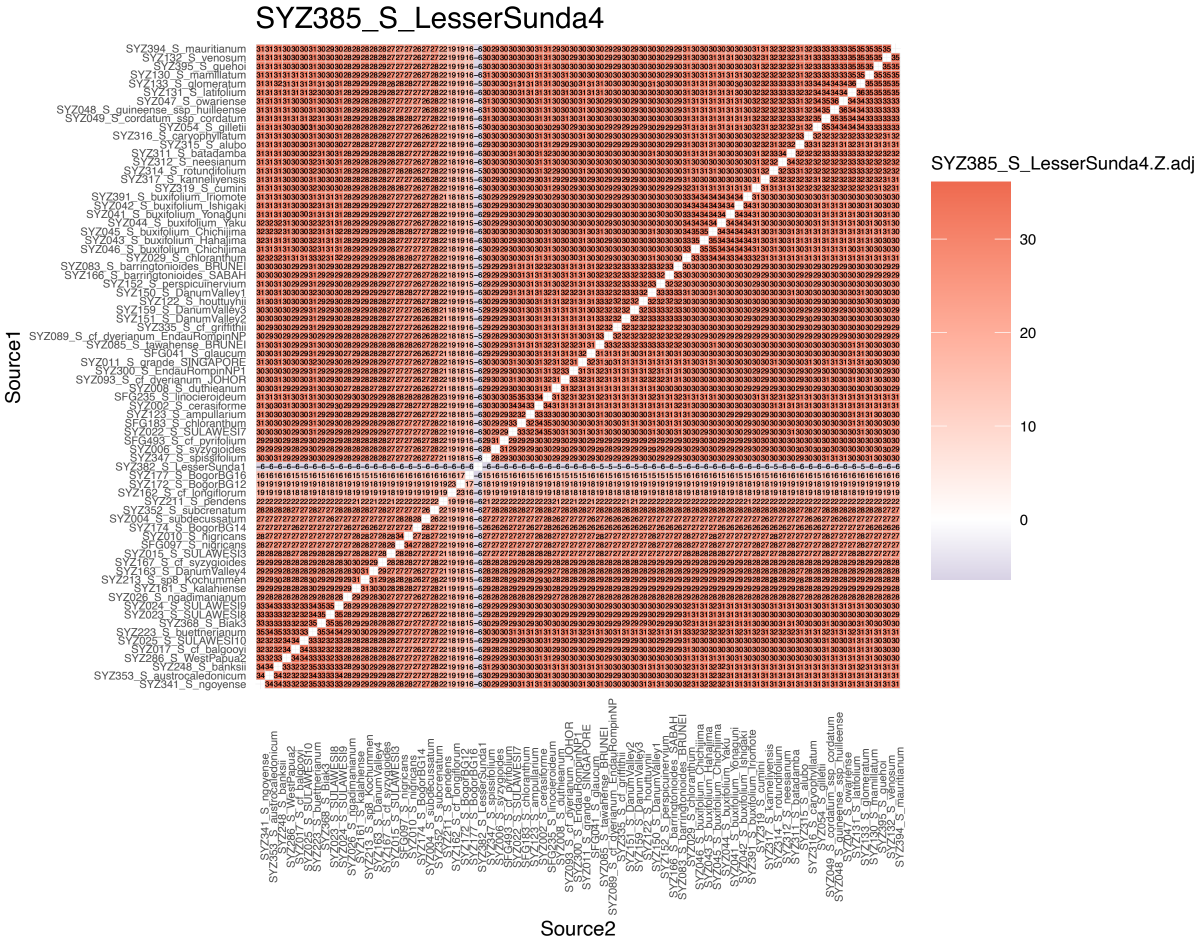

Supplementary Figure 84. F3 heatmap for SYZ385_S_LesserSunda4 as target, with other members of the *Syzygium grande* group as Source1 or Source2. *Z*-scores adjusted for multiple tests are depicted.

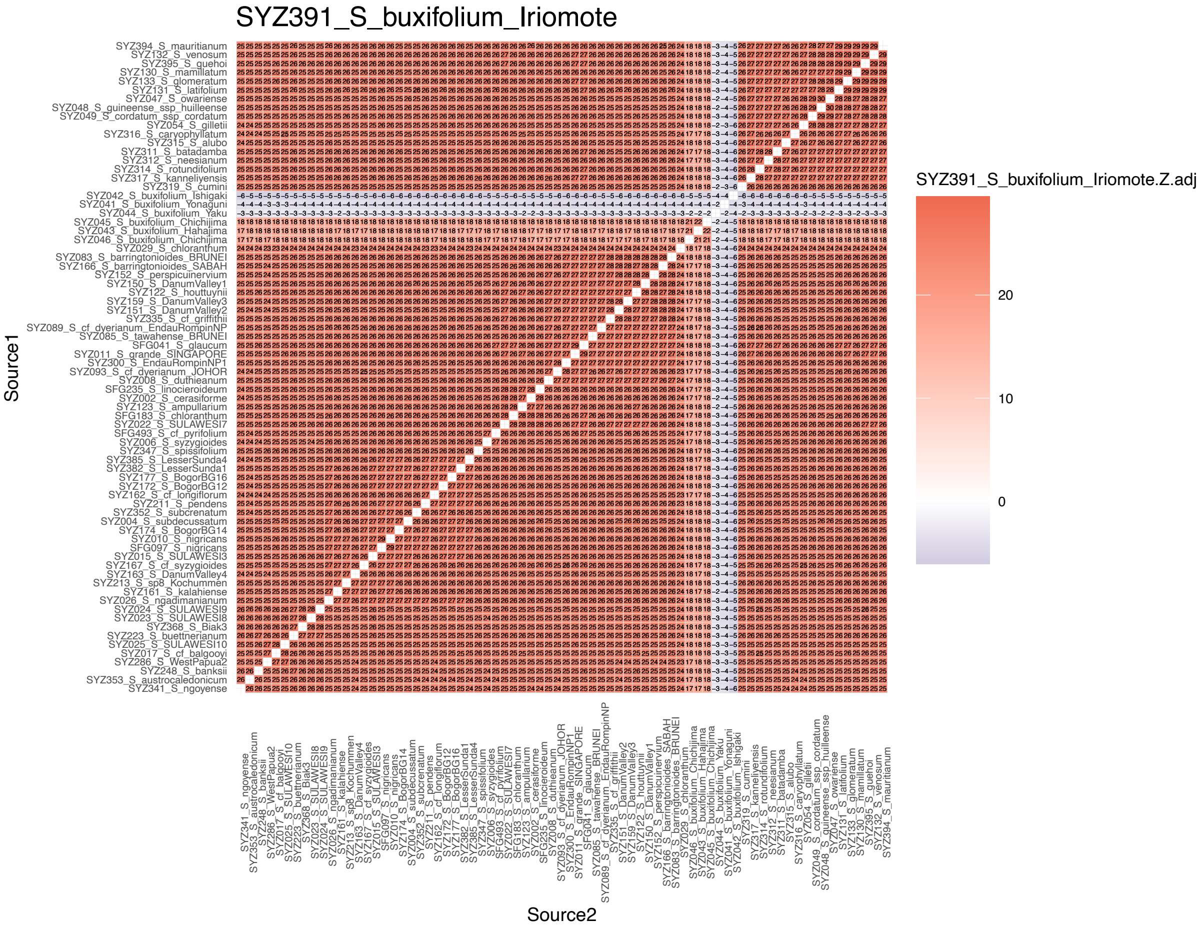

Supplementary Figure 85. F3 heatmap for SYZ391_S_buxifolium_Iriomote as target, with other members of the *Syzygium grande* group as Source1 or Source2. Z-scores adjusted for multiple tests are depicted.

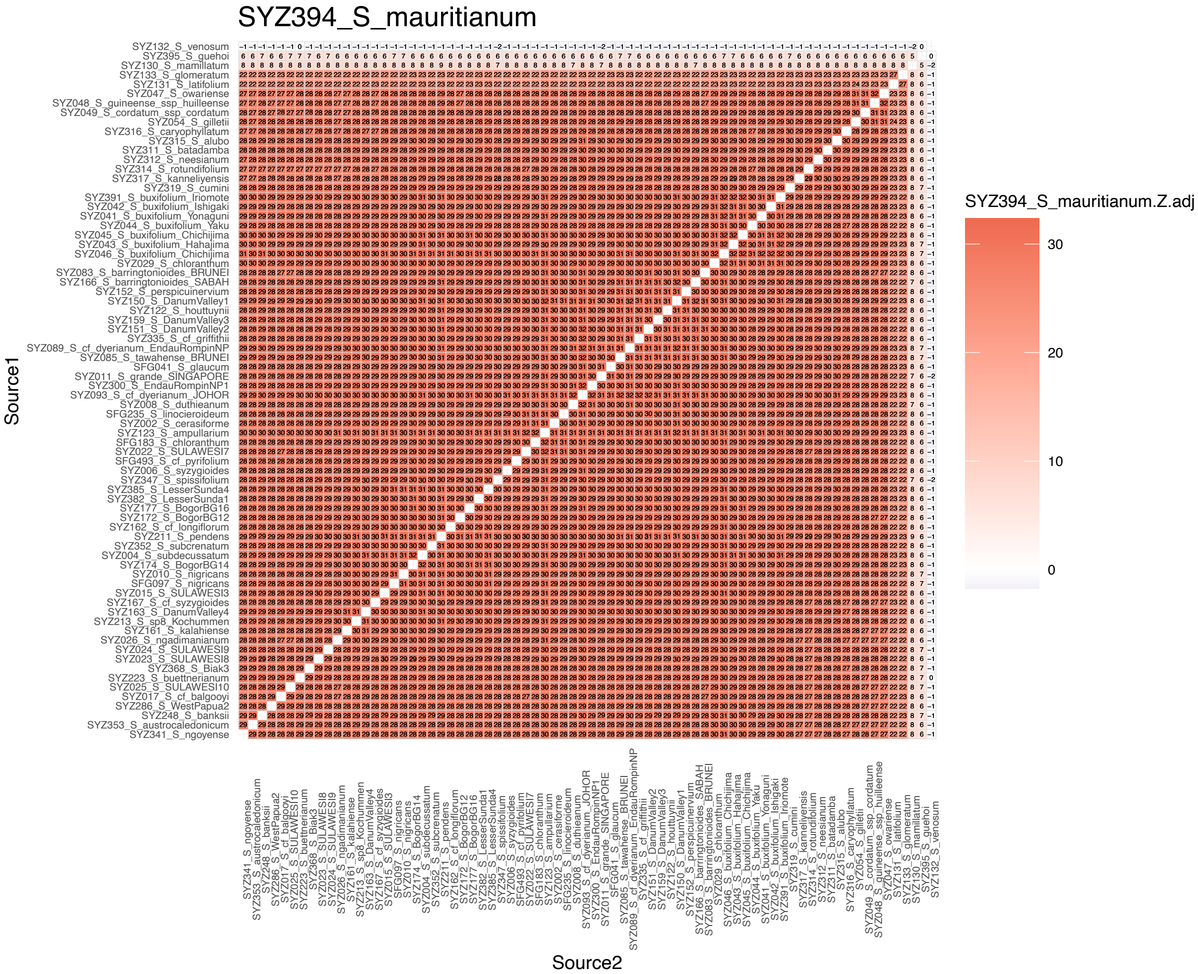

Supplementary Figure 86. F3 heatmap for SYZ394_S_mauritianum as target, with other members of the *Syzygium grande* group as Source1 or Source2. *Z*-scores adjusted for multiple tests are depicted.

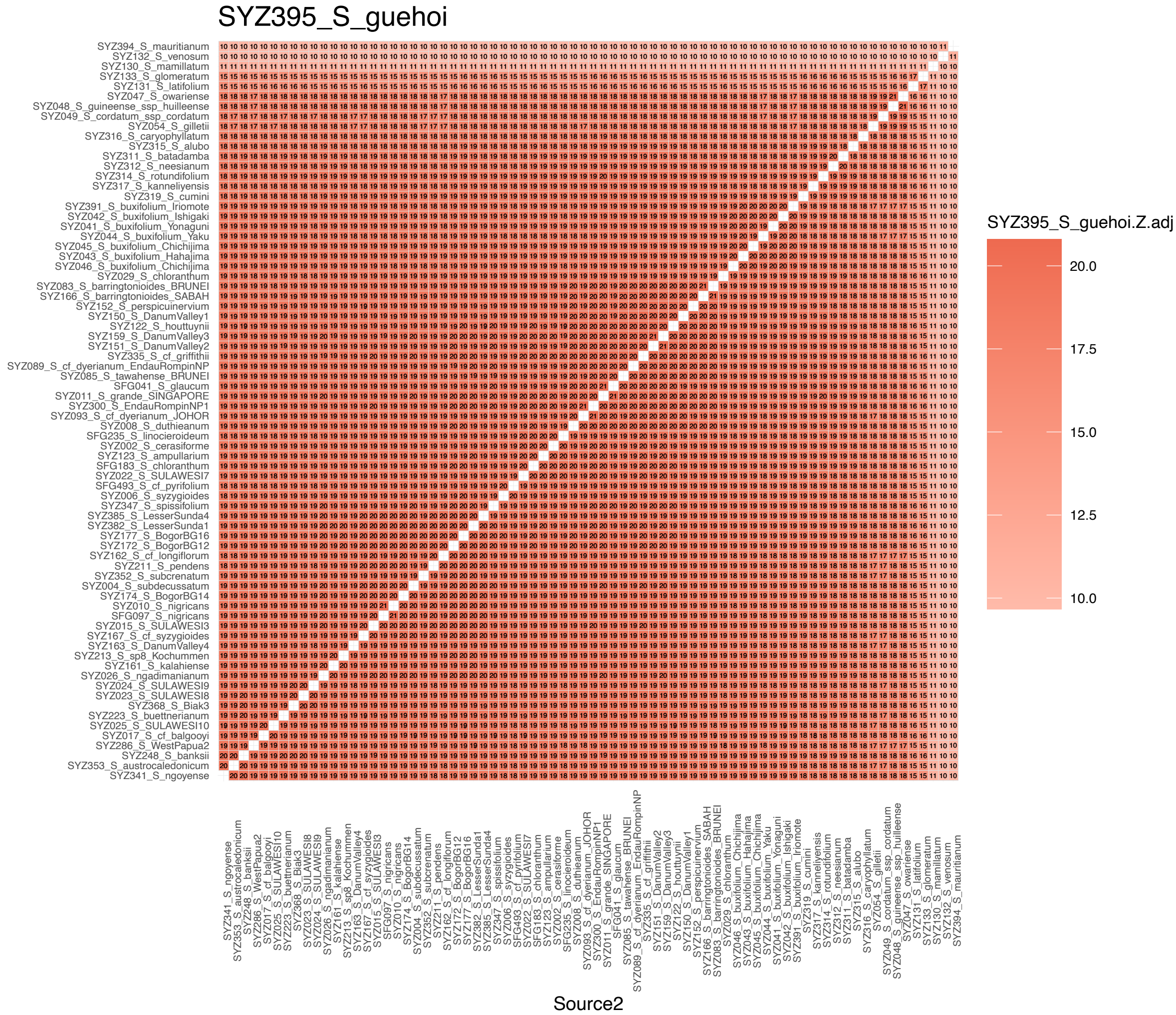

Supplementary Figure 87. F3 heatmap for SYZ395_S_guehoi as target, with other members of the *Syzygium grande* group as Source1 or Source2. Z-scores adjusted for multiple tests are depicted.

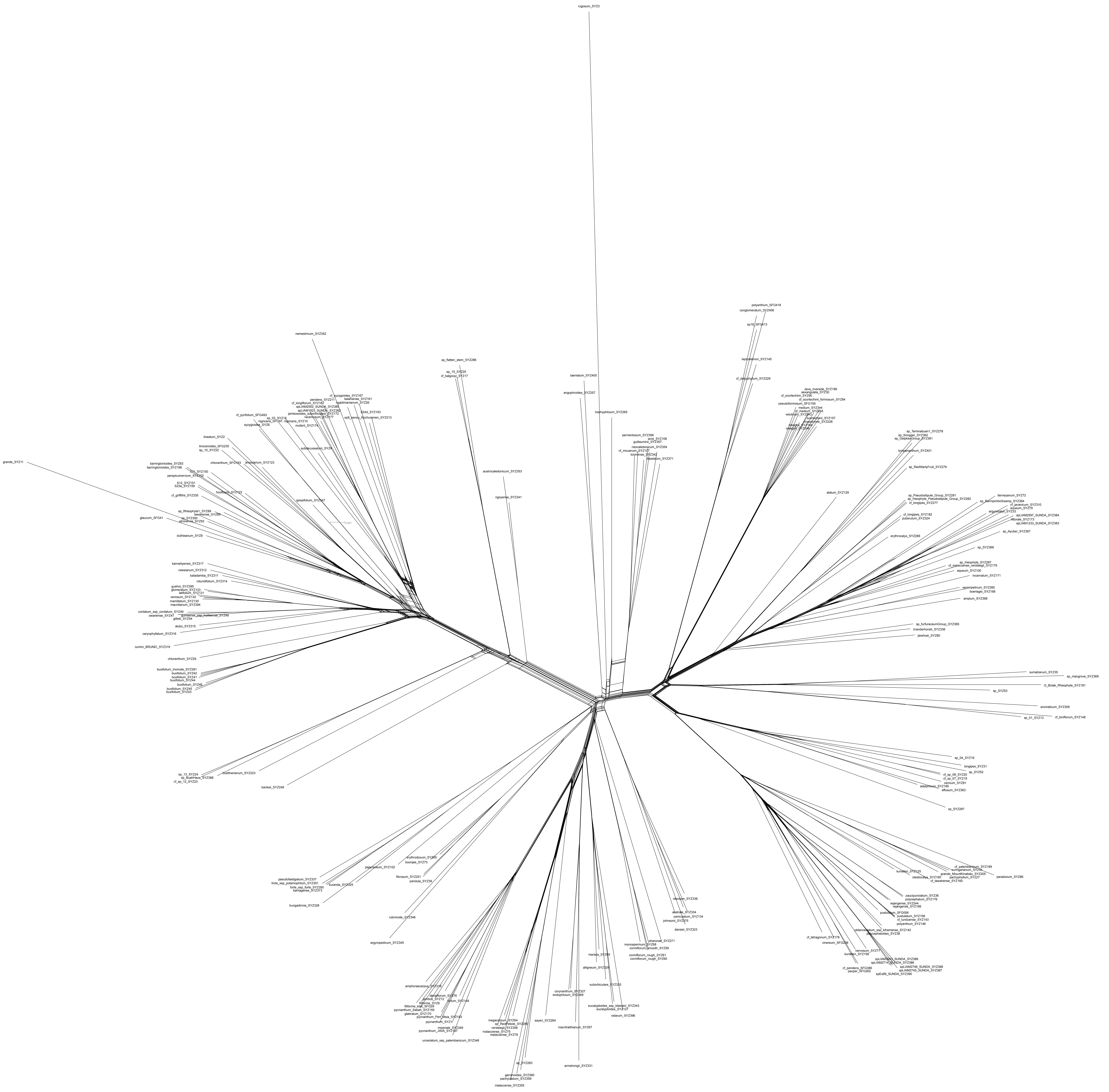

Supplementary Figure 88. NeighborNet network of *Syzygium* subg. *Syzygium*. *Syzygium rugosum* is included as outgroup.

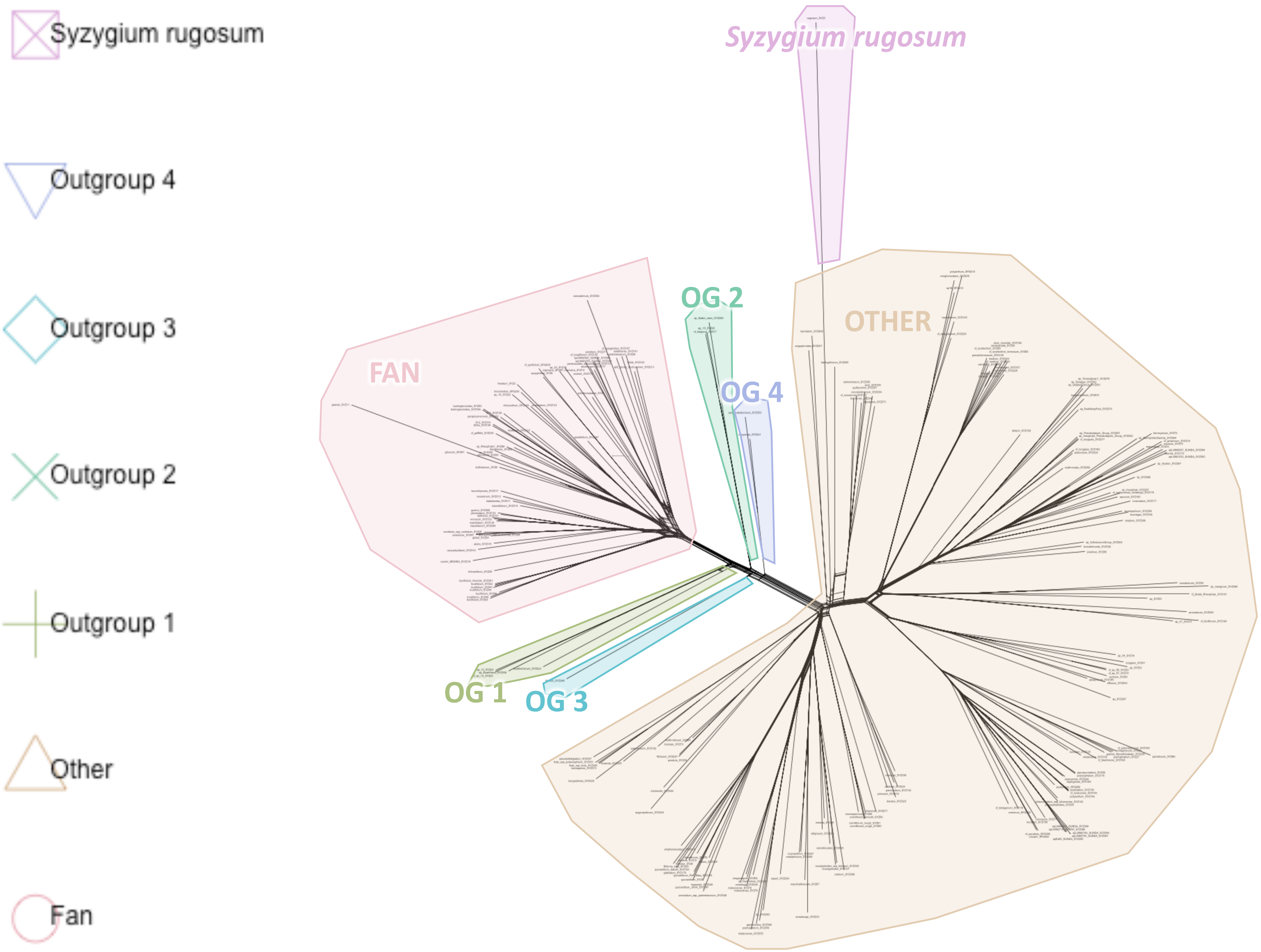

Supplementary Figure 89. Local PCA analyses. NeighborNet network, colour-coded by group of *Syzygium* subg. *Syzygium*. The NeighborNet "fan" (the *S. grande* group) and outgroups in *Syzygium* subg. *Syzygium* are shown, as is *S. rugosum*.

lcl_Scaffold_1

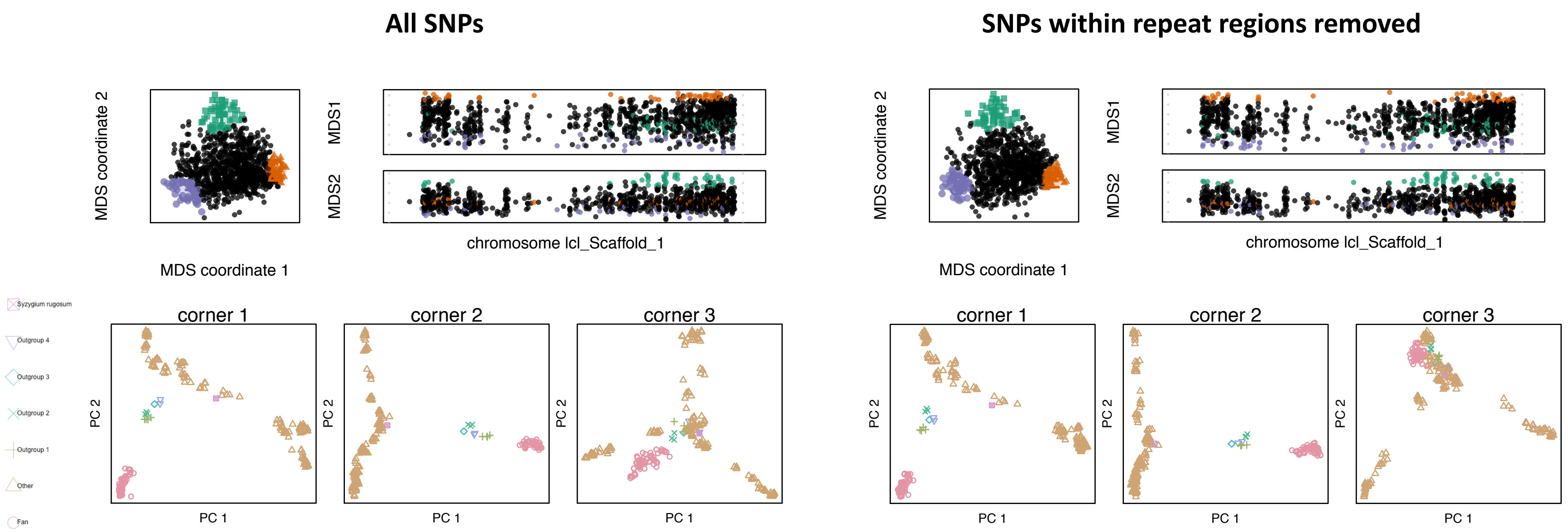

Supplementary Figure 90. Local PCA analysis for Scaffold 1 of *Syzygium grande*. Shown are the MDS plot of SNP PCA windows with colourations by corner; the windows mapped onto the scaffold and coloured by corner; and the taxa (coloured as in Supplementary Fig. 89) projected onto PC1/PC2 for each corner. Left panels are for all SNPs; right are for SNP data with repeat regions removed.

lcl_Scaffold_2

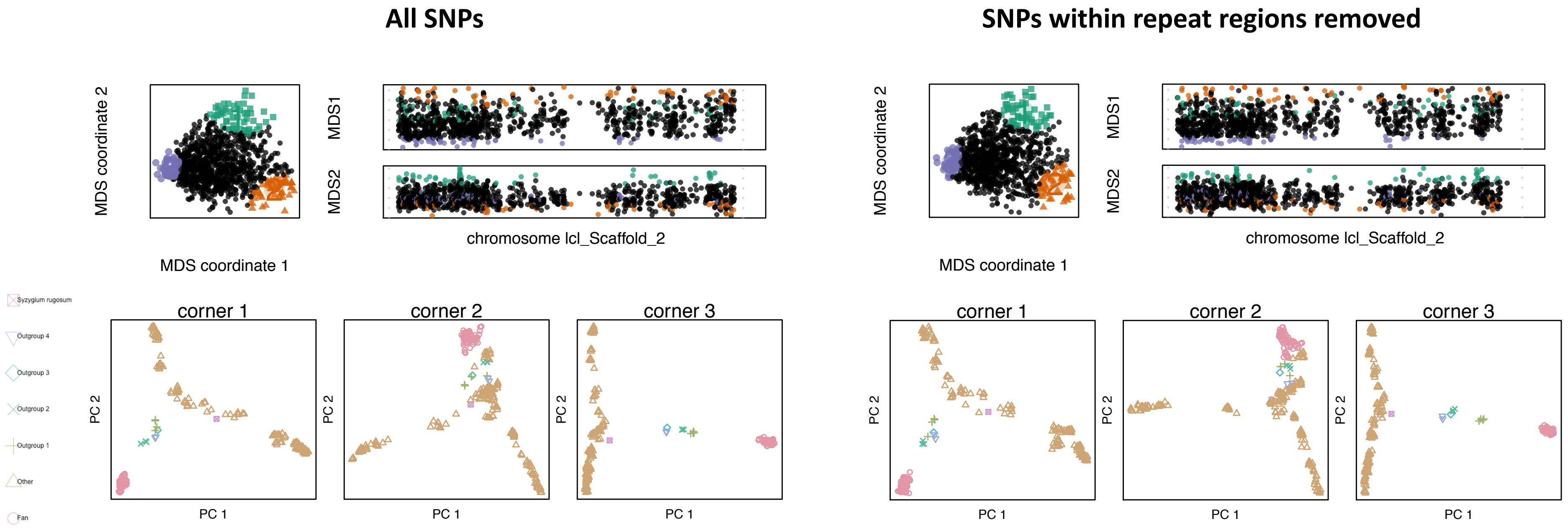

Supplementary Figure 91. Local PCA analysis for Scaffold 2 of *Syzygium grande*. Shown are the MDS plot of SNP PCA windows with colourations by corner; the windows mapped onto the scaffold and coloured by corner; and the taxa (coloured as in Supplementary Fig. 89) projected onto PC1/PC2 for each corner. Left panels are for all SNPs; right are for SNP data with repeat regions removed.

lcl_Scaffold_3

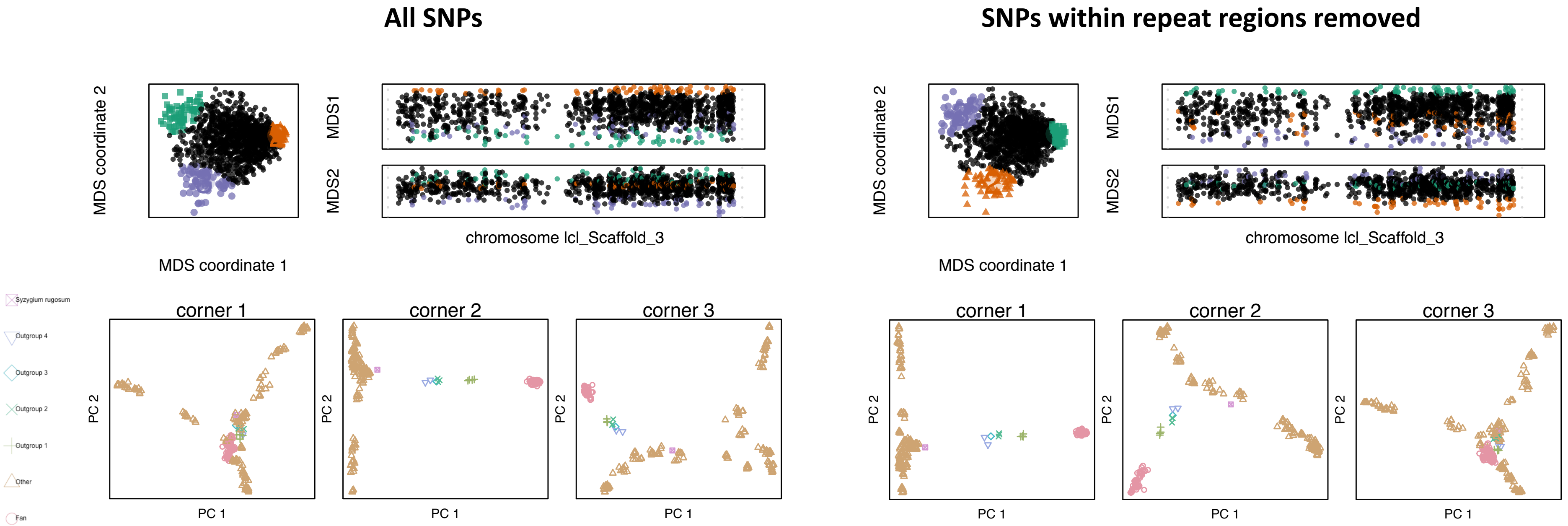

Supplementary Figure 92. Local PCA analysis for Scaffold 3 of *Syzygium grande*. Shown are the MDS plot of SNP PCA windows with colourations by corner; the windows mapped onto the scaffold and coloured by corner; and the taxa (coloured as in Supplementary Fig. 89) projected onto PC1/PC2 for each corner. Left panels are for all SNPs; right are for SNP data with repeat regions removed.

lcl_Scaffold_4

All SNPs **SNPs within repeat regions removed**

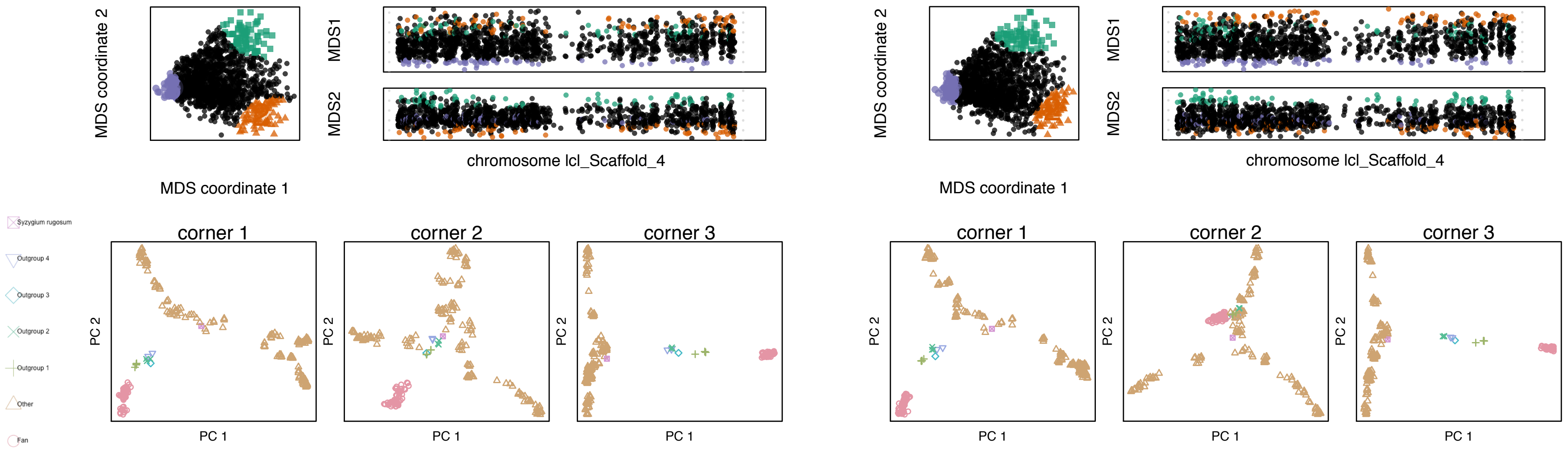

Supplementary Figure 93. Local PCA analysis for Scaffold 4 of *Syzygium grande*. Shown are the MDS plot of SNP PCA windows with colourations by corner; the windows mapped onto the scaffold and coloured by corner; and the taxa (coloured as in Supplementary Fig. 89) projected onto PC1/PC2 for each corner. Left panels are for all SNPs; right are for SNP data with repeat regions removed.

lcl_Scaffold_5

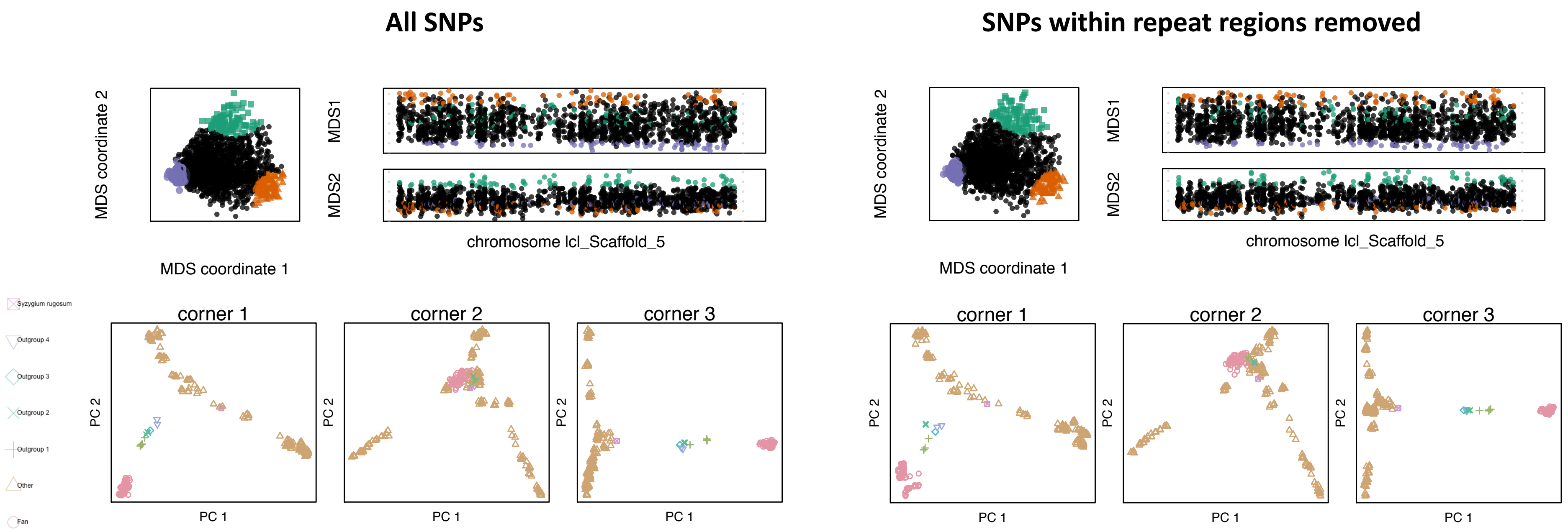

Supplementary Figure 94. Local PCA analysis for Scaffold 5 of *Syzygium grande*. Shown are the MDS plot of SNP PCA windows with colourations by corner; the windows mapped onto the scaffold and coloured by corner; and the taxa (coloured as in Supplementary Fig. 89) projected onto PC1/PC2 for each corner. Left panels are for all SNPs; right are for SNP data with repeat regions removed.

lcl_Scaffold_6

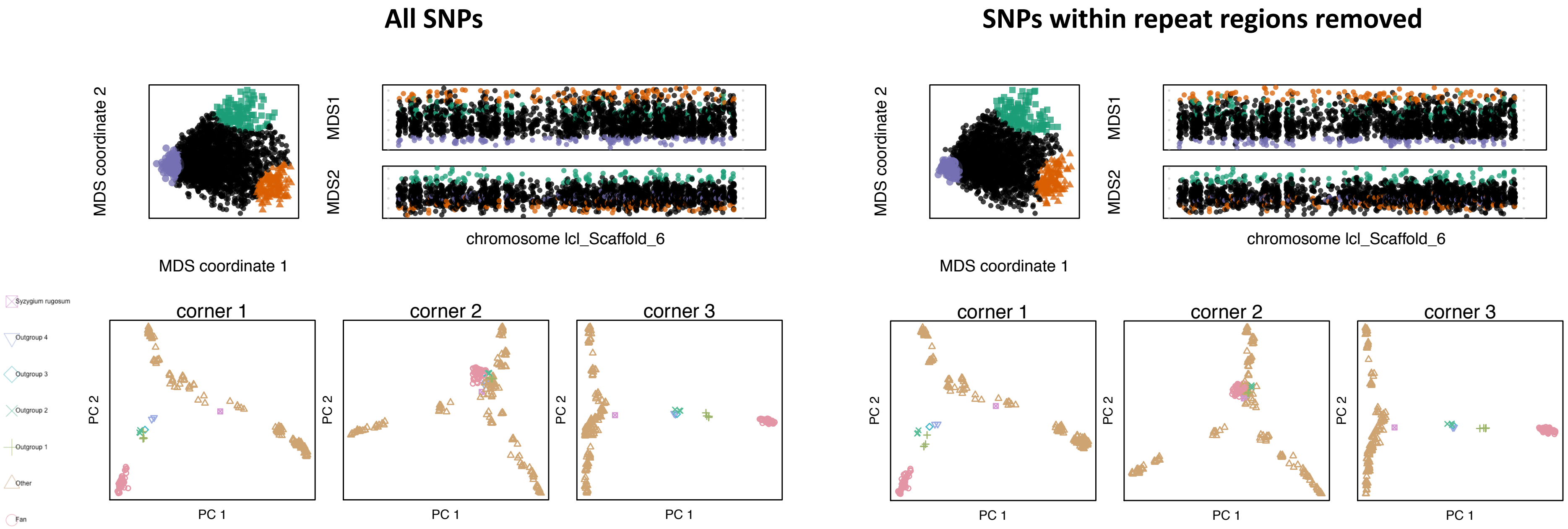

Supplementary Figure 95. Local PCA analysis for Scaffold 6 of *Syzygium grande*. Shown are the MDS plot of SNP PCA windows with colourations by corner; the windows mapped onto the scaffold and coloured by corner; and the taxa (coloured as in Supplementary Fig. 89) projected onto PC1/PC2 for each corner. Left panels are for all SNPs; right are for SNP data with repeat regions removed.

lcl_Scaffold_7

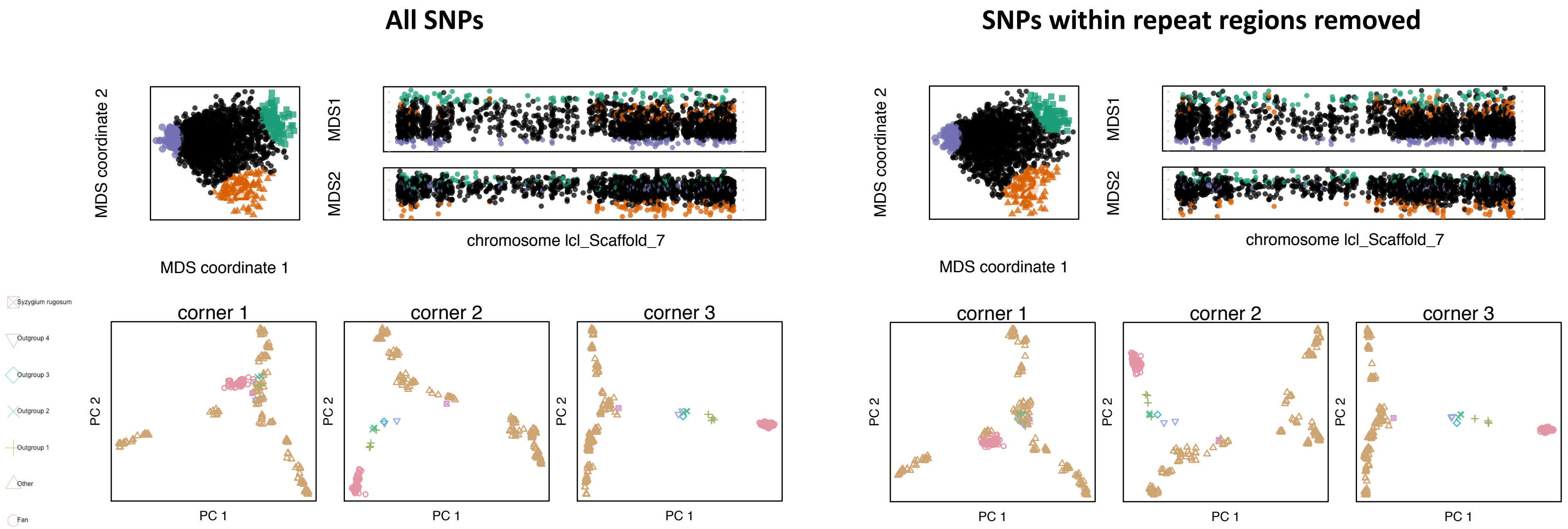

Supplementary Figure 96. Local PCA analysis for Scaffold 7 of *Syzygium grande*. Shown are the MDS plot of SNP PCA windows with colourations by corner; the windows mapped onto the scaffold and coloured by corner; and the taxa (coloured as in Supplementary Fig. 89) projected onto PC1/PC2 for each corner. Left panels are for all SNPs; right are for SNP data with repeat regions removed.

lcl_Scaffold_8

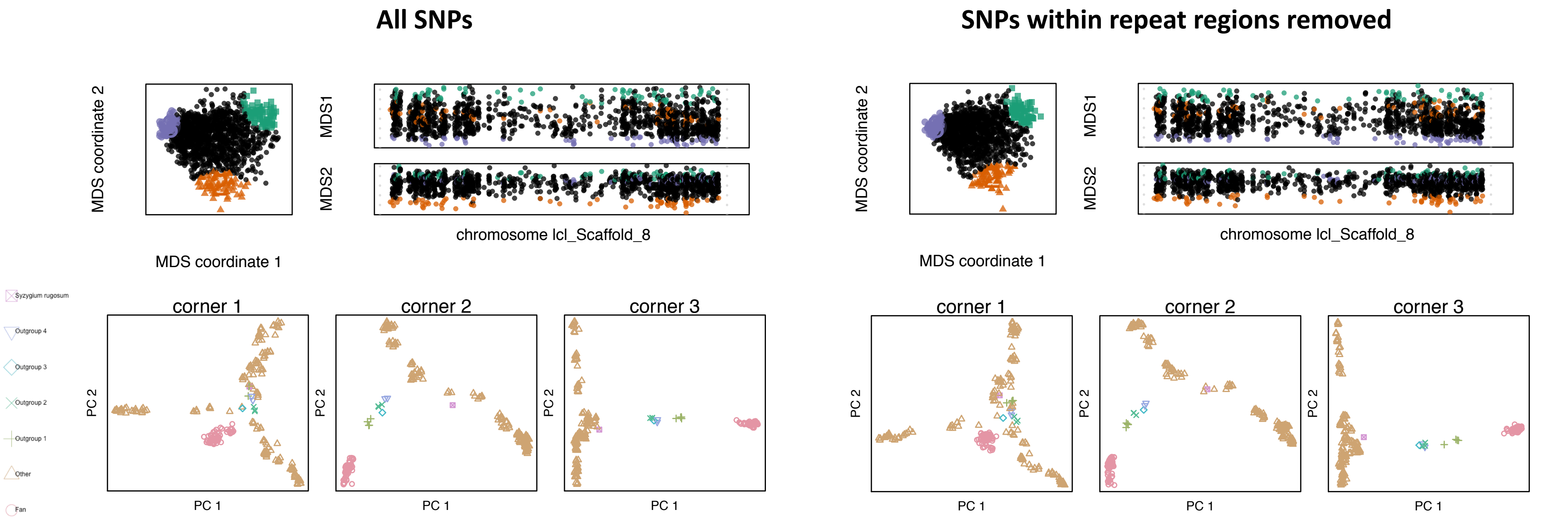

Supplementary Figure 97. Local PCA analysis for Scaffold 8 of *Syzygium grande*. Shown are the MDS plot of SNP PCA windows with colourations by corner; the windows mapped onto the scaffold and coloured by corner; and the taxa (coloured as in Supplementary Fig. 89) projected onto PC1/PC2 for each corner. Left panels are for all SNPs; right are for SNP data with repeat regions removed.

lcl_Scaffold_9

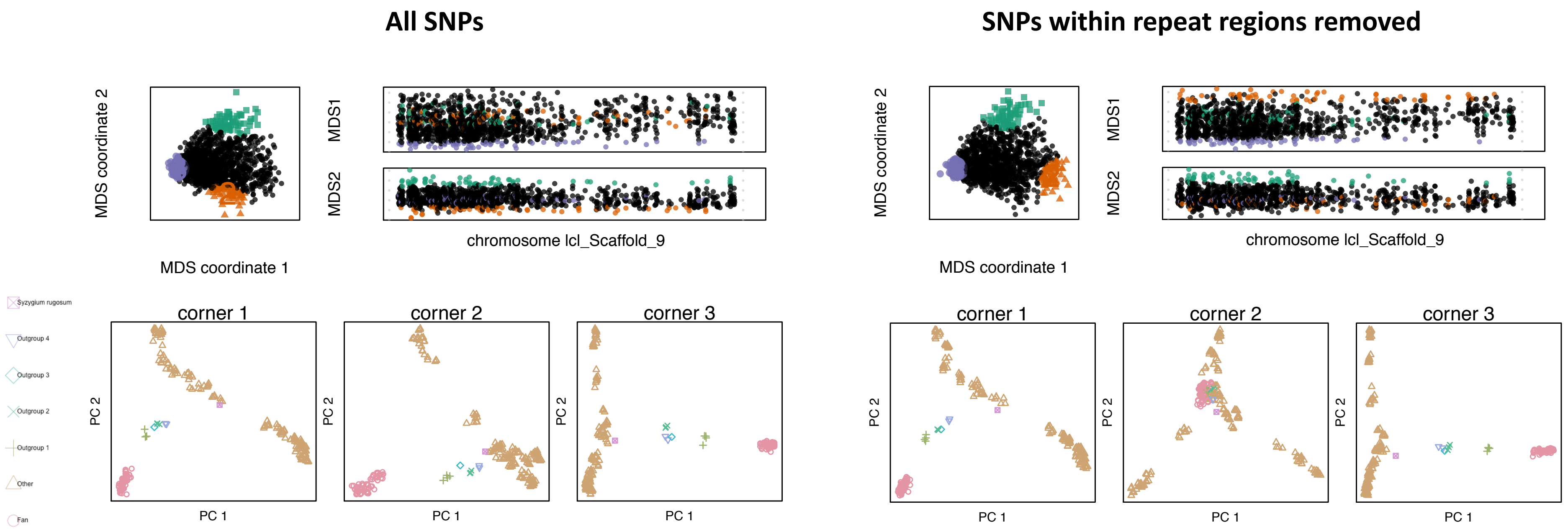

Supplementary Figure 98. Local PCA analysis for Scaffold 9 of *Syzygium grande*. Shown are the MDS plot of SNP PCA windows with colourations by corner; the windows mapped onto the scaffold and coloured by corner; and the taxa (coloured as in Supplementary Fig. 89) projected onto PC1/PC2 for each corner. Left panels are for all SNPs; right are for SNP data with repeat regions removed.

lcl_Scaffold_10

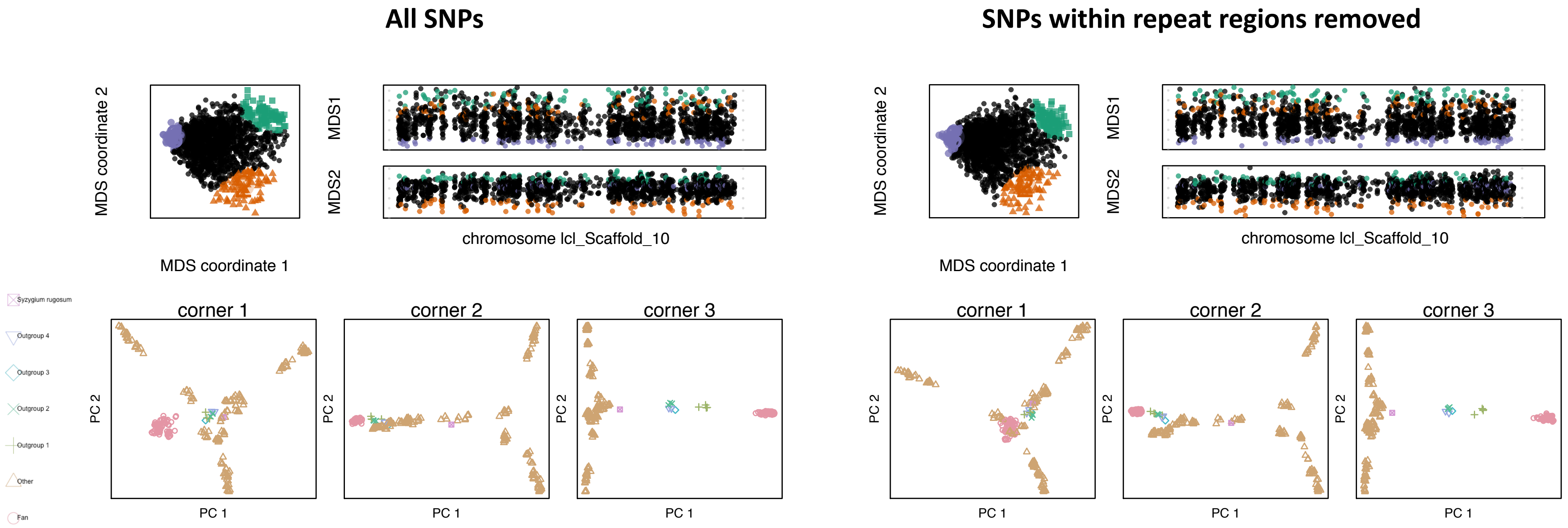

Supplementary Figure 99. Local PCA analysis for Scaffold 10 of *Syzygium grande*. Shown are the MDS plot of SNP PCA windows with colourations by corner; the windows mapped onto the scaffold and coloured by corner; and the taxa (coloured as in Supplementary Fig. 89) projected onto PC1/PC2 for each corner. Left panels are for all SNPs; right are for SNP data with repeat regions removed.

lcl_Scaffold_11

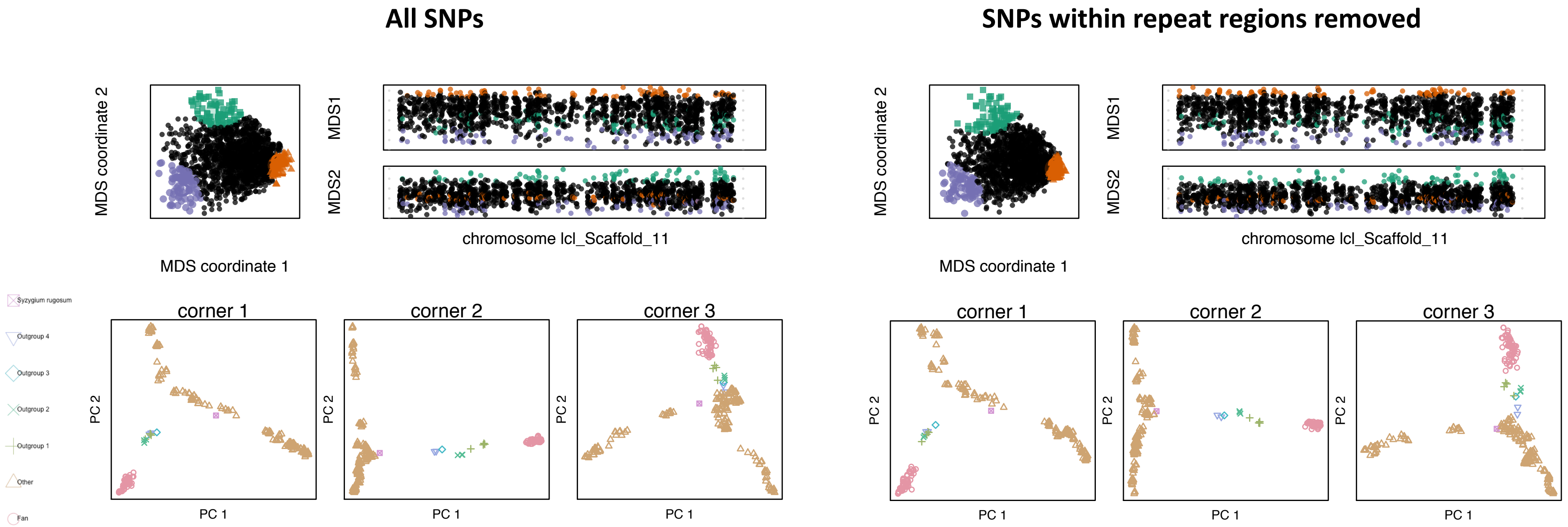

Supplementary Figure 100. Local PCA analysis for Scaffold 11 of *Syzygium grande*. Shown are the MDS plot of SNP PCA windows with colourations by corner; the windows mapped onto the scaffold and coloured by corner; and the taxa (coloured as in Supplementary Fig. 89) projected onto PC1/PC2 for each corner. Left panels are for all SNPs; right are for SNP data with repeat regions removed.

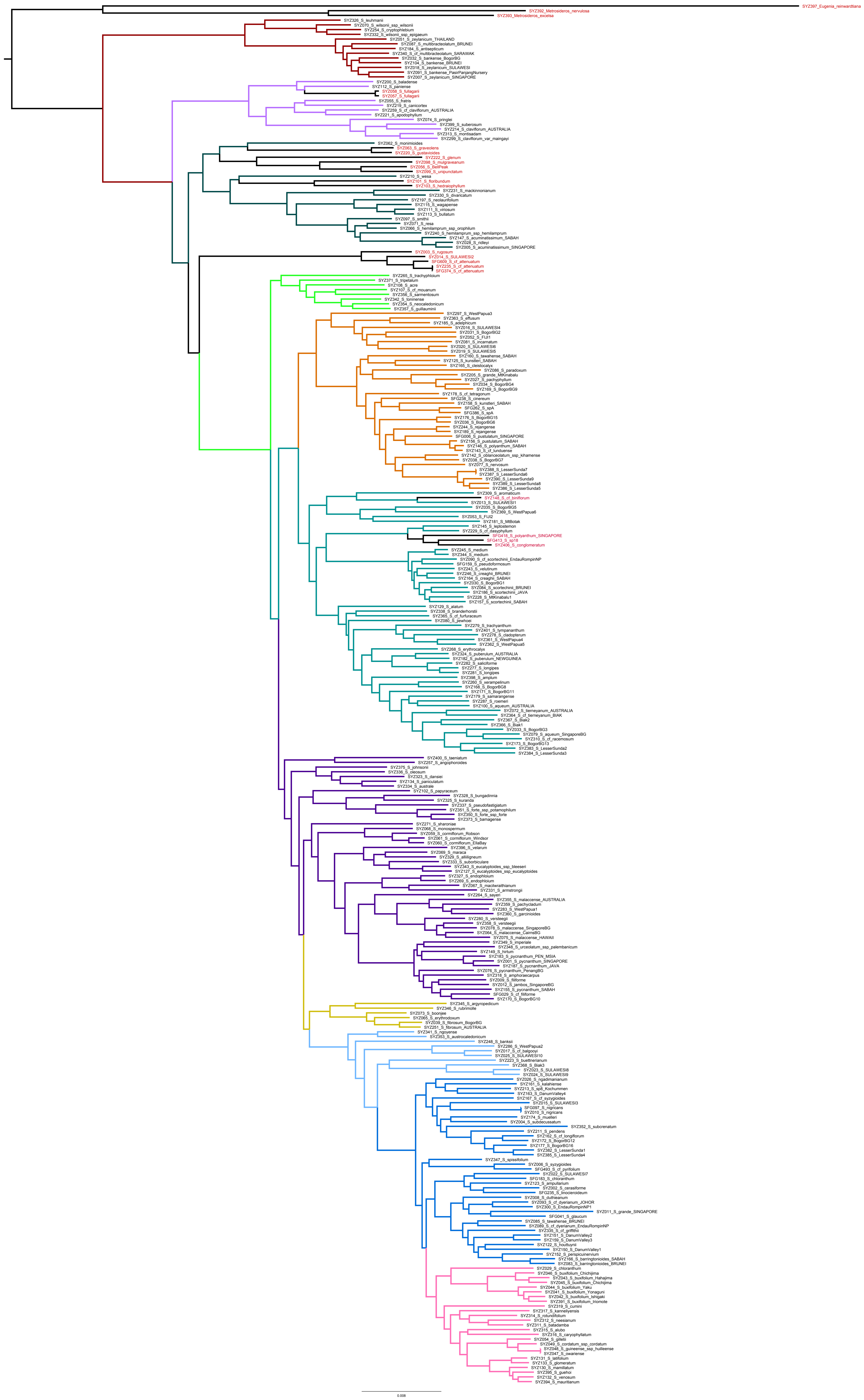

Supplementary Figure 101. PCA analysis of main SNP dataset. BUSCO tree coloured according to taxon labelling used. The taxon names coloured in red indicate those individuals automatically excluded by the smartpca software.

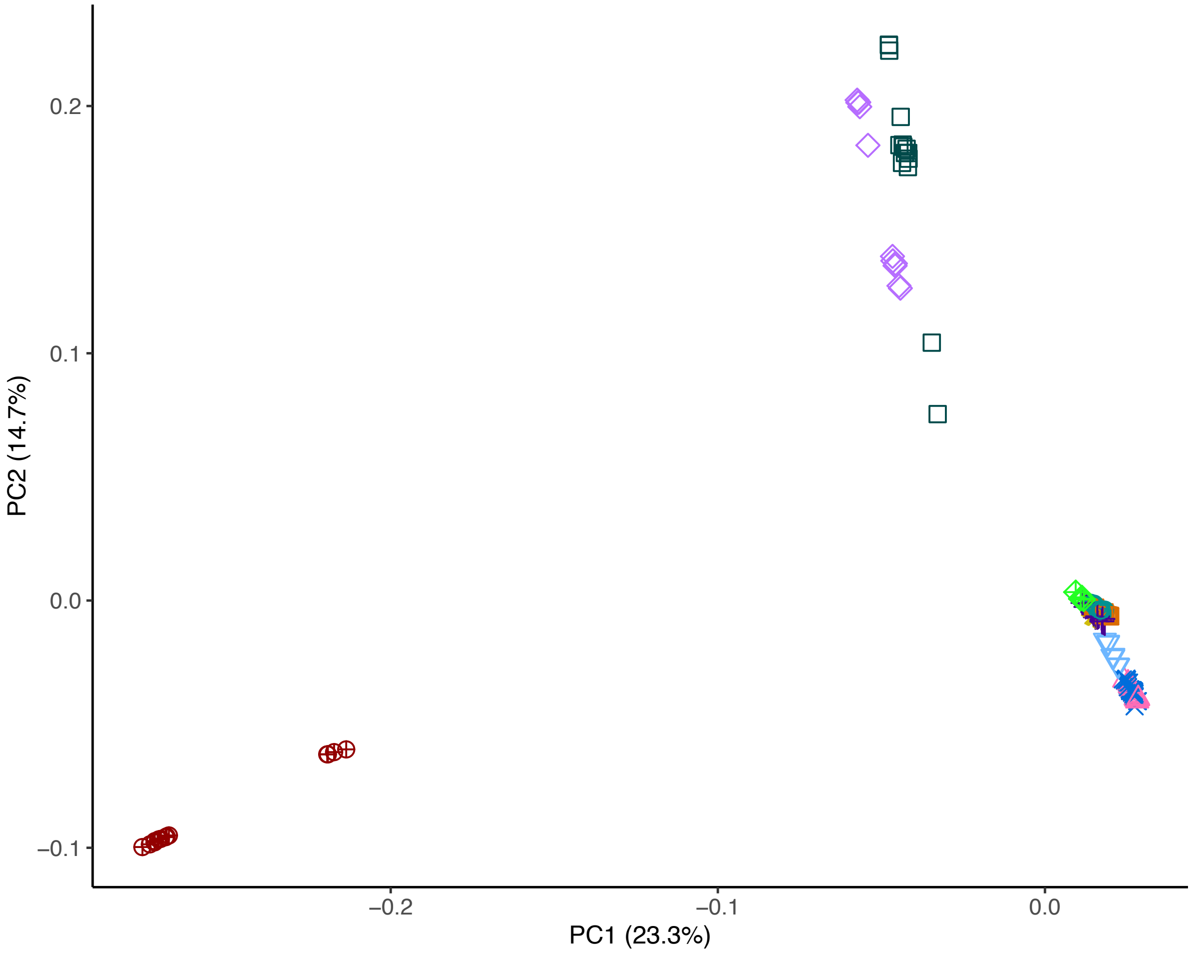

Supplementary Figure 102. PCA analysis of main SNP dataset. A plot of PC1 and PC2 is shown. Colourations follow the BUSCO species tree in Supplementary Fig. 101.

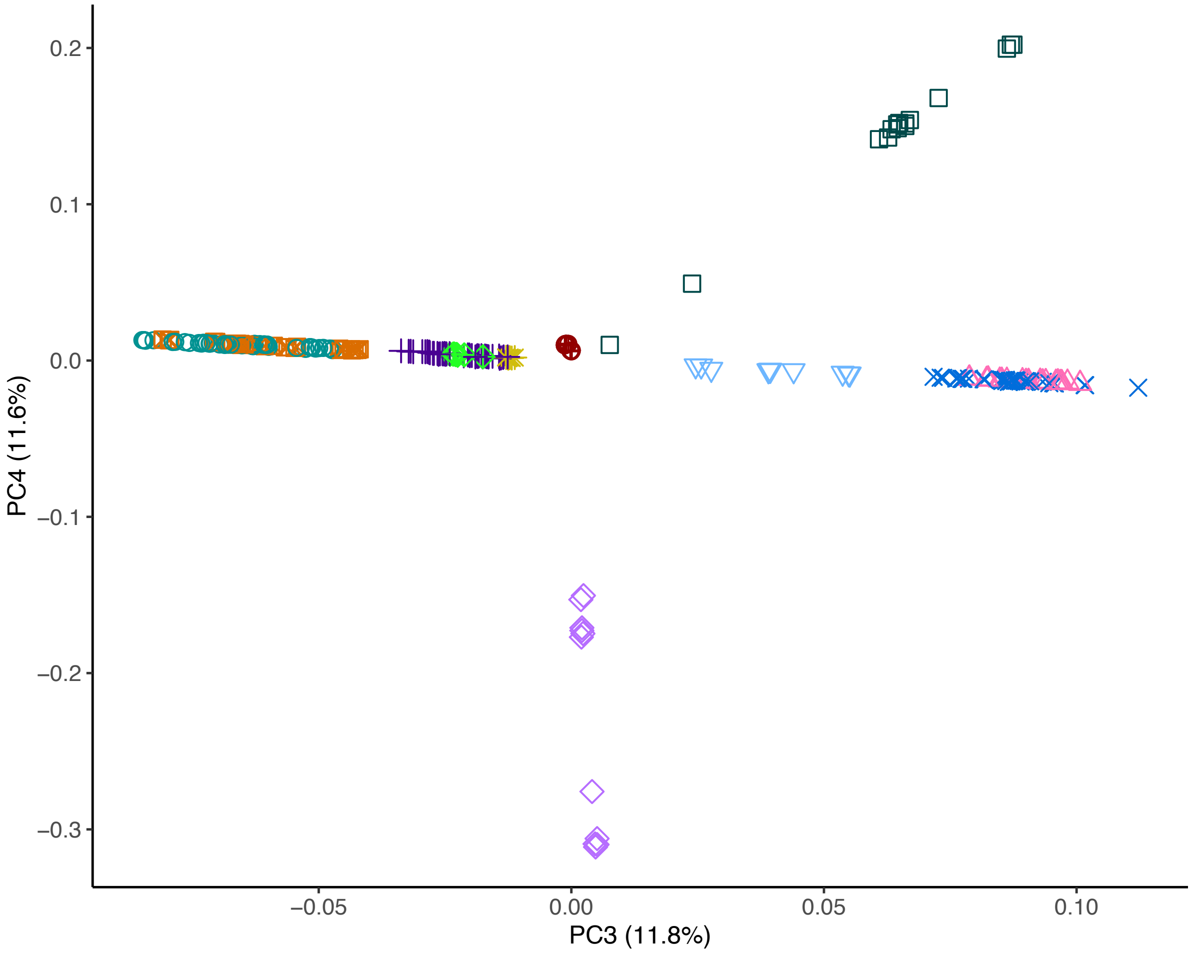

Supplementary Figure 103. PCA analysis of main SNP dataset. A plot of PC3 and PC4 is shown. Colourations follow the BUSCO species tree in Supplementary Fig. 101.

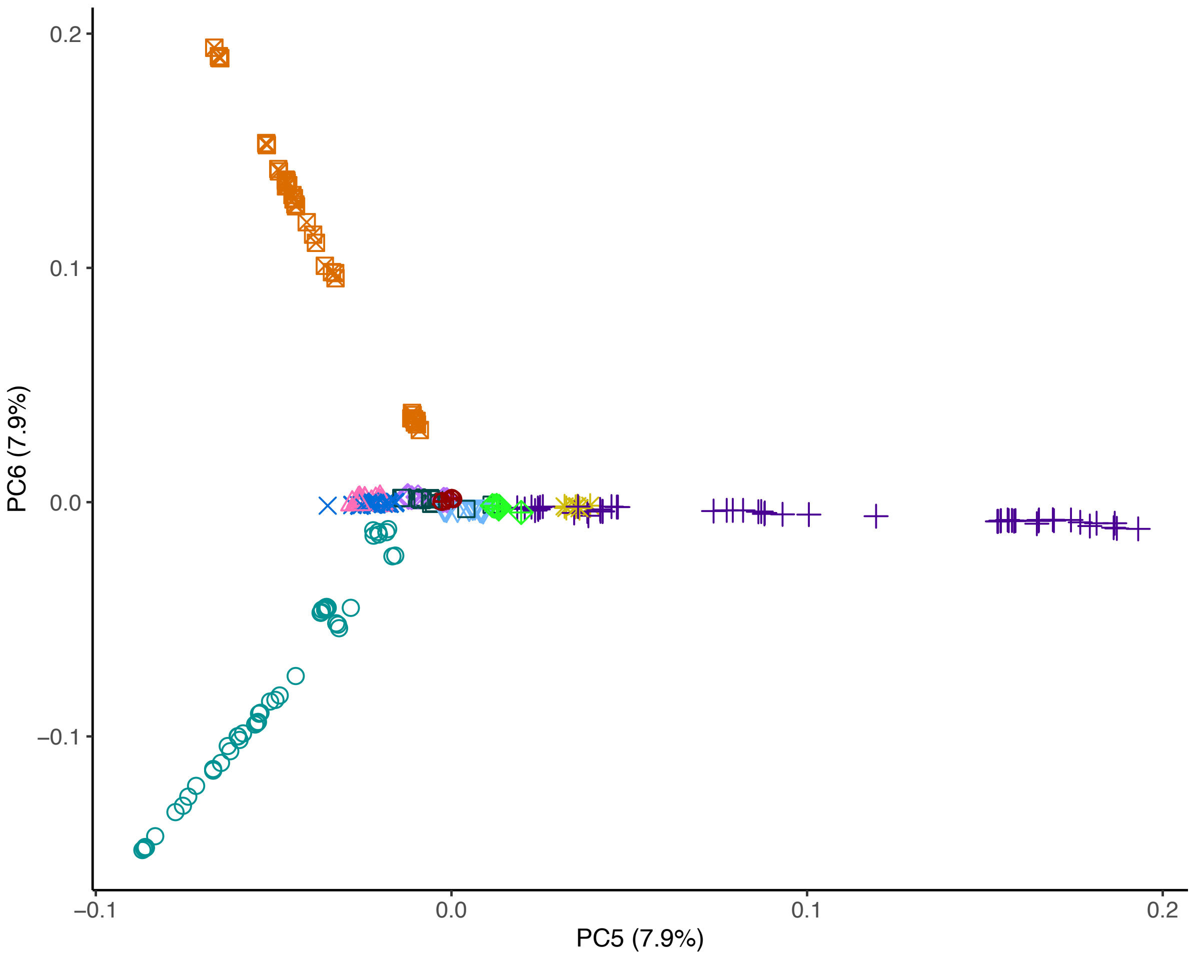

Supplementary Figure 104. PCA analysis of main SNP dataset. A plot of PC5 and PC6 is shown. Colourations follow the BUSCO species tree in Supplementary Fig. 101.

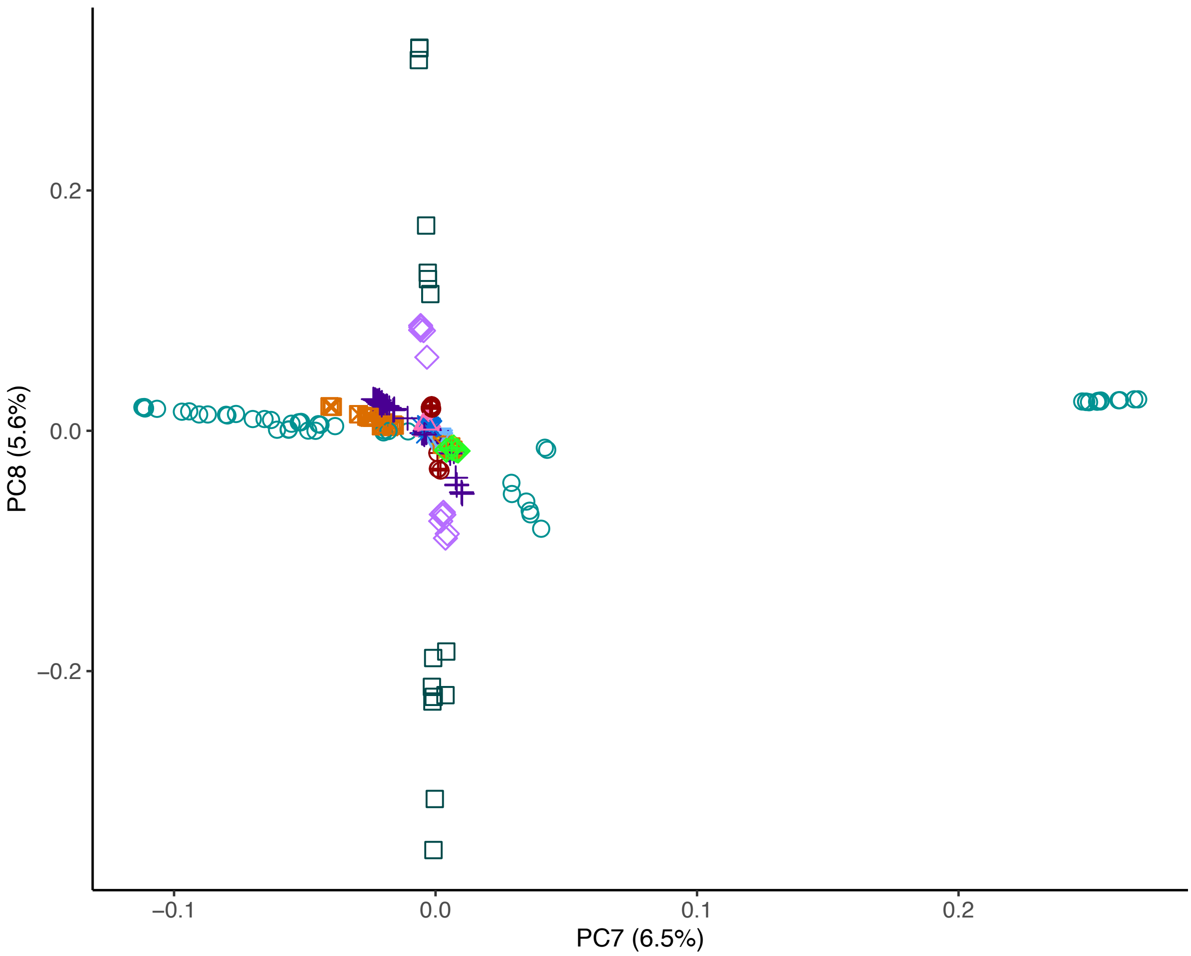

Supplementary Figure 105. PCA analysis of main SNP dataset. A plot of PC7 and PC8 is shown. Colourations follow the BUSCO species tree in Supplementary Fig. 101.

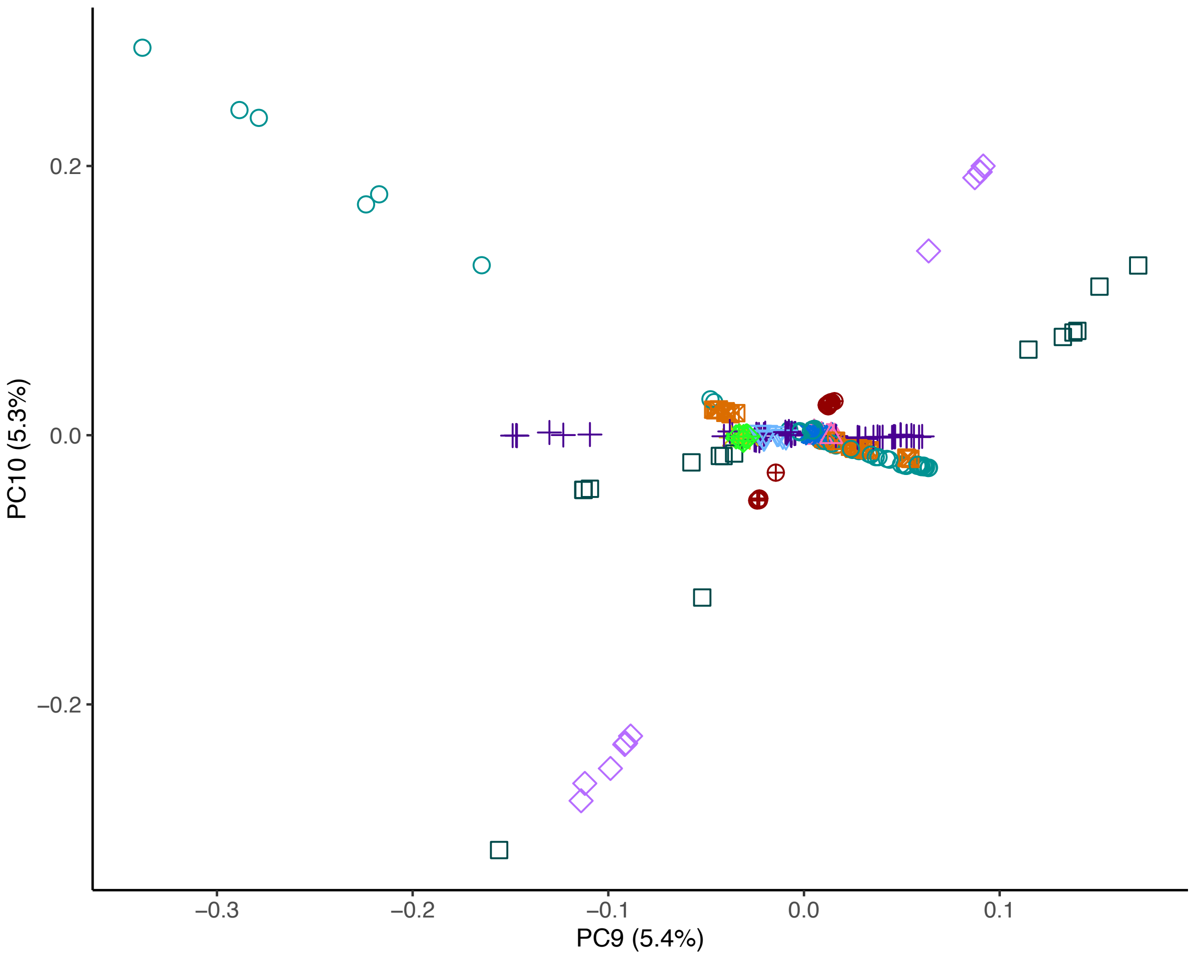

Supplementary Figure 106. PCA analysis of main SNP dataset. A plot of PC9 and PC10 is shown. Colourations follow the BUSCO species tree in Supplementary Fig. 101.

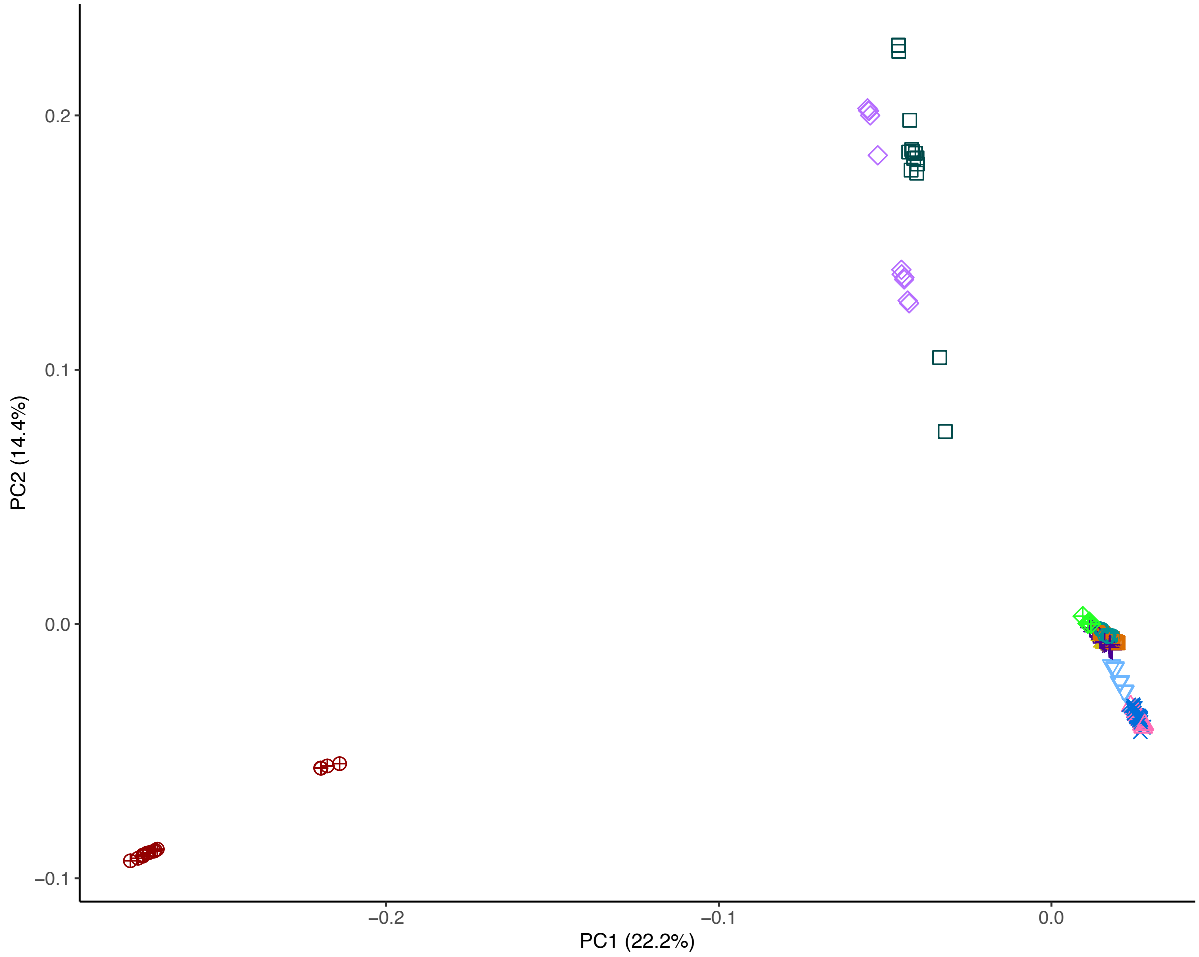

Supplementary Figure 107. PCA analysis of alternatively-filtered SNP dataset, where an LD filter check used. A plot of PC1 and PC2 is shown. Colourations follow the BUSCO species tree in Supplementary Fig. 101.

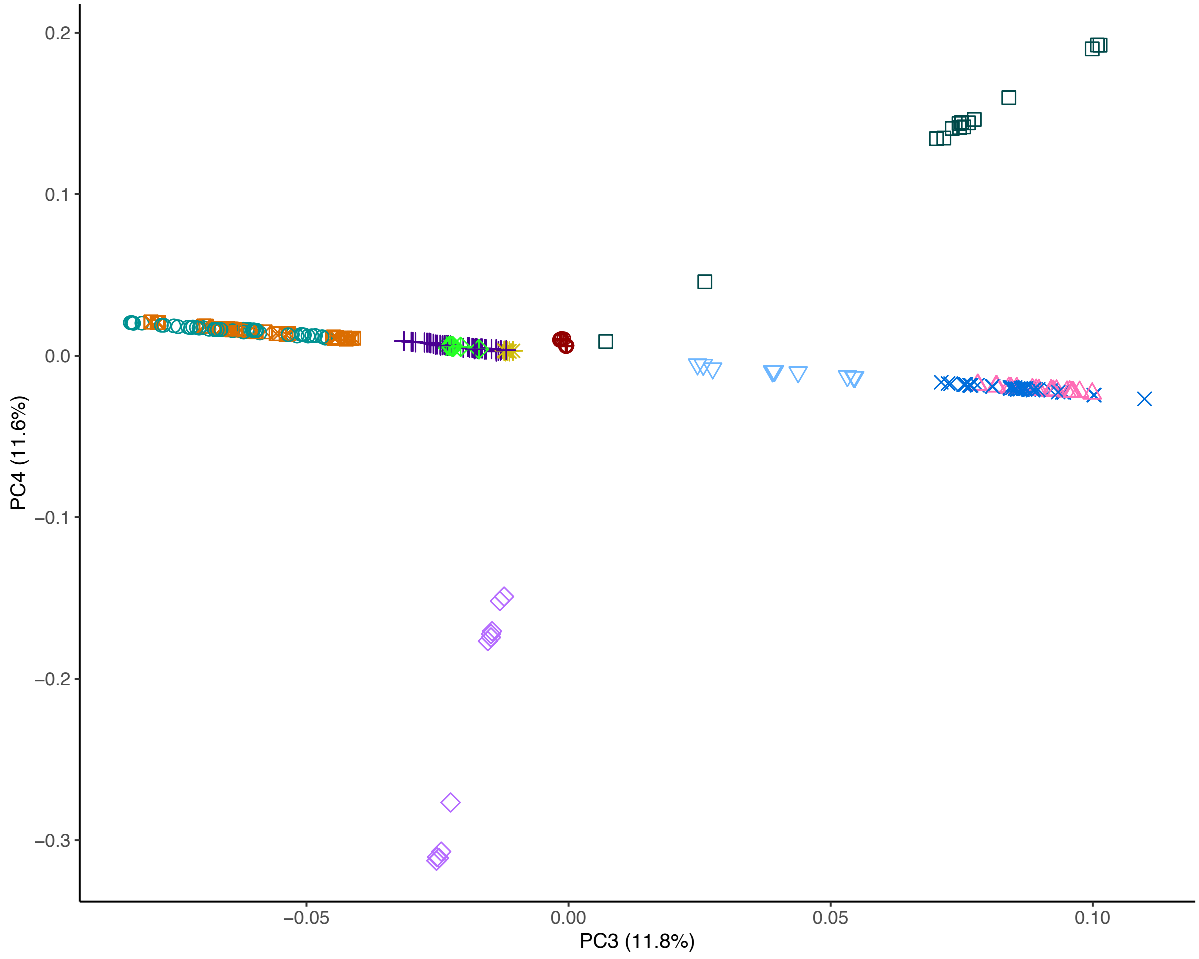

Supplementary Figure 108. PCA analysis of alternatively-filtered SNP dataset, where an LD filter check used. A plot of PC3 and PC4 is shown. Colourations follow the BUSCO species tree in Supplementary Fig. 101.

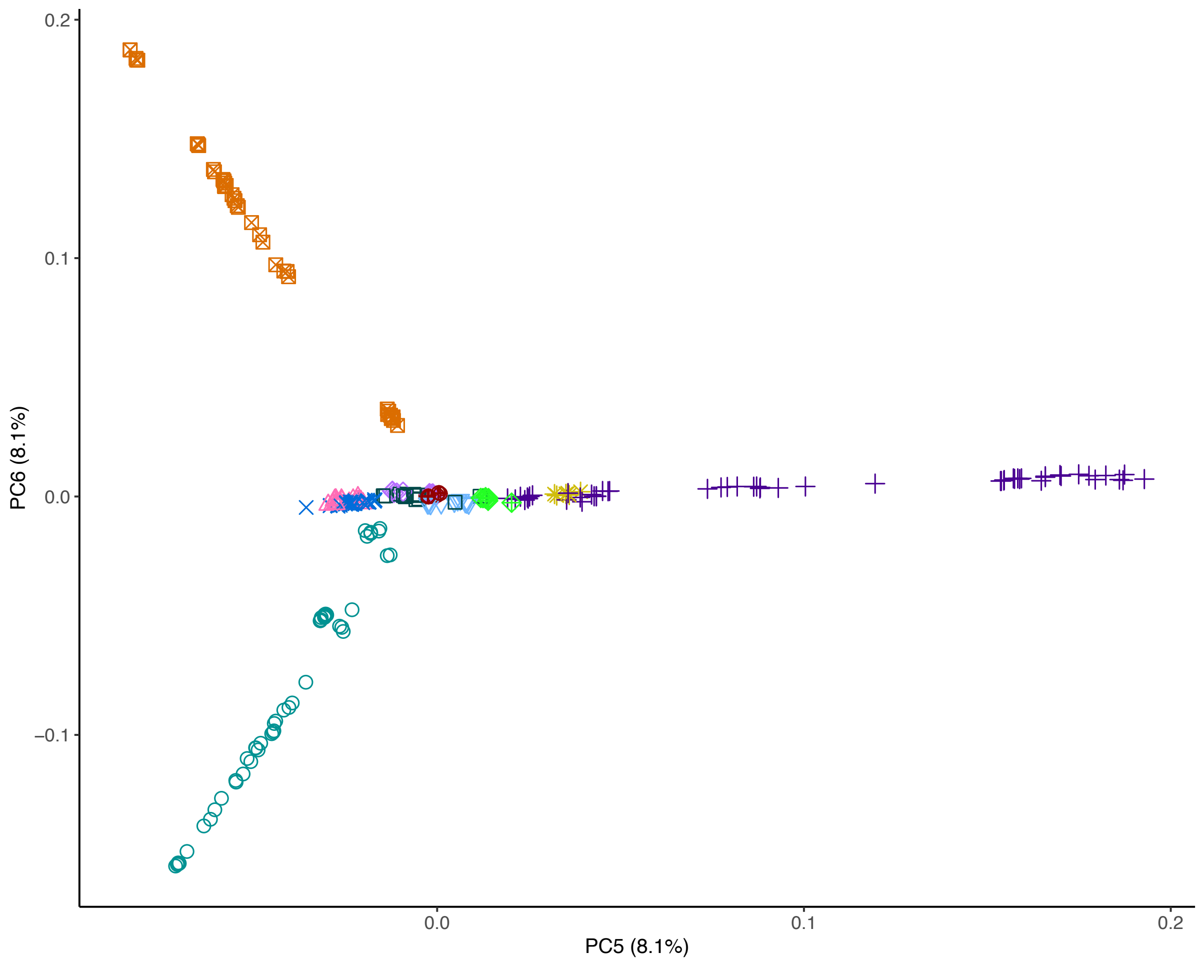

Supplementary Figure 109. PCA analysis of alternatively-filtered SNP dataset, where an LD filter check used. A plot of PC5 and PC6 is shown. Colourations follow the BUSCO species tree in Supplementary Fig. 101.

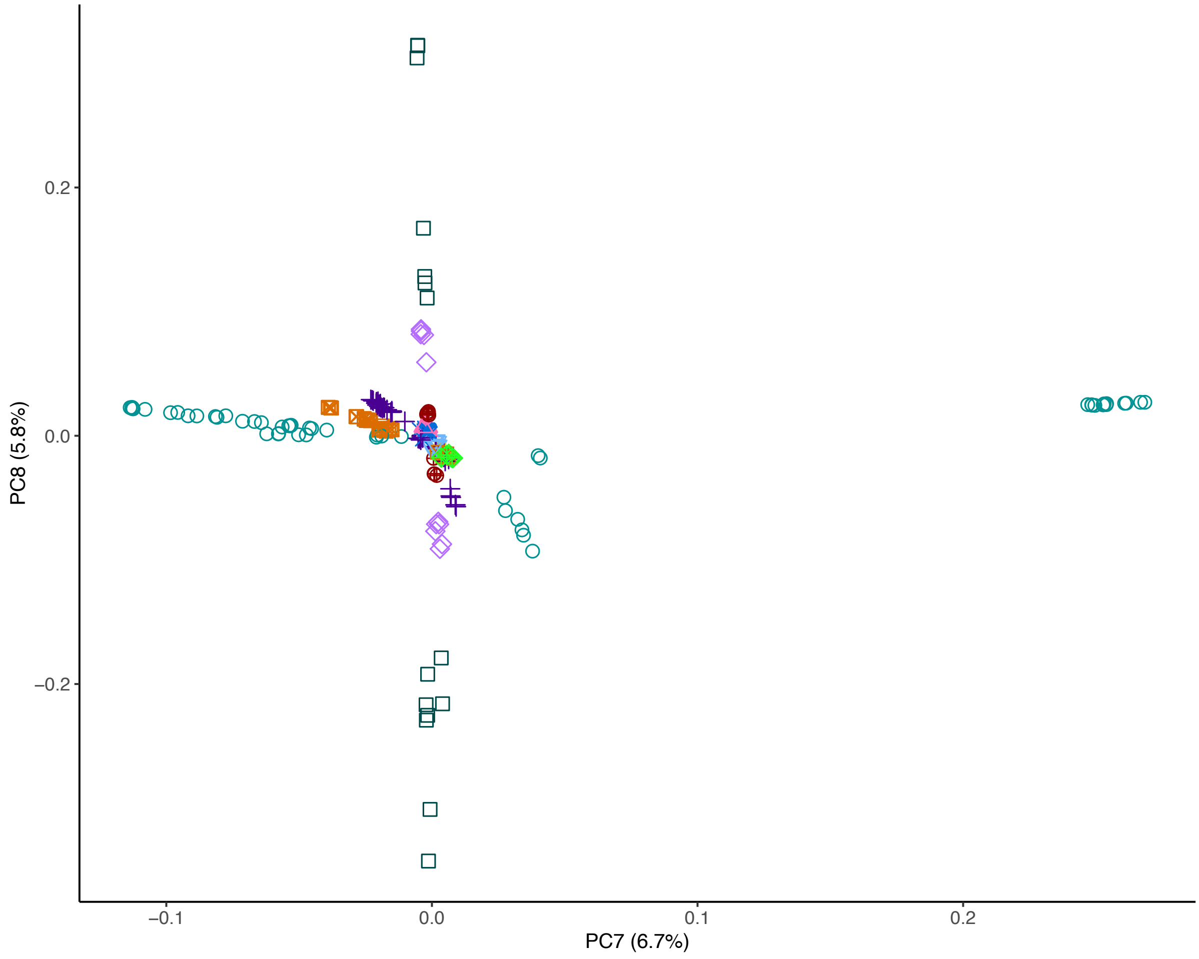

Supplementary Figure 110. PCA analysis of alternatively-filtered SNP dataset, where an LD filter check used. A plot of PC7 and PC8 is shown. Colourations follow the BUSCO species tree in Supplementary Fig. 101.

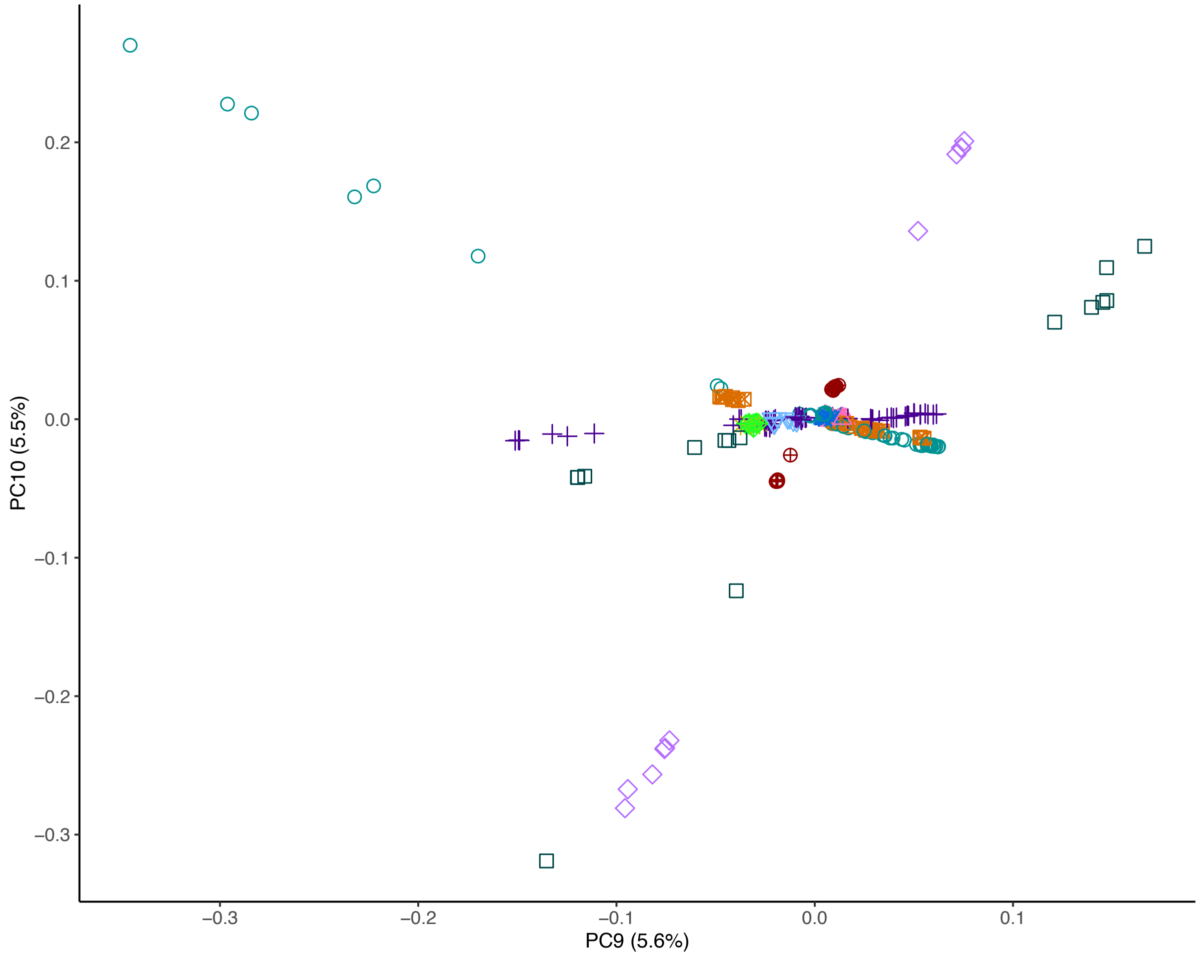

Supplementary Figure 111. PCA analysis of alternatively-filtered SNP dataset, where an LD filter check used. A plot of PC9 and PC10 is shown. Colourations follow the BUSCO species tree in Supplementary Fig. 101.

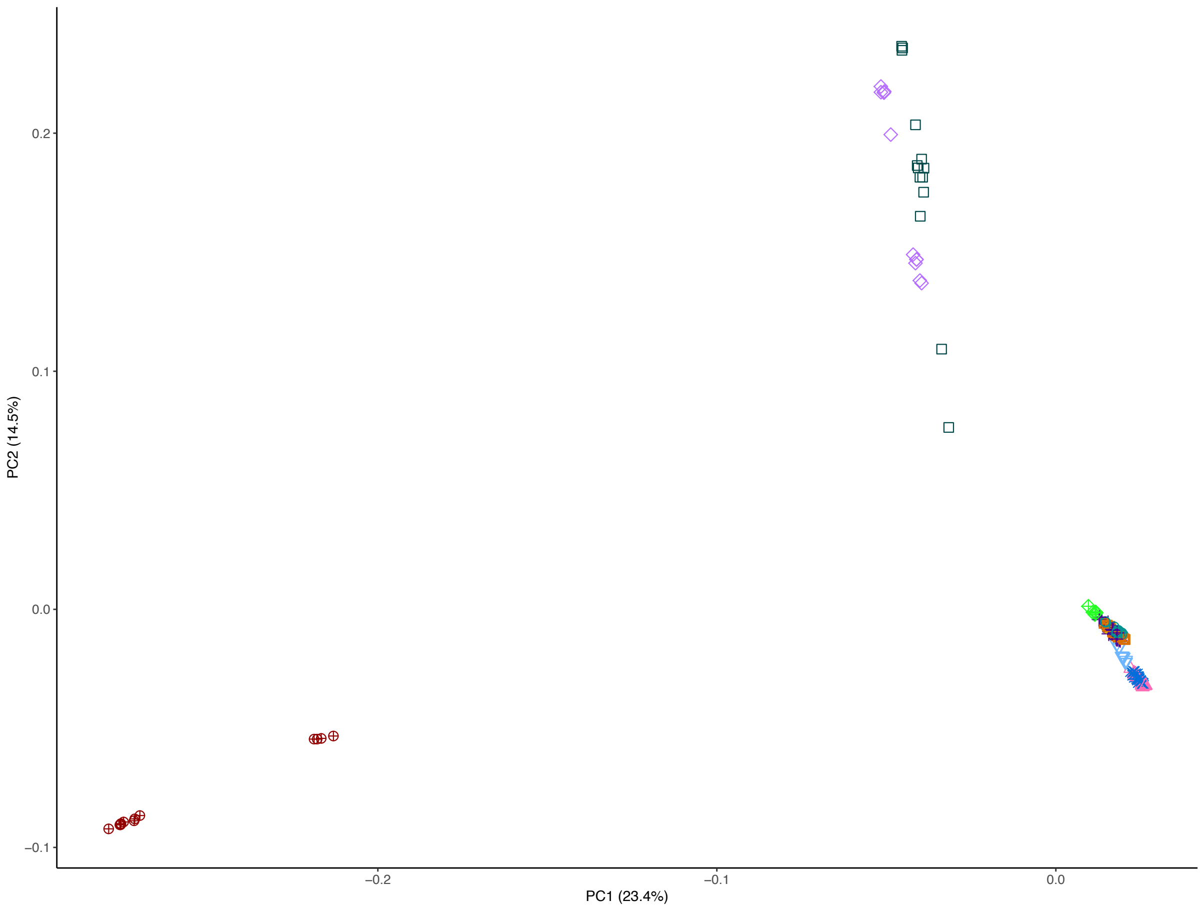

Supplementary Figure 112. PCA analysis of alternatively-filtered SNP dataset, where a minDP 20 filter was used. A plot of PC1 and PC2 is shown. Colourations follow the BUSCO species tree in Supplementary Fig. 101.

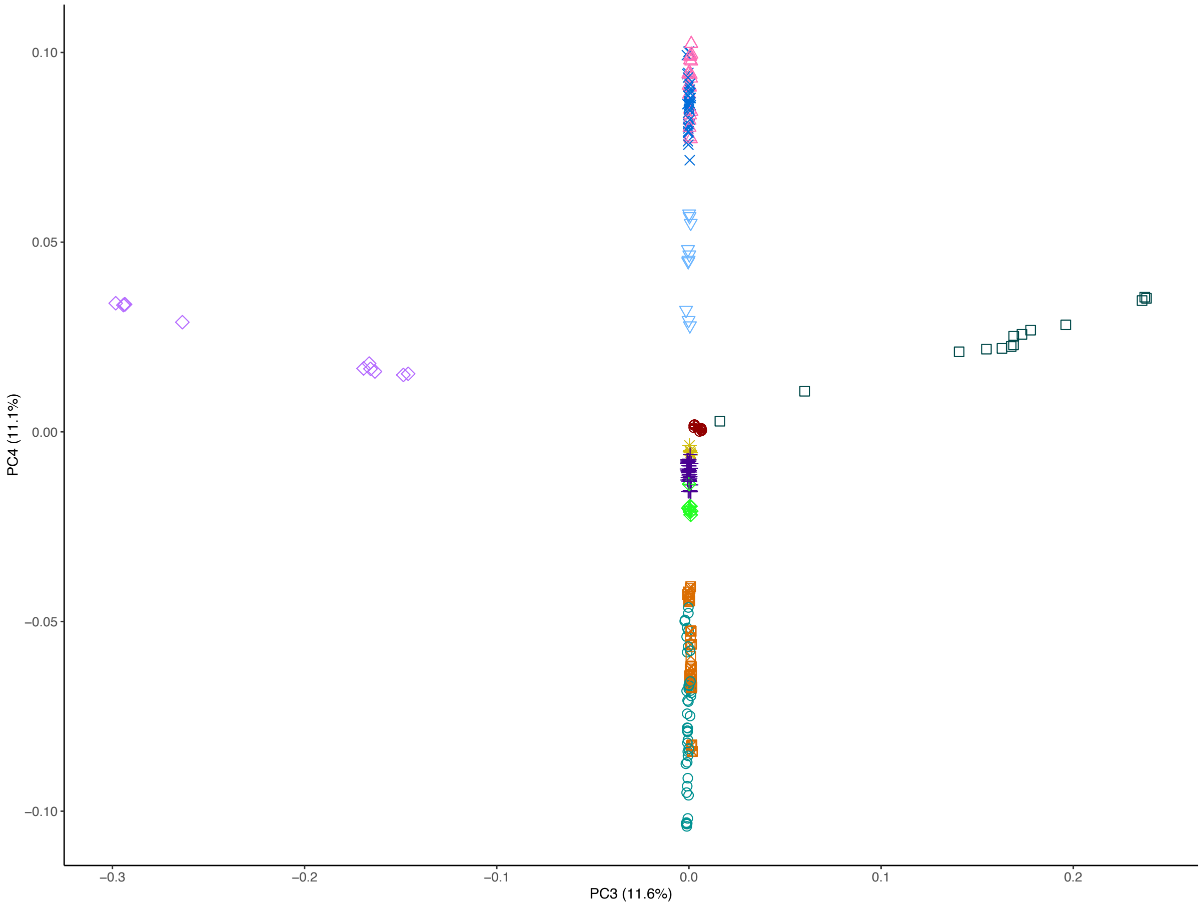

Supplementary Figure 113. PCA analysis of alternatively-filtered SNP dataset, where a minDP 20 filter was used. A plot of PC3 and PC4 is shown. Colourations follow the BUSCO species tree in Supplementary Fig. 101.

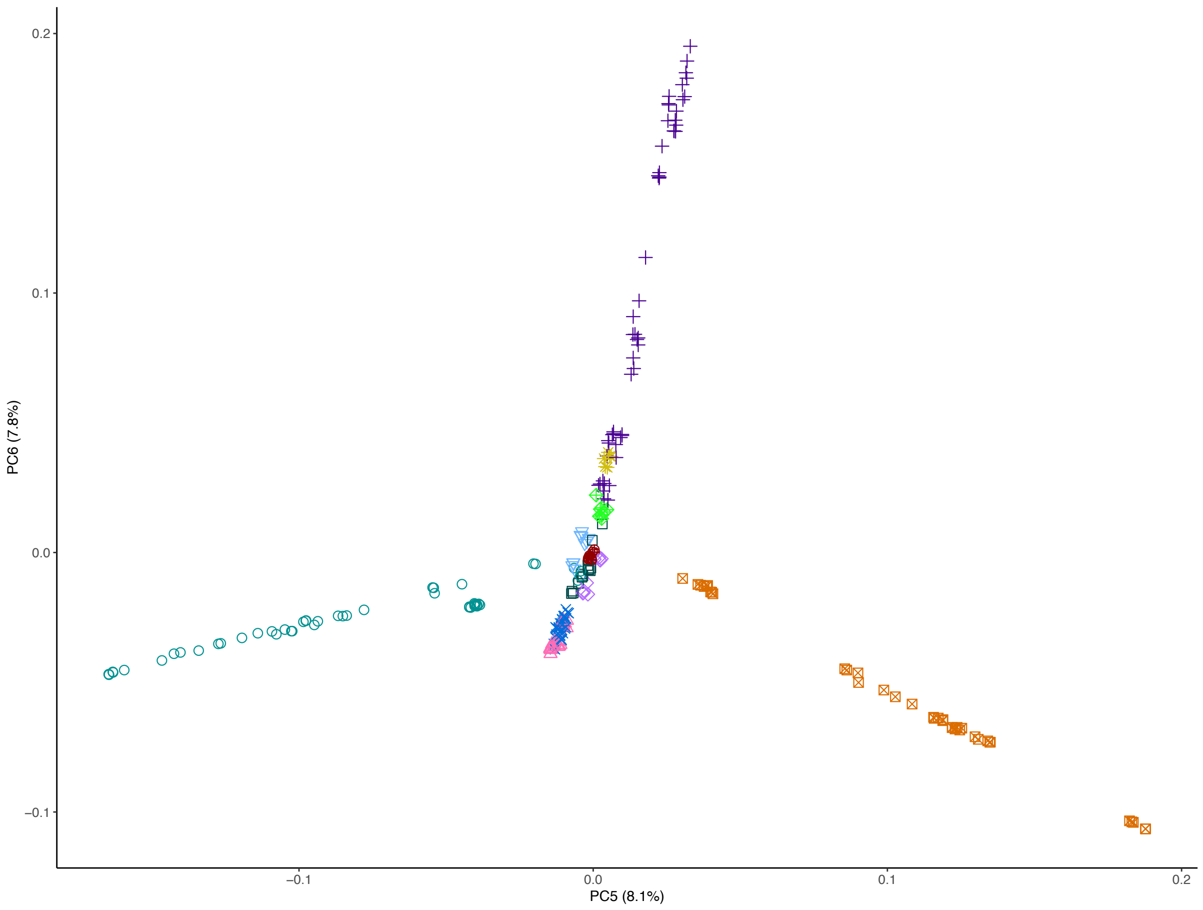

Supplementary Figure 114. PCA analysis of alternatively-filtered SNP dataset, where a minDP 20 filter was used. A plot of PC5 and PC6 is shown. Colourations follow the BUSCO species tree in Supplementary Fig. 101.

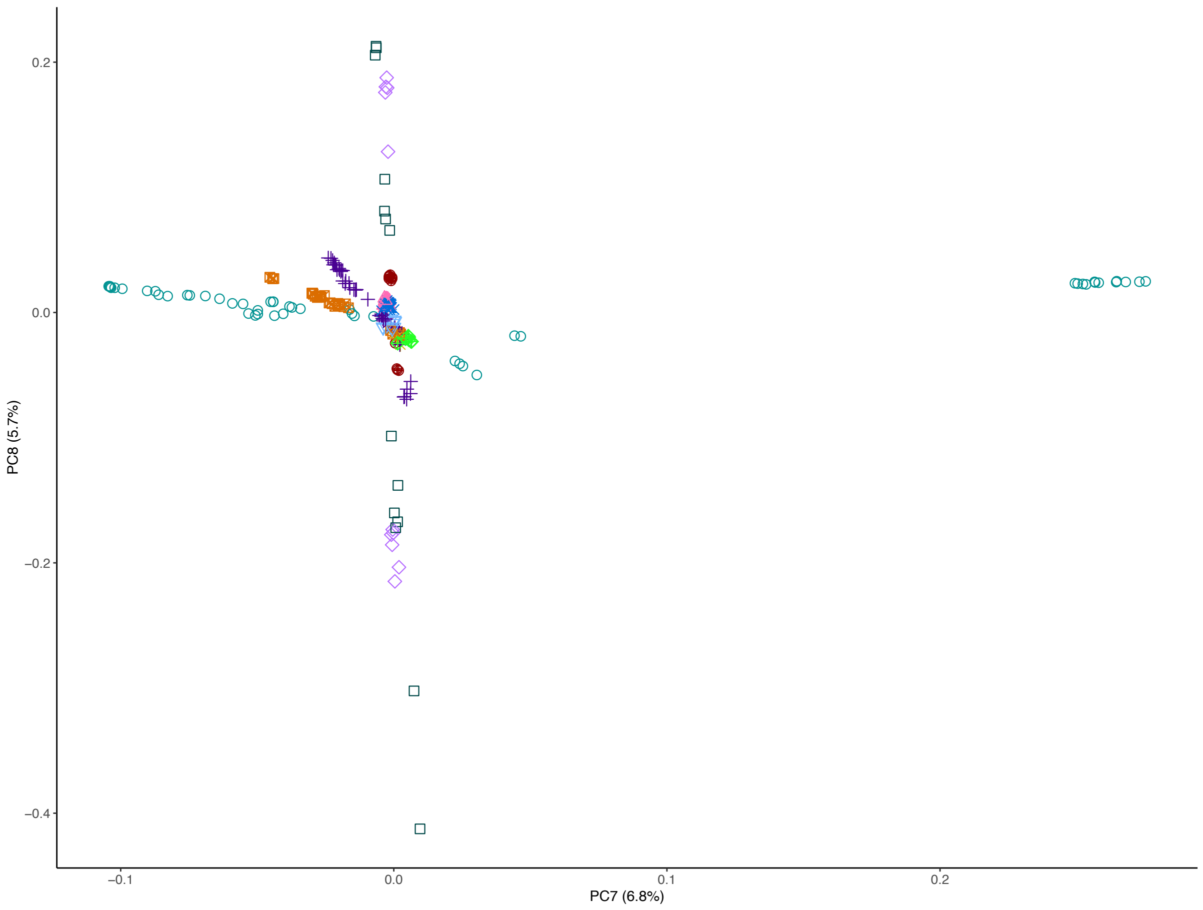

Supplementary Figure 115. PCA analysis of alternatively-filtered SNP dataset, where a minDP 20 filter was used. A plot of PC7 and PC8 is shown. Colourations follow the BUSCO species tree in Supplementary Fig. 101.

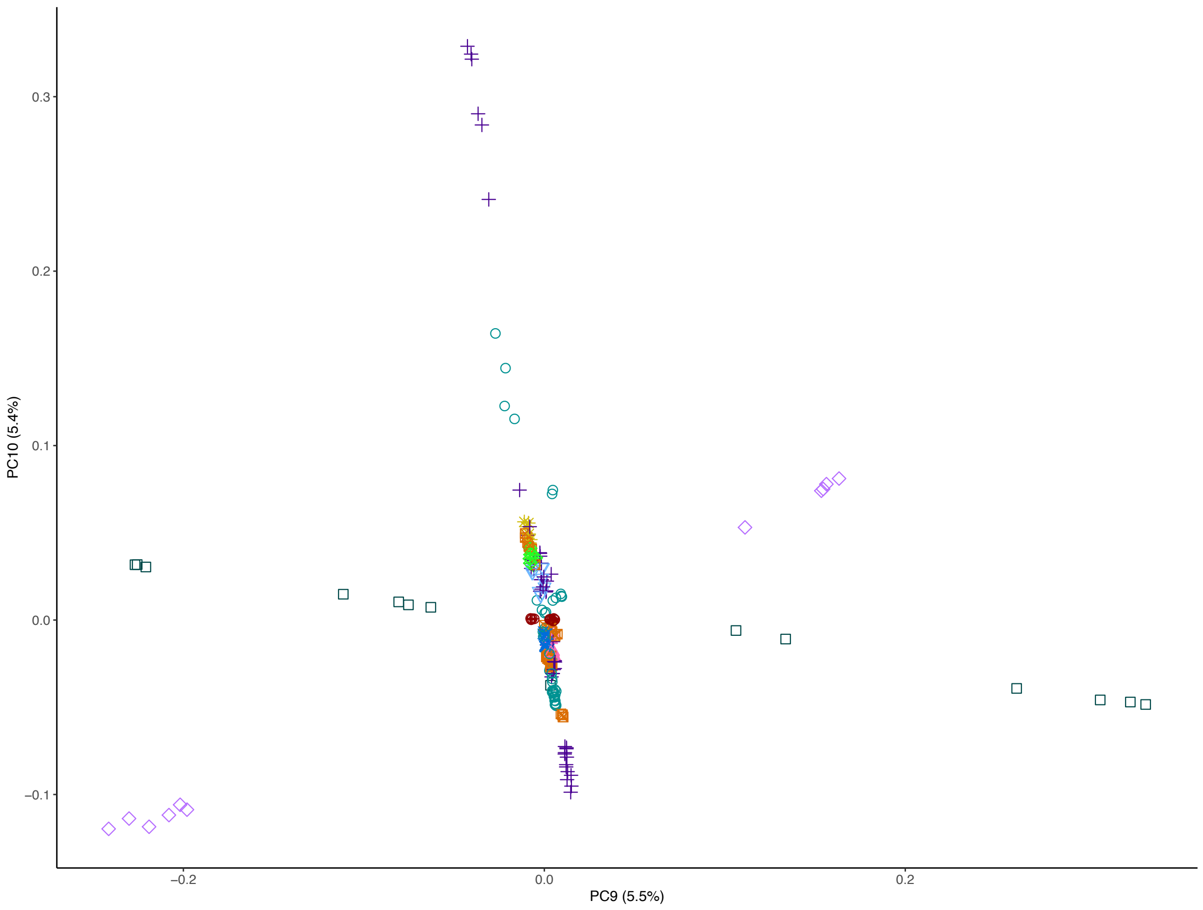

Supplementary Figure 116. PCA analysis of alternatively-filtered SNP dataset, where a minDP 20 filter was used. A plot of PC9 and PC10 is shown. Colourations follow the BUSCO species tree in Supplementary Fig. 101.

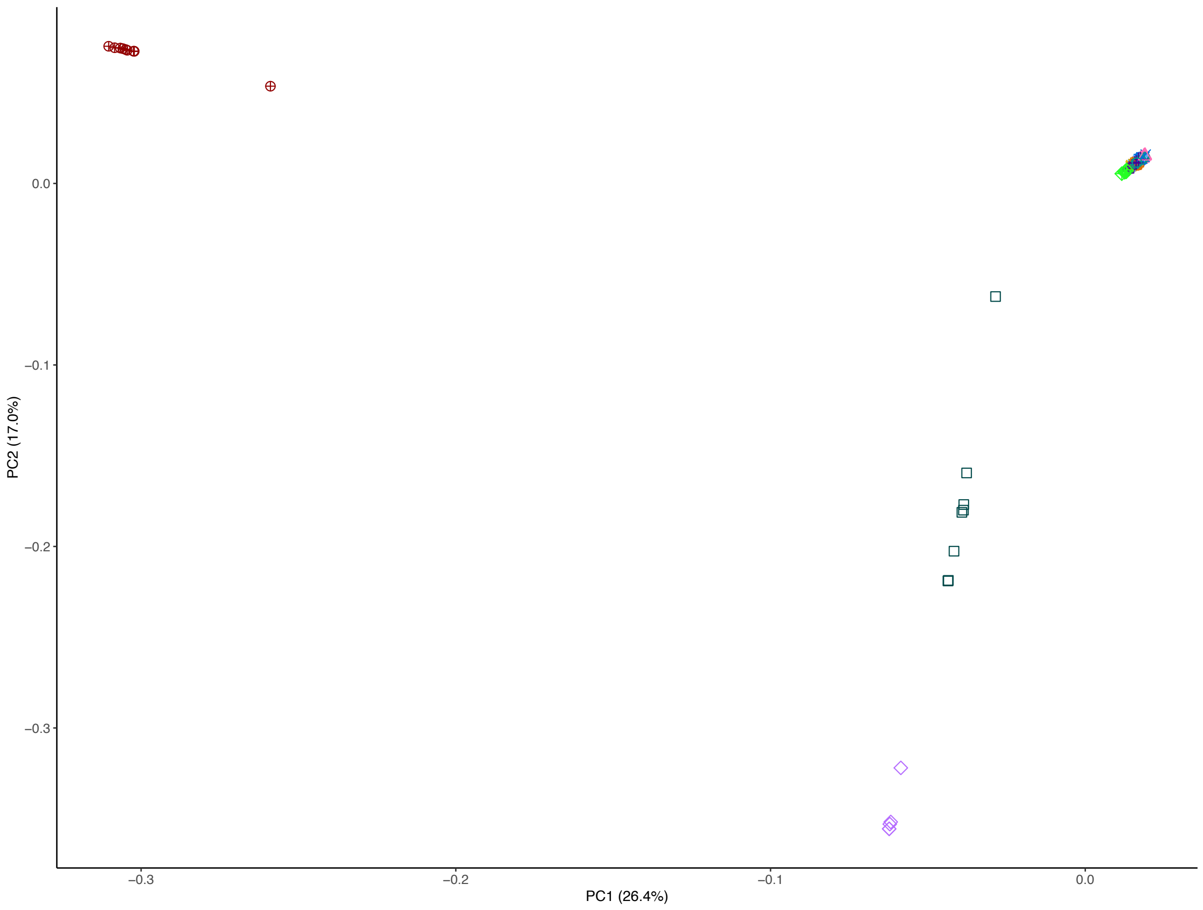

Supplementary Figure 117. PCA analysis of alternatively-filtered SNP dataset, where no HETs filter was used. A plot of PC1 and PC2 is shown. Colourations follow the BUSCO species tree in Supplementary Fig. 101.

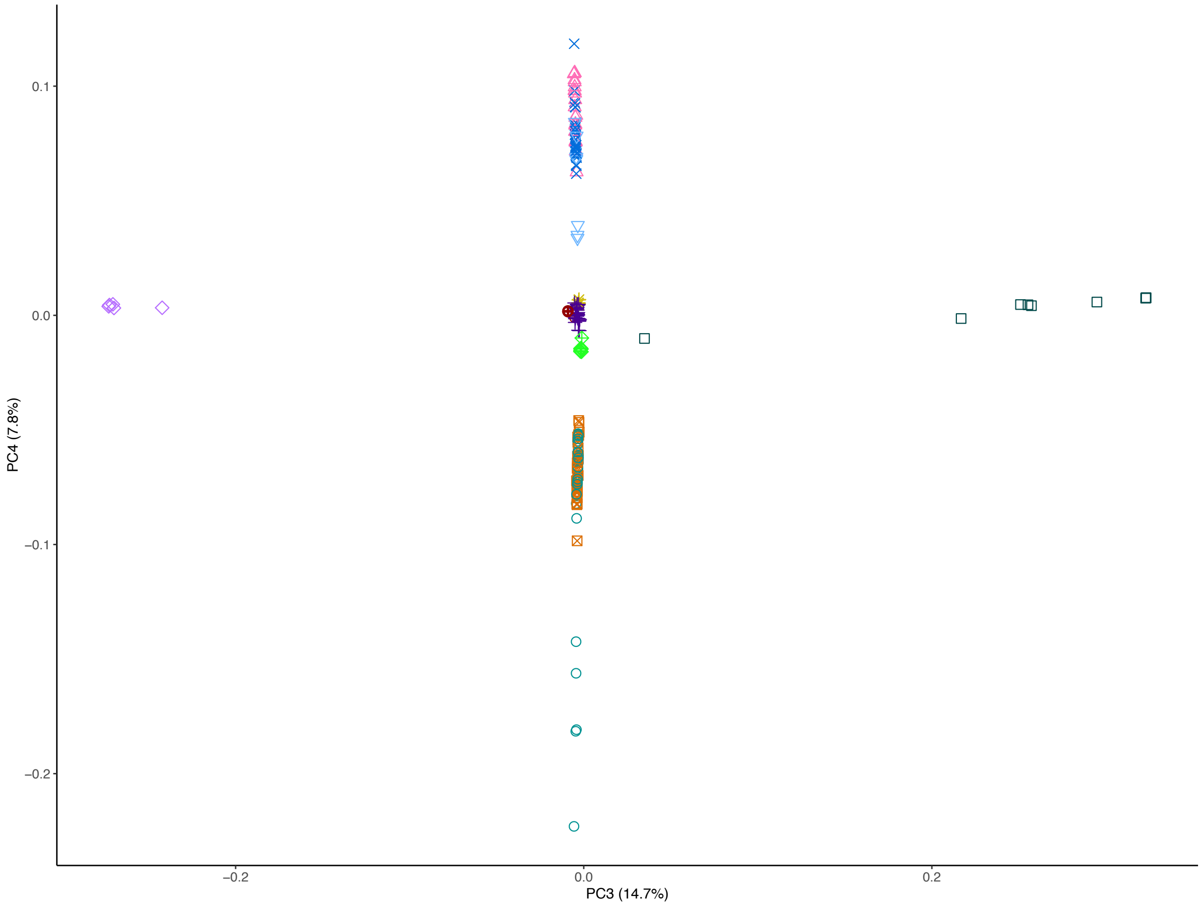

Supplementary Figure 118. PCA analysis of alternatively-filtered SNP dataset, where no HETs filter was used. A plot of PC3 and PC4 is shown. Colourations follow the BUSCO species tree in Supplementary Fig. 101.

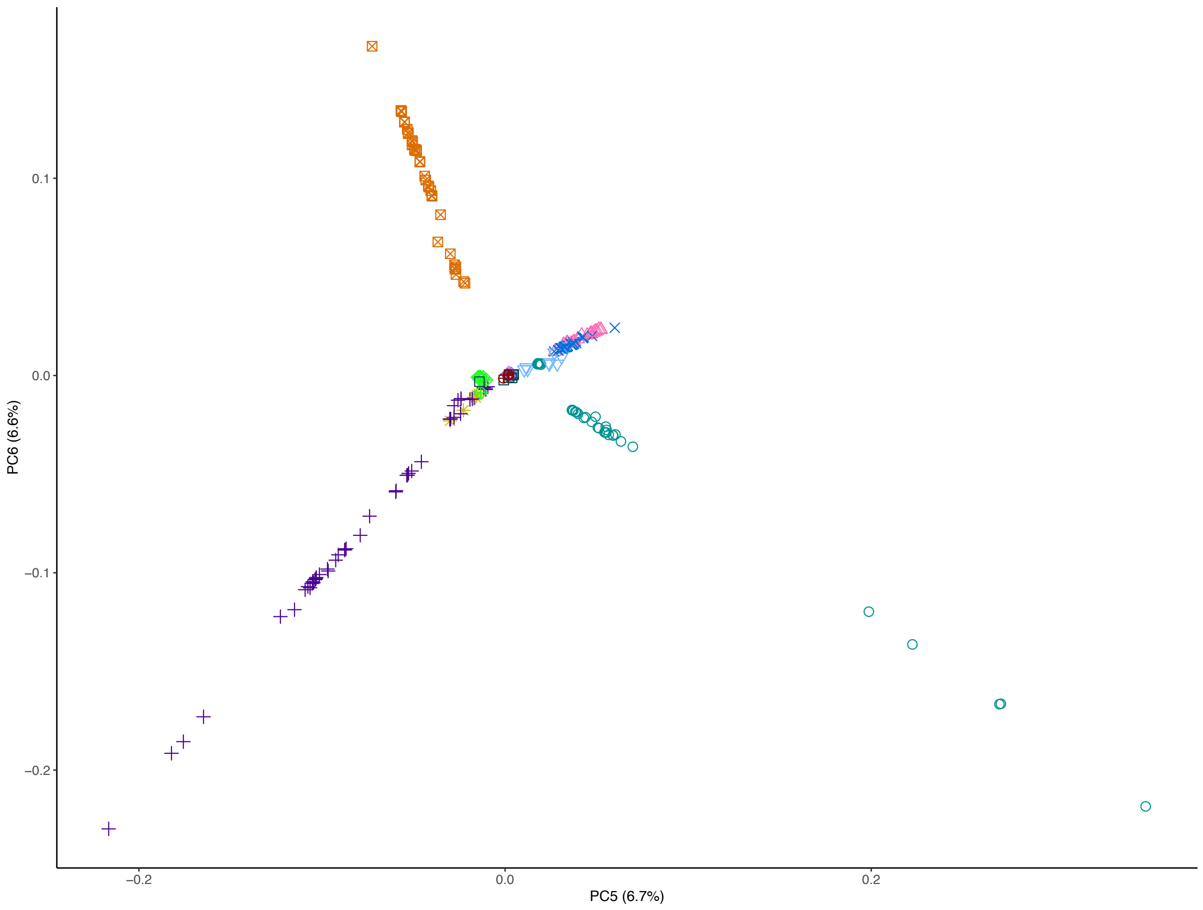

Supplementary Figure 119. PCA analysis of alternatively-filtered SNP dataset, where no HETs filter was used. A plot of PC5 and PC6 is shown. Colourations follow the BUSCO species tree in Supplementary Fig. 101.

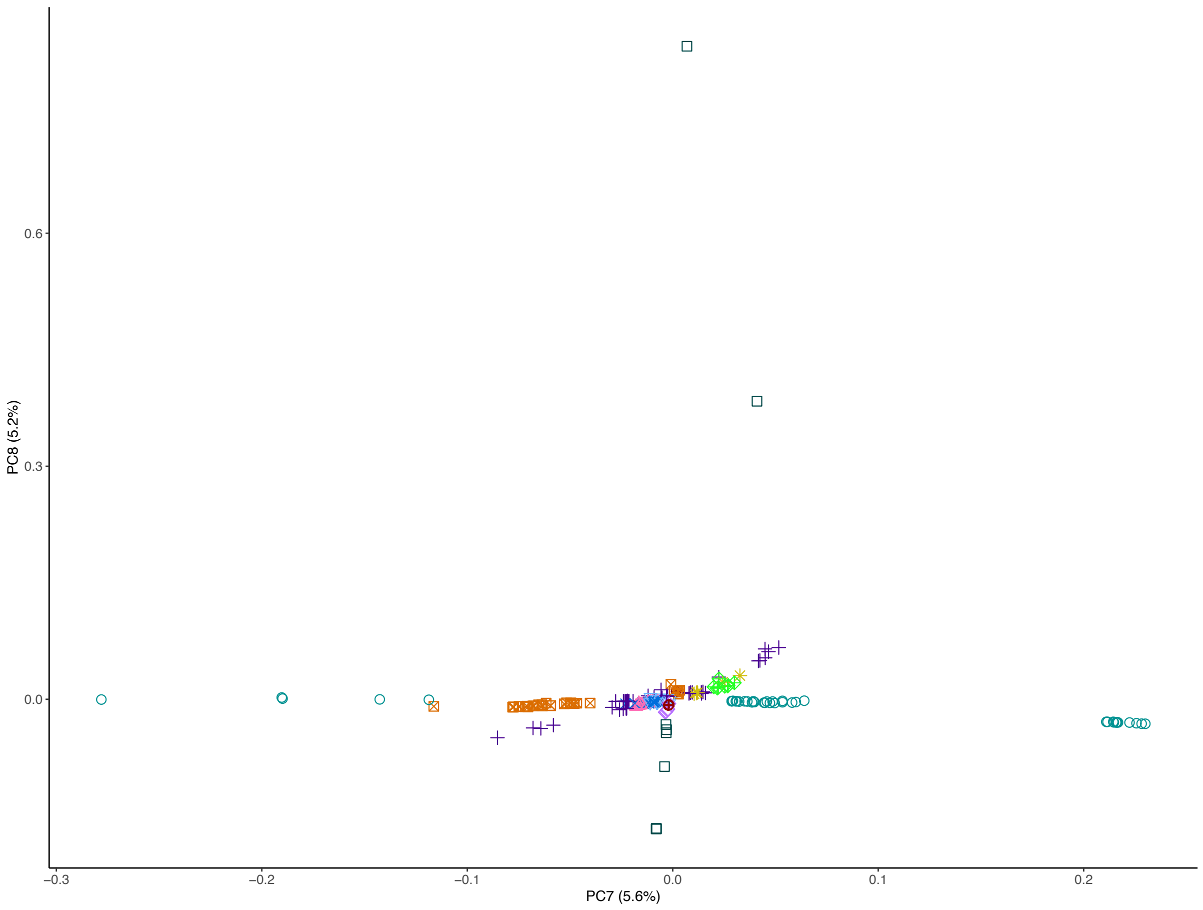

Supplementary Figure 120. PCA analysis of alternatively-filtered SNP dataset, where no HETs filter was used. A plot of PC7 and PC8 is shown. Colourations follow the BUSCO species tree in Supplementary Fig. 101.

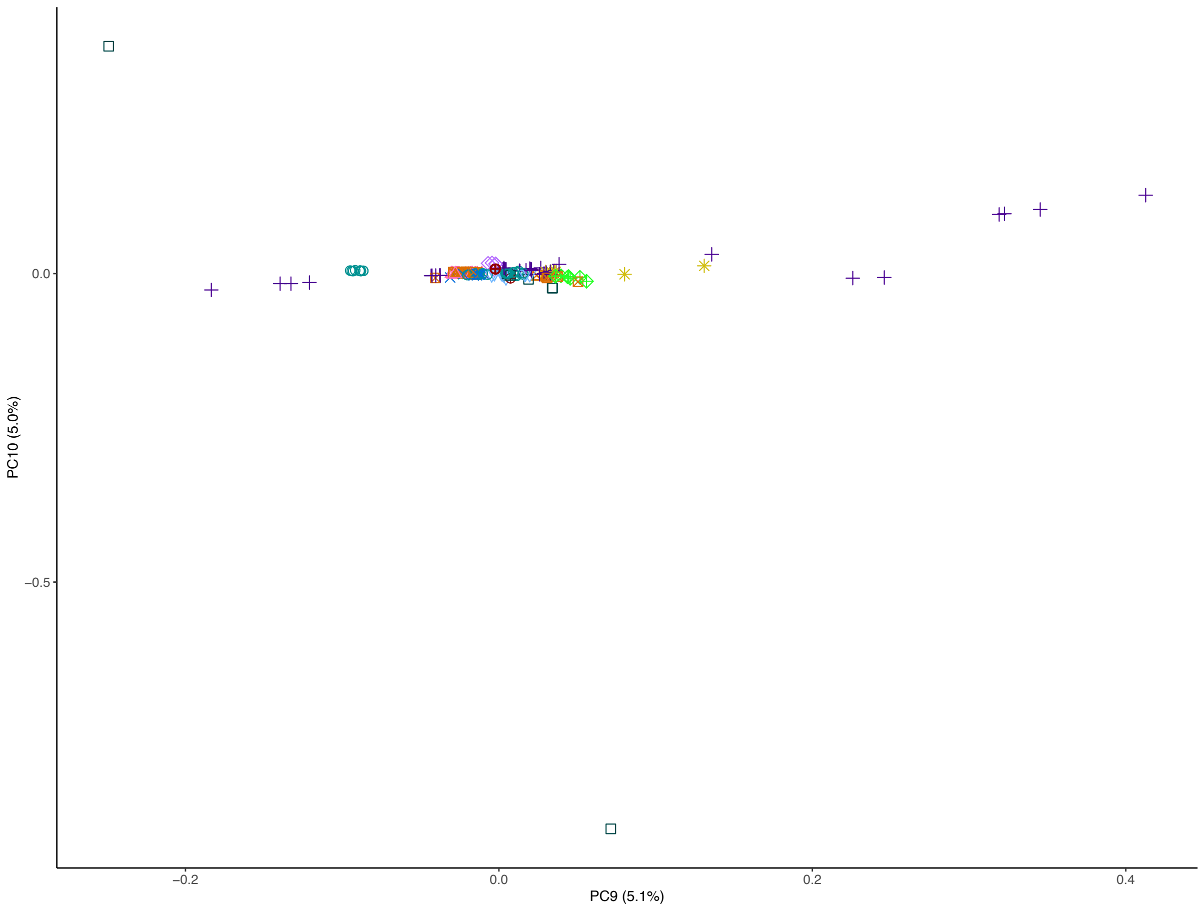

Supplementary Figure 121. PCA analysis of alternatively-filtered SNP dataset, where no HETs filter was used. A plot of PC9 and PC10 is shown. Colourations follow the BUSCO species tree in Supplementary Fig. 101.

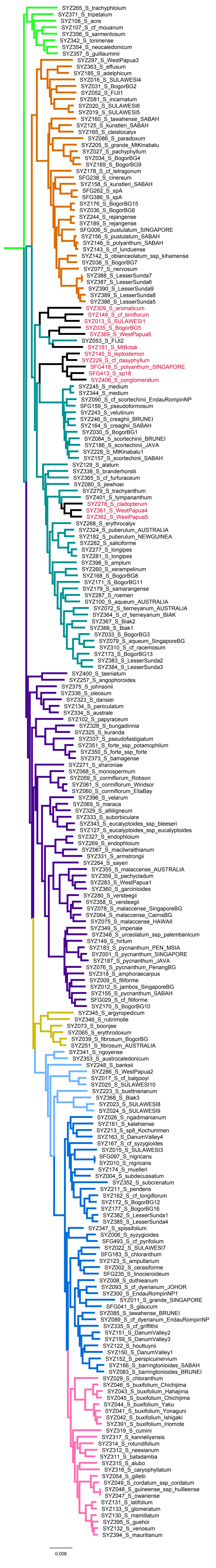

Supplementary Figure 122. PCA analysis of the Syzygium grande group. SNP tree coloured according to taxon labelling used. The taxon names coloured in red indicate those individuals automatically excluded by the smartpca software.

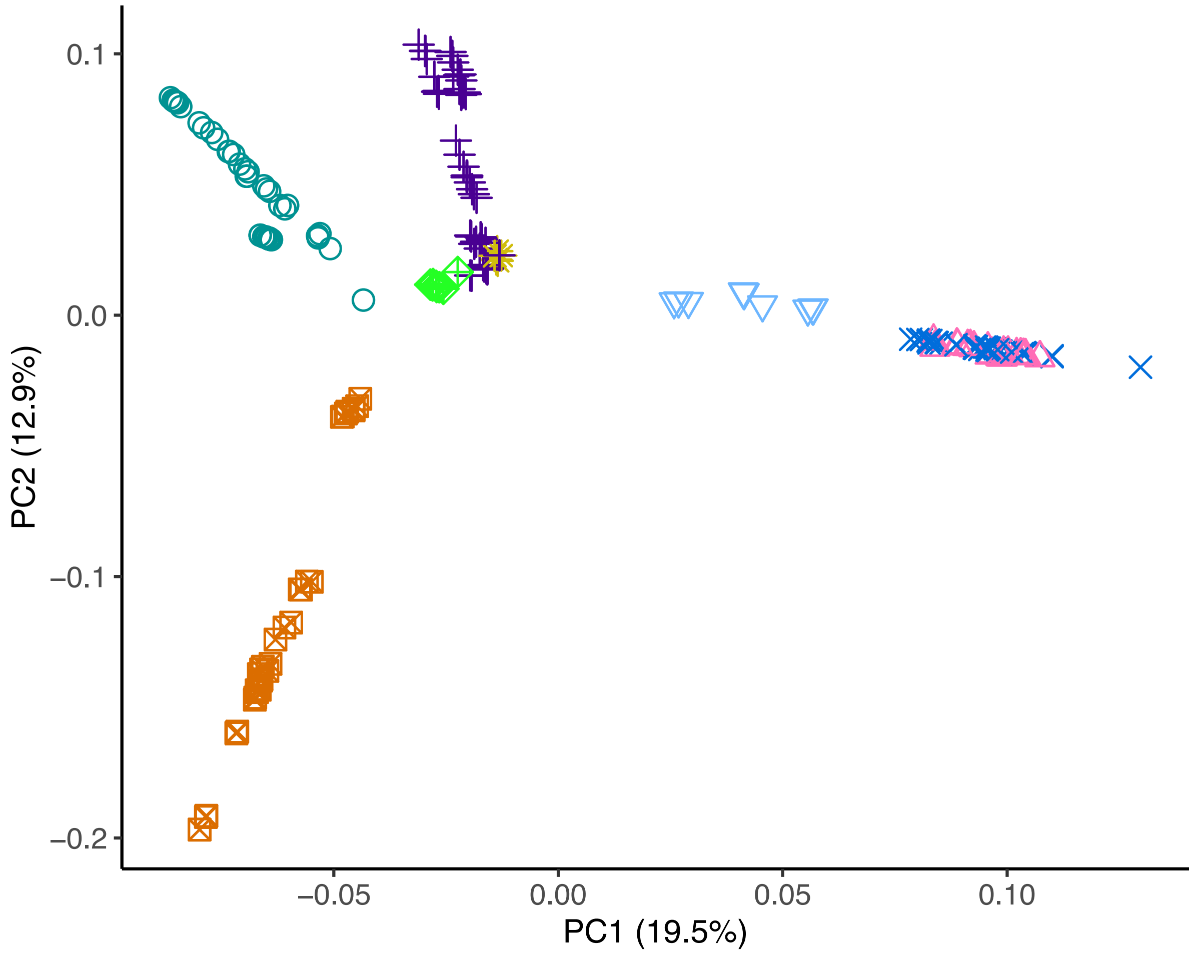

Supplementary Figure 123. PCA analysis of the *Syzygium grande* group. A plot of PC1 and PC2 is shown. Colourations follow the SNP tree in Supplementary Fig. 122.

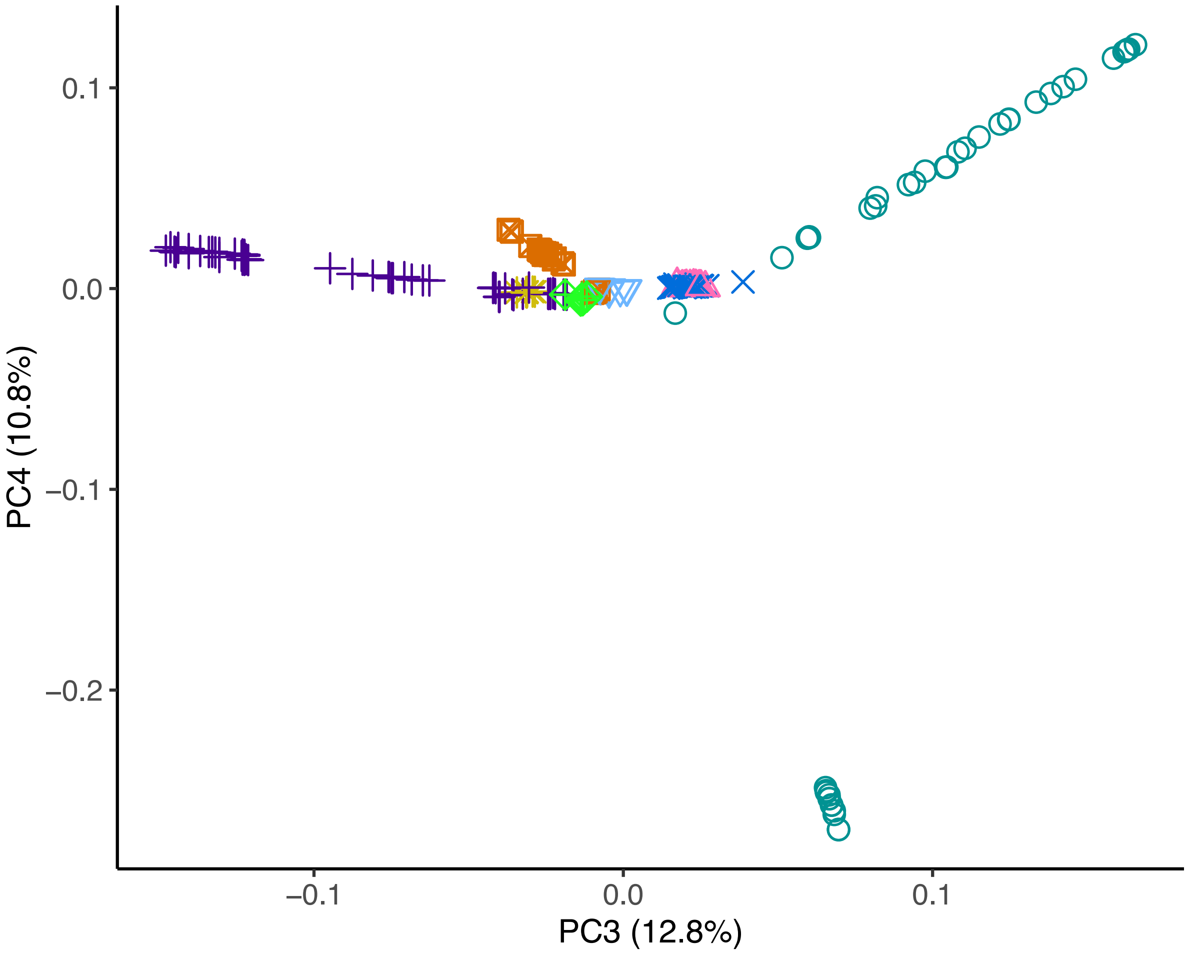

Supplementary Figure 124. PCA analysis of the *Syzygium grande* group. A plot of PC3 and PC4 is shown. Colourations follow the SNP tree in Supplementary Fig. 122.

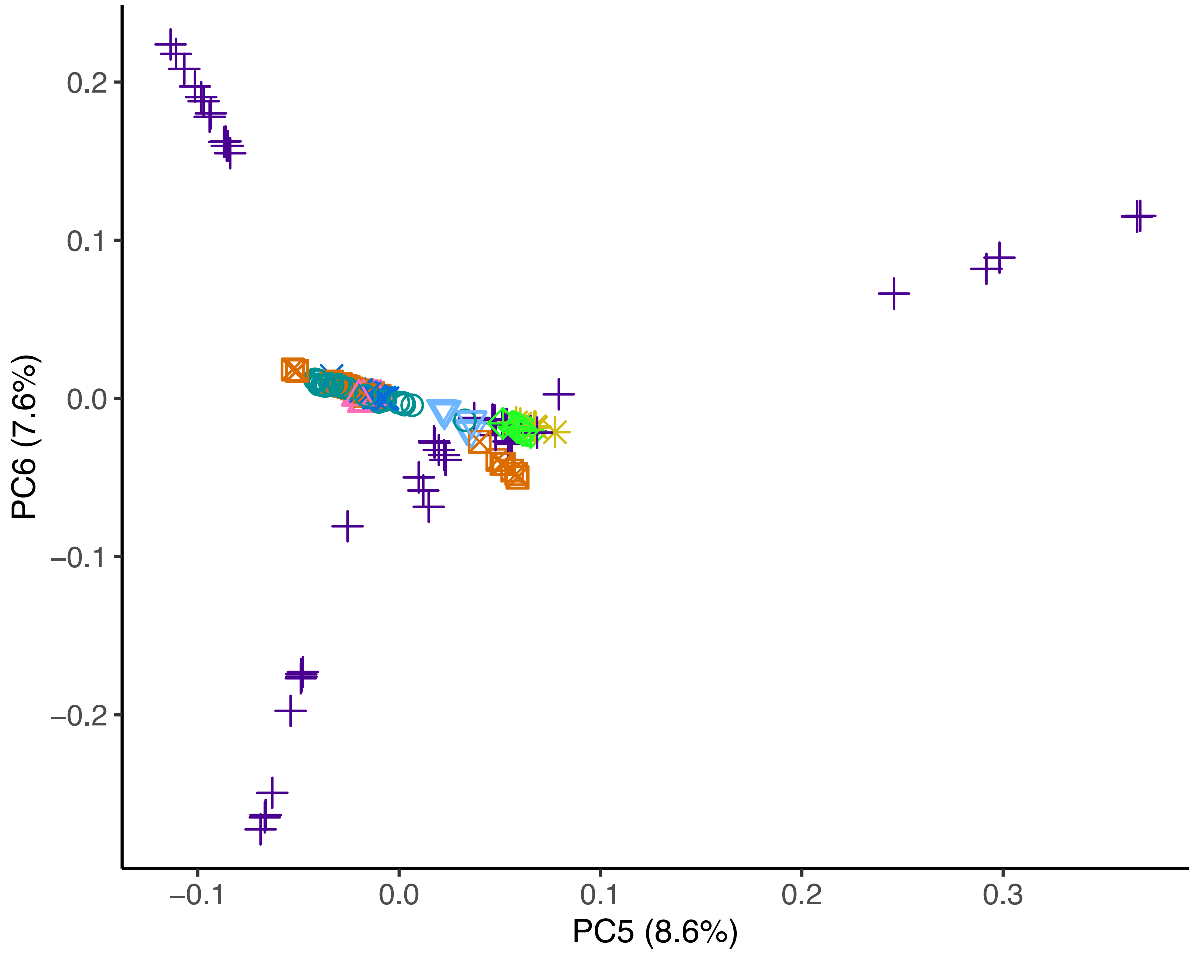

Supplementary Figure 125. PCA analysis of the *Syzygium grande* group. A plot of PC5 and PC6 is shown. Colourations follow the SNP tree in Supplementary Fig. 122.

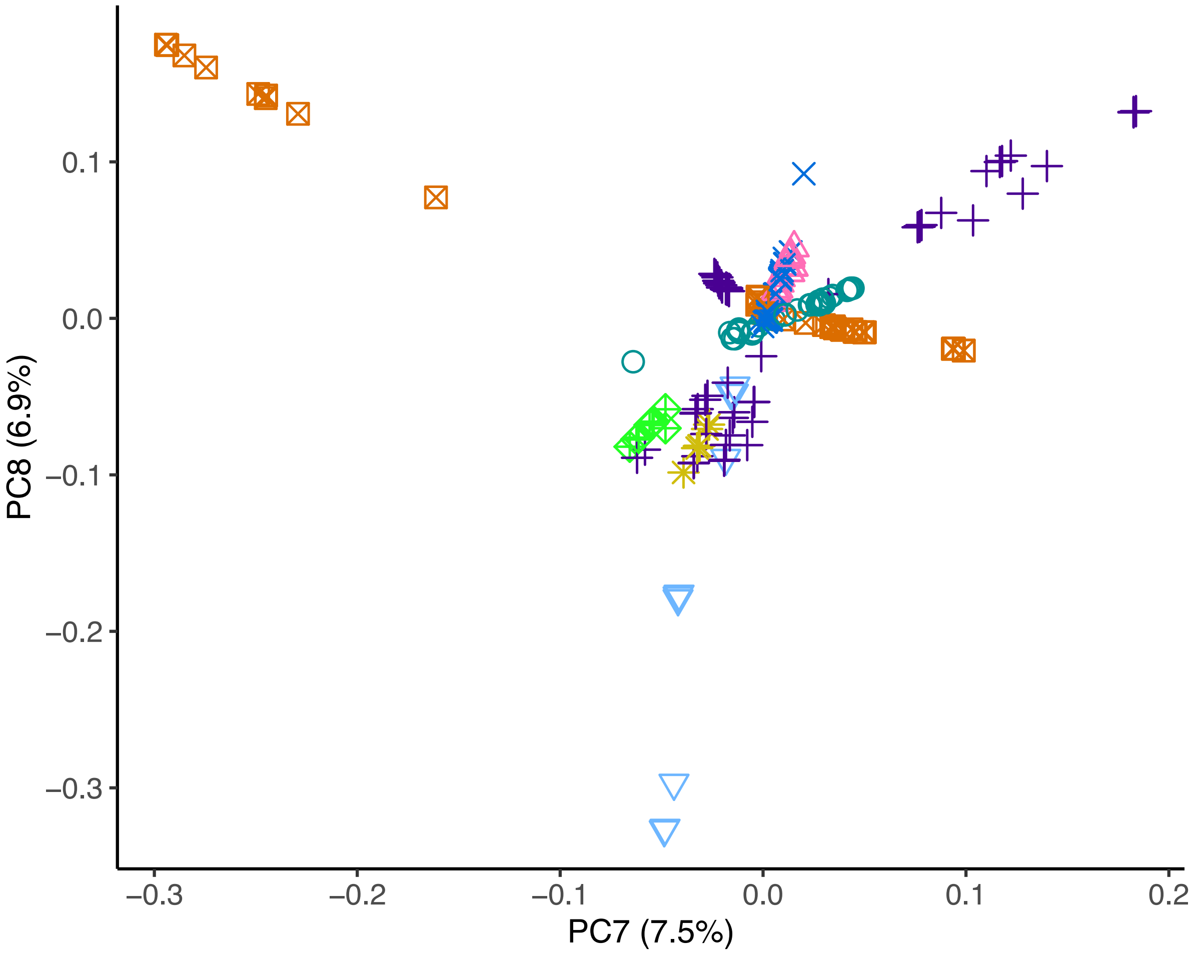

Supplementary Figure 126. PCA analysis of the *Syzygium grande* group. A plot of PC7 and PC8 is shown. Colourations follow the SNP tree in Supplementary Fig. 122.

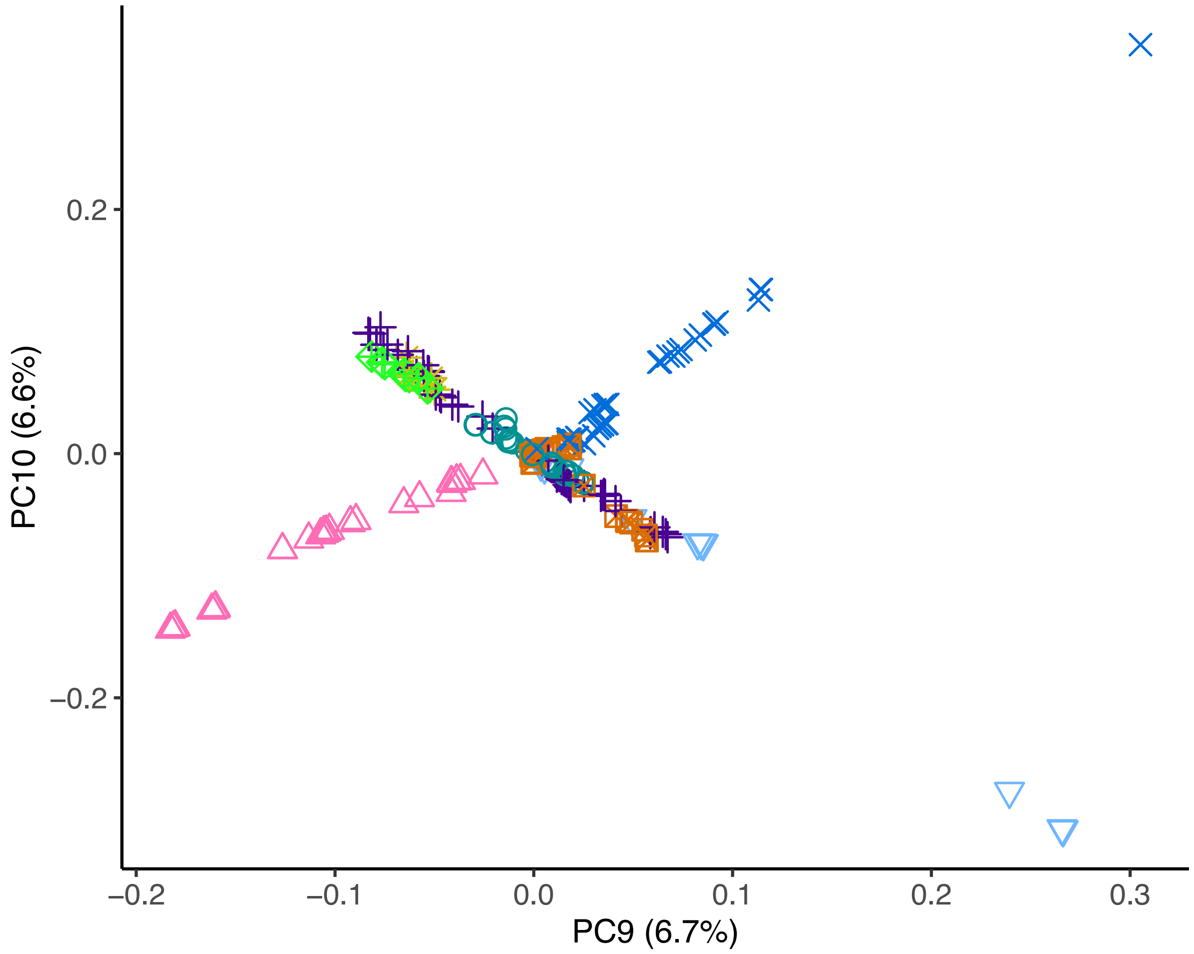

Supplementary Figure 127. PCA analysis of the *Syzygium grande* group. A plot of PC9 and PC10 is shown. Colourations follow the SNP tree in Supplementary Fig. 122.

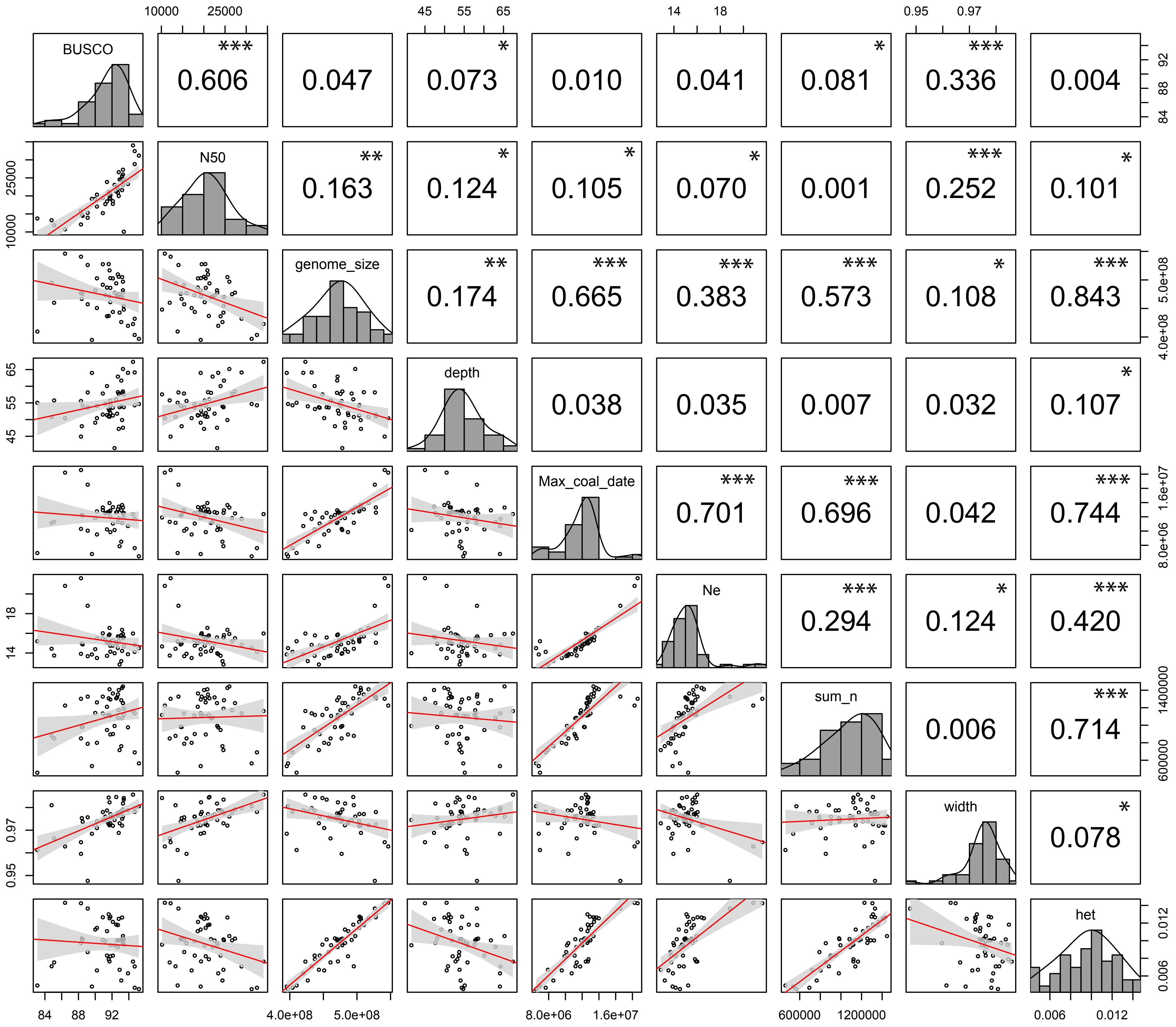

Supplementary Figure 128. Pairwise correlation matrix, including all linear regressions for BUSCO scores, assembly N50s, genome sizes, coverage depths, maximum coalescence date, effective population size (Ne), segregating sites (sum_n) calculated by PSMC analyses, coverage widths, and heterozygosities from SNP calls (het). Shown below the diagonal are scatterplots with linear regressions and 95% confidence intervals; sampling histograms are on the diagonal; above the diagonal are R-squared values and stars representing significance p-values (<0.001=***, <0.01=**, and <0.05 =*).

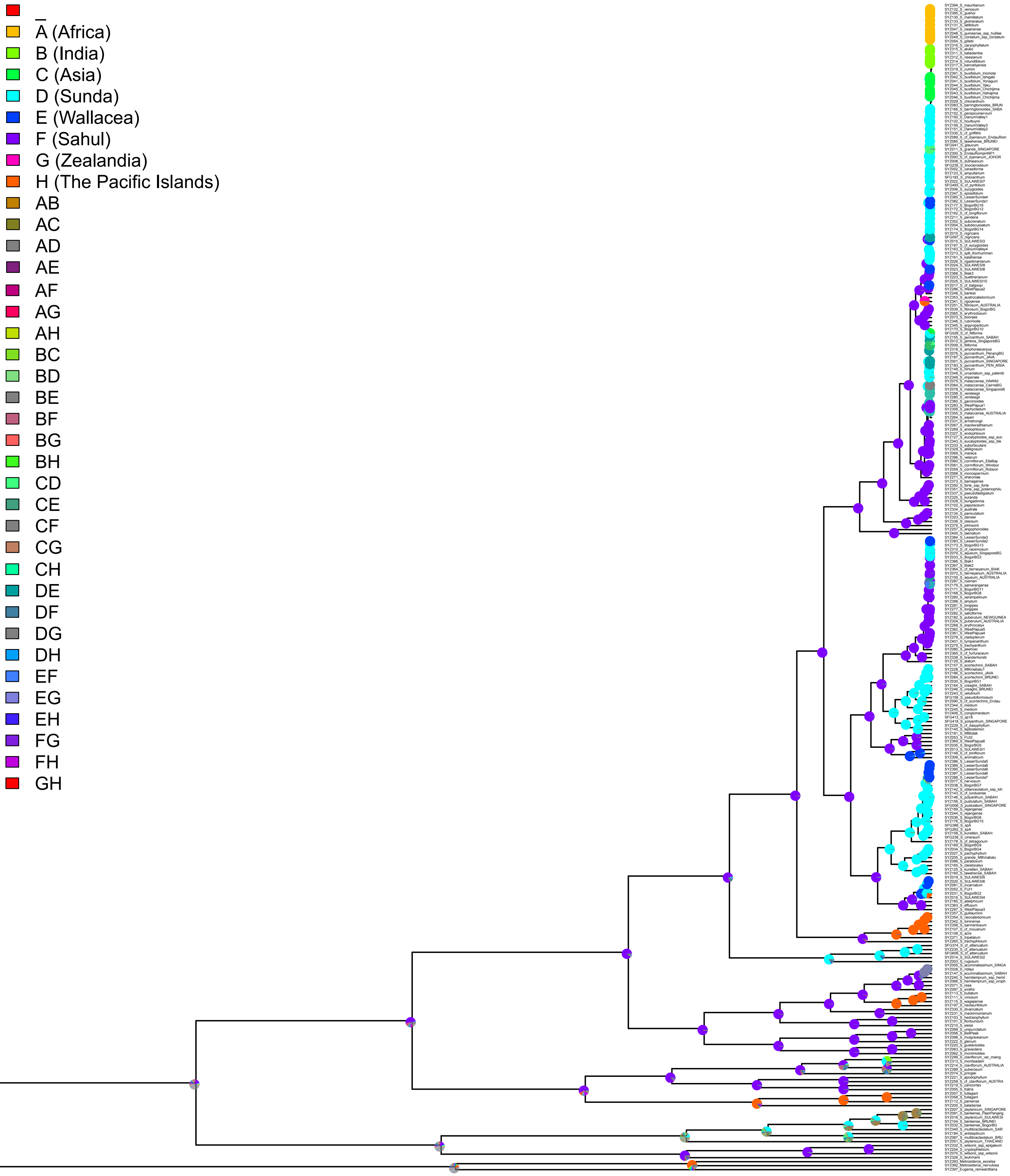

Supplementary Figure 129. Biogeographic reconstruction for the genus *Syzygium* based on BioGeoBEARS software. Analysis based on the SNP tree.

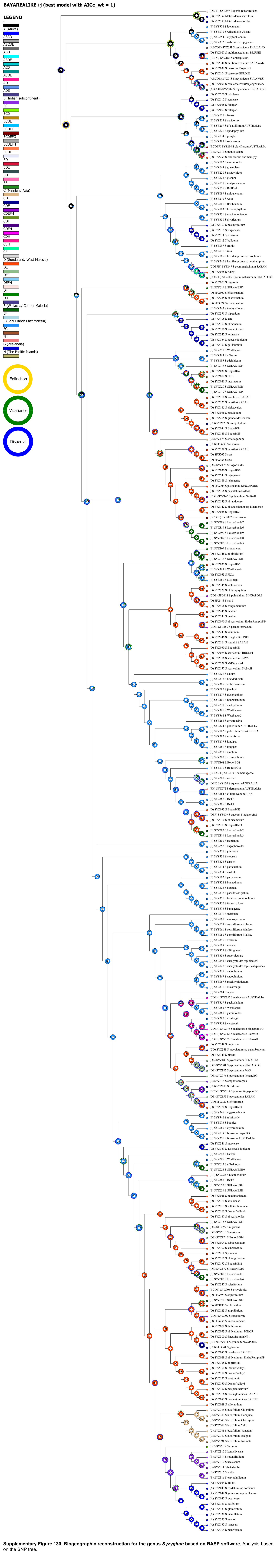

Supplementary Figure 130. Biogeographic reconstruction for the genus *Syzygium* based on RASP software. Analysis based on the SNP tree.

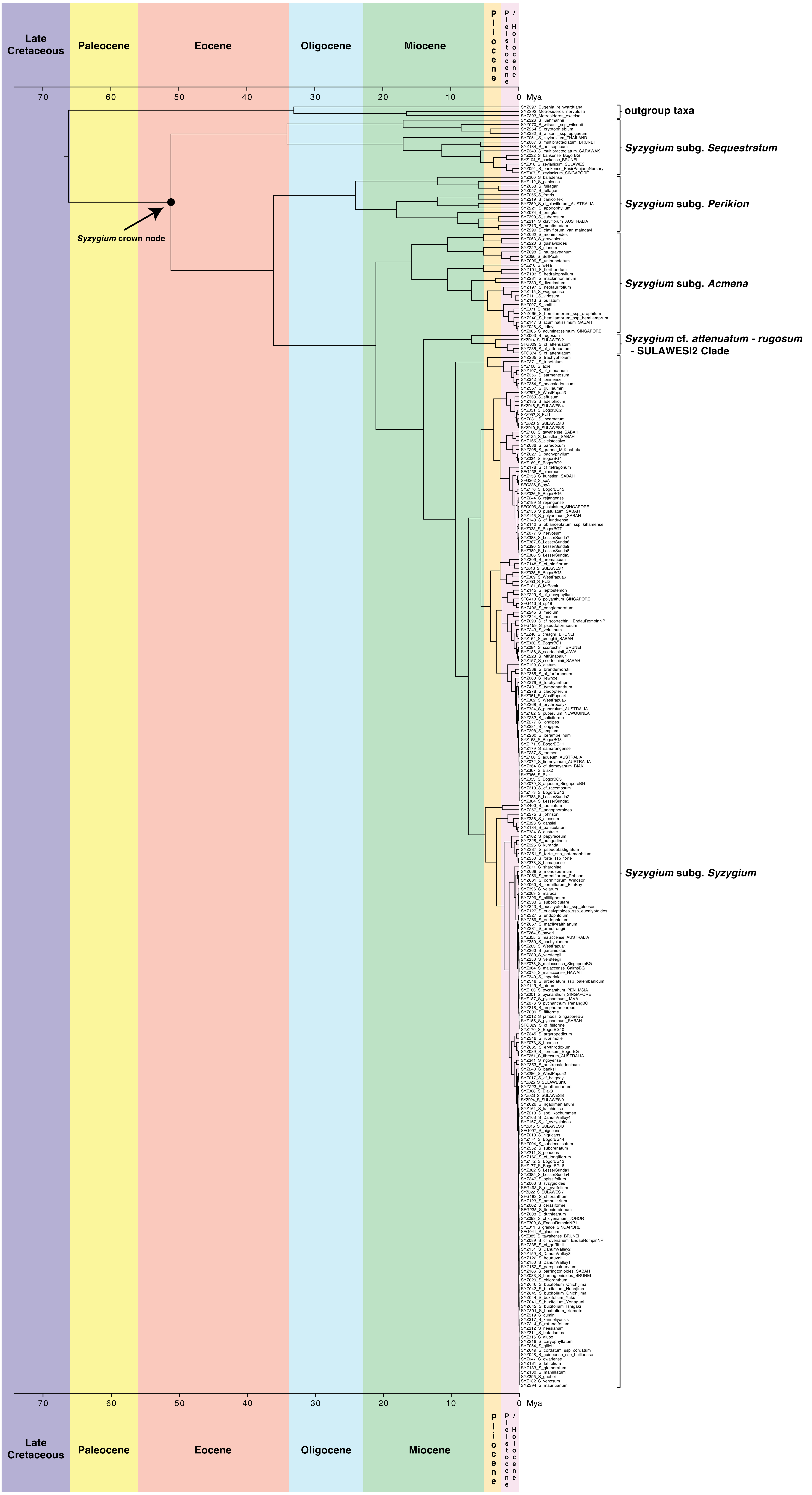

Supplementary Figure 131. Time-calibrated SNP tree for the 292 resequenced Myrtaceae accessions. Absolute time, geological periods, and taxonomic groups are indicated.

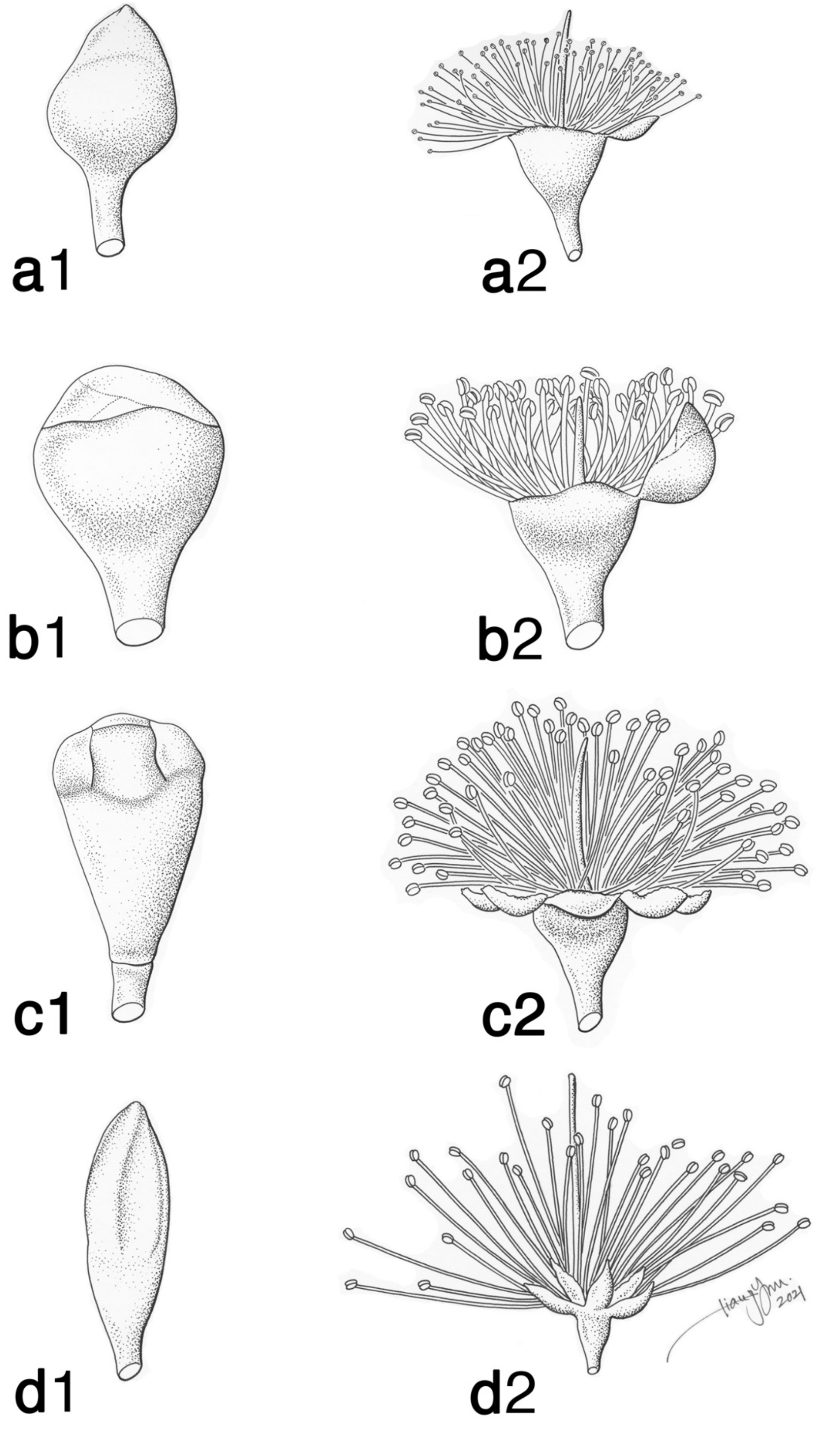

Supplementary Figure 132. Perianth traits used for morphological character-state optimisations with Mesquite. See Supplementary Note 2 for details.

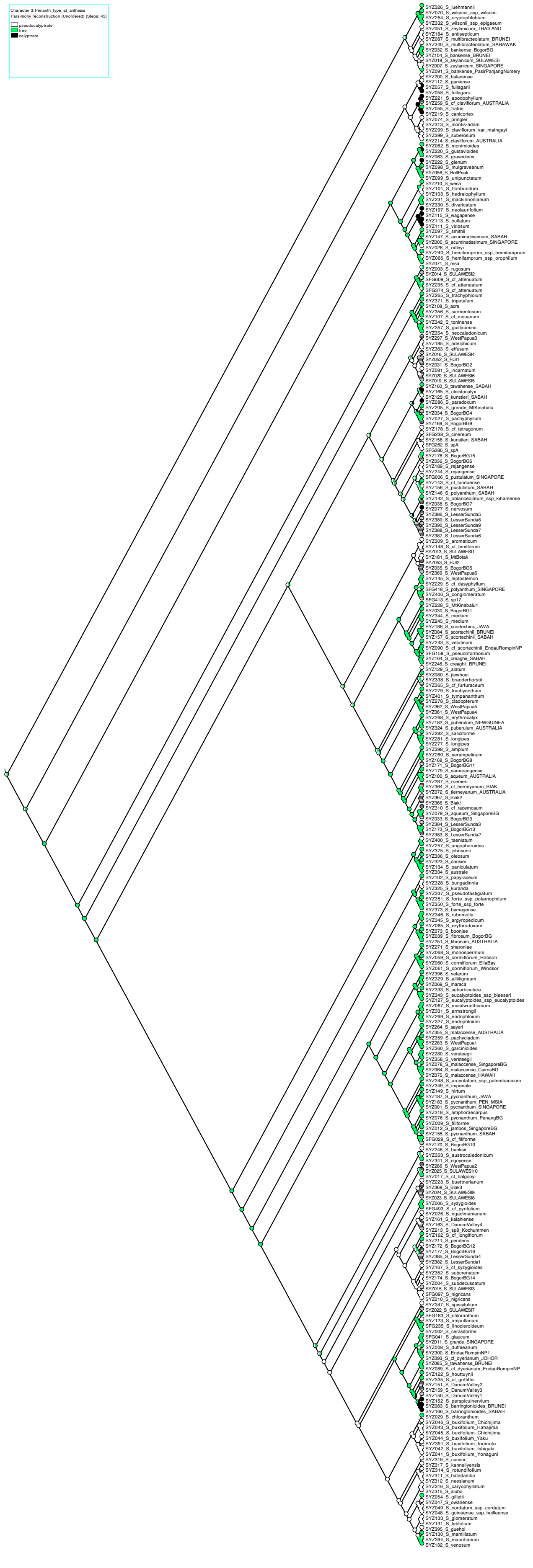

Supplementary Figure 133. Mesquite parsimony optimisation for perianth types, as shown on the BUSCO species tree. See Supplementary Fig. 132.

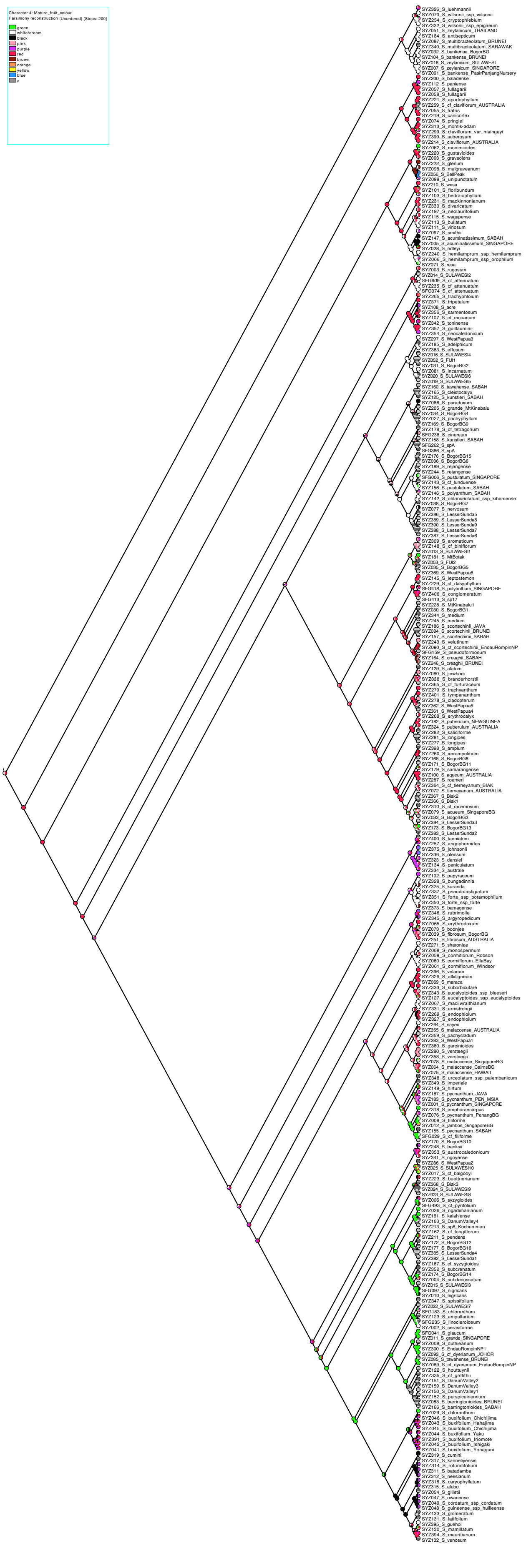

Supplementary Figure 134. Mesquite parsimony optimisation for mature fruit colour, as shown on the BUSCO species tree. See Supplementary Note 2 for details.

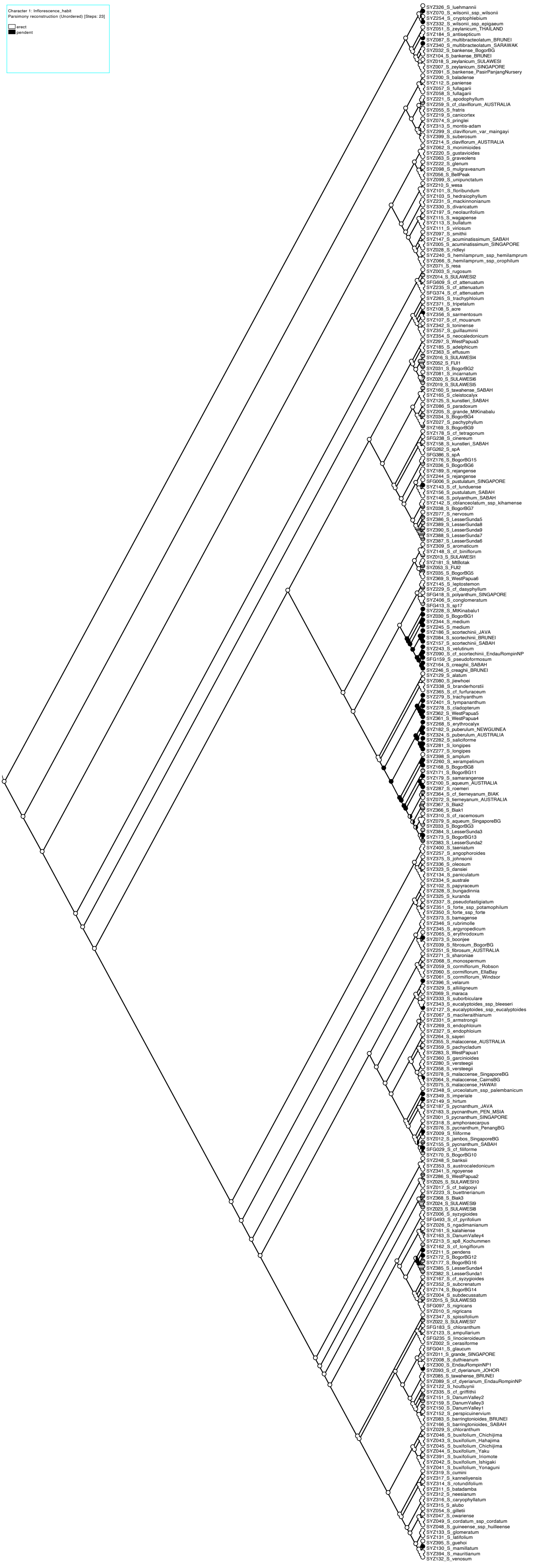

Supplementary Figure 135. Mesquite parsimony optimisation for inflorescence habit, as shown on the BUSCO species tree. See Supplementary Note 2 for details.

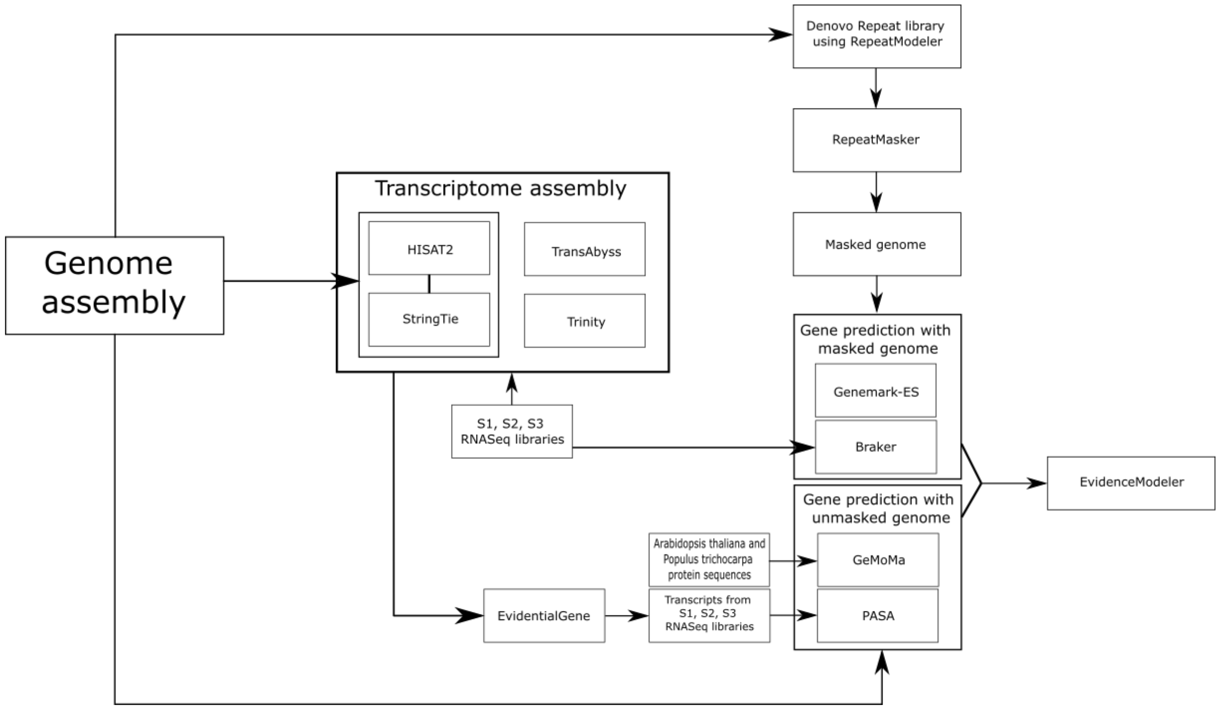

Supplementary Figure 136. Workflow diagram to describe genome annotation pipeline. The workflow includes applications used and steps taken.

C. Supplementary Tables

Supplementary Table 1. Summary of repetitive DNA content annotated in the *Syzygium grande* reference genome assembly.

Class	Count	bpMasked	%masked
=====			
**DNA**			
CMC-EnSpm	1899	2202678	0.54%
MULE-MuDR	5294	2055064	0.51%
PIF-Harbinger	614	574480	0.14%
hAT-Ac	2290	1570501	0.39%
**LINE**			
L1	5521	5378442	1.33%
**LTR**			
Caulimovirus	1203	1965098	0.49%
Copia	25199	22088800	5.45%
Gypsy	40940	48814122	12.05%
**RC**			
Helitron	2996	1874534	0.46%
Unknown	278456	89889423	22.19%
	-------------	-------------	-------------
total interspersed	364412	176413142	43.55%
Low_complexity	15055	733371	0.18%
Simple_repeat	100059	5516132	1.36%
	-------------	-------------	-------------
Total	479526	182662645	45.09%

Supplementary Table 2. SNP datasets used for various population genomic and phylogenetic analyses.

Dataset	Samples	Filters	Downstream analyses	# of sites
FRSA-GATK	ALL	Indels removed, GATK best guidelines filtering	Heterozygosity	56,164,035
FRSA-1	ALL	Indels removed, no missing data, Depth 5-500, mac 2, min GQ 30, GATK best guidelines filtering	RAxML,PCA, ADMIXTURE, Heterozygosity	1,867,173
FRSA-2	ALL but 3 outgroups	Indels removed, no missing data, Depth 5-500, mac 2, min GQ 30, GATK best guidelines filtering	RAxML, PCA, ADMIXTURE	2,273,619
FRSA-3	Only subgenus Syzygium with rugosum SYZ3 outgroup	Indels removed, no missing data, Depth 5-500, mac 2, min GQ 30, GATK best guidelines filtering	RAxML, PCA, ADMIXTURE	2,343,383
FRSA-5	Only subgenus Syzygium with rugosum SYZ3 outgroup	Indels removed, Depth 5-500, mac 2, min GQ 30, GATK best guidelines filtering	f3 statistics	22,601,275

Supplementary Table 3. *Syzygium* generation time data obtained from internet sources.

Species	Years	Link
Syzygium aqueum	2 to 3	https://www.nparks.gov.sg/florafaunaweb/flora/3/1/3153
Syzygium aromaticum	3 to 4	https://www.hindawi.com/journals/scientifica/2020/9701701/
Syzygium cormiflorum	8 to 12	http://anpsa.org.au/s-cor.html
Syzygium aromaticum	8 to 10	http://apps.worldagroforestry.org/treedb/AFTPDFS/Syzygium_aromaticum.PDF
Syzygium buxifolium	5 years	https://academic.oup.com/jpe/article/10/1/222/2966828
Syzygium cumini	8 to 10	https://www.pubhort.org/ejhs/86/4/4/86_4_4.pdf
Syzygium cumini	4 to 5;<br>8 to 10	https://askthegreengenie.com/tropical-plums/jamun-plum/#:~:text=Black%20plums%20begin%20to%20bear,will%20produce%20fruit%20into%20October.
Syzygium jambos	4	http://apps.worldagroforestry.org/treedb/AFTPDFS/Syzygium_jambos.PDF
Syzygium jambos	4	https://pfaf.org/user/Plant.aspx?LatinName=Syzygium+jambos#:~:text=Trees%20coppice%20very%20vigorously%5B790,plants%5B303%20%2C%20790%20%5D.
Syzygium malaccense	7 to 8	https://www.doc-developpement-durable.org/file/Culture/Arbres-Bois-de-Rapport-Reforestation/FICHES_ARBRES/Arbres-non-classes/Syzygium-Malayapple.pdf
Syzygium paniculatum	4 to 8	https://www.wildlandsplants.com/syzygium-paniculatum
Syzygium samarangense	3 to 5	https://www.rarefruitclub.org.au/WaxApple.htm
Syzygium travancoricum	8 to 12	https://peerj.com/preprints/27043.pdf

Supplementary references

1. Davies, S.J. *et al.* ForestGEO: Understanding forest diversity and dynamics through a global observatory network. *Biological Conservation* **253**, 108907 (2021).
2. Ho, B. *et al.* The plant diversity in Bukit Timah Nature Reserve, Singapore. *Gard. Bull. Singap* **71**, 41-144 (2019).
