# Supplementary material for: Genomic insights into rapid speciation within the world’s largest tree genus Syzygium
Source: Nat Commun. 2022 Sep 12;13:5031. doi: 10.1038/s41467-022-32637-x (PMC9468008; doi:10.1038/s41467-022-32637-x)
Supplement: Supplementary file 6 — Reporting Summary [file 41467_2022_32637_MOESM6_ESM.pdf]

nature portfolio

Corresponding author(s): Yee Wen Low

Last updated by author(s): 07/07/2022

Reporting Summary

Nature Portfolio wishes to improve the reproducibility of the work that we publish. This form provides structure for consistency and transparency in reporting. For further information on Nature Portfolio policies, see our Editorial Policies and the Editorial Policy Checklist.

Statistics

For all statistical analyses, confirm that the following items are present in the figure legend, table legend, main text, or Methods section.

n/a	Confirmed	
☒	☐	The exact sample size (*n*) for each experimental group/condition, given as a discrete number and unit of measurement
☒	☐	A statement on whether measurements were taken from distinct samples or whether the same sample was measured repeatedly
☒	☐	The statistical test(s) used AND whether they are one- or two-sided<br>*Only common tests should be described solely by name; describe more complex techniques in the Methods section.*
☒	☐	A description of all covariates tested
☒	☐	A description of any assumptions or corrections, such as tests of normality and adjustment for multiple comparisons
☒	☐	A full description of the statistical parameters including central tendency (e.g. means) or other basic estimates (e.g. regression coefficient) AND variation (e.g. standard deviation) or associated estimates of uncertainty (e.g. confidence intervals)
☒	☐	For null hypothesis testing, the test statistic (e.g. F, t, r) with confidence intervals, effect sizes, degrees of freedom and P value noted<br>*Give P values as exact values whenever suitable.*
☒	☐	For Bayesian analysis, information on the choice of priors and Markov chain Monte Carlo settings
☒	☐	For hierarchical and complex designs, identification of the appropriate level for tests and full reporting of outcomes
☒	☐	Estimates of effect sizes (e.g. Cohen's *d*, Pearson's *r*), indicating how they were calculated

Our web collection on statistics for biologists contains articles on many of the points above.

Software and code

Policy information about availability of computer code

Data collection	N/A
Data analysis	Software/packages/web-tools used (including software incorporated into CoGe [genomevolution.org], with version numbers if applicable): ASTRAL v5.15.1 , BCFtools 1.11, BEDTools 2.23., Bedops 2.4.38, BioGeoBEARS 1.1.2, braker 2.1.2, BUSCO v4.0.2, bwa mem 0.7.17), CodeML (CoGe), eigensoft 6.1.3 , EVidenceModeler 1.1.1, EvidentialGene v2018.06.18, FigTree 1.4.3, FractBias (CoGe), GATK version 3.8 , GeMoMa 1.6.1, genemark-es 4.46, ggplot2 3.3.3, ggpmisc 0.3.9, ggridges 0.5.3, HISAT2 2.1.0, HTSlib 1.11, LiftoverVcf 2.23.4, lostruct 0.0.0.9000, MAFFT 7.407, MaSuRCA 3.3.1 , Minimap2 2.20, pandoc 2.16.1, PASA 2.3.3, Picard MarkDuplicates 2.7.1, PSMC 0.6.5, Psych 2.2.3, qp3pop 650 of AdmixTools, Quota Align (CoGe), R 4.0.2, RASP 4.2, RAxML 8.2.12, RColorBrewer 1.1.2, RepeatMasker 4.0.9, RepeatModeler 1.0.11, samtools 0.1.19, scatterpie 0.1.6, SplitsTree 4.17.0, StringTie 2.0, SynMap (CoGe), tidyverse 1.3.1, TransAbyss 2.0.1, transanno 0.2.4, Trimmomatic 0.38, Trinity 2.8.5, VCFtools 0.1.16

For manuscripts utilizing custom algorithms or software that are central to the research but not yet described in published literature, software must be made available to editors and reviewers. We strongly encourage code deposition in a community repository (e.g. GitHub). See the Nature Portfolio guidelines for submitting code & software for further information.

Data

Policy information about availability of data

All manuscripts must include a data availability statement. This statement should provide the following information, where applicable:

- Accession codes, unique identifiers, or web links for publicly available datasets
- A description of any restrictions on data availability
- For clinical datasets or third party data, please ensure that the statement adheres to our policy

The genome data generated in this study has been deposited in the NCBI database under accession code PRJNA803434 and BioSample ID SAMN29207412. The Syzygium grande genome assembly and annotation are also available on CoGe at https://genomevolution.org/coge/GenomeInfo.pl?gid=60239. Processed data generated in this study and used for main text figures are provided here in source data files. Additional processed data are available at Dryad (https://doi.org/10.5061/dryad.h18931zpw).

Human research participants

Policy information about studies involving human research participants and Sex and Gender in Research.

Reporting on sex and gender	N/A
Population characteristics	N/A
Recruitment	N/A
Ethics oversight	N/A

Note that full information on the approval of the study protocol must also be provided in the manuscript.

Field-specific reporting

Please select the one below that is the best fit for your research. If you are not sure, read the appropriate sections before making your selection.

☐ Life sciences ☐ Behavioural & social sciences ☒ Ecological, evolutionary & environmental sciences

For a reference copy of the document with all sections, see nature.com/documents/nr-reporting-summary-flat.pdf

Ecological, evolutionary & environmental sciences study design

All studies must disclose on these points even when the disclosure is negative.

Study description	We sequenced the Syzygium grande genome using Oxford Nanopore long reads and sampled and Illumina-sequenced 292 additional accessions (including 289 Syzygium and 3 outgroup taxa) to understand evolutionary relationships within the genus Syzygium.
Research sample	We sequenced and assembled the Syzygium grande genome from a single individual and obtained RNAseq data from the same plant for gene annotation purposes. Syzygium and outgroup samples were selected for study based on special consideration to the broad range of the genus in terms of morphology, geography, habitat and phylogeny.
Sampling strategy	Syzygium and outgroup samples were selected for study based on availability of material and special consideration to the broad range of the genus in terms of morphology, geography, habitat, and phylogeny.
Data collection	High-molecular weight DNA was obtained from flash frozen mature leaves and RNA from various tissues (young twig, leaf early development and leaf late development). DNA of resequenced samples were obtained from mature leaves gathered in the field and preserved in silica gel granules.
Timing and spatial scale	Tropical plants (at maturity), including Syzygium species, flower throughout the year but peaked at the end of dry season (typically between November to February and March to April). Fieldwork for this project started in February 2017 and ended in July 2019.
Data exclusions	None.
Reproducibility	Molecular, morphological and biogeographic data used in this study are publicly available for reproducibility.
Randomization	Not applicable for genome data.
Blinding	Not applicable for genome data.

Did the study involve field work? [x] Yes [ ] No

Field work, collection and transport

Field conditions	Warm and humid tropical climates with temperatures averaging between 21 to 30 degrees Celsius. Annual precipitation in the field-collection regions is about 200 to 250 centimetres (80 to 180 inches) per year.
Location	Singapore, Africa (Angola and Congo), Australia (Northern Territory and Queensland), Brunei, Fiji (Viti Levu), Indonesia (Java, Papua, Papua Barat, Sulawesi and Sumbawa), Japan (Okinawa and Tokyo), Malaysia (Peninsular Malaysia, Sabah and Sarawak), New Caledonia, Sri Lanka, Thailand (Prachin Buri Province), Vietnam (Lam Dong Province)
Access & import/export	The following coauthors have all required research permits or permissions granted to collect the various Syzygium specimens in the field:<br>(1) Yee Wen Low – Collecting permit issued by the National Parks Board, Singapore (NP/RP17-098-1); Indonesian Ministry of Research and technology (RISTEK) issued a research permit (Application ID: 1517217008) and permission to perform fieldwork in Indonesian New Guinea; permission to collect plant specimens under the MoU signed between the National Parks Board, Singapore and the Brunei Forestry Department, Brunei; and Access License issued by the Sabah State Government, Malaysia [JKM/MBS.1000-2/2JLD.7 (84)].<br>(2) Parusuraman Athen and Serena Lee: Permission to collect at the Lam Dong Province, Vietnam was made possible under the MoU signed between the National Parks Board, Singapore and the Bidoup Nui-Ba National Park, Dalat, Vietnam.<br>(3) Ruth Bone - Fieldwork in Fiji was hosted and facilitated by Elina Nabubuniyaka-Young (The Pacific Community's Centre for Pacific Crops and Trees, Fiji).<br>(4) Martin Cheek - National Herbarium of Republic of Congo (IEC) for authorisation for botanical prospection and export of material to the Royal Botanic Gardens, Kew.<br>(5) Ian Cowie –Northern Territory Syzygium materials collected under the Northern Territory Herbarium (DNA), Department of Environment Parks and Water Security, Northern Territory, Australia.<br>(6) Darren Crayn, Bruce Gray and Stuart Worboys – Queensland Syzygium materials collected under permit PTU18-001474.<br>(7) David Goyder – Collections Unit INBAC of the Angolan Ministry of the Environment in Luanda authorised permits and permission.<br>(8) Himesh Jayasinghe – Permission to collect issued by the Department of Wildlife Conservation in collaboration with the National Herbarium (PDA), Department of National Botanic Gardens, Sri Lanka.<br>(9) Akiyo Naiki – Obtained permission to collect Syzygium buxifolium in cultivation at the Koishikawa Botanical Garden, University of Tokyo and the Herbarium of the Iriomote Station, Tropical Biosphere Research Center, University of the Ryukyus, Okinawa.<br>(10) Liam Trethowan – Permission to perform fieldwork in Sulawesi and Sumbawa was granted by the Ministry for Research and Technology (RisTek), Indonesia and the Material Transfer Agreement (MTA) was facilitated through Herbarium Bogoriense under the MoU signed between the Royal Botanic Gardens, Kew and Herbarium Bogoriense.<br>(11) Thais Vasconcelos - Assemblée de la Province Nord and Assemblée de la Province Sud facilitated permission to collect at New Caledonia.
Disturbance	Only small leaf samples and non-destructive herbarium collections were collected.

Reporting for specific materials, systems and methods

We require information from authors about some types of materials, experimental systems and methods used in many studies. Here, indicate whether each material, system or method listed is relevant to your study. If you are not sure if a list item applies to your research, read the appropriate section before selecting a response.

Materials & experimental systems

n/a	Involved in the study	
[x]	[ ]	Antibodies
[x]	[ ]	Eukaryotic cell lines
[x]	[ ]	Palaeontology and archaeology
[ ]	[x]	Animals and other organisms
[x]	[ ]	Clinical data
[x]	[ ]	Dual use research of concern

Methods

n/a	Involved in the study	
[x]	[ ]	ChIP-seq
[x]	[ ]	Flow cytometry
[x]	[ ]	MRI-based neuroimaging

Animals and other research organisms

Policy information about studies involving animals; ARRIVE guidelines recommended for reporting animal research, and Sex and Gender in Research

Laboratory animals	N/A
Wild animals	N/A
Reporting on sex	N/A
Field-collected samples	All leaf samples were collected from herbarium accessions, or from fresh leaf material into silica gel powder and stored at room temperature until use.
Ethics oversight	No ethical guidance was required.

Note that full information on the approval of the study protocol must also be provided in the manuscript.
